# Iterative Chemical Engineering of Vancomycin Leads to Novel Vancomycin Analogs With a High *in Vitro* Therapeutic Index

**DOI:** 10.3389/fmicb.2018.01175

**Published:** 2018-06-07

**Authors:** Nigam M. Mishra, Izabela Stolarzewicz, David Cannaerts, Joris Schuermans, Rob Lavigne, Yannick Looz, Bart Landuyt, Liliane Schoofs, Dominique Schols, Jan Paeshuyse, Peter Hickenbotham, Martha Clokie, Walter Luyten, Erik V. Van der Eycken, Yves Briers

**Affiliations:** ^1^Laboratory for Organic and Microwave-Assisted Chemistry, Department of Chemistry, KU Leuven, Leuven, Belgium; ^2^Department of Chemistry, Warsaw University of Life Sciences, Warsaw, Poland; ^3^Laboratory of Gene Technology, Department of Biosystems, KU Leuven, Leuven, Belgium; ^4^Laboratory of Functional Genomics and Proteomics, Department of Biology, KU Leuven, Leuven, Belgium; ^5^Laboratory of Virology and Chemotherapy, Rega Institute, Department of Microbiology and Immunology, KU Leuven, Leuven, Belgium; ^6^Laboratory for Host Pathogen Interactions, Department of Biosystems, KU Leuven, Leuven, Belgium; ^7^Department of Infection, Immunity and Inflammation, University of Leicester, Leicester, United Kingdom; ^8^Department of Pharmaceutical and Pharmacological Sciences, KU Leuven, Leuven, Belgium; ^9^Department of Organic Chemistry, Peoples’ Friendship University of Russia (RUDN University), Moscow, Russia; ^10^Laboratory of Applied Biotechnology, Department of Biotechnology, Ghent University, Ghent, Belgium

**Keywords:** vancomycin analog, VRE, MRSA, resistance, chemical engineering, *in vitro* therapeutic index

## Abstract

Vancomycin is a glycopeptide antibiotic that inhibits transpeptidation during cell wall synthesis by binding to the D-Ala-D-Ala termini of lipid II. For long, it has been used as a last resort antibiotic. However, since the emergence of the first vancomycin-resistant enterococci in 1987, vancomycin resistance has become widespread, especially in hospitals. We have synthesized and evaluated 110 vancomycin analogs modified at the C-terminal carboxyl group of the heptapeptide moiety with R_2_NHR_1_NH_2_ substituents. Through iterative optimizations of the substituents, we identified vancomycin analogs that fully restore (or even exceed) the original inhibitory activity against vancomycin-resistant enterococci (VRE), vancomycin-intermediate (VISA) and vancomycin-resistant *Staphylococcus aureus* (VRSA) strains. The best analogs have improved growth inhibitory activity and *in vitro* therapeutic indices against a broad set of VRE and methicillin-resistant *S. aureus* (MRSA) isolates. They also exceed the activity of vancomycin against *Clostridium difficile* ribotypes. Vanc-39 and Vanc-42 have a low probability to provoke antibiotic resistance, and overcome different vancomycin resistance mechanisms (VanA, VanB, and VanC1).

## Introduction

Vancomycin is a glycopeptide antibiotic that is widely used for the treatment of life-threatening infections caused by Gram-positive methicillin-resistant *Staphylococcus aureus* (MRSA), enterococci and *Clostridium difficile*, and which are unresponsive to other antibiotics. Vancomycin is also the antibiotic-of-choice for patients who are allergic to β-lactam antibiotics (Ingerman and Santoro, 1989). Vancomycin exerts its antibacterial effect through inhibition of cell wall crosslinking by binding to the terminal D-Ala-D-Ala residues of membrane-anchored lipid II (Walsh, 1999). Despite the fact that vancomycin was kept as a drug of last resort, enterococci have developed extensive resistance (vancomycin-resistant enterococci or VRE). Altered lipid II with terminal D-Ala-D-Lac is the most prevalent resistance mechanism of VRE (VanA and VanB type), leading to the loss of one hydrogen bond and a consequent 1000-fold reduced affinity for vancomycin (Arthur and Courvalin, 1993; Walsh et al., 1996). Indeed, interaction of vancomycin to the terminal D-Ala-D-Ala is mediated through hydrogen bonds with the heptapeptide backbone. In addition, *N*-methylleucine, which is the first amino acid of the heptapeptide backbone, does not take part in hydrogen bonding but is essential for an intact binding pocket as shown by molecular dynamics simulations (Wang et al., 2018). Other resistance mechanisms imply the formation of D-Ala-D-Ser termini (VanC, VanE, and VanG) (Reynolds et al., 1994; McKessar et al., 2000; Abadía Patiño et al., 2002). Vancomycin is also frequently used for the treatment of MRSA infections, and vancomycin-resistant strains have been reported. Vancomycin-intermediate *S. aureus* (VISA) strains have a thickened and poorly crosslinked cell wall with abundant D-Ala-D-Ala sites sequestering vancomycin (Cui et al., 2003). Vancomycin-resistant *S. aureus* (VRSA) strains have a similar resistance mechanism as VRE due to horizontal transfer of the *vanA* operon.

In response to the emergence of vancomycin-resistant strains, second-generation lipoglycopeptides have been designed to combat vancomycin-resistant strains. Vancomycin has been chemically modified in a semisynthetic approach either at the N- or C-terminus of the heptapeptide backbone, or at the vancosamine group in the carbohydrate moiety. These are non-binding site modifications, and thus generally do not affect the binding affinity with the terminal residues of lipid II. Accordingly, alternative mode of actions have been attributed to different groups of vancomycin analogs. Dimerization of some vancomycin analogs is achieved through hydrophobic substitutions (e.g., lipidation) at the vancosamine group and appears to result in cooperative binding of the lipid II termini (Allen et al., 1996). These substitutions also increase membrane anchoring, which may conceivably result in a better binding of the vancomycin analog to the lipid II termini. Other vancomycin analogs are suggested to show improved bactericidal activity by membrane depolarization, e.g., through coupling a lipophilic cationic quaternary ammonium moiety to the C-terminus of vancomycin (Yarlagadda et al., 2014). Chlorobiphenyl (CBP) substituents on the disaccharide result in analogs that do not bind D-Ala-D-Lac but directly inhibit transglycosylase activity through enzyme binding (Chen et al., 2003). In spite of the efficacy of modifications with lipophilic substituents, the latter may also result in long elimination half-lifes and accumulative toxicity. Recently, Guan et al. (2018) reported that the introduction of hydrophilic substituents like sugar moieties counteracts the negative effects of lipophilic substituents, resulting in an enhanced efficacy, optimal pharmacokinetics and a lower toxicity. Modifications in the core structure of vancomycin, which comprises the binding site of vancomycin to D-Ala-D-Ala, have remained largely unexplored, as this requires total synthesis of the vancomycin analogs. This is particularly challenging given the structural complexity of vancomycin. However, Crowley and Boger (2006) succeeded in reengineering the binding pocket for balanced, dual binding characteristics to both D-Ala-D-Ala and D-Ala-D-Lac using total synthesis. Parallel to this study, the same group recently presented several vancomycin analogs with synergistically acting modifications at the heptapeptide backbone, the C-terminus (cationic quaternary ammonium salt), and the vancomycin disaccharide (CBP substituent), resulting in more durable antibiotics with less propensity to acquire resistance against the three mechanisms of actions (Okano et al., 2017a). A complete review on the total syntheses of vancomycin-related glycopeptide antibiotics including their modifications and key analogs has been published by the Boger group (Okano et al., 2017b). Oritavancin (a chlorophenylbenzyl derivative of chloroeremomycin, a vancomycin-like glycopeptide), televancin (derived from vancomycin with substituents on the vancosamine group and the peptide core) and dalbavancin (a semi-synthetic derivative of the natural teicoplanin-like lipoglycopeptide A-40926) are three FDA-approved semisynthetic second-generation glycolpeptides.

Another approach is the synthesis of bifunctional hybrids such as vancomycin-nisin conjugates. These conjugates were found to show improved antibacterial activity against VRE compared to the unconjugated molecules (Arnusch et al., 2008). We previously reported the synthesis of conjugates of vancomycin and cathelicidin-related antimicrobial peptide (CRAMP) using click chemistry with diverse hydrophilic and hydrophobic linkers. Our hypothesis was that CRAMP could act as a carrier molecule for the transfer of the vancomycin moiety through the outer membrane of Gram-negative bacteria, conferring antibacterial activity against both Gram-positive and Gram-negative bacteria to the conjugates. Small hydrophobic linkers with an aromatic group eventually resulted in the most active conjugates against planktonic Gram-negative bacteria, while maintaining the high activity of vancomycin against Gram-positive bacteria (Mishra et al., 2015). In an attempt to understand these findings, we synthesized vancomycin analogs substituted with the linker molecules corresponding to our previous study, but without the CRAMP moiety. These analogs lost their activity against Gram-negative species, but serendipitously, we found that the vancomycin analogs with the hydrophobic substituents showed a strongly increased inhibitory activity against VRE. These initial analogs with hydrophobic substituents proved, however, highly toxic for cell lines *in vitro*, presumably due to the increased hydrophobicity. We therefore initiated a program to synthesize novel vancomycin analogs in iterative rounds to gradually find an optimal balance between hydrophobicity and hydrophilicity of the substituent, and identified analogs with superior antibacterial activity against VRE, MRSA and *C. difficile* without *in vitro* cytotoxicity.

## Materials and Methods

### Synthesis of Vancomycin Analogs

All vancomycin analogs were designed and synthesized by amide coupling. The substituents (R^2^NHR^1^) were synthesized in two or more synthetic steps with good yield. These R^2^NHR^1^NH_2_ moieties were attached to the C-terminus of the heptapeptide backbone of the vancomycin. For the amidation of the glycopeptide, coupling reagents EDC⋅HCl and HOAt were used to provide the target amide as predominant product. All vancomycin derivatives were purified by preparative HPLC. Full details on the synthesis have been added as Supplementary Information. In total, seven different synthesis rounds of new vancomycin analogs were performed.

### Bacterial Strains, Growth Conditions and Antibiotics

Vancomycin-sensitive and vancomycin-resistant *Enterococcus faecalis* strains and methicillin-resistant *S. aureus* (MRSA) strains included clinical isolates collected at the university hospital UZ Leuven (Belgium). The vancomycin-sensitive *S. aureus* strain was ATCC 6538 (Rosenbach). The vancomycine-intermediate *S. aureus* (VISA) HIP5827 (Tenover et al., 1998) and vancomycin-resistant *S. aureus* (VRSA) were kindly donated by Olivier Denis (Université Libre de Bruxelles, Belgium). *E. faecalis* and *S. aureus* strains were grown at 37°C on brain heart infusion (BHI; Becton-Dickinson, Erembodegem, Belgium) agar and lysogeny broth (LB; also known as Luria-Bertani medium) agar plates, respectively. *C. difficile* strain ATJ ribotype 014/20 and strain AIU ribotype 027 were kept on brain heart infusion agar plates, grown at 37°C in an anaerobic cabinet. Vancomycin, daptomycin, tigeglycine, and linezolid were purchased from Selleck Chemicals (Munich, Germany).

### Minimum Inhibitory Concentration and Minimum Bactericidal Concentration Against *E. faecalis* and *S. aureus*

The minimum inhibitory concentration (MIC) was determined using the broth microdilution method according to the antimicrobial susceptibility testing standards of the clinical and laboratory standards institute (Clinical and Laboratory Standards Institute [CLSI], 2012). Ultrapure water and non-inoculated broth were used as controls. In one case, a final concentration of 10 μM vancomycin was included with the vancomycin analog or control antibiotic. The MIC was determined after 24 h at 37°C as the minimum concentration that fully inhibited cell growth.

To monitor resistance development upon exposure to subinhibitory doses, the initial MIC of Vanc-39, Vanc-42 and daptomycin was determined against VRE 39. A new MIC assay was prepared using 1,000-fold diluted cells grown at MIC/2 (occasionally MIC/4 if growth in MIC/2 was too limited). This was repeated for 20 cycles and changes in MIC were monitored.

To determine the minimal bactericidal concentration (MBC), the same MIC experiment as described above was set up, and the content of wells showing no growth after 24 h was completely plated on BHI and LB agar plates for VRE and MRSA, respectively. Plates were incubated overnight at 37°C and colony growth was inspected. The compound concentration that corresponded to the well which did not contain viable bacteria is the minimum bactericidal concentration.

### Minimum Inhibitory Concentration Against *C. difficile*

Bacterial lawns of both *C. difficile* strains were prepared by adding 0.5 mL overnight culture to 8 mL sloppy BHI agar (0.4% agar) supplemented with 1.5 g/L CaCl_2_⋅2H_2_O and 81.2 g/L MgCl_2_⋅6H_2_O, and pouring the mixture into Petri plates. A twofold dilution series of the selected vancomycin analogs, vancomycin (0.012 μM) and daptomycin (25 μM) was spotted (10 μL) on the bacterial lawn. Spots were air-dried and the plates were incubated overnight at 37°C in an anaerobic cabinet. Ultrapure water was included as negative control. The assay was performed in triplicate. Inhibition was observed by cleared spots.

### Cytostatic Activity Against Mammalian Cell Lines

Murine leukemia (L1210), human T-lymphocyte (CEM) and the human cervix carcinoma (HeLa) cell line were suspended at 300,000–500,000 cells/mL of culture medium, and 100 μL of a cell suspension was added to 100 μL of an appropriate dilution of the test compounds in wells of 96-well microtiter plates. After incubation at 37°C for 2 (L1210), 3 (CEM), or 4 (HeLa) days, the cell number was determined using a Coulter counter. The IC_50_ was defined per cell type as the compound concentration required to inhibit cell proliferation by 50% compared to the negative control (Glowacka et al., 2017).

### Time-Kill Curves

Time-kill curves were determined based on the CLSI guidelines (Clinical and Laboratory Standards Institute [CLSI], 1999). Single colonies grown overnight at 37°C were resuspended in Ca^2+^-adjusted (0.05 mg/mL) Mueller Hinton broth up to an optical density (625 nm) of 0.08–00.10 (corresponding to the 0.5 McFarland standard), followed by a 10-fold dilution in the same broth. Subsequently, 0.2 mL inoculum was added to 1.7 mL Ca^2+^-adjusted (0.05 mg/mL) Mueller Hinton broth, followed by addition of the antibacterial compound (0.1 mL). The final concentration of the antibacterial compound corresponded to the 4x MIC value for that compound. For the antibiotic-free control, the compound was replaced by an equal volume of ultrapure water. The final inoculum concentration was approximately 10^6^ CFU/mL. The cultures were continuously shaken at 37°C. After 1, 2, 4, 6, 8, and 24 h, a 0.1 mL aliquot was removed, serially diluted in cold 0.9% w/v NaCl and plated on BHI (VRE) and LB (MRSA) agar plates. Plates were incubated overnight at 37°C and colonies were counted.

## Results

### Vancomycin Analogs Substituted With Hydrophobic Groups Are Active Against VRE

**FIGURE 1:**
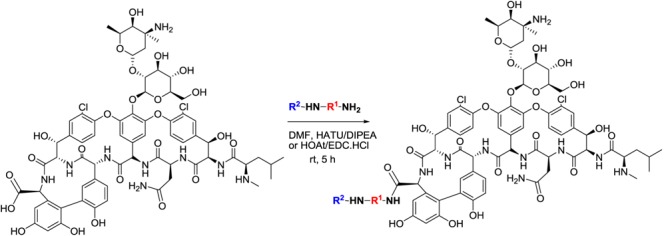
General structure of vancomycin analogs. The C-terminal carboxyl group of the peptide within the vancomycin structure is substituted with R^2^NHR^1^NH-groups.

Eighteen vancomycin analogs substituted with hydrophilic and hydrophobic groups, corresponding to the linkers between vancomycin and CRAMP used in our previous study (Mishra et al., 2015), were synthesized and purified (**Figure 1** and Supplementary Information). The minimum inhibitory concentration (MIC) was determined against a *Bacillus subtilis*, an *Escherichia coli*, a vancomycin-sensitive *E. faecalis* and two vancomycin-resistant strains (VRE6 and VRE53; both VanA type). The former two were selected as model organisms for a Gram-positive and Gram-negative bacterium, respectively, similar to our previous study. Enterococci were included to analyze the effects of substitution on vancomycin resistance. The data are expressed in μM to allow for direct comparison given the molecular weight differences between the analogs. The vancomycin analogs show a slightly decreased MIC against *B. subtilis* (0.5- to 2-fold MIC decreases) or *E. coli* (1- to 8-fold decreases) compared to vancomycin, but six substituents resulted in analogs with also a strongly decreased MIC against vancomycin-sensitive *E. faecalis* (2- to 64-fold) and vancomycin-resistant *E. faecalis* strains (4- to 256-fold) (Supplementary Table S1). These substituents are (unlike the other substituents used) all hydrophobic. Specifically, five of six vancomycin analogs (Vanc-N, Vanc-Q, Vanc-R, Vanc-S, and Vanc-T) share a (poly)aromatic substituent (aniline-glycine and benzoic acid/cinnamic/naphthoic acid derivatives) whereas the sixth, weaker compound (Vanc-B) has an aliphatic substituent (Supplementary Information).

### Iterative Rounds of Vancomycin Analog Synthesis Lead to Improved Compounds

**FIGURE 2:**
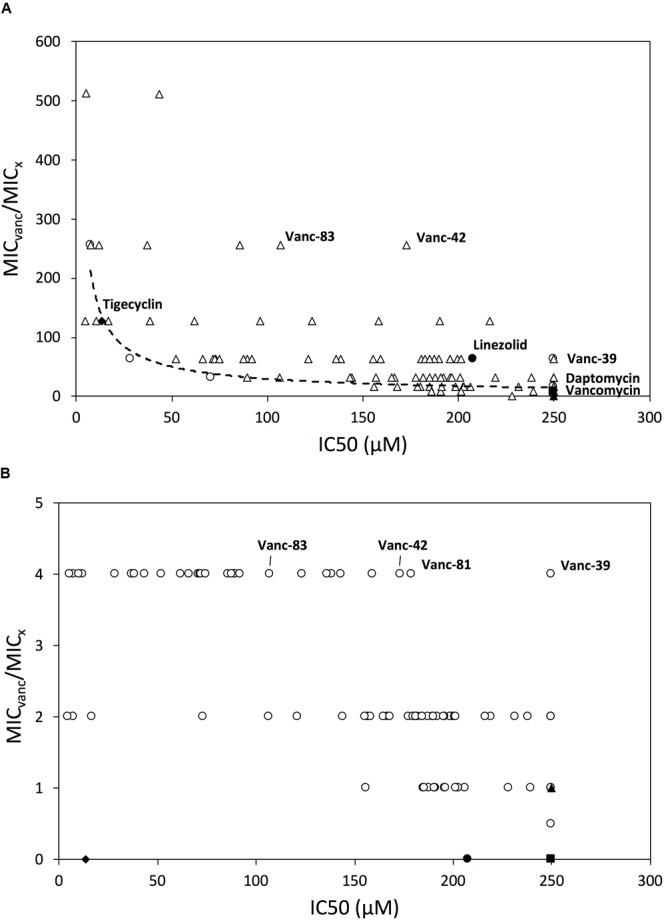
Relationship between inhibitory activity and mammalian cytotoxicity. The relationship between the IC_50_ value and MIC-fold change compared to vancomycin is shown for all vancomycin analogs and control antibiotics against VRE **(A)** and MRSA **(B)**. The IC_50_ value is the average value of the IC_50_ against the L1210, CEM and HeLa cell lines; if it is > 250 μM, then it is plotted as if it were equal to 250 μM. Symbols: first series of analogs (Vanc-B-Vanc-T) (○), other series of vancomycin analogs (Δ), tigeclycine (♦), linezolid (●), daptomycin (■) and vancomycin (▲) are shown. Vancomycin analogs selected for further characterization are highlighted.

Because hydrophobic substituents may result in undesired cytotoxic effects, the concentration that inhibits the growth *in vitro* of three cell lines (L1210, CEM, HeLa) by 50% (IC_50_) was determined. Whereas vancomycin, Vanc-B, Vanc-N, and Vanc-Q do not show toxicity (IC_50_ > 250 μM), Vanc-R, Vanc-S, and Vanc-T have low average IC_50_ values between 1.4 ± 0.6 and 15 ± 1, 5.6 ± 1.1 and 70 ± 8, and 60 ± 10 and 77 ± 17, respectively, depending on the cell line used (Supplementary Table S2). When correlating the MIC values of these six vancomycin analogs against VRE to their IC_50_ values, a generally positive correlation between inhibitory effect and toxicity is observed, with the strongest inhibitory compounds also being relatively more toxic, as illustrated for VRE29 (**Figure 2A**). In subsequent series of chemical synthesis of novel vancomycin analogs (Supplementary Information), we aimed to design molecules that have a strong inhibitory effect, but lack or have a reduced toxic effect on mammalian cells. In total, ninety-two additional vancomycin analogs were synthesized, purified and characterized. All analogs were evaluated for their inhibitory effect (MIC) against a set of one vancomycin-sensitive *E. faecalis* and four vancomycin-resistant strains (all VanA type), and for their *in vitro* cytotoxic effect against the above mentioned three cell lines. Three antibiotics (tigecycline, daptomycin, and linezolid) which are currently used (off-label) against VRE infections, and vancomycin were included as controls (Supplementary Tables S2, S3). Vancomycin analogs showing a better profile (i.e., higher inhibitory effects and lower or no cytotoxicity) were identified. These superior analogs (e.g., Vanc-42 and Vanc-83, **Table 1**) show a fully restored inhibitory activity with similar MIC values for VRE strains as vancomycin for a vancomycin-sensitive strain (VSE; 1.56 μM), while IC_50_ values increased. Vanc-39 shows no cytotoxicity (IC_50_ > 250 μM), while displaying a good inhibitory effect (**Figure 2A**).

**Table 1:** Substituents of selected vancomycin analogs.

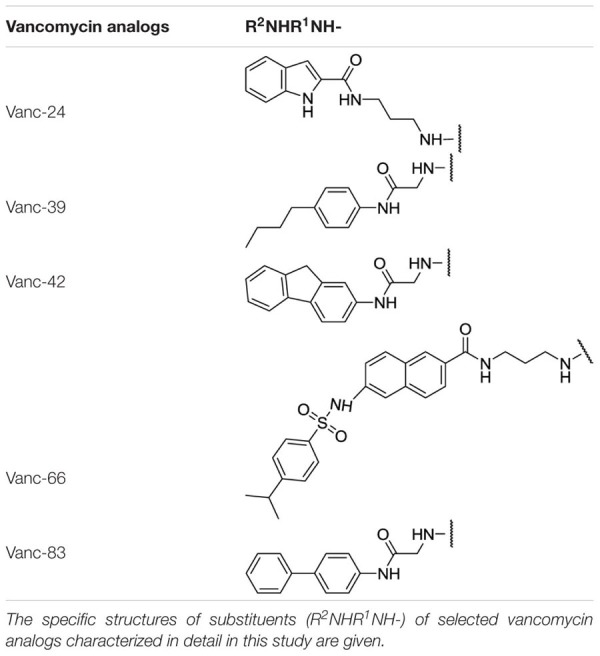

We investigated the susceptibility of a methicillin-susceptible *S. aureus* (MSSA) strain, four MRSA strains, one VISA and one VRSA strain for all vancomycin analogs and control antibiotics (Supplementary Table S4). The large majority of vancomycin analogs showed an improved inhibitory effect against MSSA (up to 16-fold) and MRSA (up to 8-fold), but mainly against VISA (up to 128-fold), and VRSA (up to 512-fold). The best vancomycin analogs against VRSA show a MIC value that is up to fourfold lower than the MIC value of unmodified vancomycin against the tested VSSA. This shows that the resistance is fully reverted. These observations for both VRSA and VRE show that the analogs either have an improved binding to D-Ala-D-Lac termini, or that the analogs have a secondary mode of action. The generally increased susceptibility of vancomycin-susceptible strains such as VSSA, MRSA and VSE support the latter hypothesis. In addition, the substituents are added at the C-terminus, which is a non-binding site for vancomycin.

Since VRSA strains are relatively rare, the correlation between the IC_50_ and MIC values was analyzed for the more prevalent MRSA strains instead. **Figure 2B** shows that Vanc-39, Vanc-42, and Vanc-83 which display a good profile against VRE strains, also perform well for MRSA. In contrast Vanc-81 performs well in case of MRSA but only shows a moderate improvement against VRE. Vanc-39, Vanc-42, and Vanc-83 were therefore selected for further analysis, together with Vanc-24 and Vanc-66, which have a more moderate profile, but differ significantly from the other selected compounds in terms of the chemical nature of the substituent (**Table 1**). Vanc-24 possesses an indole ring and Vanc-66 has 4-isopropyl-N-(naphthalen-2-yl)benzenesulfonamide moiety as R^2^ and a propyl chain as R^1^, whereas in Vanc-39, Vanc-42 and Vanc-83 R^1^ is an acetyl group. Vanc-39, Vanc-42 and Vanc-83 possess a butyl benzene, a fluorene moiety and a biphenyl ring as R^2^ substituent, respectively.

### The Best Analogs Have Improved *in Vitro* Therapeutic Indices Compared to Current Antibiotics

**FIGURE 3:**
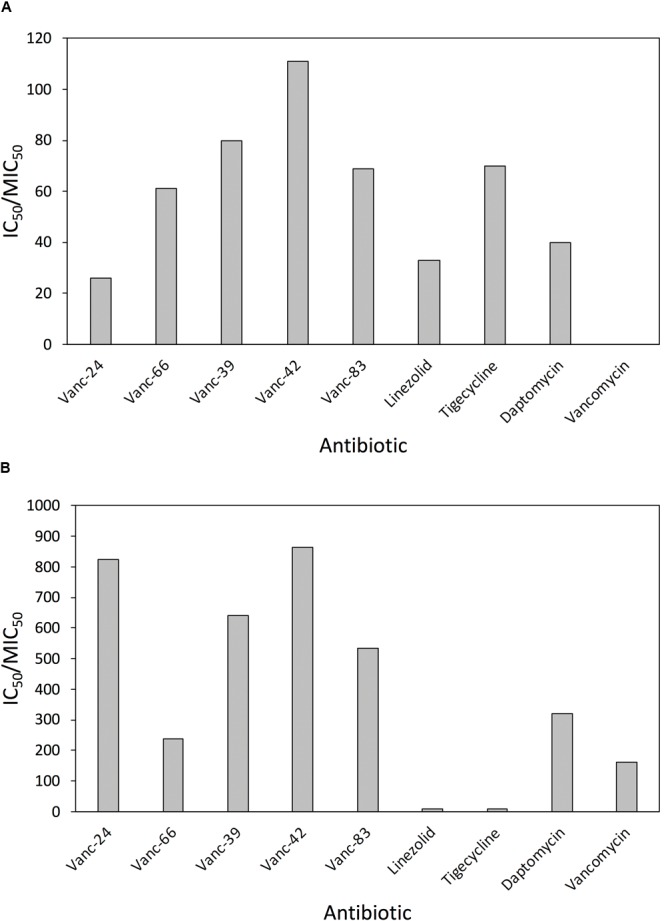
Comparison of *in vitro* therapeutic indices. The *in vitro* therapeutic indices, calculated as the ratio of IC_50_ and MIC_50_ (i.e., the median MIC value for the respective set of isolates) are compared for five selected vancomycin analogs and four control antibiotics against 33 clinical VRE **(A)** and 24 clinical MRSA **(B)** isolates.

Vanc-24, Vanc-39, Vanc-42, Vanc-66, and Vanc-83 were tested against a broad set of 33 VRE isolates and 24 MRSA isolates, along with the control antibiotics linezolid, tigecycline, daptomycin and vancomycin. The MIC_50_ value was determined as the median value for each set and used for the calculation of an *in vitro* estimator of the therapeutic index (TI = IC_50_/MIC_50_) as a reference for the window between the *in vitro* effective inhibitory and mammalian cytotoxic dose (Supplementary Table S5). Comparison of the *in vitro* therapeutic indices for VRE (**Figure 3A**) shows similar or improved values compared to current alternatives, with Vanc-42 (111) showing the best index, exceeding those of linezolid, tigecycline and daptomycine. Due to resistance, the MIC_50_ value of vancomycin is higher than its IC_50_ value, resulting in a TI < 1. In the case of MRSA, the *in vitro* TI values are much higher, peaking at 865 for Vanc-42, which is approximately 100 times higher than linezolid and tigecycline tested under the same conditions. Also other vancomycin analogs score well above an *in vitro* therapeutic index of 200 (**Figure 3B**).

### A Selection of Vancomycin Analogs Shows Improved Activity Against *C. difficile* Compared to Vancomycin

Oral vancomycin is the treatment of choice for severe *C. difficile* colitis in patients who cannot tolerate the first-choice antibiotic metronidazole (Shen and Surawicz, 2008). Therefore, we investigated a subset of vancomycin analogs against the two most prevalent *C. difficile* ribotypes (014/20 and 027). All vancomycin analogs showed an improved inhibitory effect against both ribotypes, but to a different extent. The increased inhibitory effect is largest for Vanc-42, Vanc-48, and Vanc-66 with up to 45- and 58-fold reduced MIC values for ribotype 014/20 and 027, respectively (Supplementary Table S6).

### Vancomycin Analogs Are Bactericidal Against MRSA but Bacteriostatic Against VRE

Besides the inhibitory concentrations, also the minimum bactericidal concentrations (MBCs) were determined against VRE and MRSA. In the case of the latter, Vanc-24, Vanc-39, Vanc-42, Vanc-66, Vanc-83, vancomycin and daptomycin have a MBC equaling their MIC values, showing the bactericidal action of these antibacterials against MRSA. Linezolid and tigecycline have an MBC that is more than four times higher than the corresponding MIC. For VRE, the MBC values were at least four times higher than the MIC values for all antibiotics. All compounds thus display a lower bactericidal activity against VRE compared to MRSA.

**FIGURE 4:**
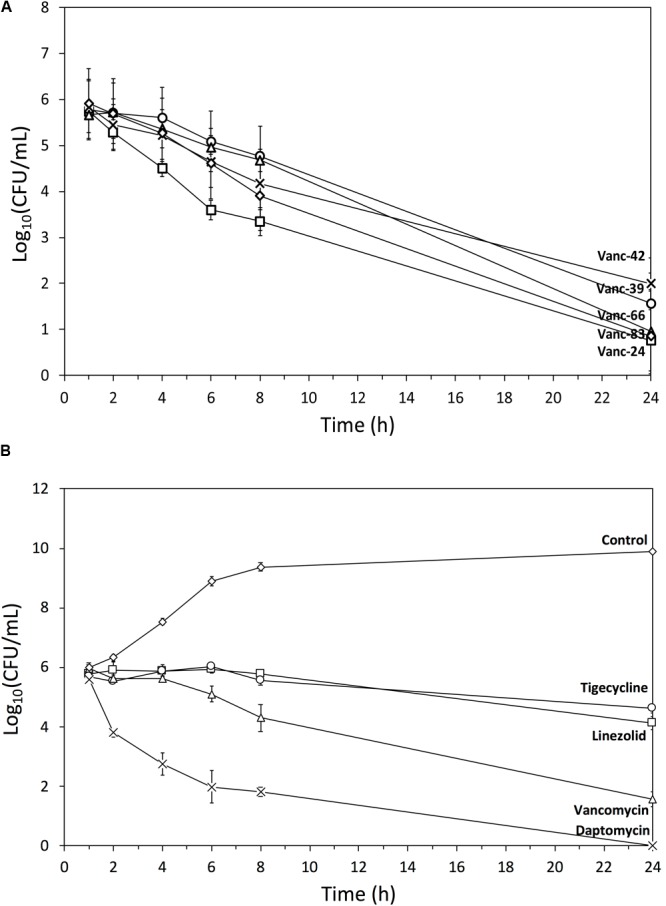
Time-kill curves. Semi-log plot of time-kill curves (*n* = 3, mean ± standard deviation) of MRSA exposed to a dose corresponding to 4x MIC of **(A)** Vanc-24 (□), Vanc-39 (○), Vanc-42 (x), Vanc-66 (Δ), and Vanc-83 (♢); **(B)** daptomycin (x), vancomycin (Δ), linezolid (□), tigecycline (○), and ultrapure water (♢) are shown.

To quantify the bactericidal effects of the five selected vancomycin analogs against MRSA, time-kill curves were determined (**Figure 4**). Stationary phase cells (10^6^ CFU/ml) were exposed to an antibiotic concentration corresponding to 4x MIC and the surviving number of cells was quantified after 1, 2, 4, 6, 8, and 24 h. All vancomycin analogs cause a relatively similar time-dependent bactericidal effect with a 4–5 log reduction after 24 h, with Vanc-24 showing the quickest onset of killing. The time-kill curves of the analogs are similar to that of vancomycin, which acts slower than daptomycin that causes a complete elimination after 24 h. Linezolid and tigecycline show a typical bacteriostatic time-kill curve.

### Vanc-39 and Vanc-42 Have a Low Probability to Provoke Antibiotic Resistance by Genetic Alterations

**FIGURE 5:**
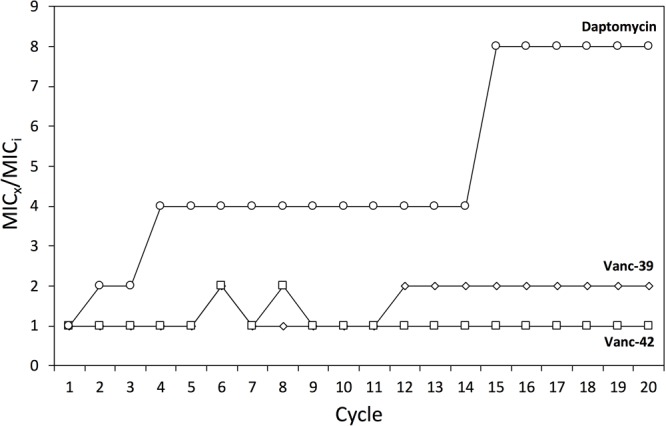
Resistance development. Changes in the MIC against Vanc-39 (♢), Vanc-42 (□) and daptomycin (○) were monitored over 20 cycles of serial exposure to subinhibitory doses.

The risk to develop resistance by genetic mutations against two selected vancomycin analogs (Vanc-39, Vanc-42) was evaluated through serial exposure of VRE to subinhibitory doses (=MIC/2). Daptomycin was included as control antibiotic. The MIC was determined after each cycle. For daptomycin, the MIC increased stepwise in cycle 2, 4, and 14 to an eightfold higher concentration, which is best explained by the putative gain of specific mutations [e.g., in the cardiolipin synthase (Palmer et al., 2011)]. The MIC values for Vanc-39 and Vanc-42 oscillated between the original value and a twofold increase, which falls within the typical variation of MIC determinations (**Figure 5**). These observations indicate that Vanc-39 and Vanc-42 have a lower probability to elicit resistance development by genetic mutations (vertical transfer) compared to daptomycin.

### The Vancomycin Analogs Overcome Different Vancomycin Resistance Mechanisms

All VRE strains analyzed for susceptibility so far have the VanA genotype with D-Ala-D-Lac termini. Therefore, five VanB- and one VanC1-type vancomycin-resistant *Enterococcus* strains were evaluated (Supplementary Table S7). VanB-type strains have also the D-Ala-D-Lac dipeptide, but differ in the regulation of the operon that leads to the production of altered peptidoglycan precursors. These strains are sensitive to teicoplanin unlike VanA-type strains. The VanC-type resistance mechanism is due to the presence of a D-Ala-D-Ser dipeptide, which has less affinity for vancomycin because of steric hindrance. The *vanC1* operon is found in *Enterococcus gallinarum*. All strains showed a similar susceptibility, albeit at the lower end of the range (Vanc-39, Vanc-42, and Vanc-83) or just below (Vanc-24 and Vanc-66) the range observed for VanA-type strains (Supplementary Table S5). The VanC1-type strain was generally slightly more susceptible than VanB-type strains, especially for Vanc-24.

We evaluated the hypothesis that vancomycin analogs may have restored activity against VRE strains due to the lack of recognition of these analogs by membrane-bound VanS. This component of the VanR-VanS two-component regulator senses the presence of vancomycin and activates transcription of the *vanA* operon leading to the hydrolysis of D-Ala-D-Ala precursors and the synthesis of D-Ala-D-Lac precursors. If the vancomycin analogs are no longer recognized, peptidoglycan precursors will not be altered, explaining why these strains remain susceptible. Therefore, the MIC of VRE37 was analyzed in the presence of 10 μM vancomycin, which is significantly above the MIC of a vancomycin-sensitive strain and is expected to induce the *vanA* gene cluster with subsequent production of D-Ala-D-Lac peptidoglycan precursors. However, no significant increases in MIC were observed (Supplementary Table S8), refuting this hypothesis. In addition, the VanC1-type strain, which constitutively expresses vancomycin resistance independent of recognition, is also susceptible to the different vancomycin analogs tested (Supplementary Table S7), confirming these findings.

## Discussion

The golden era of antibiotic discovery yielded a multitude of antibiotic classes by screening antibacterial activity of actinomycetes, via the so-called Waksman platform. Since then, many semi-synthetic derivatives of these natural antibiotics have entered the clinic. For instance, the latest variants of quinolones and cephalosporines belong to the fourth generation. Vancomycin was discovered in 1953, but remarkably few semi-synthetic derivatives have entered the market. Those that did were only recently approved, such as televancin for complicated skin and skin structure infections (2009), and hospital-acquired and ventilator-associated bacterial pneumonia (2013) caused by *S. aureus*; dalbavancin and oritavancin were recently approved for the treatment of acute bacterial skin and skin structure infections (2014). This may be explained best by (1) the complex structure of vancomycin, which was only correctly determined in 1982 almost 30 years after its discovery (Harris et al., 1983), (2) the limited number of functional groups available for modification, (3) the initial difficulties associated with total organic synthesis, which were only eliminated in the late 1990s when three independent groups successfully achieved total synthesis of vancomycin (Okano et al., 2017b) and (4) the late emergence of vancomycin resistance. Starting from our efforts to synthesize vancomycin-CRAMP hybrids that show activity against Gram-negative bacteria (Mishra et al., 2015), we serendipitously discovered vancomycin analogs that were not affected by vancomycin resistance mechanisms in enterococci. An initial series of vancomycin analogs with substituents showing large chemical diversity highlighted the potential of hydrophobic substituents to avoid vancomycin resistance mechanisms. These findings are consistent with the increased inhibitory activity of other vancomycin analogs with hydrophobic substituents, albeit with a different structure, a different attachment site and different covalent linkage (Chen et al., 2003; Mu et al., 2004; Olsuf’eva and Preobrazhenskaya, 2006; Pinchman and Boger, 2013; Printsevskaya et al., 2013; Yarlagadda et al., 2014). Iterative rounds of synthesis of novel substituents yielded vancomycin analogs with high inhibitory activity and lower or no *in vitro* toxicity for mammalian cell lines. The best analogs (e.g., Vanc-83) completely restore *in vitro* susceptibility of VRE strains to the level of susceptibility of VSE for vancomycin. Others show no *in vitro* mammalian cytotoxicity, while preserving an increased inhibitory activity against VRE (e.g., Vanc-39). We used an *in vitro* estimator of the therapeutic index to select the most promising analogs with the broadest window between the minimum inhibitory dose and mammalian cytotoxic dose. Vanc-42 was identified as the best analog on a broad set of clinical VRE isolates, and shows *in vitro* a better therapeutic index than drugs currently used for VRE treatment. Moreover, Vanc-42 shows inhibitory activity against both VanA-, VanB-, and VanC-type strains, and a low probability for resistance development by vertical transfer was demonstrated. Vanc-42 was also confirmed as a promising candidate with the best *in vitro* therapeutic index against a broad set of MRSA isolates. In addition, Vanc-42 is among the best *in vitro* inhibitors of relevant strains of the two most prevalent *C. difficile* ribotypes. Noteworthy, the best vancomycin analogs show an improved balance in hydrophobicity and hydrophilicity, with the hydrophobic moiety likely contributing to an increased efficacy against vancomycin-resistance strains, while the hydrophilic moiety appears to compensate for the observed toxicity, eventually leading to analogs with improved therapeutic indices. These findings are in agreement with a recent study by Guan et al. (2018), reporting on the development of new vancomycin analogs. They introduced extra hydrophilic sugar moieties to eliminate the toxicity induced by the lipophilic substitutions.

## Conclusion

Our study shows that the amide-based coupling of R^2^NHR^1^ substituents to vancomycin is convenient as it requires only two synthetic steps. The method can be efficiently used in an iterative approach to generate libraries of analogs with an improved *in vitro* therapeutic index against VRE, MRSA, and *C. difficile* as potential next generation vancomycin analogs for therapeutic use.

## Author Contributions

NM, IS, and DC performed the chemical engineering designed and supervised by EE. NM, JS, and YL performed the microbiological work designed and supervised by RL, BL, and YB. PH and MC designed and executed the experiments with *Clostridium difficile*. JP and DS executed the cytotoxicity assays. WL, BL, LS, RL, EE, and YB conceptualized and sponsored the project. NM, JP, WL, and YB wrote the manuscript. EE and YB coordinated the project.

## Conflict of Interest Statement

The authors declare that the research was conducted in the absence of any commercial or financial relationships that could be construed as a potential conflict of interest.
