# Supplementary material for: Iterative Chemical Engineering of Vancomycin Leads to Novel Vancomycin Analogs With a High in Vitro Therapeutic Index
Source: Front Microbiol. 2018 Jun 7;9:1175. doi: 10.3389/fmicb.2018.01175 (PMC6001238; doi:10.3389/fmicb.2018.01175)
Supplement: Supplementary file 1 [file Data_Sheet_1.docx]

**Supporting information for:**

**Iterative chemical engineering of vancomycin leads to novel vancomycin analogues with a high *in vitro* therapeutic index**

**against vancomycin-resistant strains**

Nigam M. Mishra^1, 11^, Izabela Stolarzewicz^1, 2^, David Cannaerts^1^, Joris Schuermans^3^, Rob Lavigne^3^, Yannick Looz^4^, Bart Landuyt^4^_,_ Liliane Schoofs^4^, Dominique Schols^5^, Jan Paeshuyse^6^, Peter Hickenbotham^7^, Martha Clokie^7^, Walter Luyten^4,8^, Erik V. Van der Eycken^1, 9^ and Yves Briers^3, 10,*^

^1^Laboratory for Organic & Microwave-Assisted Chemistry (LOMAC), Department of Chemistry, KU Leuven, Leuven, Belgium

^2^Department of Chemistry, Warsaw University of Life Science, Warsaw, Poland

³Laboratory of Gene Technology, Department of Biosystems, KU Leuven, Leuven, Belgium

^4^Laboratory of Functional Genomics and Proteomics, Department of Biology, KU Leuven, Leuven, Belgium

^5^Laboratory of Virology and Chemotherapy, Rega Institute, Department of Microbiology and Immunology, KU Leuven, Leuven, Belgium

^6^Laboratory for Host Pathogen Interactions, Department of Biosystems, KU Leuven, Leuven, Belgium

^7^Department of Infection, Immunity and Inflammation, University of Leicester, Leicester, United Kingdom

^8^Department of Pharmaceutical and Pharmacological Sciences, KU Leuven, Leuven, Belgium

^9^ Department of Organic Chemistry, Peoples Friendship University of Russia (RUDN University), Moscow, Russia

^10^Laboratory of Applied Biotechnology, Department of Biotechnology, Ghent University, Gent, Belgium

^11^Department of Pharmaceutical Sciences, System college of Pharmacy, University of North Texas Health Science center, Fort Worth, Texas, USA

# Corresponding author: Yves Briers, [yves.briers@ugent.be](mailto:yves.briers@ugent.be)

Table of Contents Page

1. Minimal inhibitory concentrations of first series of vancomycin analogues……….S3-S4
2. Cytotoxicity of vancomycin analogues and control antibiotics……………………………..S5-S7
3. Minimal inhibitory concentrations of all vancomycin analogues and control antibiotics against *E. faecalis*………………………………………………………………………………………………S8-S13
4. Minimal inhibitory concentrations of all vancomycin analogues and control antibiotics against *S. aureus*……………………………………………………………………………………………..S14-S19
5. *In vitro* therapeutic indices against VRE and MRSA…………………………………………..…….S20
6. Minimal inhibitory concentrations of a subset of analogues against *C. difficile*……….S21
7. Minimum inhibitory concentrations against VanB- and VanC1-type VRE strains………S22
8. General remarks………………………………………….……….……………..………………………….S24-S25
9. General experimental methods and characterisation of vancomycin intermediates……………………………………………………………………………………………….S26-S132
10. Characterisation of ^1^H NMR spectra of vancomycin analogues……………..…………………………………………………………………………………....S133-S182
11. References………………………………………………………………………………………………..…….…..S183

**Table S1. Minimal inhibitory concentrations of first series of vancomycin analogues.** The MIC values of vancomycin and 18 analogues were determined against *B. subtilis* PSB3, *E. coli* XL-1 blue MRF’ and three clinical *E. faecalis* isolates (one vancomycin-sensitive strain VSE and two vancomycin-resistant strains VRE 6 and VRE 53). Fold changes in MIC values are expressed as the ratio of the MIC of vancomycin and the MIC of an analogue against the respective strains. MIC values of the best-performing analogues against *E. faecalis* are highlighted in bold.

|  | **MIC (µM)** | | | | |  | **MICvanc/MICi** | | | | |
| --- | --- | --- | --- | --- | --- | --- | --- | --- | --- | --- | --- |
|  | ***B. subtilis* PSB3** | ***E. coli* XL-1 blue MRF'** | ***E. faecalis* VSE 1** | ***E. faecalis* VRE 6** | ***E. faecalis* VRE 53** |  | ***B. subtilis* PSB3** | ***E. coli* XL-1 blue MRF'** | ***E. faecalis* VSE 1** | ***E. faecalis* VRE 6** | ***E. faecalis* VRE 53** |
| **Vancomycin.HCl** | 0.39 | 100 | 1.56 | 200 | 400 |  | 1 | 1 | 1 | 1 | 1 |
| **Vanc-A** | 0.39 | 50 | 0.39 | 50 | 100 |  | 1 | 2 | 4 | 4 | 4 |
| **Vanc-B** | 0.20 | 25 | **0.78** | **50** | **12.5** |  | 2 | 4 | **2** | **4** | **32** |
| **Vanc-C** | 0.39 | 25 | 0.39 | 100 | 50 |  | 1 | 4 | 4 | 2 | 8 |
| **Vanc-D** | 0.39 | 12.5 | 0.78 | 100 | 50 |  | 1 | 8 | 2 | 2 | 8 |
| **Vanc-E** | 0.39 | 25 | 0.78 | 25 | 50 |  | 1 | 4 | 2 | 8 | 8 |
| **Vanc-F** | 0.20 | 25 | 0.39 | 25 | 100 |  | 2 | 4 | 4 | 8 | 4 |
| **Vanc-G** | 0.78 | 12.5 | 0.39 | 100 | 50 |  | 0.5 | 8 | 4 | 2 | 8 |
| **Vanc-J** | 0.20 | 25 | 0.78 | 200 | 100 |  | 2 | 4 | 2 | 1 | 4 |
| **Vanc-K** | 0.39 | 25 | 0.78 | 100 | 100 |  | 1 | 4 | 2 | 2 | 4 |
| **Vanc-L** | 0.78 | 50 | 0.78 | 100 | 50 |  | 0.5 | 2 | 2 | 2 | 8 |
| **Vanc-M** | 0.39 | 12.5 | 0.78 | 100 | 50 |  | 1 | 8 | 2 | 2 | 8 |
| **Vanc-N** | 0.20 | 50 | **0.10** | **3.13** | **6.25** |  | 2 | 2 | **16** | **64** | **64** |
| **Vanc-Q** | 0.39 | 12.5 | **0.10** | **6.25** | **6.25** |  | 1 | 8 | **16** | **32** | **64** |
| **Vanc-R** | 0.78 | 100 | **0.20** | **3.13** | **6.25** |  | 0.5 | 1 | **8** | **64** | **64** |
| **Vanc-S** | 0.20 | 50 | **0.03** | **0.78** | **3.13** |  | 2 | 2 | **64** | **256** | **128** |
| **Vanc-T** | 0.39 | 25 | **0.05** | **3.13** | **3.13** |  | 1 | 4 | **32** | **64** | **128** |
| **Vanc-H5A** | 0.39 | 25 | 0.39 | 50 | 25 |  | 1 | 4 | 4 | 4 | 16 |
| **Vanc-Tridecane** | 0.78 | 50 | 1.56 | 100 | 50 |  | 0.5 | 2 | 1 | 2 | 8 |

**Table S2. Cytotoxicity of vancomycin analogues and control antibiotics.** IC_50_ corresponds to the compound concentration (in µM) required to inhibit the proliferation of lymphocytic B-cells (L1210), CD4+ T-cells (CEM) or cervical cells (HeLa) by 50%. Values are the mean ± standard deviation of three independent experiments. The highest concentration tested is 250 µM.

|  |  | **Toxicity (IC_50_ in µM)** | | | |
| --- | --- | --- | --- | --- | --- |
|  |  | **Mammalian cell lines** | | |  |
| **Series** | **Vancomycin analogues** | **L1210** | **CEM** | **HeLa** | **Average** |
| 1 | **Vanc-B** | > 250 | > 250 | > 250 | >250 |
| 1 | **Vanc-N** | > 250 | > 250 | > 250 | >250 |
| 1 | **Vanc-Q** | > 250 | > 250 | > 250 | >250 |
| 1 | **Vanc-R** | 5.7 ± 0.7 | 1.4 ± 0.6 | 15 ± 1 | 7 |
| 1 | **Vanc-S** | 10 ± 4 | 5.6 ± 1.1 | 70 ± 8 | 29 |
| 1 | **Vanc-T** | 77 ± 17 | 60 ± 10 | 75 ± 20 | 71 |
| 2 | **Vanc- 1** | > 250 | > 250 | 72 ± 10 | 191 |
| 2 | **Vanc- 2** | > 250 | > 250 | 158 ± 84 | 219 |
| 2 | **Vanc- 3** | > 250 | > 250 | > 250 | >250 |
| 2 | **Vanc- 4** | > 250 | > 250 | > 250 | >250 |
| 2 | **Vanc- 5** | > 250 | > 250 | > 250 | >250 |
| 2 | **Vanc- 6** | > 250 | 159 ± 13 | 91 ± 33 | 167 |
| 2 | **Vanc- 7** | > 250 | > 250 | > 250 | >250 |
| 2 | **Vanc- 8** | > 250 | ≥ 250 | 107 ± 25 | 202 |
| 2 | **Vanc- 9** | > 250 | 240 ± 14 | 83 ± 5 | 191 |
| 2 | **Vanc- 10** | > 250 | > 250 | 214 ± 51 | 238 |
| 2 | **Vanc- 11** | > 250 | 196 ± 15 | 87 ± 9 | 178 |
| 2 | **Vanc- 12** | > 250 | > 250 | 194 ± 78 | 231 |
| 2 | **Vanc-13** | > 250 | > 250 | 149 ± 6 | 216 |
| 2 | **Vanc-14** | 153 ± 55 | 114 ± 18 | 148 ± 6 | 138 |
| 2 | **Vanc-15** | > 250 | > 250 | 87 ± 12 | 196 |
| 2 | **Vanc-16** | > 250 | > 250 | 184 ± 60 | 228 |
| 2 | **Vanc-17** | > 250 | > 250 | 119 ± 48 | 206 |
| 2 | **Vanc-18** | > 250 | > 250 | > 250 | >250 |
| 2 | **Vanc-19** | > 250 | > 250 | 63 ± 5 | 188 |
| 2 | **Vanc-20** | 138 ± 5 | 146 ± 24 | 80 ± 3 | 121 |
| 2 | **Vanc-21** | > 250 | > 250 | > 250 | >250 |
| 2 | **Vanc-22** | > 250 | > 250 | 76 ± 11 | 192 |
| 2 | **Vanc-23** | > 250 | > 250 | > 250 | >250 |
| 2 | **Vanc-24** | 164 ± 20 | 248 ± 3 | 83 ± 9 | 165 |
| 2 | **Vanc-25** | > 250 | > 250 | 98 ± 32 | 199 |
| 2 | **Vanc-26** | >250 | > 250 | 42 ± 23 | 181 |
| 2 | **Vanc-27** | > 250 | 229 ± 30 | 76 ± 8 | 185 |
| 2 | **Vanc-28** | > 250 | 229 ± 12 | 68 ± 16 | 182 |
| 2 | **Vanc-29** | > 250 | > 250 | 54 ± 44 | 185 |
| 2 | **Vanc-30** | > 250 | > 250 | 45 ± 25 | 182 |
| 2 | **Vanc-31** | 8.8 ± 0.6 | 17 ± 5 | 86 ± 14 | 37 |
| 2 | **Vanc-32** | 164 ± 46 | 200 ± 12 | 106 ± 2 | 157 |
| 2 | **Vanc-33** | 57 ± 20 | 78 ± 16 | 84 ± 8 | 73 |
| 2 | **Vanc-34** | 42 ± 7 | 34 ± 2 | 110 ± 8 | 62 |
| 2 | **Vanc-35** | 4.1 ± 0.5 | 0.98 ± 0.06 | 17 ± 2 | 7 |
| 2 | **Vanc-36** | > 250 | > 250 | 89 ± 18 | 196 |
| 2 | **Vanc-37** | 14 ± 7 | 24 ± 4 | 118 ± 10 | 52 |
| 2 | **Vanc-38** | 8.0 ± 3.1 | 4.4 ± 0.5 | 24 ± 1 | 12 |
| 3 | **Vanc-39** | > 250 | > 250 | > 250 | >250 |
| 3 | **Vanc-40** | > 250 | > 250 | > 250 | >250 |
| 3 | **Vanc-41** | > 250 | > 250 | > 250 | >250 |
| 3 | **Vanc-42** | 177 ± 103 | 92 ± 3 | > 250 | 173 |
| 3 | **Vanc-43** | > 250 | > 250 | > 250 | >250 |
| 3 | **Vanc-44** | ≥ 250 | ≥ 250 | 108 ± 38 | 203 |
| 3 | **Vanc-45** | > 250 | > 250 | > 250 | >250 |
| 3 | **Vanc-46** | 197 ± 18 | 235 ± 21 | 108 ± 9 | 180 |
| 3 | **Vanc-47** | ≥ 250 | 240 ± 4 | 106 ± 14 | 199 |
| 3 | **Vanc-48** | 142 ± 21 | 83 ± 11 | > 250 | 158 |
| 4 | **Vanc-49** | 63 ± 8 | 36 ± 8 | 99 ± 1 | 66 |
| 4 | **Vanc-50** | > 250 | 86 ± 2 | 96 ± 4 | 144 |
| 4 | **Vanc-51** | 80 ± 13 | 82 ± 21 | 106 ± 21 | 89 |
| 4 | **Vanc-52** | ≥ 250 | > 250 | 104 ± 13 | 201 |
| 4 | **Vanc-53** | 176 ± 18 | 152 ± 43 | 176 ± 103 | 168 |
| 4 | **Vanc-54** | > 250 | ≥ 250 | 54 ± 29 | 185 |
| 4 | **Vanc-55** | > 250 | ≥ 250 | 103 ± 10 | 201 |
| 4 | **Vanc-56** | > 250 | ≥ 250 | 104 ± 17 | 201 |
| 4 | **Vanc-57** | 6.3 ± 0.2 | 3.7 ± 0.2 | 41 ± 12 | 17 |
| 4 | **Vanc-58** | > 250 | 197 ± 75 | 125 ± 57 | 191 |
| 4 | **Vanc-59** | 177 ± 90 | 23 ± 6 | 88 ± 37 | 96 |
| 4 | **Vanc-60** | > 250 | 219 ± 44 | 75 ± 29 | 181 |
| 4 | **Vanc-61** | ≥ 250 | ≥ 250 | 87 ± 37 | 196 |
| 4 | **Vanc-62** | > 250 | ≥ 250 | 86 ± 42 | 195 |
| 4 | **Vanc-63** | > 250 | ≥ 250 | 69 ± 74 | 190 |
| 4 | **Vanc-64** | > 250 | 210 ± 56 | 102 ± 39 | 187 |
| 4 | **Vanc-65** | > 250 | ≥ 250 | 54 ± 45 | 185 |
| 4 | **Vanc-66** | > 250 | 243 ± 10 | 78 ± 49 | 190 |
| 5 | **Vanc-67** | ≥ 250 | 165 ± 32 | 52 ± 19 | 156 |
| 5 | **Vanc-68** | 42 ± 14 | 19 ± 1 | 55 ± 20 | 39 |
| 5 | **Vanc-69** | 185 ± 93 | 29 ± 0 | 55 ± 37 | 90 |
| 5 | **Vanc-70** | ≥ 250 | 143 ± 113 | 73 ± 10 | 155 |
| 5 | **Vanc-71** | > 250 | 137 ± 41 | 91 ± 36 | 159 |
| 5 | **Vanc-72** | 173 ± 106 | 17 ± 1 | 25 ± 10 | 72 |
| 5 | **Vanc-73** | 108 ± 42 | 130 ± 6 | 81 ± 6 | 106 |
| 5 | **Vanc-74** | ≥ 250 | 78 ± 20 | 102 ± 46 | 143 |
| 5 | **Vanc-75** | 17 ± 7 | 15 ± 3 | 83 ± 4 | 38 |
| 5 | **Vanc-76** | 11 ± 7 | 3.2 ± 0.6 | 17 ± 2 | 10 |
| 5 | **Vanc-77** | 192 ± 101 | 26 ± 1 | 58 ± 7 | 92 |
| 6 | **Vanc-78** | > 250 | > 250 | 57 ± 32 | 186 |
| 6 | **Vanc-79** | > 250 | > 250 | > 250 | >250 |
| 6 | **Vanc-80** | 230 ± 3 | > 250 | 238 ± 0 | 239 |
| 6 | **Vanc-81** | 164 ± 6 | > 250 | 122 ± 13 | 179 |
| 6 | **Vanc-82** | > 250 | > 250 | > 250 | >250 |
| 6 | **Vanc-83** | 179 ± 41 | 37 ± 6 | 105 ± 27 | 107 |
| 7 | **Vanc-84** | 1.5 ± 0.1 | 1.6 ± 0.9 | 13 ± 0 | 5 |
| 7 | **Vanc-85** | 31 ± 9 | 5.6 ± 0.6 | 94 ± 12 | 44 |
| 7 | **Vanc-86** | 3.0 ± 2.3 | 1.0 ± 0.2 | 9.8 ± 5.8 | 5 |
| 7 | **Vanc-87** | 56 ± 33 | 81 ± 22 | 80 ± 0 | 72 |
| 7 | **Vanc-88** | 127 ± 32 | 32 ± 1 | 98 ± 23 | 86 |
| 7 | **Vanc-89** | 71 ± 56 | 38 ± 10 | 154 ± 30 | 88 |
| 7 | **Vanc-90** | 175 ± 106 | 68 ± 8 | 127 ± 27 | 123 |
| 7 | **Vanc-91** | 154 ± 93 | 107 ± 25 | 147 ± 11 | 136 |
| 7 | **Vanc-92** | 95 ± 75 | 31 ± 7 | 98 ± 12 | 75 |
| Control | **Tigecycline** | 9.4 ± 3.2 | 7.1 ± 2.1 | 24 ± 17 | 14 |
| Control | **Daptomycin** | >250 | >250 | >250 | >250 |
| Control | **Linezolid** | 157 ± 7 | > 250 | 216 ± 48 | 208 |
| Control | **Vancomycin** | > 250 | > 250 | > 250 | >250 |
|  |  | **L1210** | **CEM** | **HeLa** | **Average** |

**Table S3. Minimal inhibitory concentrations of all vancomycin analogues and control antibiotics against *E. faecalis*.** The MIC values of the best analogues of the first series (Vanc-B to Vanc-T) and all other series (Vanc-1 tot Vanc-92) are shown against one vancomycin-sensitive *E. faecalis* strain (VSE) and four vancomycin-resistant strains (VRE 6, VRE 29, VRE 37 and VRE 53). Fold changes in MIC values are expressed as the ratio of the MIC of vancomycin and the MIC of an analogue against the respective strains.

|  |  | **MIC (µM)** | | | | |  | **MIC-fold improvement vs. vancomycin** | | | | |
| --- | --- | --- | --- | --- | --- | --- | --- | --- | --- | --- | --- | --- |
|  |  | ***Enterococcus faecalis*** | | | | |  | ***Enterococcus faecalis*** | | | | |
| **Series** | **Vancomycin analogues** | **VSE 1** | **VRE 6** | **VRE 29** | **VRE 37** | **VRE 53** |  | **VSE 1** | **VRE 6** | **VRE 29** | **VRE 37** | **VRE 53** |
| 1 | **Vanc-B** | 0.78 | 50 | 100 | 100 | 12.5 |  | 2 | 4 | 4 | 8 | 32 |
| 1 | **Vanc-N** | 0.10 | 3.13 | 6.25 | 25 | 6.25 |  | 16 | 64 | 64 | 32 | 64 |
| 1 | **Vanc-Q** | 0.10 | 6.25 | 25 | 50 | 6.25 |  | 16 | 32 | 16 | 16 | 64 |
| 1 | **Vanc-R** | 0.19 | 3.13 | 1.56 | 12.5 | 6.25 |  | 8 | 16 | 256 | 64 | 64 |
| 1 | **Vanc-S** | 0.03 | 0.78 | 6.25 | 25 | 3.13 |  | 64 | 256 | 64 | 32 | 128 |
| 1 | **Vanc-T** | 0.05 | 3.13 | 12.5 | 25 | 3.13 |  | 32 | 64 | 32 | 32 | 128 |
| 2 | **Vanc- 1** | 0.78 | 25 | >50 | 50 | 25 |  | 2 | 8 | <8 | 16 | 16 |
| 2 | **Vanc- 2** | 0.39 | 12.5 | 12.5 | 25 | 25 |  | 4 | 16 | 32 | 32 | 16 |
| 2 | **Vanc- 3** | 0.78 | 12.5 | 25 | 25 | 25 |  | 2 | 16 | 16 | 32 | 16 |
| 2 | **Vanc- 4** | 0.39 | 3.13 | 6.25 | 6.25 | 6.25 |  | 4 | 64 | 64 | 128 | 64 |
| 2 | **Vanc- 5** | 0.78 | 6.25 | 12.5 | 12.5 | 12.5 |  | 2 | 32 | 32 | 64 | 32 |
| 2 | **Vanc- 6** | 0.39 | 3.13 | 6.25 | 12.5 | 12.5 |  | 4 | 64 | 32 | 64 | 32 |
| 2 | **Vanc- 7** | 0.78 | 12.5 | 12.5 | 12.5 | 12.5 |  | 2 | 16 | 32 | 64 | 32 |
| 2 | **Vanc- 8** | 0.78 | 12.5 | 25 | 25 | 12.5 |  | 2 | 16 | 16 | 32 | 32 |
| 2 | **Vanc- 9** | 0.78 | 6.25 | 25 | 25 | 25 |  | 2 | 32 | 16 | 32 | 16 |
| 2 | **Vanc- 10** | 0.78 | 6.25 | 12.5 | 12.5 | 12.5 |  | 2 | 32 | 32 | 64 | 32 |
| 2 | **Vanc- 11** | 0.39 | 3.13 | 6.25 | 12.5 | 12.5 |  | 4 | 64 | 32 | 64 | 32 |
| 2 | **Vanc- 12** | 0.78 | 6.25 | 12.5 | 25 | 12.5 |  | 2 | 32 | 16 | 32 | 32 |
| 2 | **Vanc-13** | 0.39 | 12.5 | 12.5 | 3.13 | 12.5 |  | 4 | 16 | 128 | 256 | 32 |
| 2 | **Vanc-14** | 0.20 | 1.56 | 3.13 | 6.25 | 3.13 |  | 8 | 128 | 64 | 128 | 128 |
| 2 | **Vanc-15** | 1.56 | 6.25 | 12.5 | 25 | 25 |  | 1 | 32 | 32 | 16 | 16 |
| 2 | **Vanc-16** | 0.78 | 12.5 | >50 | 50 | 25 |  | 2 | 16 | <8 | 8 | 16 |
| 2 | **Vanc-17** | 0.78 | 6.25 | 25 | 50 | 25 |  | 2 | 32 | 16 | 8 | 16 |
| 2 | **Vanc-18** | 1.56 | 12.5 | 25 | 50 | 25 |  | 1 | 16 | 16 | 8 | 16 |
| 2 | **Vanc-19** | 1.56 | 3.13 | 12.5 | 25 | 25 |  | 1 | 64 | 32 | 16 | 16 |
| 2 | **Vanc-20** | 0.39 | 1.56 | 6.25 | 12.5 | 12.5 |  | 4 | 128 | 64 | 32 | 32 |
| 2 | **Vanc-21** | 0.39 | 1.56 | 12.5 | 12.5 | 12.5 |  | 4 | 128 | 32 | 32 | 32 |
| 2 | **Vanc-22** | 0.39 | 1.56 | 12.5 | 12.5 | 25 |  | 4 | 128 | 32 | 32 | 16 |
| 2 | **Vanc-23** | 0.39 | 3.13 | 12.5 | 25 | 25 |  | 4 | 64 | 32 | 16 | 16 |
| 2 | **Vanc-24** | 0.20 | 0.78 | 12.5 | 6.25 | 25 |  | 8 | 256 | 32 | 64 | 16 |
| 2 | **Vanc-25** | 0.39 | 0.78 | 6.25 | 12.5 | 25 |  | 4 | 256 | 64 | 32 | 16 |
| 2 | **Vanc-26** | 0.20 | 3.13 | 6.25 | 12.5 | 25 |  | 8 | 64 | 64 | 32 | 16 |
| 2 | **Vanc-27** | 0.39 | 3.13 | 12.5 | 12.5 | 25 |  | 4 | 64 | 32 | 32 | 16 |
| 2 | **Vanc-28** | 0.20 | 3.13 | 6.25 | 12.5 | 25 |  | 8 | 64 | 64 | 32 | 16 |
| 2 | **Vanc-29** | 0.20 | 1.56 | 6.25 | 12.5 | 25 |  | 8 | 128 | 64 | 32 | 16 |
| 2 | **Vanc-30** | 0.39 | 3.13 | 12.5 | 25 | 25 |  | 4 | 64 | 32 | 16 | 16 |
| 2 | **Vanc-31** | 0.39 | 0.39 | 1.56 | 1.56 | 3.13 |  | 4 | 513 | 256 | 256 | 128 |
| 2 | **Vanc-32** | 0.39 | 3.13 | 12.5 | 25 | 50 |  | 4 | 64 | 32 | 16 | 8 |
| 2 | **Vanc-33** | 0.20 | 1.56 | 6.25 | 12.5 | 25 |  | 8 | 128 | 64 | 32 | 16 |
| 2 | **Vanc-34** | 0.20 | 0.39 | 3.13 | 6.25 | 12.5 |  | 8 | 513 | 128 | 64 | 32 |
| 2 | **Vanc-35** | 0.20 | 0.39 | 1.56 | 1.56 | 6.25 |  | 8 | 513 | 256 | 256 | 64 |
| 2 | **Vanc-36** | 0.39 | 1.56 | 12.5 | 12.5 | 50 |  | 4 | 128 | 32 | 32 | 8 |
| 2 | **Vanc-37** | 0.20 | 1.56 | 6.25 | 1.56 | 3.13 |  | 8 | 128 | 64 | 256 | 128 |
| 2 | **Vanc-38** | 0.20 | 0.39 | 1.56 | 3.13 | 3.13 |  | 8 | 513 | 256 | 128 | 128 |
| 3 | **Vanc-39** | 0.20 | 3.13 | 6.25 | 6.25 | 50 |  | 8 | 64 | 64 | 64 | 8 |
| 3 | **Vanc-40** | 0.78 | 6.25 | 12.5 | 12.5 | 25 |  | 2 | 32 | 32 | 32 | 16 |
| 3 | **Vanc-41** | 0.39 | 6.25 | 12.5 | 12.5 | 25 |  | 4 | 32 | 32 | 32 | 16 |
| 3 | **Vanc-42** | 0.20 | 0.78 | 1.56 | 3.13 | 12.5 |  | 8 | 256 | 256 | 128 | 32 |
| 3 | **Vanc-43** | 0.39 | 1.56 | 6.25 | 6.25 | 25 |  | 4 | 128 | 64 | 64 | 16 |
| 3 | **Vanc-44** | 0.78 | 12.5 | 25 | 25 | 50 |  | 2 | 16 | 16 | 16 | 8 |
| 3 | **Vanc-45** | 0.39 | 3.13 | 12.5 | 12.5 | 25 |  | 4 | 64 | 32 | 32 | 16 |
| 3 | **Vanc-46** | 0.78 | 6.25 | 25 | 25 | 25 |  | 2 | 32 | 16 | 16 | 16 |
| 3 | **Vanc-47** | 0.78 | 6.25 | 25 | 25 | 50 |  | 2 | 32 | 16 | 16 | 8 |
| 3 | **Vanc-48** | 0.78 | 1.56 | 3.13 | 3.13 | 6.25 |  | 2 | 128 | 128 | 128 | 64 |
| 4 | **Vanc-49** | 0.39 | 6.25 | 6.25 | 6.25 | 6.25 |  | 4 | 32 | 64 | 64 | 64 |
| 4 | **Vanc-50** | 0.39 | 6.25 | 12.5 | 12.5 | 12.5 |  | 4 | 32 | 32 | 32 | 32 |
| 4 | **Vanc-51** | 0.39 | 6.25 | 12.5 | 12.5 | 12.5 |  | 4 | 32 | 32 | 32 | 32 |
| 4 | **Vanc-52** | 0.78 | 25 | 50 | 50 | 25 |  | 2 | 8 | 8 | 8 | 16 |
| 4 | **Vanc-53** | 0.78 | 12.5 | 25 | 25 | 12.5 |  | 2 | 16 | 16 | 16 | 32 |
| 4 | **Vanc-54** | 0.78 | 12.5 | 25 | 25 | 25 |  | 2 | 16 | 16 | 16 | 16 |
| 4 | **Vanc-55** | 0.39 | 6.25 | 12.5 | 12.5 | 12.5 |  | 4 | 32 | 32 | 32 | 32 |
| 4 | **Vanc-56** | 0.39 | 3.13 | 6.25 | 6.25 | 6.25 |  | 4 | 64 | 64 | 64 | 64 |
| 4 | **Vanc-57** | ≤0.05 | 1.56 | 3.13 | 1.56 | 12.5 |  | ≥32 | 128 | 128 | 256 | 32 |
| 4 | **Vanc-58** | 0.78 | 6.25 | 12.5 | 12.5 | 25 |  | 2 | 32 | 32 | 32 | 16 |
| 4 | **Vanc-59** | 0.20 | 1.56 | 3.13 | 1.56 | 12.5 |  | 8 | 128 | 128 | 256 | 32 |
| 4 | **Vanc-60** | 0.39 | 12.5 | 12.5 | 25 | 12.5 |  | 4 | 16 | 32 | 16 | 32 |
| 4 | **Vanc-61** | 0.39 | 3.13 | 6.25 | 12.5 | 6.25 |  | 4 | 64 | 64 | 32 | 64 |
| 4 | **Vanc-62** | 0.39 | 3.13 | 6.25 | 12.5 | 25 |  | 4 | 64 | 64 | 32 | 16 |
| 4 | **Vanc-63** | 0.39 | 3.13 | 6.25 | 12.5 | 25 |  | 4 | 64 | 64 | 32 | 16 |
| 4 | **Vanc-64** | 0.39 | 3.13 | 6.25 | 6.25 | 12.5 |  | 4 | 64 | 64 | 64 | 32 |
| 4 | **Vanc-65** | 0.39 | 3.13 | 6.25 | 6.25 | 25 |  | 4 | 64 | 64 | 64 | 16 |
| 4 | **Vanc-66** | 0.39 | 1.56 | 3.13 | 3.13 | 25 |  | 4 | 128 | 128 | 128 | 16 |
| 5 | **Vanc-67** | 0.78 | 12.5 | 25 | 25 | 25 |  | 2 | 16 | 16 | 16 | 16 |
| 5 | **Vanc-68** | 0.78 | 3.13 | 3.13 | 3.13 | 3.13 |  | 2 | 64 | 128 | 128 | 128 |
| 5 | **Vanc-69** | 0.78 | 3.13 | 6.25 | 6.25 | 6.25 |  | 2 | 64 | 64 | 64 | 64 |
| 5 | **Vanc-70** | 0.78 | 6.25 | 6.25 | 6.25 | 6.25 |  | 2 | 32 | 64 | 64 | 64 |
| 5 | **Vanc-71** | 0.78 | 3.13 | 6.25 | 6.25 | 6.25 |  | 2 | 64 | 64 | 64 | 64 |
| 5 | **Vanc-72** | 0.78 | 3.13 | 6.25 | 6.25 | 6.25 |  | 2 | 64 | 64 | 64 | 64 |
| 5 | **Vanc-73** | 0.78 | 6.25 | 12.5 | 25 | 25 |  | 2 | 32 | 32 | 16 | 16 |
| 5 | **Vanc-74** | 0.39 | 1.56 | 12.5 | 6.25 | 6.25 |  | 4 | 128 | 32 | 64 | 64 |
| 5 | **Vanc-75** | 0.78 | 3.13 | 3.13 | 3.13 | 3.13 |  | 2 | 64 | 128 | 128 | 128 |
| 5 | **Vanc-76** | 0.78 | 1.56 | 3.13 | 3.13 | 3.13 |  | 2 | 128 | 128 | 128 | 128 |
| 5 | **Vanc-77** | 0.39 | 1.56 | 6.25 | 6.25 | 6.25 |  | 4 | 128 | 64 | 64 | 64 |
| 6 | **Vanc-78** | 0.78 | 3.13 | 50 | 50 | 50 |  | 2 | 64 | 8 | 8 | 8 |
| 6 | **Vanc-79** | 0.78 | 1.56 | 50 | 50 | 50 |  | 2 | 128 | 8 | 8 | 8 |
| 6 | **Vanc-80** | 1.56 | 6.25 | 50 | 50 | 50 |  | 1 | 32 | 8 | 8 | 8 |
| 6 | **Vanc-81** | 0.78 | ≤0.39 | 25 | 25 | 25 |  | 2 | >512 | 16 | 16 | 16 |
| 6 | **Vanc-82** | 1.56 | 6.25 | >50 | >50 | 50 |  | 1 | 32 | <8 | <8 | 8 |
| 6 | **Vanc-83** | 0.39 | 0.78 | 1.56 | 1.56 | 1.56 |  | 4 | 256 | 256 | 256 | 256 |
| 7 | **Vanc-84** | ≤0.05 | 0.78 | 0.78 | 3.13 | 3.13 |  | ≥32 | 256 | 512 | 128 | 128 |
| 7 | **Vanc-85** | ≤0.05 | 0.78 | 1.56 | 3.13 | 12.5 |  | ≥32 | 256 | 512 | 128 | 32 |
| 7 | **Vanc-86** | ≤0.05 | 1.56 | 3.13 | 6.25 | 12.5 |  | ≥32 | 128 | 128 | 64 | 32 |
| 7 | **Vanc-87** | 0.10 | 3.13 | 6.25 | 6.25 | 25 |  | 16 | 64 | 64 | 64 | 16 |
| 7 | **Vanc-88** | ≤0.05 | 1.56 | 1.56 | 3.13 | 12.5 |  | ≥32 | 128 | 256 | 128 | 32 |
| 7 | **Vanc-89** | ≤0.05 | 3.13 | 6.25 | 6.25 | 12.5 |  | ≥32 | 64 | 64 | 64 | 32 |
| 7 | **Vanc-90** | ≤0.05 | 1.56 | 3.13 | 3.13 | 12.5 |  | ≥32 | 128 | 128 | 128 | 32 |
| 7 | **Vanc-91** | ≤0.05 | 1.56 | 6.25 | 6.25 | 25 |  | ≥32 | 128 | 64 | 64 | 16 |
| 7 | **Vanc-92** | 0.10 | 6.25 | 6.25 | 6.25 | 25 |  | 16 | 32 | 64 | 64 | 16 |
| Control | **Tigecycline** | 3.13 | 3.13 | 3.13 | 3.13 | 3.13 |  | 0.5 | 64 | 128 | 128 | 128 |
| Control | **Daptomycin** | >6.25 | 6.25 | 50 | 50 | 50 |  | <0.2 | 32 | 8 | 8 | 8 |
| Control | **Linezolid** | >3.13 | 12.5 | 6.25 | 6.25 | 6.25 |  | <0.5 | 16 | 64 | 64 | 64 |
| Control | **Vancomycin** | 1.56 | 200 | 400 | 400 | 400 |  | 1 | 1 | 1 | 1 | 1 |
|  |  | **VSE 1** | **VRE 6** | **VRE 29** | **VRE 37** | **VRE 53** |  | **VSE 1** | **VRE 6** | **VRE 29** | **VRE 37** | **VRE 53** |

**Table S4. Minimal inhibitory concentrations of all vancomycin analogues and control antibiotics against *S. aureus*.** The MIC values of analogues of the first series (Vanc-B to Vanc-T) and all other series (Vanc-1 tot Vanc-92) are shown against one vancomycin-sensitive *S. aureus* strain (VSSA), a vancomycin-intermediate resistant strain (VISA/HIP5827), a vancomycin-resistant strain (VRSA), and four methicillin-resistant strains (MRSA KS2, KS6, KS89, KS1). Fold changes in MIC values are expressed as the ratio of the MIC of vancomycin and the MIC of an analogue against the respective strains.

|  |  | **MIC (µM)** | | | | | | | |  | **MIC-fold improvement vs. vancomycin** | | | | | | | |
| --- | --- | --- | --- | --- | --- | --- | --- | --- | --- | --- | --- | --- | --- | --- | --- | --- | --- | --- |
|  |  | ***Staphyloccus aureus*** | | | | | | | |  | ***Staphylococcus aureus*** | | | | | | | |
| **Series** | **Vancomycin analogues** | **VSSA** | **VISA/ HIP5827** | **VRSA** |  | **MRSA KS2** | **MRSA KS6** | **MRSA KS8** | **MRSA KS1** |  | **VSSA** | **VISA/ HIP5827** | **VRSA** |  | **MRSA KS2** | **MRSA KS6** | **MRSA KS8** | **MRSA KS1** |
| 1 | **Vanc-B** | 0.78 | 3.13 | 12.5 |  | 0.78 | 1.56 | 1.56 | 0.78 |  | 1 | 2 | 8 |  | 1 | 0.5 | 1 | 1 |
| 1 | **Vanc-N** | 0.10 | 0.78 | 1.56 |  | 0.39 | 0.39 | 0.39 | 0.20 |  | 8 | 8 | 64 |  | 2 | 2 | 2 | 4 |
| 1 | **Vanc-Q** | 0.20 | 1.56 | 12.5 |  | 1.56 | 0.78 | 0.78 | 0.39 |  | 4 | 4 | 8 |  | 0.5 | 1 | 1 | 2 |
| 1 | **Vanc-R** | 0.10 | 0.78 | 1.56 |  | 0.39 | 0.39 | 0.39 | 0.78 |  | 8 | 8 | 64 |  | 2 | 2 | 2 | 1 |
| 1 | **Vanc-S** | 0.10 | 0.39 | 0.78 |  | 0.20 | 0.39 | 0.20 | <0.10 |  | 8 | 16 | 128 |  | 4 | 2 | 4 | >8 |
| 1 | **Vanc-T** | 0.10 | 1.56 | 1.56 |  | 0.20 | 0.39 | 0.20 | 0.20 |  | 8 | 4 | 64 |  | 4 | 2 | 4 | 4 |
| 2 | **Vanc- 1** | 0.78 | 0.78 | 25 |  | 0.78 | 0.78 | 0.78 | 0.78 |  | 1 | 8 | 4 |  | 1 | 1 | 1 | 1 |
| 2 | **Vanc- 2** | 0.78 | 0.78 | 12.5 |  | 0.39 | 0.39 | 0.39 | 0.39 |  | 1 | 8 | 8 |  | 2 | 2 | 2 | 2 |
| 2 | **Vanc- 3** | 0.78 | 0.78 | 25 |  | 0.78 | 0.78 | 0.78 | 0.78 |  | 1 | 8 | 4 |  | 1 | 1 | 1 | 1 |
| 2 | **Vanc- 4** | 0.39 | 0.39 | 6.25 |  | 0.20 | 0.39 | 0.39 | 0.39 |  | 2 | 16 | 16 |  | 4 | 2 | 2 | 2 |
| 2 | **Vanc- 5** | 0.39 | 0.39 | 12.5 |  | 0.39 | 0.78 | 0.78 | 0.78 |  | 2 | 16 | 8 |  | 2 | 1 | 1 | 1 |
| 2 | **Vanc- 6** | 0.39 | 0.39 | 3.13 |  | 0.39 | 0.39 | 0.39 | 0.39 |  | 2 | 16 | 32 |  | 2 | 2 | 2 | 2 |
| 2 | **Vanc- 7** | 0.78 | 0.78 | 12.5 |  | 0.39 | 0.78 | 0.78 | 0.78 |  | 1 | 8 | 8 |  | 2 | 1 | 1 | 1 |
| 2 | **Vanc- 8** | 1.56 | 1.56 | 25 |  | 0.78 | 1.56 | 0.78 | 0.78 |  | 0.5 | 4 | 4 |  | 1 | 0.5 | 1 | 1 |
| 2 | **Vanc- 9** | 0.78 | 1.56 | 25 |  | 0.78 | 0.78 | 0.78 | 0.78 |  | 1 | 4 | 4 |  | 1 | 1 | 1 | 1 |
| 2 | **Vanc- 10** | 0.78 | 0.78 | 12.5 |  | 0.39 | 0.78 | 0.39 | 0.39 |  | 1 | 8 | 8 |  | 2 | 1 | 2 | 2 |
| 2 | **Vanc- 11** | 0.78 | 0.78 | 12.5 |  | 0.78 | 0.39 | 0.39 | 0.78 |  | 1 | 8 | 8 |  | 1 | 2 | 2 | 1 |
| 2 | **Vanc- 12** | 0.39 | 0.39 | 12.5 |  | 0.39 | 0.78 | 0.39 | 0.39 |  | 2 | 16 | 8 |  | 2 | 1 | 2 | 2 |
| 2 | **Vanc-13** | 0.39 | 0.39 | 3.13 |  | 0.20 | 0.39 | 0.39 | 0.39 |  | 2 | 16 | 32 |  | 4 | 2 | 2 | 2 |
| 2 | **Vanc-14** | 0.20 | 0.10 | 3.13 |  | 0.20 | 0.39 | 0.20 | 0.39 |  | 4 | 64 | 32 |  | 4 | 2 | 4 | 2 |
| 2 | **Vanc-15** | 0.78 | 1.56 | 12.5 |  | 0.39 | 0.78 | 0.78 | 0.78 |  | 1 | 4 | 8 |  | 2 | 1 | 1 | 1 |
| 2 | **Vanc-16** | 0.78 | 0.78 | 25 |  | 0.78 | 0.78 | 0.78 | 0.78 |  | 1 | 8 | 4 |  | 1 | 1 | 1 | 1 |
| 2 | **Vanc-17** | 0.78 | 0.78 | 12.5 |  | 0.39 | 0.78 | 0.78 | 0.78 |  | 1 | 8 | 8 |  | 2 | 1 | 1 | 1 |
| 2 | **Vanc-18** | 0.78 | 0.78 | 25 |  | 0.39 | 0.78 | 0.78 | 0.78 |  | 1 | 8 | 4 |  | 2 | 1 | 1 | 1 |
| 2 | **Vanc-19** | 0.78 | 3.13 | 12.5 |  | 0.78 | 1.56 | 0.78 | 0.78 |  | 1 | 2 | 8 |  | 1 | 0.5 | 1 | 1 |
| 2 | **Vanc-20** | 0.39 | 0.39 | 3.13 |  | 0.20 | 0.39 | 0.39 | 0.39 |  | 2 | 16 | 32 |  | 4 | 2 | 2 | 2 |
| 2 | **Vanc-21** | 0.78 | 0.78 | 1.56 |  | 0.39 | 0.78 | 0.78 | 0.78 |  | 1 | 8 | 64 |  | 2 | 1 | 1 | 1 |
| 2 | **Vanc-22** | 0.39 | 1.56 | 3.13 |  | 0.39 | 0.39 | 0.39 | 0.39 |  | 2 | 4 | 32 |  | 2 | 2 | 2 | 2 |
| 2 | **Vanc-23** | 0.39 | 0.78 | 6.25 |  | 0.39 | 0.78 | 0.78 | 0.78 |  | 2 | 8 | 16 |  | 2 | 1 | 1 | 1 |
| 2 | **Vanc-24** | 0.20 | 0.19 | 1.56 |  | 0.20 | 0.39 | 0.39 | 0.39 |  | 4 | 32 | 64 |  | 4 | 2 | 2 | 2 |
| 2 | **Vanc-25** | 0.39 | 0.39 | 3.13 |  | 0.39 | 0.39 | 0.39 | 0.39 |  | 2 | 16 | 32 |  | 2 | 2 | 2 | 2 |
| 2 | **Vanc-26** | 0.39 | 0.39 | 3.25 |  | 0.39 | 0.39 | 0.39 | 0.39 |  | 2 | 16 | 32 |  | 2 | 2 | 2 | 2 |
| 2 | **Vanc-27** | 0.39 | 0.39 | 6.25 |  | 0.39 | 0.39 | 0.78 | 0.39 |  | 2 | 16 | 16 |  | 2 | 2 | 1 | 2 |
| 2 | **Vanc-28** | 0.39 | 0.39 | 3.13 |  | 0.20 | 0.20 | 0.39 | 0.39 |  | 2 | 16 | 32 |  | 4 | 4 | 2 | 2 |
| 2 | **Vanc-29** | 0.39 | 0.39 | 3.13 |  | 0.39 | 0.39 | 0.39 | 0.39 |  | 2 | 16 | 32 |  | 2 | 2 | 2 | 2 |
| 2 | **Vanc-30** | 0.39 | 0.39 | 3.13 |  | 0.39 | 0.39 | 0.39 | 0.39 |  | 2 | 16 | 32 |  | 2 | 2 | 2 | 2 |
| 2 | **Vanc-31** | 0.20 | 0.05 | 0.78 |  | 0.20 | 0.20 | 0.20 | 0.78 |  | 4 | 128 | 128 |  | 4 | 4 | 4 | 1 |
| 2 | **Vanc-32** | 0.78 | 0.78 | 6.25 |  | 0.39 | 0.39 | 0.39 | 0.39 |  | 1 | 8 | 16 |  | 2 | 2 | 2 | 2 |
| 2 | **Vanc-33** | 0.20 | 0.20 | 0.78 |  | 0.20 | 0.39 | 0.39 | 0.39 |  | 4 | 32 | 128 |  | 4 | 2 | 2 | 2 |
| 2 | **Vanc-34** | 0.10 | 0.10 | 0.78 |  | 0.10 | 0.20 | 0.20 | 0.20 |  | 8 | 64 | 128 |  | 8 | 4 | 4 | 4 |
| 2 | **Vanc-35** | 0.05 | 0.05 | 1.56 |  | 0.39 | 0.20 | 0.20 | 0.78 |  | 16 | 128 | 64 |  | 2 | 4 | 4 | 1 |
| 2 | **Vanc-36** | 0.39 | 0.39 | nd |  | 0.39 | 0.39 | 0.78 | 0.78 |  | 2 | 16 | nd |  | 2 | 2 | 1 | 1 |
| 2 | **Vanc-37** | 0.10 | 0.10 | 1.56 |  | 0.20 | 0.10 | 0.20 | 0.20 |  | 8 | 64 | 64 |  | 4 | 8 | 4 | 4 |
| 2 | **Vanc-38** | 0.05 | 0.05 | 0.78 |  | 0.20 | 0.20 | 0.20 | 0.20 |  | 16 | 128 | 128 |  | 4 | 4 | 4 | 4 |
| 3 | **Vanc-39** | 0.05 | 0.39 | 0.78 |  | 0.20 | 0.20 | 0.20 | 0.20 |  | 16 | 16 | 128 |  | 4 | 4 | 4 | 4 |
| 3 | **Vanc-40** | 0.78 | 1.56 | 3.13 |  | 0.39 | 0.39 | 0.39 | 0.39 |  | 1 | 4 | 32 |  | 2 | 2 | 2 | 2 |
| 3 | **Vanc-41** | 0.78 | 1.56 | 6.25 |  | 0.39 | 0.39 | 0.39 | 0.39 |  | 1 | 4 | 16 |  | 2 | 2 | 2 | 2 |
| 3 | **Vanc-42** | 0.78 | 0.78 | 1.56 |  | 0.20 | 0.20 | 0.20 | 0.39 |  | 1 | 8 | 64 |  | 4 | 4 | 4 | 2 |
| 3 | **Vanc-43** | 0.39 | 1.56 | 1.56 |  | 0.39 | 3.13 | 0.39 | 0.39 |  | 2 | 4 | 64 |  | 2 | 0.25 | 2 | 2 |
| 3 | **Vanc-44** | 0.78 | 1.56 | 12.5 |  | 0.78 | 3.13 | 0.78 | 0.78 |  | 1 | 4 | 8 |  | 1 | 0.25 | 1 | 1 |
| 3 | **Vanc-45** | 0.39 | 1.56 | 1.56 |  | 0.39 | 3.13 | 0.39 | 0.39 |  | 2 | 4 | 64 |  | 2 | 0.25 | 2 | 2 |
| 3 | **Vanc-46** | 0.78 | 1.56 | 6.25 |  | 0.39 | 3.13 | 0.39 | 0.78 |  | 1 | 4 | 16 |  | 2 | 0.25 | 2 | 1 |
| 3 | **Vanc-47** | 0.78 | 1.56 | 3.13 |  | 0.39 | 3.13 | 0.39 | 0.39 |  | 1 | 4 | 32 |  | 2 | 0.25 | 2 | 2 |
| 3 | **Vanc-48** | 0.20 | 1.56 | 1.56 |  | 0.20 | 3.13 | 0.39 | <0.10 |  | 4 | 4 | 64 |  | 4 | 0.25 | 2 | >8 |
| 4 | **Vanc-49** | 0.20 | 0.39 | 0.39 |  | 0.20 | 0.20 | 0.20 | <0.10 |  | 4 | 16 | 256 |  | 4 | 4 | 4 | >8 |
| 4 | **Vanc-50** | 0.39 | 0.78 | 0.78 |  | 0.20 | 0.39 | 0.39 | 0.20 |  | 2 | 8 | 128 |  | 4 | 2 | 2 | 4 |
| 4 | **Vanc-51** | 0.39 | 0.78 | 0.39 |  | 0.20 | 0.20 | 0.20 | 0.20 |  | 2 | 8 | 256 |  | 4 | 4 | 4 | 4 |
| 4 | **Vanc-52** | 0.39 | 0.78 | 1.56 |  | 0.39 | 0.78 | 0.78 | 0.39 |  | 2 | 8 | 64 |  | 2 | 1 | 1 | 2 |
| 4 | **Vanc-53** | 0.39 | 0.78 | 0.39 |  | 0.39 | 0.39 | 0.39 | 0.39 |  | 2 | 8 | 256 |  | 2 | 2 | 2 | 2 |
| 4 | **Vanc-54** | 0.39 | 0.78 | 0.39 |  | 0.39 | 0.39 | 0.39 | 0.39 |  | 2 | 8 | 256 |  | 2 | 2 | 2 | 2 |
| 4 | **Vanc-55** | 0.78 | 0.78 | 0.39 |  | 0.20 | 0.39 | 0.39 | 0.20 |  | 1 | 8 | 256 |  | 4 | 2 | 2 | 4 |
| 4 | **Vanc-56** | 0.39 | 0.78 | 0.39 |  | 0.20 | 0.20 | 0.39 | 0.78 |  | 2 | 8 | 256 |  | 4 | 4 | 2 | 1 |
| 4 | **Vanc-57** | 0.20 | 0.39 | 1.56 |  | 0.20 | 1.56 | 0.39 | 0.10 |  | 4 | 16 | 64 |  | 4 | 0.5 | 2 | 8 |
| 4 | **Vanc-58** | 0.78 | 1.56 | 0.78 |  | 0.78 | 0.78 | 0.78 | 1.56 |  | 1 | 4 | 128 |  | 1 | 1 | 1 | 0.5 |
| 4 | **Vanc-59** | 0.39 | 0.39 | 0.20 |  | nd | nd | nd | nd |  | 2 | 16 | 512 |  | nd | nd | nd | nd |
| 4 | **Vanc-60** | 0.39 | 0.78 | 0.39 |  | 0.39 | 0.78 | 0.39 | 0.39 |  | 2 | 8 | 256 |  | 2 | 1 | 2 | 2 |
| 4 | **Vanc-61** | 0.20 | 0.78 | 0.39 |  | 0.20 | 0.39 | 0.39 | 0.39 |  | 4 | 8 | 256 |  | 4 | 2 | 2 | 2 |
| 4 | **Vanc-62** | 0.39 | 1.56 | 0.78 |  | 0.20 | 0.39 | 0.39 | 0.20 |  | 2 | 4 | 128 |  | 4 | 2 | 2 | 4 |
| 4 | **Vanc-63** | 0.39 | 0.78 | 0.78 |  | 0.39 | 0.39 | 0.39 | 0.39 |  | 2 | 8 | 128 |  | 2 | 2 | 2 | 2 |
| 4 | **Vanc-64** | 0.39 | 0.78 | 0.39 |  | 0.20 | 0.20 | 0.39 | 0.20 |  | 2 | 8 | 256 |  | 4 | 4 | 2 | 4 |
| 4 | **Vanc-65** | 0.39 | 0.78 | 0.39 |  | 0.20 | 0.39 | 0.39 | 0.78 |  | 2 | 8 | 256 |  | 4 | 2 | 2 | 1 |
| 4 | **Vanc-66** | 0.78 | 0.78 | 0.39 |  | 0.39 | 0.20 | 0.39 | 0.78 |  | 1 | 8 | 256 |  | 2 | 4 | 2 | 1 |
| 5 | **Vanc-67** | 0.78 | 1.56 | 6.25 |  | 0.39 | 0.78 | 0.78 | 0.78 |  | 1 | 4 | 16 |  | 2 | 1 | 1 | 1 |
| 5 | **Vanc-68** | 0.78 | 0.39 | 1.56 |  | nd | nd | nd | nd |  | 1 | 16 | 64 |  | nd | nd | nd | nd |
| 5 | **Vanc-69** | 0.78 | 0.78 | 1.56 |  | 0.20 | 0.20 | 0.20 | 0.78 |  | 1 | 8 | 64 |  | 4 | 4 | 4 | 1 |
| 5 | **Vanc-70** | 0.78 | 0.78 | 1.56 |  | 0.20 | 0.20 | 0.39 | 0.20 |  | 1 | 8 | 64 |  | 4 | 4 | 2 | 4 |
| 5 | **Vanc-71** | 0.39 | 0.78 | 1.56 |  | 0.20 | 0.20 | 0.20 | 0.20 |  | 2 | 8 | 64 |  | 4 | 4 | 4 | 4 |
| 5 | **Vanc-72** | 0.39 | 0.78 | 0.78 |  | 0.20 | 0.20 | 0.20 | 1.56 |  | 2 | 8 | 128 |  | 4 | 4 | 4 | 0.5 |
| 5 | **Vanc-73** | 0.78 | 1.56 | 6.25 |  | 0.39 | 0.39 | 0.39 | 0.78 |  | 1 | 4 | 16 |  | 2 | 2 | 2 | 1 |
| 5 | **Vanc-74** | 0.39 | 0.78 | 1.56 |  | 0.20 | 0.20 | 0.20 | 0.78 |  | 2 | 8 | 64 |  | 4 | 4 | 4 | 1 |
| 5 | **Vanc-75** | 0.78 | 0.78 | 1.56 |  | 0.20 | 0.39 | 0.20 | 0.20 |  | 1 | 8 | 64 |  | 4 | 2 | 4 | 4 |
| 5 | **Vanc-76** | 0.39 | 0.78 | 0.78 |  | 0.20 | 0.20 | 0.20 | <0.10 |  | 2 | 8 | 128 |  | 4 | 4 | 4 | >8 |
| 5 | **Vanc-77** | 0.39 | 0.78 | 0.78 |  | 0.20 | 0.20 | 0.20 | <0.10 |  | 2 | 8 | 128 |  | 4 | 4 | 4 | >8 |
| 6 | **Vanc-78** | 0.78 | 3.13 | 3.13 |  | nd | nd | 0.78 | nd |  | 1 | 2 | 32 |  | nd | nd | 1 | nd |
| 6 | **Vanc-79** | 0.78 | 6.25 | 1.56 |  | nd | nd | 0.39 | nd |  | 1 | 1 | 64 |  | nd | nd | 2 | nd |
| 6 | **Vanc-80** | 0.78 | 6.25 | 1.56 |  | nd | nd | 0.78 | nd |  | 1 | 1 | 64 |  | nd | nd | 1 | nd |
| 6 | **Vanc-81** | 0.39 | 6.25 | 0.78 |  | nd | nd | 0.20 | nd |  | 2 | 1 | 128 |  | nd | nd | 4 | nd |
| 6 | **Vanc-82** | 1.56 | >12.5 | 3.13 |  | nd | nd | 0.78 | nd |  | 0.5 | <0.5 | 32 |  | nd | nd | 1 | nd |
| 6 | **Vanc-83** | 0.20 | 1.56 | 0.39 |  | nd | nd | 0.20 | nd |  | 4 | 4 | 256 |  | nd | nd | 4 | nd |
| 7 | **Vanc-84** | 0.10 | 0.39 | 0.78 |  | 0.10 | 0.20 | 0.20 | 0.10 |  | 8 | 16 | 128 |  | 8 | 4 | 4 | 8 |
| 7 | **Vanc-85** | 0.20 | 0.78 | 1.56 |  | 0.20 | 0.39 | 0.20 | 0.10 |  | 4 | 8 | 64 |  | 4 | 2 | 4 | 8 |
| 7 | **Vanc-86** | 0.39 | 0.78 | 1.56 |  | 0.20 | 0.39 | 0.39 | 0.20 |  | 2 | 8 | 64 |  | 4 | 2 | 2 | 4 |
| 7 | **Vanc-87** | 0.20 | 0.78 | 1.56 |  | 0.20 | 0.20 | 0.20 | 0.10 |  | 4 | 8 | 64 |  | 4 | 4 | 4 | 8 |
| 7 | **Vanc-88** | 0.39 | 0.39 | 0.78 |  | 0.10 | 0.39 | 0.20 | 0.10 |  | 2 | 16 | 128 |  | 8 | 2 | 4 | 8 |
| 7 | **Vanc-89** | 0.10 | 0.78 | 0.78 |  | 0.10 | 0.20 | 0.20 | 0.10 |  | 8 | 8 | 128 |  | 8 | 4 | 4 | 8 |
| 7 | **Vanc-90** | 0.10 | 0.39 | 0.78 |  | 0.10 | 0.20 | 0.20 | 0.10 |  | 8 | 16 | 128 |  | 8 | 4 | 4 | 8 |
| 7 | **Vanc-91** | 0.10 | 0.39 | 0.39 |  | 0.10 | 0.10 | 0.20 | 0.10 |  | 8 | 16 | 256 |  | 8 | 8 | 4 | 8 |
| 7 | **Vanc-92** | 0.20 | 0.78 | 0.78 |  | 0.20 | 0.20 | 0.20 | 0.20 |  | 4 | 8 | 128 |  | 4 | 4 | 4 | 4 |
| Control | **Tigecycline** | 3.13 | 3.13 | 3.13 |  | 1.56 | 1.56 | 1.56 | 1.56 |  | 0.3 | 2 | 32 |  | 0.5 | 0.5 | 0.5 | 0.5 |
| Control | **Daptomycin** | >6.25 | >12.5 | 12.5 |  | 12.5 | >12.5 | 6.25 | 12.5 |  | <0.1 | <0.5 | 8 |  | 0.05 | >0.05 | 0.1 | 0.05 |
| Control | **Linezolid** | >3.13 | 3.13 | 6.25 |  | 6.25 | 6.25 | 6.25 | 12.5 |  | <0.2 | 2 | 16 |  | 0.1 | 0.1 | 0.1 | 0.05 |
| Control | **Vancomycin** | 0.78 | 6.25 | 100 |  | 0.78 | 0.78 | 0.78 | 0.78 |  | 1 | 1 | 1 |  | 1 | 1 | 1 | 1 |
|  |  | **VSSA** | **VISA/ HIP5827** | **VRSA** |  | **MRSA KS2** | **MRSA KS6** | **MRSA KS8** | **MRSA KS1** |  | **VSSA** | **VISA/ HIP5827** | **VRSA** |  | **MRSA KS2** | **MRSA KS6** | **MRSA KS8** | **MRSA KS1** |

**Table S5. *In vitro* therapeutic indices against VRE and MRSA.** The MIC ranges of five selected vancomycin analogues and four control antibiotics have been determined against 33 clinical VRE isolates (top) and 24 clinical MRSA isolates (bottom). The MIC_50_ value is determined as the median MIC value against the respective set of isolates. The *in vitro* therapeutic index TI is calculated as the ratio of IC_50_ over MIC_50_.

|  | Vanc-24 | Vanc-66 | Vanc-39 | Vanc-42 | Vanc-83 | Linezolid | Tigecycline | Daptomycin | Vancomycin |  |
| --- | --- | --- | --- | --- | --- | --- | --- | --- | --- | --- |
| Range (µM) | 3.13 - 50 | 1.56 - 12.5 | 0.78 - 12.5 | 0.39 - 6.25 | 0.78 - 3.13 | 3.13 - 50 | ≤0.02 - 0.78 | 3.13 - 12.5 | 50 - >800 | VRE |
| MIC_50_ (µM) | 6.25 | 3.13 | 3.13 | 1.56 | 2 | 6.25 | 0.2 | 6.25 | 800 |  |
| IC_50_ (µM) | 165 | 190 | 250 | 173 | 107 | 208 | 14 | 250 | 250 |  |
| TI (IC_50_/MIC_50_) | 26 | 61 | 80 | 111 | 69 | 33 | 70 | 40 | 0.3 |  |
| Range (µM) | 0.10 – 0.78 | 0.20 – 3.13 | 0.20 – >0.78 | 0.10 – 0.39 | 0.10 – 0.78 | 6.25 – 25 | 0.78 – 6.25 | 0.78 – 12.5 | 0.78 – 6.25 | MRSA |
| MIC_50_ (µM) | 0.20 | 0.78 | 0.39 | 0.20 | 0.20 | 25 | 1.56 | 0.78 | 1.56 |  |
| IC_50_ (µM) | 165 | 190 | 250 | 173 | 107 | 208 | 14 | 250 | 250 |  |
| TI (IC_50_/MIC_50_) | 825 | 238 | 641 | 865 | 535 | 8 | 9 | 321 | 160 |  |

**Table S6. Minimal inhibitory concentrations of a subset of analogues against *C. difficile*.** The MIC of a subset of 15 vancomycin analogues was determined against representative strains of the two most prevalent *C. difficile* ribotypes using a plate drop assay. Daptomycin and vancomycin were included as control antibiotics.

|  |  |  |  |  |  |
| --- | --- | --- | --- | --- | --- |
|  | **MIC (µM)** | |  | **MIC-fold improvement vs. vancomycin** | |
|  | ***Clostridium difficile*** | |  | ***Clostridium difficile*** | |
| **Vancomycin analogue** | **ATJ ribotype 014/20** | **AIU ribotype 027** |  | **ATJ ribotype 014/20** | **AIU ribotype 027** |
| **Vanc- N** | 6.25 | 1.56 |  | 5.6 | 29 |
| **Vanc- 4** | 6.25 | >25 |  | 5.6 | <1.8 |
| **Vanc- 14** | 1.56 | 1.56 |  | 22 | 29 |
| **Vanc- 21** | >25 | 12.5 |  | <1.4 | 4 |
| **Vanc- 22** | 6.25 | 6.25 |  | 5.6 | 7 |
| **Vanc- 24** | 3.13 | 3.13 |  | 11 | 14 |
| **Vanc- 25** | 6.25 | 12.5 |  | 5.6 | 4 |
| **Vanc- 29** | 6.25 | 6.25 |  | 5.6 | 7 |
| **Vanc- 34** | 1.56 | 1.56 |  | 22 | 29 |
| **Vanc- 39** | 1.56 | 3.13 |  | 22 | 14 |
| **Vanc- 42** | 1.56 | 0.78 |  | 22 | 58 |
| **Vanc- 43** | 1.56 | 1.56 |  | 22 | 29 |
| **Vanc- 48** | 0.78 | 0.78 |  | 45 | 58 |
| **Vanc- 59** | 1.56 | 1.56 |  | 22 | 29 |
| **Vanc- 66** | 0.78 | 0.78 |  | 45 | 58 |
| **Daptomycin** | 1.56 | 1.56 |  | 22 | 29 |
| **Vancomycin** | 35 | 45 |  | 1 | 1 |
|  |  |  |  |  |  |

**Table S7. Minimum inhibitory concentrations against VanB- and VanC1-type VRE strains.** The susceptibility of five VanB strains and one VanC1 strain was analyzed for Vanc-24, Vanc-39, Vanc-42, Vanc-66 and Vanc-83. Control antibiotics (linezolid, tigecycline, daptomycin, vancomycine) were included.

|  |  |  |  |  |  |  |
| --- | --- | --- | --- | --- | --- | --- |
|  | **MIC (µM)** | | | | | |
| Strain | **VE 148** | **VE 149** | **VE 150** | **VE 152** | **VE 157** | **VE 82** |
| **Vanc-24** | 3.13 | 1.56 | 1.56 | 1.56 | 1.56 | 0.39 |
| **Vanc-39** | 3.13 | 0.78 | 1.56 | 0.78 | 0.78 | 0.78 |
| **Vanc-42** | 3.13 | 3.13 | 3.13 | 3.13 | 3.13 | 1.56 |
| **Vanc-66** | 1.56 | 0.78 | 0.78 | 0.78 | 0.78 | 0.78 |
| **Vanc-83** | 1.56 | 1.56 | 1.56 | 1.56 | 1.56 | 0.78 |
| **Linezolid** | 12.5 | 12.5 | 12.5 | 12.5 | 12.5 | 12.5 |
| **Tigecycline** | 3.13 | 1.56 | 1.56 | 1.56 | 1.56 | 0.78 |
| **Daptomycin** | 25 | 25 | 25 | 25 | 12.5 | 12.5 |
| **Vancomycin** | 100 | 50 | 100 | 50 | 100 | 3.13 |
| Resistance mechanism | **VanB** | **VanB** | **VanB** | **VanB** | **VanB** | **VanC1** |
|  |  |  |  |  |  |  |

**Table S8. Comparison of the minimum inhibitory concentrations in the absence and presence of vancomycin.** Vancomycin (10 µM) was included in a MIC assay against VRE39 to ensure the induction of the *vanA* operon and the production of modified cell wall precursors under the hypothesis that vancomycin analogues would not induce the *vanA* operon.

|  |  |  |
| --- | --- | --- |
|  | **MIC (µM)** | |
|  | **0 µM vancomycin** | **10 μM vancomycin** |
| **Vanc-24** | 3.13 | 6.25 |
| **Vanc-39** | 1.56 | 6.25 |
| **Vanc-42** | 1.56 | 1.56 |
| **Vanc-66** | 3.13 | 3.13 |
| **Vanc-83** | 1.56 | 3.13 |
| **Vancomycin** | 800 | 400 |
|  |  |  |

**Supplementary text.** Experimental details about the synthesis of all vancomycin analogues and assessment of their purity.

General Remarks.

Air- and/or moisture-sensitive reactions were carried out under an argon atmosphere in oven-dried glassware and with anhydrous solvents. All compounds were purchased from commercial sources unless otherwise noted, and used without further purification.

Vancomycin was purchased from A K Scientific, Inc. USA. Boc-glycine was perchased from Irish biotech GmbH, Germany. Piperidine, pyridine, bis(triphenylphosphine)palladium(II) dichloride, palladium(II) acetate, N-methyl morpholine, N-boc-ethylenediamine, HATU, sodium azide, ethynyltrimethylsilane, triethylamine, copper(I) iodide, alkyl bromide(s), tetrabutylammonium bromide, potassium acetate, N-boc -p-phenylenediamine, benzenesulfonyl chloride, phenylamine(s) and DIPEA were purchased from Sigma Aldrich, Belgium. N-boc-p-phenylenediamine, sodium hydroxide, DMSO, DCM, THF, chloroform, diethyl ether, tetrabutylammonium fluoride, lithium hydroxide, and N-boc-1,3 propanediamine were purchased from Acros Organics, Belgium.

**Equipments used.**

NMR spectra were recorded on a Bruker Avance 300 MHz, 400MHz and 600 MHz instrument using DMSO-d_6_, CDCl_3,_ D_2_O, or methanol-d_4_ as solvent unless otherwise stated. The ^1^H and ^13^C chemical shifts are reported in parts per million relative to tetramethylsilane using the residual solvent signal as an internal reference. For, TLC, analytical TLC plates (Alu-gram SIL G/UV_254_) and 70-230 mesh silica gel (E. M. Merck) were used.

**Mass spectrometry.**

Mass spectra (MW < 500) were recorded by using a Kratos MS50TC and a Kratos Mach III system. The ion source temperature was 150-250 °C, as required. High-resolution electron impact (EI) mass spectra were performed with a resolution of 10,000. The low-resolution spectra were obtained with an HP5989A MS instrument.

**ESI-MS (MW <2000)**: a Thermo Electron LCQ Advantage apparatus with Agilent 1100 pomp- and injection system coupled to an Xcalibur data analysing software was used. MeOH or ACN were used as eluents.

**FT-ICR (MW >2000):** Bruker, APEX-Qe with data analysis software was used.

**LC-MS:** an Alltech Prevail RP-C18, 5 µm, 150 mm x 2.1 mm, and Phenomenex Luna RP-C18, 3µm, 150mm x 2.0 mm columns were used. System 1: Agilent 1100 degasser, binary pomp and autosampler coupled to a Thermo Electron LCQ Advantage mass spectrometer with APCI - or ESI source, and Xcalibur data analysis software. System 2: Agilent 1100 degasser, quaternary pomp, auto sampler, UV-DAD detector and thermostatted column module coupled to Agilent 6110 single-quadrupole MS and Agilent LC/MSD chemstation software.

**RP-HPLC:** a Waters Delta 600 analytical/preparative system equipped with a Waters 996 photodiode array detector was used. Preparative columns: Alltech C18 Prevail 5 µm, 150 mm x 22 mm, and Phenomenex Luna C18, 5µm, 150 mm x 22 mm.

**General experimental methods.**

**Synthetic Scheme**

**Scheme 1.**

**Table S8:** Structure of side chain R2NHR1NH2 for vancomycin analogues Vanc-A to Vanc-tridecane

| No. |  | **R^1^** | **R^2^** | Calculated average mass | Found ESI-MS [m/z] | Molecular formula |
| --- | --- | --- | --- | --- | --- | --- |
| 1 | **Vanc- 1** | -COCH_2_- |  | 1597.4155 | 1598.5 [M+H]^+^ | C_74_H_83_Cl_2_N_11_O_25_ |
| 2 | **Vanc- 2** | -COCH_2_- |  | 1629.88 | 1630.4 [M+H]^+^ | C_75_H_84_Cl_3_N_11_O_24_ |
| 3 | **Vanc- 3** | -COCH_2_- |  | 1581.41 | 1582.3  [M+H]^+^ | C_74_H_83_Cl_2_N_11_O_24_ |
| 4 | **Vanc- 4** | -COCH_2_- |  | 1595.44 | 1596.4  [M+H]^+^ | C_75_H_85_Cl_2_N_11_O_24_ |
| 5 | **Vanc- 5** | -COCH_2_- |  | 1611.44 | 1612.3  [M+H]^+^ | C_74_H_83_Cl_2_N_11_O_25_ |
| 6 | **Vanc- 6** | -COCH_2_- |  | 1615.86 | 1616.3  [M+H]^+^ | C_74_H_82_Cl_3_N_11_O_24_ |
| 7 | **Vanc- 7** | -COCH_2_- |  | 1641.46 | 1642.3  [M+H]^+^ | C_76_H_87_Cl_2_N_11_O_26_ |
| 8 | **Vanc- 8** | -COCH_2_- |  | 1666.52 | 1667.3  [M+H]^+^ | C_78_H_90_Cl_2_N_12_O_25_ |
| 9 | **Vanc- 9** | -COCH_2_- |  | 1623.49 | 1624.4  [M+H]^+^ | C_77_H_89_Cl_2_N_11_O_24_ |
| 10 | **Vanc- 10** | -COCH_2_- |  | 1599.40 | 1600.3  [M+H]^+^ | C_74_H_82_Cl_2_FN_11_O_24_ |
| 11 | **Vanc- 11** | -COCH_2_- |  | 1609.46 | 1610.3  [M+H]^+^ | C_76_H_87_Cl_2_N_11_O_24_ |
| 12 | **Vanc- 12** | -COCH_2_- |  | 1611.44 | 1612.3  [M+H]^+^ | C_75_H_85_Cl_2_N_11_O_25_ |
| 13 | **Vanc-13** | -COCH_2_- |  | 1609.46 | 1609.5  [M+]^+^ | C_76_H_87_Cl_2_N_11_O_24_ |
| 14 | **Vanc-14** | -COCH_2_- |  | 1650.30 | 1650.2  [M+H]^+^ | C_74_H_81_Cl_4_N_11_O_24_ |
| 15 | **Vanc-15** | -COCH_2_- |  | 1609.46 | 1610.4  [M+H]^+^ | C_76_H_87_Cl_2_N_11_O_24_ |
| 16 | **Vanc-16** | -COCH_2_- |  | 1617.39 | 1618.3  [M+H]^+^ | C_74_H_81_Cl_2_F_2_N_11_O_24_ |
| 17 | **Vanc-17** | -COCH_2_- |  |  | 1642.2  [M+H]^+^ | C_76_H_87_Cl_2_N_11_O_26_ |
| 18 | **Vanc-18** | -COCH_2_- |  | 1611.44 | 1612.3  [M+H]^+^ | C_75_H_85_Cl_2_N_11_O_25_ |
| 19 | **Vanc-19** | -COCH_2_- |  | 1623.49 | 1624.3  [M+H]^+^ | C_77_H_89_Cl_2_N_11_O_24_ |
| 20 | **Vanc-20** | -CH_2_CH_2_- |  | 1655.92 | 1656.3  [M+H]^+^ | C_77_H_86_Cl_3_N_11_O_24_ |
| 21 | **Vanc-21** | -CH_2_CH_2_- |  | 1621.47 | 1622.3  [M+H]^+^ | C_77_H_87_Cl_2_N_11_O_24_ |
| 22 | **Vanc-22** | -CH_2_CH_2_- |  | 1639.47 | 1640.2  [M+H]^+^ | C_77_H_86_Cl_3_F_11_N_11_O_24_ |
| 23 | **Vanc-23** | -CH_2_CH_2_- |  | 1651.40 | 1652.4  [M+H]^+^ | C_78_H_89_Cl_2_N_11_O_25_ |
| 24 | **Vanc-24** | -CH_2_CH_2_CH_2_- |  | 1648.50 | 1651.6  [M+H]^+^ | C_78_H_88_Cl_2_N_12_O_24_ |
| 25 | **Vanc-25** | -CH_2_CH_2_CH_2_- |  | 1649.49 | 1650.7  [M+H]^+^ | C_78_H_87_Cl_2_N_11_O_25_ |
| 26 | **Vanc-26** | -CH_2_CH_2_CH_2_- |  | 1665.55 | 1666.6  [M+H]^+^ | C_78_H_87_Cl_2_N_11_O_24_S |
| 27 | **Vanc-27** | -CH_2_CH_2_CH_2_- |  | 1660.51 | 1660.6  [M+H]^+^  830.90 [2M+H]^2+^ | C_79_H_88_Cl_2_N_12_O_24_ |
| 28 | **Vanc-28** | -CH_2_CH_2_CH_2_- |  | 1659.52 | 1660.6  [M+H]^+^ | C_80_H_89_Cl_2_N_11_O_24_ |
| 29 | **Vanc-29** | -CH_2_CH_2_CH_2_- |  | 1689.55 | 845.0 [M+2H]^+^ | C_81_H_91_Cl_2_N_11_O_25_ |
| 30 | **Vanc-30** | -CH_2_CH_2_CH_2_- |  | 1673.54081 | 837.9 [M+2H]^+^ | C_80_H_89_Cl_2_N_11_O_25_ |
| 31 | **Vanc-31** | -CH_2_CH_2_CH_2_- |  | 1707.65 | 1708.3 [M+H]^+^ | C_83_H_101_Cl_2_N_11_O_24_ |
| 32 | **Vanc-32** | -CH_2_CH_2_CH_2_- |  | 1677.46 | 1677.2  [M+]^+^  1679.2  [M+H]^+^ | C_77_H_86_Cl_2_F_3_N_11_O_24_ |
| 33 | **Vanc-33** | -CH_2_CH_2_CH_2_- |  | 1685.56 | 842.0 [2M+H]^2+^ | C_82_H_91_Cl_2_N_11_O_24_ |
| 34 | **Vanc-34** | -CH_2_CH_2_CH_2_- |  | 1703.55 | 1703.0 [M+H]^+^ | C_82_H_90_Cl_2_FN_11_O_24_ |
| 35 | **Vanc-35** | -CH_2_CH_2_CH_2_- |  | 1761.66 | 1762.7  [M+H]^+^ | C_88_H_95_Cl_2_N_11_O_24_ |
| 36 | **Vanc-36** | -CH_2_CH_2_- |  | 1651.50 | 1653.0 [M+H]^+^ | C_78_H_89_Cl_2_N_11_O_25_ |
| 37 | **Vanc-37** | -COCH_2_- |  | 1661.54 | 1660.9 monoisotopic mass  830.9 [M+H]^2+^ | C_80_H_91_Cl_2_N_11_O_24_ |
| 38 | **Vanc-38** | -COCH_2_- |  | 1681.53 | 1682.8 [M+H]^+^  840.8 [M+H]^2+^ | C_82_H_87_Cl_2_N_11_O_24_ |
| 39 | **Vanc-39** | -COCH_2_- |  | 1637.52 | 1638.5  [M+H]^+^ | C_78_H_91_Cl_2_N_11_O_24_ |
| 40 | **Vanc-40** | -COCH_2_- |  | 1649.41 | 1650.4  [M+H]^+^ | C_75_H_82_Cl_2_F_3_N_11_O_24_ |
| 41 | **Vanc-41** | -COCH_2_- |  | 1631.47 | 1631.7  [M+H]^+^ | C_78_H_85_Cl_2_N_11_O_24_ |
| 42 | **Vanc-42** | -COCH_2_- |  | 1669.52 | 1670.5  [M+H]^+^ | C_81_H_87_Cl_2_N_11_O_24_ |
| 43 | **Vanc-43** | -COCH_2_- |  | 1672.52 | 1673.5  [M+H]^+^ | C_80_H_88_Cl_2_N_12_O_24_ |
| 44 | **Vanc-44** | -COCH_2_- |  | 1614.45522 | 1616.84  [M+H]^+^ | C_73_H_81_Cl_3_N_12_O_24_ |
| 45 | **Vanc-45** | -CH_2_CH_2_CH_2_- |  | 1666.49 | 1667.6  [M+H]^+^ | C_78_H_87_Cl_2_N_12_O_24_ |
| 46 | **Vanc-46** | -CH_2_CH_2_- |  | 1683.54 | 1684.6  [M+H]^+^ | C_82_H_89_Cl_2_N_11_O_24_ |
| 47 | **Vanc-47** | -CH_2_CH_2_- |  | 1699.59 | 1700.6  [M+H]^+^ | C_83_H_93_Cl_2_N_11_O_24_ |
| 48 | **Vanc-48** | -CH_2_CH_2_- |  | 1701.60 | 1702.6  [M+H]^+^ | C_82_H_95_Cl_2_N_11_O_24_ |
| 49 | **Vanc-49** | -CH_2_CH_2_CH_2_- |  | 1679.62 | 1680.7  [M+H]^+^ | C_81_H_97_Cl_2_N_11_O_24_ |
| 50 | **Vanc-50** | -CH_2_CH_2_CH_2_- |  | 1681.57 | 1682.6  [M+H]^+^ | C_80_H_95_Cl_3_N_11_O_25_ |
| 51 | **Vanc-51** | -CH_2_CH_2_- |  | 1689.44 | 1690.5  [M+H]^+^ | C_77_H_85_Cl_4_N_11_O_24_ |
| 52 | **Vanc-52** | -CH_2_CH_2_CH_2_- |  | 1649.49 | 1649.6  [M+H]^+^ | C_77_H_87_Cl_3_N_13_O_24_ |
| 53 | **Vanc-53** | -CH_2_CH_2_- |  | 1635.50 | 1636.6  [M+H]^+^ | C_78_H_89_Cl_2_N_11_O_24_ |
| 54 | **Vanc-54** | -CH_2_CH_2_- |  | 1776.65 | 1778.1  [M+H]^+^ | C_83_H_92_Cl_2_N_12_O_26_S |
| 55 | **Vanc-55** | -CH_2_CH_2_- |  | 1790.68 | 1762.4 [M+H]^+^, 1194.65[M+2H]^3+^, 865.40 [M+H]^2+^ | C_84_H_94_Cl_2_N_12_O_26_S |
| 56 | **Vanc-56** | -CH_2_CH_2_- |  | 1818.73 | 1819.4  [M+H]^+^ | C_86_H_98_Cl_2_N_12_O_26_S |
| 57 | **Vanc-57** |  |  | 1687.53 | 1689.3  [M+H]^+^ | C_81_H_89_Cl_2_N_11_O_25_ |
| 58 | **Vanc-58** | -COCH_2_- |  | 1698.56 | 1699.4  [M+H]^+^ | C_82_H_90_Cl_3_N_12_O_24_ |
| 59 | **Vanc-59** | -COCH_2_- |  | 1681.53 | 1682.8  [M+H]^+^ | C_82_H_87_Cl_2_N_11_O_24_ |
| 60 | **Vanc-60** | -COCH_2_- |  | 1736.59 | 1737.6  [M+H]^+^ | C_80_H_88_Cl_2_N_12_O_26_S |
| 61 | **Vanc-61** | -COCH_2_- |  | 1804.42 | 1805.3  [M+H]^+^ | C_80_H_86_Cl_4_N_12_O_26_S |
| 62 | **Vanc-62** | -COCH_2_- |  | 1771.03 | 1810.9 [M+K]^+^ | C_80_H_87_Cl_3_N_12_O_26_S |
| 63 | **Vanc-63** | -CH_2_CH_2_CH_2_- |  | 1814.70 | 1815.6  [M+H]^+^ | C_86_H_94_Cl_2_N_12_O_26_S |
| 64 | **Vanc-64** | -CH_2_CH_2_- |  | 1845.54 | 1845.3  [M+H]^+^ | C_83_H_90_Cl_4_N_12_O_26_S |
| 65 | **Vanc-65** | -CH_2_CH_2_CH_2_- |  | 1828.72 | 1829.7  [M+H]^+^ | C_87_H_96_Cl_2_N_12_O_26_S |
| 66 | **Vanc-66** | -CH_2_CH_2_CH_2_- |  | 1856.78 | 1857.5  [M+H]^+^ | C_89_H_100_Cl_2_N_12_O_26_S |
| 67 | **Vanc-67** | -COCH_2_- |  | 1626.41 | 1627.2  [M+H]^+^ | C_74_H_82_Cl_2_N_12_O_26_ |
| 68 | **Vanc-68** | -COCH_2_- |  | 1647.51 | 1647.0  [M+]^+^ | C_79_H_89_Cl_2_N_11_O_24_ |
| 69 | **Vanc-69** | -COCH_2_- |  | 1673.55 | 1673.2  [M+H]^+^ | C_81_H_91_Cl_2_N_11_O_24_ |
| 70 | **Vanc-70** | -COCH_2_- |  | 1645.50 | 1646.4  [M+H]^+^ | C_79_H_87_Cl_2_N_11_O_24_ |
| 71 | **Vanc-71** | -CH_2_CH_2_CH_2_- |  | 1673.51 | 1674.8  [M+H]^+^ | C_80_H_87_Cl_2_N_11_O_24_ |
| 72 | **Vanc-72** | -COCH_2_- |  | 1754.58 | 1755.5  [M+H]^+^ | C_83_H_90_Cl_2_N_14_O_25_ |
| 73 | **Vanc-73** | -CH_2_CH_2_CH_2_- |  | 1665.57 | 1666.6  [M+H]^+^ | C_80_H_95_Cl_2_N_11_O_24_ |
| 74 | **Vanc-74** | -COCH_2_- |  | 1651.54 | 1652.5  [M+H]^+^ | C_79_H_93_Cl_2_N_11_O_24_ |
| 75 | **Vanc-75** | -COCH_2_- |  | 1665.57 | 1666.6  [M+H]^+^ | C_80_H_95_Cl_2_N_11_O_24_ |
| 76 | **Vanc-76** | -COCH_2_- |  | 1738.58 | 1739.8  [M+H]^+^ | C_83_H_90_Cl_2_N_14_O_24_ |
| 77 | **Vanc-77** | -COCH_2_- |  | 1724.53 | 1725.8  [M+H]^+^ | C_82_H_88_Cl_2_N_14_O_24_ |
| 78 | **Vanc-78** | -COCH_2_- |  | 1978.75 | 1979.4  [M+H]^+^ | C_90_H_102_Cl_2_N_14_O_33_ |
| 79 | **Vanc-79** | -COCH_2_- |  | 1794.75 | 1795.7  [M+H]^+^ | C_85_H_108_Cl_2_N_13_O_24_^+^ |
| 80 | **Vanc-80** | -COCH_2_- |  | 1778.67 | 1779.9  [M+H]^+^ | C_84_H_106_Cl_2_N_13_O_24_^+^ |
| 81 | **Vanc-81** | -COCH_2_- |  | 1822.72 | 1822.2 [M+H]^+^ | C_87_H_112_Cl_2_N_13_O_24_^+^ |
| 82 | **Vanc-82** | -COCH_2_- |  | 1709.58 | 1710.5  [M+H]^+^ | C_79_H_95_Cl_2_N_13_O_24_ |
| 83 | **Vanc-83** | -COCH_2_- |  | 1657.51 | 1658.8  [M+H]^+^ | C_80_H_87_Cl_2_N_11_O_24_ |
| 84 | **Vanc-84** | -COCH_2_- |  | 1691.95 | 1692.9  [M+H]^+^ | C_80_H_86_Cl_3_N_11_O_24_ |
| 85 | **Vanc-85** | -COCH_2_- |  | 1699.54 | 1699.2, 1701.8 [M+H]^+^ | C_82_H_89_Cl_2_N_11_O_25_ |
| 86 | **Vanc-86** | -COCH_2_- |  | 1701.53 | 1701.8  [M+H]^+^ | C_82_H_91_Cl_2_N_11_O_25_ |
| 87 | **Vanc-87** | -COCH_2_- |  | 1669.54 | 1670.8  [M+H]^+^ | C_81_H_89_Cl_2_N_11_O_24_ |
| 88 | **Vanc-88** | -COCH_2_- |  | 1683.52 | 1684.4  [M+H]^+^ | C_81_H_87_Cl_2_N_11_O_25_ |
| 89 | **Vanc-89** | -COCH_2_- |  | 1683.50 | 1684.4  [M+H]^+^ | C_81_H_85_Cl_3_N_11_O_25_ |
| 90 | **Vanc-90** | -COCH_2_- |  | 1669.52 | 1670.5  [M+H]^+^ | C_81_H_87_Cl_2_N_11_O_24_ |
| 91 | **Vanc-91** | -COCH_2_- |  | 1718.59 | 1719.8  [M+H]^+^ | C_81_H_94_Cl_2_N_14_O_24_ |
| 92 | **Vanc-92** | -COCH_2_- |  | 1732.62 | 1733.3  [M+H]^+^ | C_82_H_96_Cl_2_N_14_O_24_ |
| 93 | **Vanc- B** | -CH_2_CH_2_CH_2_- |  | 1585.50 | 1586.0 [M+H]^+^,1059.8 [2M+3H]^3+^ | C_74_H_87_Cl_2_N_11_O_24_ |
| 94 | **Vanc- N** | -COCH_2_- |  | 1605.50 | 1606.9 [M+H]^+^ | C_76_H_83_Cl_2_N_11_O_24_ |
| 95 | **Vanc- Q** | -CH_2_CH_2_- |  | 1643.53 | 1644.9 [M+H]^+^ | C_79_H_87_Cl_2_N_11_O_24_ |
| 96 | **Vanc- R** | -COCH_2_- |  | 1705.54 | 1706.2 [M+H]^+^ | C_84_H_89_Cl_2_N_11_O_24_ |
| 97 | **Vanc- S** | -CH_2_CH_2_CH_2_- |  | 1707.56 | 1709.0 [M+H]^+^ | C_84_H_91_Cl_2_N_11_O_24_ |
| 98 | **Vanc- T** | -CH_2_CH_2_CH_2_- |  | 1681.54 | 1683.9 [M+H]^+^ | C_82_H_89_Cl_2_N_11_O_24_ |

**Table S9:** Structure of side chain R2NHR1NH2 for vancomycin analogues Vanc-A to Vanc-tridecane

| No. | Entry | R^2^NHR^1^NH_2_ | Calculated average mass | Found ESI-MS [m/z] | Molecular formula |
| --- | --- | --- | --- | --- | --- |
| 1 | Vanc-A |  | 1571.5 | 1572.2 [M+H]^+^ , 1048.2 [2M+3H]^3+^ | C_73_H_85_Cl_2_N_11_O_24_ |
| 2 | Vanc-B |  | 1585.50 | 1586.0 [M+H]^+^,1059.8 [2M+3H]^3+^ | C_74_H_87_Cl_2_N_11_O_24_ |
| 3 | Vanc-C |  | 1514.5 | 1515.1 [M+H]^+^ | C_71_H_82_Cl_2_N_10_O_23_ |
| 4 | Vanc-D |  | 1599.5 | 1600.9 [M+H]^+^ | C_75_H_89_Cl_2_N_11_O_24_ |
| 5 | Vanc-E |  | 1613.5 | 1614.5 [M+H]^+^ | C_76_H_91_Cl_2_N_11_O_24_ |
| 6 | Vanc-F |  | 1613.5 | 1614.0 [M+H]^+^ | C_76_H_91_Cl_2_N_11_O_24_ |
| 7 | Vanc-G |  | 1486.5 | 1487.6 [M+H]^+^ | C_69_H_78_Cl_2_N_10_O_23_ |
| 8 | Vanc-J |  | 1543.5 | 1544.9 [M+H]^+^ | C_71_H_81_Cl_2_N_11_O_24_ |
| 9 | Vanc-K |  | 1557.5 | 1558.9 [M+H]^+^ | C_72_H_83_Cl_2_N_11_O_24_ |
| 10 | Vanc-L |  | 1597.5 | 1598.7 [M+H]^+^ | C_75_H_87_Cl_2_N_11_O_24_ |
| 11 | Vanc-M |  | 1500.5 | 1501.3 [M+H]^+^ | C_70_H_80_Cl_2_N_10_O_23_ |
| 12 | Vanc-H5A |  | 1627.5 | 1628.9 [M+H]^+^ ,1086.5 [2M+3H]^3+^ | C_77_H_93_Cl_2_N_11_O_24_ |
| 13 | Vanc-Tridecane |  | 1731.5 | 1731.8 [M]^+^, 1156.0 [2M+3H]^3+^ | C_81_H_101_Cl_2_N_11_O_27_ |

**Synthesis of Vancomycin analogues (modification at C-terminus of vancomycin)**

**Procedure:**

Vancomycin.HCl (75 mg, 0.051 mmol), EDC·HCl (12 mg, 0.0.067 mmol) and HOAt (9 mg, 0.067mmol) were dissolved in DMF (5 mL) and the mixture was stirred for 5 min. To this solution were added: a solution of linker R^2^NHR^1^NH_2_ (1.1 equiv) in DMF (1 mL) and *N*-methyl morpholine (till pH 8), and the mixture was stirred for 7-8h at rt. The reaction was monitored by ESI-MS. DMF was removed *in* *vacuo* and the residue was purified by preparative RP-HPLC (0-40% ACN/H_2_O with 0.1 % HCOOH in 40 min). The retention time was in the range of 27-35 min. The acetonitrile was removed *in vacuo* and the remaining water was lyophilised affording compounds **11a-w** as a white powder in 30-50% yield. Compounds thus obtained were characterized by mass spectrometric analysis.

**Synthesis of linker R^2^NHR^1^NH_2_**

Synthesis was classified into 3 categories based upon the selection of **R^1^**.

R^1^ was prepared from:

1) N- Boc Glycine

2) N-Boc-1,2 ethylenediamine

3) N-Boc-1,3 propanediamine

1. **From Boc glycine**

**Scheme 2.**

**Reagents and conditions:** i) Ethyl chloroformate, THF, TEA, overnight, 0 ^o^C to rt; ii) H_2_O/dioxane (9:1), 140 ^o^C, 3h; ii’) 4N HCl in 1,4-dioxane, DCM, 0 ^o^C to rt, 1h.

**Detailed synthetic route for the development of vancomycin analogues side chain R^2^NHR^1^NH_2_**

- 1. **Synthetic route for vancomycin analogues with substituents derived from aryl amines (11n, Vanc- 1-19, 37-44, 58-59, 67-71, 74, 75, 83-90).**

**Scheme 3.**

**Reagents and conditions:** i) Ethyl chloroformate, *N*-Boc-glycine **2**, THF, TEA, overnight, 0 ^o^C to rt; ii) If R= terminal alkyne: H_2_O/dioxane (9:1), 140 ^o^C, 3h; ii’) 4N HCl in 1,4-dioxane, DCM, 0 ^o^C to rt, 1h.

**Procedure:**

**Step-i**

To a solution of N-Boc-Glycine **2** (1.44 mmol) in THF (2 mL) at 0 ^o^C were added: triethylamine (1.44 mmol) and ethyl chloroformate (1.44 mmol) over a period of 10 min, and the reaction mixture was stirred for 30 min. After the addition of the phenyl amine **1** (1.44 mmol), the mixture was stirred for an additional 1 h at 0 ^o^C. Then, the reaction mixture was warmed to room temperature, and kept stirring overnight. After completion of the reaction, the solvent was evaporated under reduced pressure; the residue was dissolved in ethyl acetate (15mL x 2) and washed with brine solution (15 mL). The organic layer was dried over Na_2_SO_4_ and the solvent was evaporated under reduced pressure. Crude product **3** was purified by column chromatography using EA / Heptane, (40/60) as eluents. Yield: 65-85 %.^1^

**Step-ii** (if R= terminal alkyne)

Compound **3** (0.5 mmol) was dissolved in 0.5 mL dioxane and 4.5 mL H_2_O in a screw-capped vial and heated at 140 ^o^C for 3h. After completion of the reaction, the reaction mass was cooled to room temperature hence product **4** fell out as white- to yellow-colored solid (depending upon the substitution). It was filtered through a buchner funnel, washed with diethyl ether and dried under vacuum.^2^

**Step-ii‘**

Compound **3** was dissolved in CH_2_Cl_2_ (5 mL), HCl-dioxane (6 M solution, 4 mL) was added, and the mixture was stirred at rt for 3-4h. The reaction was monitored by TLC (MeOH/DCM, 1:9, Rf = 0.1). After completion of the reaction, the solvents were removed *in vacuo* and the product **4** was washed with diethyl ether and dried *in* *vacuo*. Yield: 80-95%

**Table S10**. Vancomycin analogues with substituents derived from aryl amines

| Entry | Product | R | Yield %  **3a-aah** | Yield %  **4a-aah** | Vancomycin analogue |
| --- | --- | --- | --- | --- | --- |
| 1 | **4a** | 4-OH-Ph | 45 | 95 | Vanc-1 |
| 2 | **4b** | 2-Cl, 4-CH_3_-Ph | 55 | 97 | Vanc-2 |
| 3 | **4c** | Ph | 36 | 92 | Vanc-3 |
| 4 | **4d** | 4-CH_3_-Ph | 52 | 89 | Vanc-4 |
| 5 | **4e** | 4-OCH_3_-Ph | 57 | 91 | Vanc-5 |
| 6 | **4f** | 4-Cl-Ph | 61 | 95 | Vanc-6 |
| 7 | **4g** | 3,4-di-OCH_3_-Ph | 68 | 98 | Vanc-7 |
| 8 | **4h** | 4-phenyl morpholine | 70 | 97 | Vanc-8 |
| 9 | **4i** | 2,4,6 tri-CH_3_-Ph | 61 | 85 | Vanc-9 |
| 10 | **4j** | 3-F-Ph | 59 | 91 | Vanc-10 |
| 11 | **4k** | 3,5-di-CH_3_-Ph | 62 | 92 | Vanc-11 |
| 12 | **4l** | 3-OCH_3_-Ph | 62 | 95 | Vanc-12 |
| 13 | **4m** | 4-C2H_5_-Ph | 63 | 91 | Vanc-13 |
| 14 | **4n** | 2,4-di-Cl-Ph | 48 | 92 | Vanc-14 |
| 15 | **4o** | 2,4-di-CH_3_-Ph | 51 | 90 | Vanc-15 |
| 16 | **4p** | 2,4-di-F-Ph | 59 | 94 | Vanc-16 |
| 17 | **4q** | 3,5-di-OCH_3_-Ph | 67 | 96 | Vanc-17 |
| 18 | **4r** | 2-OH, 5-CH_3_-Ph | 63 | 86 | Vanc-18 |
| 19 | **4s** | 2-ethyl, 6-methyl-Ph | 61 | 90 | Vanc-19 |
| 20 | **4t** | 4-C_4_H_9_-Ph | 72 | 92 | Vanc-39 |
| 21 | **4u** | 4-CF_3_-Ph | 70 | 93 | Vanc-40 |
| 22 | **4v** | naphthalene-1-amine | 69 | 94 | Vanc-41 |
| 23 | **4w** | 9*H*-floren-2-amine | 69 | 95 | Vanc-42 |
| 24 | **4x** | 4-*N*-phenylaniline | 70 | 90 | Vanc-43 |
| 25 | **4y** | 5-Cl-pyridin-2-yl | 67 | 90 | Vanc-44 |
| 26 | **4z** | 2-anthracene | 72 | 91 | Vanc-59 |
| 27 | **4aa** | 4-NO_2_-Ph | 68 | 87 | Vanc-68 |
| 28 | **4ab** | 4-phenoxy phenyl | 74 | 96 | Vanc-72 |
| 29 | **4ac** | 4-C_5_H_11_-Ph | 76 | 94 | Vanc-74 |
| 30 | **4ad** | 4-C_6_H_13_-Ph | 75 | 95 | Vanc-75 |
| 31 | **4ae** | 4-Bn-Ph | 69 | 92 | Vanc-87 |
| 32 | **4af** | 4-benzophenone | 70 | 90 | Vanc-88 |
| 33 | **4ag** | 2-(9*H*-fluoren-9-one) | 68 | 89 | Vanc-89 |
| 34 | **4ah** | 9-(9*H*-fluorene) | 69 | 91 | Vanc-90 |

**Spectral data**

**tert-butyl 2-(4-hydroxyphenylamino)-2-oxoethylcarbamate (3a).**

White solid; ^1^H NMR (300 MHz, DMSO-d6): δ 9.63 (s, 1H), 9.17 (s, 1H), 7.35 (d, *J* = 8.9 Hz, 2H), 6.99 (t, *J* = 6.0 Hz, 1H), 6.69 (d, *J* = 8.9 Hz, 2H), 3.67 (d, *J* = 6.1 Hz, 2H), 1.39 (s, 9H); 13C NMR (75 MHz): δ 167.4, 155.8, 153.18, 130.55, 120.78, 115.01, 77.95, 43.57, 28.16. HRMS (EI): calcd. for C_13_H_18_N_2_O_4_, 266.1266, found: 266.12736.

**tert-butyl 2-(2-chloro-4-methylphenylamino)-2-oxoethylcarbamate (3b)**

White solid; 1H NMR (300 MHz, Chloroform-d) δ: 8.42 (s, 1H), 8.19 (d, *J* = 8.4 Hz, 1H), 7.17 (s, 1H), 7.05 (d, *J* = 8.4 Hz, 1H), 5.35 (s, 1H), 3.96 (d, *J* = 5.9 Hz, 2H), 2.29 (s, 3H), 1.48 (s, 9H); 13C NMR (75 MHz, CDCl_3_): δ 167.81, 156.08, 135.01, 131.56, 129.35, 128.29, 122.86, 121.43, 80.76, 45.59, 28.29, 20.65; HRMS (EI): calcd. for C_14_H_19_ClN_2_O_3_: 298.1084, found: 298.10964.

**tert-butyl 2-oxo-2-(phenylamino)ethylcarbamate (3c)**

White solid; ^1^H NMR (300 MHz, DMSO-d6): δ 9.91 (s, 1H), 7.58 (d, *J* = 8.6 Hz, 2H), 7.33 – 7.26 (m, 2H), 7.10 – 6.98 (m, 1H), 3.72 (d, *J* = 6.1 Hz, 2H), 1.40 (s, 9H); ^13^C NMR (75 MHz, DMSO): δ 168.16, 155.89, 138.92, 128.69, 123.10, 119.00, 78.00, 43.72, 28.18; HRMS (EI): calcd. for C_13_H_18_N_2_O_3:_ 250.1317, found: 250.13117

**tert-butyl 2-oxo-2-(p-tolylamino)ethylcarbamate (3d)**

White solid ^1^H NMR (300 MHz, DMSO-d6) δ 9.81 (s, 1H), 7.46 (d, *J* = 8.4 Hz, 2H), 7.10 (d, *J* = 8.3 Hz, 2H), 7.02 (t, *J* = 6.0, 6.0 Hz, 1H), 3.70 (d, *J* = 6.1 Hz, 2H), 2.24 (s, 3H), 1.39 (s, 7H); ^13^C NMR (75 MHz, DMSO): δ 167.90, 155.88, 136.41, 131.96, 129.06, 119.01, 77.98, 43.67, 28.17, 20.39; HRMS (EI): calcd. for C_14_H_20_N_2_O_3_: 264.1473, found: 264.14801.

**tert-butyl 2-(4-methoxyphenylamino)-2-oxoethylcarbamate (3e)**

White solid; ^1^H NMR (300 MHz, DMSO-d6): δ 9.76 (s, 1H), 7.49 (d, *J* = 9.0 Hz, 2H), 7.02 (t, *J* = 6.0, 6.0 Hz, 1H), 6.88 (d, *J* = 9.0 Hz, 2H), 3.72 (s, 3H), 3.69 (d, *J* = 6.2 Hz, 3H), 1.39 (s, 8H); ^13^C NMR (75 MHz, DMSO): δ 167.63, 155.86, 155.06, 132.06, 120.53, 113.78, 77.96, 55.08, 43.60, 28.16; HRMS (EI): calcd. for C_14_H_20_N_2_O_4:_ 280.1423, found: 280.14214.

**tert-butyl 2-(4-chlorophenylamino)-2-oxoethylcarbamate (3f)**

White solid, ^1^H NMR (300 MHz, DMSO-d6): δ 10.07 (s, 1H), 7.62 (d, *J* = 8.8 Hz, 2H), 7.36 (d, *J* = 8.8 Hz, 2H), 7.06 (d, *J* = 6.0 Hz, 1H), 3.74 (s, 2H), 1.38 (s, 9H); ^13^C NMR (75 MHz, DMSO) δ 168.37, 155.89, 137.87, 128.59, 126.65, 120.55, 78.04, 43.75, 28.16; HRMS (EI): calcd. for C_13_H_17_ClN_2_O_3_: 284.0927, found: 284.09157.

**tert-butyl 2-(3,4-dimethoxyphenylamino)-2-oxoethylcarbamate (3g)**

Pink solid; ^1^H NMR (300 MHz, DMSO-d6) δ 9.77 (s, 1H), 7.30 (s, 1H), 7.11 (d, *J* = 8.5 Hz, 1H), 7.03 (t, *J* = 6.0, 6.0 Hz, 1H), 6.91 (d, *J* = 8.7 Hz, 1H), 3.74 (d, *J* = 2.3 Hz, 6H), 3.70 (s, 2H), 1.42 (s, 9H); ^13^C NMR (75 MHz, CDCl_3_): δ 167.91, 156.64, 148.90, 145.87, 131.16, 112.16, 111.23, 104.97, 80.32, 56.04, 55.81, 45.07, 28.32; HRMS (EI): calcd. for C_15_H_22_N_2_O_5_: 310.1528, found: 310.15416.

**tert-butyl 2-(4-morpholinophenylamino)-2-oxoethylcarbamate (3h)**

White solid ^;1^H NMR (300 MHz, DMSO-d6) δ 9.70 (s, 1H), 7.44 (d, *J* = 9.0 Hz, 2H), 7.02 (s, 1H), 6.89 (d, *J* = 9.1 Hz, 2H), 3.71 (dt, *J* = 10.4, 5.3, 5.3 Hz, 6H), 3.06 – 3.00 (m, 4H), 1.39 (s, 9H); ^13^C NMR (75 MHz, DMSO) δ 167.49, 155.85, 147.09, 131.21, 120.11, 115.40, 77.95, 66.07, 48.88, 43.60, 28.17; HRMS (EI): calcd. for C_17_H_25_N_3_O_4_: 335.1845, found HRMS: 335.18349.

**tert-butyl 2-(mesitylamino)-2-oxoethylcarbamate (3i)**

White solid; ^1^H NMR (300 MHz, Chloroform-d) δ 7.74 (s, 1H), 6.87 (s, 2H), 5.51 (t, *J* = 5.7, 5.7 Hz, 1H), 3.94 (d, *J* = 5.9 Hz, 2H), 2.25 (s, 3H), 2.15 (s, 6H), 1.46 (s, 9H); ^13^C NMR (75 MHz, CDCl_3_) δ 168.50, 156.48, 137.08, 136.32, 135.13, 130.63, 129.57, 128.88, 80.32, 50.71, 44.77, 28.30, 20.92, 18.18; HRMS (EI): calcd. for C_16_H_24_N_2_O_3_: 292.1786, found: 292.1811.

**tert-butyl 2-(3-fluorophenylamino)-2-oxoethylcarbamate (3j)**

White solid, ^1^H NMR (300 MHz, Chloroform-d) δ 8.74 (s, 1H), 7.47 (d, *J* = 10.9 Hz, 1H), 7.30 – 7.11 (m, 2H), 6.79 (t, *J* = 8.2, 8.2 Hz, 1H), 5.59 (t, *J* = 5.6, 5.6 Hz, 1H), 3.95 (d, *J* = 5.8 Hz, 2H), 1.47 (s, 9H); ^13^C NMR (75 MHz, CDCl_3_) δ 168.15, 164.53, 161.29, 156.74, 139.15, 139.00, 129.98, 115.20, 115.16, 111.27, 110.98, 107.57, 107.22, 80.83, 45.41, 28.30. HRMS (EI): calcd. for C_13_H_17_FN_2_O_3_: 268.1223, found: 268.12366.

**tert-butyl 2-(3,5-dimethylphenylamino)-2-oxoethylcarbamate (3k)**

White solid; ^1^H NMR (300 MHz, Chloroform-d) δ 8.26 (s, 1H), 7.14 (s, 2H), 6.75 (s, 1H), 5.45 (s, 1H), 3.93 (d, *J* = 5.6 Hz, 3H), 2.27 (s, 6H), 1.47 (s, 9H); ^13^C NMR (75 MHz, CDCl_3_) δ 167.71, 156.41, 138.67, 137.28, 126.23, 117.77, 80.53, 45.46, 28.31, 21.33; HRMS (EI): calcd. for C_15_H_22_N_2_O_3_: 278.1630, found: 278.16088.

**tert-butyl 2-(3-methoxyphenylamino)-2-oxoethylcarbamate (3l)**

Off white solid; ^1^H NMR (300 MHz, Chloroform-d) δ 8.39 (s, 1H), 7.27-7.26 (m, 1H), 7.20 (t, *J* = 8.1, 8.1 Hz, 1H), 7.03 – 6.95 (m, 1H), 6.70 – 6.62 (m, 1H), 5.44 (s, 1H), 3.92 (d, *J* = 5.8 Hz, 2H), 3.79 (s, 3H), 1.47 (s, 9H); ^13^C NMR (75 MHz, CDCl_3_) δ 167.90, 160.10, 156.56, 138.69, 129.67, 112.12, 110.29, 105.70, 80.75, 55.29, 45.45, 28.30. HRMS (EI): calcd. for C_14_H_20_N_2_O_4_: 280.1423, found: 280.14273.

**tert-butyl 2-(4-ethylphenylamino)-2-oxoethylcarbamate (3m)**

White solid ; ^1^H NMR (300 MHz, DMSO-d6) δ 9.83 (s, 1H), 7.49 (d, *J* = 8.3 Hz, 2H), 7.13 (d, *J* = 8.2 Hz, 2H), 7.03 (t, *J* = 5.6, 5.6 Hz, 1H), 3.71 (s, 2H), 2.54 (q, *J* = 7.8, 7.6, 7.6 Hz, 2H), 1.4 (s, 1H), 1.15 (t, *J* = 7.6, 7.6 Hz, 3H); ^13^C NMR (75 MHz, DMSO) δ 167.90, 155.87, 138.43, 136.60, 127.85, 119.08, 77.96, 43.67, 28.16, 27.54, 15.65; HRMS (EI): calcd. for C_15_H_22_N_2_O_3_: 278.1630, found: 278.16409

**tert-butyl 2-(2,4-dichlorophenylamino)-2-oxoethylcarbamate (3n)**

Off White solid ; ^1^H NMR (300 MHz, DMSO-d6) δ 10.25 (s, 1H), 7.99 (d, *J* = 2.3 Hz, 1H), 7.55 (s, 1H), 7.49 (dd, *J* = 8.8, 2.3 Hz, 1H), 7.12 (t, *J* = 6.0, 6.0 Hz, 1H), 3.73 (d, *J* = 6.1 Hz, 2H), 1.40 (s, 9H); ^13^C NMR (75 MHz, DMSO) δ 168.78, 155.88, 138.97, 130.92, 130.64, 124.52, 120.18, 119.04, 78.09, 43.80, 39.46, 28.14; HRMS (EI): calcd. for C_13_H_16_Cl_2_N_2_O_3_: 318.0538, found: 318.05452.

**tert-butyl 2-(2,4-dimethylphenylamino)-2-oxoethylcarbamate (3o)**

Off White solid; ^1^H NMR (300 MHz, Chloroform-d): δ 8.01 (s, 1H), 7.63 (d, *J* = 8.6 Hz, 1H), 6.99 (d, *J* = 6.1 Hz, 3H), 5.44 (s, 1H), 3.92 (d, *J* = 6.0 Hz, 2H), 2.28 (s, 4H), 2.19 (s, 4H), 1.47 (s, 9H);^13^C NMR (75 MHz, CDCl_3_): δ 168.06, 156.48, 134.97, 132.67, 131.12, 129.34, 127.19, 123.01, 80.65, 45.58, 28.30, 20.86, 17.60; HRMS (EI): calcd. for C_15_H_22_N_2_O_3_: 278.1630, found: 278.16397.

**tert-butyl 2-(2,4-difluorophenylamino)-2-oxoethylcarbamate (3p**)

White solid; ^1^H NMR (300 MHz, DMSO-d6) δ 9.70 (s, 1H), 7.91 – 7.77 (m, 1H), 7.32 (ddd, *J* = 11.6, 9.0, 2.9 Hz, 1H), 7.12-7.03 (m, 2H), 3.77 (d, *J* = 6.1 Hz, 2H), 1.40 (s, 9H); ^13^C NMR (75 MHz, DMSO) δ 168.68, 155.88, 125.29, 125.12, 122.51, 122.36, 111.20, 110.86, 104.40, 104.08, 103.73, 78.08, 43.46, 28.13; HRMS (EI): calcd. for C_13_H_16_F_2_N_2_O_3_: 286.1129, found: 286.11153.

**tert-butyl 2-(3,5-dimethoxyphenylamino)-2-oxoethylcarbamate (3q)**

White solid; ^1^H NMR (300 MHz, DMSO-d6) δ 9.87 (s, 1H), 7.03 (t, *J* = 6.0, 6.0 Hz, 1H), 6.84 (d, *J* = 2.2 Hz, 2H), 6.21 (t, *J* = 2.2, 2.2 Hz, 1H), 3.71 (s, 6H), 3.68 (s, 2H), 1.40 (s, 9H); ^13^C NMR (75 MHz, DMSO) δ 168.24, 160.43, 155.86, 140.53, 97.34, 95.06, 78.00, 54.99, 43.75, 28.15; HRMS (EI): calcd. for C_15_H_22_N_2_O_5_: 310.1528, found: 310.14482.

**tert-butyl 2-(2-hydroxy-5-methylphenylamino)-2-oxoethylcarbamate (3r)**

Light brown solid; ^1^H NMR (300 MHz, DMSO-d6) δ 9.66 (s, 1H), 8.95 (s, 1H), 7.77 (s, 1H), 7.32 (t, *J* = 5.6, 5.6 Hz, 1H), 6.79 – 6.66 (m, 2H), 3.72 (d, *J* = 6.0 Hz, 2H), 2.18 (s, 3H), 1.41 (s, 9H); ^13^C NMR (75 MHz, DMSO): δ 168.05, 155.91, 144.38, 127.50, 125.80, 124.32, 120.95, 114.79, 78.35, 44.21, 28.12, 20.48; HRMS (EI): calcd. for C_14_H_20_N_2_O_4_: 280.1423, found: 280.14227.

**tert-butyl 2-(2-ethyl-5-methylphenylamino)-2-oxoethylcarbamate (3s)**

White solid; ^1^H NMR (300 MHz, DMSO-d6) δ 9.16 (s, 1H), 7.17 - 7.09 (m, 4H), 3.71 (d, *J* = 6.1 Hz, 2H), 2.49 (q, *J* = 7.6, 7.6, 7.5 Hz, 2H), 2.11 (s, 3H), 1.40 (s, 9H), 1.07 (t, *J* = 7.5, 7.5 Hz, 3H); ^13^C NMR (75 MHz, DMSO) δ 168.47, 155.85, 141.15, 135.70, 134.32, 127.50, 126.65, 125.84, 77.96, 43.46, 28.13, 24.18, 17.98, 14.49; HRMS (EI): calcd. for C_16_H_24_N_2_O_3_: 292.1786, found: 292.17953.

**tert-butyl 2-(4-butylphenylamino)-2-oxoethylcarbamate (3t)**

White solid; ^1^H NMR (300 MHz, DMSO-d6) δ 9.82 (s, 1H), 7.48 (d, *J* = 8.4 Hz, 2H), 7.10 (d, *J* = 8.5 Hz, 2H), 7.03 (t, *J* = 6.1, 6.1 Hz, 1H), 3.71 (d, *J* = 6.1 Hz, 2H), 1.61 – 1.42 (m, 2H), 1.40 (s, 9H), 1.36 – 1.22 (m, 2H), 0.88 (t, *J* = 7.3, 7.3 Hz, 3H); ^13^C NMR (75 MHz, DMSO) δ 167.89, 155.86, 136.97, 136.59, 128.35, 119.01, 77.94, 43.67, 34.20, 33.18, 28.14, 21.65, 13.72; HRMS (EI): calcd. for C_17_H_26_N_2_O_3_: 306.1943, found: 306.1933.

**tert-butyl 2-oxo-2-(4-(trifluoromethyl)phenylamino)ethylcarbamate (3u)**

White solid; ^1^H NMR (300 MHz, Methanol-d4) δ 10.33 (s, 1H), 7.82 (d, *J* = 8.5 Hz, 2H), 7.68 (d, *J* = 8.6 Hz, 2H), 7.13 (t, *J* = 6.0, 6.0 Hz, 1H), 3.79 (d, *J* = 6.0 Hz, 2H), 1.41 (s, 9H); ^13^C NMR (75 MHz, CH_3_OH+D_2_O) δ 171.60, 158.61, 145.16, 128.67, 126.48, 126.05, 125.63, 125.22, 121.56, 80.75, 46.54, 30.81; HRMS (EI): calcd. for C_14_H_17_F_3_N_2_O_3_: 318.1191, found: 318.1196.

**tert-butyl 2-(naphthalen-1-ylamino)-2-oxoethylcarbamate (3v)**

White solid; ^1^H NMR (300 MHz, DMSO-d6) δ 9.91 (s, 1H), 8.14 – 8.05 (m, 1H), 7.96-7.90 (m, 1H), 7.76 (d, *J* = 8.1 Hz, 1H), 7.70 (d, *J* = 7.3 Hz, 1H), 7.58 – 7.43 (m, 3H), 7.18 (t, *J* = 5.2, 5.2 Hz, 1H), 3.92 (d, *J* = 5.7 Hz, 2H), 1.43 (s, 9H); ^13^C NMR (75 MHz, DMSO) δ 169.05, 156.01, 133.67, 133.32, 128.07, 127.71, 125.98, 125.70, 125.53, 125.21, 122.69, 121.41, 78.10, 43.83, 28.17; HRMS (EI): calcd. for C_17_H_20_N_2_O_3_: 300.1473, found: 300.1482.

**tert-butyl 2-(9H-fluoren-2-ylamino)-2-oxoethylcarbamate (3w)**

Light pink solid; ^1^H NMR (300 MHz, DMSO-d6) δ 10.02 (s, 1H), 7.94 (s, 1H), 7.85 – 7.76 (m, 2H), 7.60 – 7.49 (m, 2H), 7.35 (t, *J* = 7.4, 7.4 Hz, 1H), 7.25 (t, *J* = 7.3, 7.3 Hz, 1H), 7.09 (t, *J* = 5.9, 5.9 Hz, 1H), 3.89 (s, 2H), 3.77 (d, *J* = 6.0 Hz, 2H), 1.41 (s, 9H); ^13^C NMR (75 MHz, DMSO) δ 168.11, 155.92, 143.70, 142.70, 140.94, 137.98, 136.24, 126.66, 126.01, 124.94, 120.07, 119.36, 117.81, 115.84, 78.00, 43.82, 36.46, 28.17; HRMS (EI): calcd. for C_20_H_22_N_2_O_3_: 338.1360, found: 338.1646.

**tert-butyl 2-oxo-2-(4-(phenylamino)phenylamino)ethylcarbamate (3x)**

Light brown solid; ^1^H NMR (300 MHz, DMSO-d6) δ 9.76 (s, 1H), 8.03 (s, 1H), 7.47 (d, *J* = 8.8 Hz, 2H), 7.19 (t, *J* = 7.8, 7.8 Hz, 2H), 7.02 (t, *J* = 9.1, 9.1 Hz, 5H), 6.76 (t, *J* = 7.3, 7.3 Hz, 1H), 3.71 (d, *J* = 6.0 Hz, 2H), 1.40 (s, 9H); ^13^C NMR (75 MHz, DMSO) δ 167.55, 155.87, 144.02, 138.70, 131.81, 129.07, 120.36, 118.91, 117.81, 115.70, 77.96, 43.64, 28.17; HRMS (EI): calcd. for C19H23N3O3: 341.1739; found: 341.1751.

**tert-butyl 2-(5-chloropyridin-2-ylamino)-2-oxoethylcarbamate (3y)**

White solid; ^1^H NMR (300 MHz, DMSO-d6): δ 10.62 (s, 1H), 8.36 (d, *J* = 2.6 Hz, 1H), 8.12 (d, *J* = 8.9 Hz, 1H), 7.92 – 7.87 (m, 1H), 7.09 (t, *J* = 5.9, 5.9 Hz, 1H), 3.83 (d, *J* = 6.0 Hz, 2H), 1.41 (s, 9H); ^13^C NMR (75 MHz, CDCl_3_) δ 168.71, 155.40, 149.94, 145.79, 137.39, 124.47, 113.88, 77.58, 43.24, 39.00, 27.65; HRMS (EI): calcd. for C_12_H_16_ClN_3_O_3_: 285.088, found: 285.0885

**tert-butyl 2-(anthracen-2-ylamino)-2-oxoethylcarbamate (3z)**

White solid; ^1^H NMR (300 MHz, DMSO-d6) δ 10.21 (s, 1H), 8.50 (s, 2H), 8.45 (s, 1H), 8.08 – 8.03 (m, 3H), 7.58 (dd, *J* = 9.1, 1.8 Hz, 1H), 7.51 – 7.44 (m, 2H), 7.15 (t, *J* = 6.1, 6.1 Hz, 1H), 3.83 (d, *J* = 6.1 Hz, 2H), 1.42 (s, 9H); ^13^C NMR (75 MHz, DMSO) δ 168.64, 155.94, 135.88, 131.66, 131.58, 130.35, 128.83, 128.44, 128.02, 127.64, 125.80, 125.58, 124.90, 120.84, 113.77, 78.04, 43.88, 28.19. HRMS (EI): calcd. for C_21_H_22_N_2_O_3_: 350.1630, found: 350.1636.

**tert-butyl 2-(4-nitrophenylamino)-2-oxoethylcarbamate (3aa)**

White solid; ^1^H NMR (300 MHz, DMSO-d6) δ 10.58 (s, 1H), 8.23 (d, *J* = 9.2 Hz, 2H), 7.83 (d, *J* = 9.2 Hz, 2H), 7.15 (t, *J* = 6.0, 6.0 Hz, 1H), 3.78 (d, *J* = 6.1 Hz, 2H), 1.40 (s, 9H); ^13^C NMR (151 MHz, DMSO) δ 169.70, 156.33, 145.49, 142.51, 125.41, 119.10, 78.55, 44.37, 28.56; HRMS (EI): calcd. for C_13_H_17_N_3_O_5_: 295.1168, found: 295.11652.

**tert-butyl 2-oxo-2-(4-phenoxyphenylamino)ethylcarbamate (3ab)**

White solid; ^1^H NMR (300 MHz, DMSO-d6) δ 9.96 (s, 1H), 7.63 (d, *J* = 8.9 Hz, 2H), 7.38 (t, *J* = 7.7, 7.7 Hz, 2H), 7.13 – 6.97 (m, 6H), 3.74 (d, *J* = 6.0 Hz, 2H), 1.42 (s, 9H); ^13^C NMR (75 MHz, DMSO) δ 168.01, 157.36, 155.90, 151.59, 134.88, 129.90, 122.90, 120.72, 119.45, 117.79, 78.02, 43.71, 28.18; HRMS (EI): calcd. for C_19_H_22_N_2_O_4_: 342.1579, found: 342.1567

**tert-butyl 2-oxo-2-(4-pentylphenylamino)ethylcarbamate (3ac)**

White solid; ^1^H NMR (300 MHz, DMSO-d6) δ 9.82 (s, 1H), 7.47 (d, *J* = 8.5 Hz, 2H), 7.11 (d, *J* = 8.5 Hz, 2H), 7.03 (t, *J* = 6.1, 6.1 Hz, 1H), 3.69 (d, *J* = 6.1 Hz, 2H), 1.58 – 1.48 (m, 2H), 1.39 (s, 9H), 1.35 – 1.20 (m, 6H), 0.88 – 0.83 (m, 3H); ^13^C NMR (101 MHz, DMSO) δ 168.41, 156.38, 137.56, 137.10, 128.89, 119.57, 78.49, 44.21, 34.99, 31.31, 28.69, 27.87, 22.43, 14.38; HRMS (EI): calcd. for C_18_H_28_N_2_O_3_: 320.2099, found: 320.2098.

**tert-butyl 2-(4-hexylphenylamino)-2-oxoethylcarbamate (3ad)**

White solid; ^1^H NMR (300 MHz, DMSO-d6) δ 9.82 (s, 1H), 7.48 (d, *J* = 8.3 Hz, 2H), 7.10 (d, *J* = 8.4 Hz, 2H), 7.02 (t, *J* = 6.0, 6.0 Hz, 1H), 3.71 (d, *J* = 6.0 Hz, 2H), 2.51 (t, *J* = 7.5, 7.5 Hz, 2H), 1.59 – 1.45 (m, 2H), 1.40 (s, 9H), 1.29-1.21 (m, 6H), 0.85 (t, *J* = 6.4, 6.4 Hz, 3H); ^13^C NMR (75 MHz, DMSO): δ 167.88, 155.85, 137.01, 136.58, 128.34, 119.03, 77.95, 43.69, 34.52, 31.07, 30.95, 28.22, 22.02, 13.88; HRMS (EI): calcd. for C_19_H_30_N_2_O_3_: 334.2256, found: 334.2260.

**tert-butyl 2-(3-benzylphenylamino)-2-oxoethylcarbamate (3ae)**

White solid; ^1^H NMR (300 MHz, DMSO-d6) δ 9.86 (s, 1H), 7.46 – 7.38 (m, 2H), 7.32 – 7.25 (m, 2H), 7.23-7.17 (m, 4H), 7.02 (t, *J* = 5.9, 5.9 Hz, 1H), 6.92 (d, *J* = 7.5 Hz, 1H), 3.90 (s, 2H), 3.68 (d, *J* = 6.1 Hz, 2H), 1.39 (s, 9H); ^13^C NMR (75 MHz, DMSO) δ 168.12, 155.86, 141.78, 141.05, 139.02, 128.73, 128.66, 128.39, 125.96, 123.61, 119.27, 116.80, 77.98, 43.68, 41.15, 28.18; HRMS (EI): calcd. for c_20_H_24_N_2_O_3_: 340.1786, found: ESI-MS: 340.7 [M+H]^+^.

**tert-butyl 2-(3-benzoylphenylamino)-2-oxoethylcarbamate (3af)**

White solid; ^1^H NMR (300 MHz, DMSO-d6) δ 10.36 (s, 1H), 7.77 (s, 4H), 7.71 (d, *J* = 7.6 Hz, 2H), 7.65 (d, *J* = 7.5 Hz, 1H), 7.56 (t, *J* = 7.6, 7.6 Hz, 2H), 7.13 (t, *J* = 6.0, 6.0 Hz, 1H), 3.78 (d, *J* = 6.0 Hz, 2H), 1.41 (s, 9H); ^13^C NMR (75 MHz, CDCl_3_) δ 199.76, 174.17, 161.18, 148.35, 142.77, 137.46, 136.44, 134.59, 133.71, 123.52, 83.34, 49.15, 33.43; HRMS (EI): calcd. for c_20_H_22_N_2_O_4_: 354.1579, found: 355.1

**tert-butyl 2-oxo-2-(9-oxo-9H-fluoren-2-ylamino)ethylcarbamate (3ag)**

Yellow solid; ^1^H NMR (300 MHz, DMSO-d6) δ 10.23 (s, 1H), 7.95 (s, 1H), 7.70 (d, *J* = 9.5 Hz, 3H), 7.62 – 7.55 (m, 2H), 7.32 (t, *J* = 7.3, 7.3 Hz, 1H), 7.13 (t, *J* = 6.0, 6.0 Hz, 1H), 3.76 (d, *J* = 6.0 Hz, 2H), 1.41 (s, 9H); ^13^C NMR (75 MHz, DMSO) δ 192.96, 168.62, 155.92, 144.10, 140.14, 138.32, 135.42, 134.03, 133.40, 128.61, 124.58, 123.90, 121.65, 120.58, 114.58, 78.09, 43.84, 28.17; HRMS (EI): calcd. for c_20_H_20_N_2_O_4_ : 352.1423, found: 353.2

**tert-butyl 2-(9H-fluoren-9-ylamino)-2-oxoethylcarbamate (3ah)**

White solid; ^1^H NMR (300 MHz, DMSO-d6) δ 8.43 (d, *J* = 8.3 Hz, 1H), 7.88 (d, *J* = 7.4 Hz, 2H), 7.53 – 7.41 (m, 4H), 7.34 (t, *J* = 7.4, 7.4 Hz, 2H), 7.02 (t, *J* = 5.9, 5.9 Hz, 1H), 6.04 (d, *J* = 8.3 Hz, 1H), 3.68 (d, *J* = 6.3 Hz, 2H), 1.42 (s, 9H);^13^C NMR (75 MHz, DMSO) δ 170.35, 155.78, 144.68, 140.00, 128.35, 127.52, 124.84, 120.10, 77.96, 54.07, 43.30, 28.18; ESI-MS: calcd. for c_20_H_22_N_2_O_3_ : 338.1630, found: 339.0

**Spectral data of Compound 4a-4ah**

**2-amino-N-(4-hydroxyphenyl)acetamide (4a)**

White solid;^1^H NMR (300 MHz, DMSO-d6) δ 10.58 (s, 1H), 9.40 (s, 1H), 8.31 (s, 3H), 7.41 (d, *J* = 8.9 Hz, 2H), 6.75 (d, *J* = 8.9 Hz, 2H), 3.73 (s, 2H); ^13^C NMR (75 MHz, DMSO) δ 163.81, 153.73, 129.88, 120.75, 115.20, 66.30; HRMS (EI): calcd. for C_8_H_10_N_2_O_2_: 166.0742, found:166.07329.

**2-amino-N-(2-chloro-4-methylphenyl)acetamide (4b)**

White solid; ^1^H NMR (300 MHz, DMSO-d6) δ 10.26 (s, 1H), 8.46 (s, 3H), 7.55 (d, *J* = 8.2 Hz, 1H), 7.35 (s, 1H), 7.17 (d, *J* = 9.5 Hz, 1H), 3.85 (s, 2H), 2.30 (s, 3H); ^13^C NMR (75 MHz, DMSO) δ 165.32, 136.68, 131.32, 129.72, 128.09, 126.54, 126.00, 40.58, 20.12; HRMS (EI): calcd. for C_9_H_11_ClN_2_O: 198.0559, found:198.05484.

**2-amino-N-phenylacetamide (4c)**

White solid; ^1^H NMR (300 MHz, DMSO-d6) δ 10.97 (s, 1H), 8.39 (s, 3H), 7.65 (d, *J* = 7.6 Hz, 2H), 7.32 (t, *J* = 7.9, 7.9 Hz, 2H), 7.07 (t, *J* = 7.4, 7.4 Hz, 1H), 3.81 (s, 2H); ^13^C NMR (75 MHz, DMSO) δ 164.67, 138.36, 128.81, 123.65, 119.05, 40.90; HRMS (EI): calcd. for C_8_H_10_N_2_O: 150.0793, found:150.07831.

**2-amino-N-p-tolylacetamide (4d)**

White solid; ^1^H NMR (300 MHz, DMSO-d6) δ 10.85 (s, 1H), 8.37 (s, 3H), 7.54 (d, *J* = 8.4 Hz, 2H), 7.14 (d, *J* = 8.2 Hz, 2H), 3.79 (s, 2H), 2.26 (s, 3H); ^13^C NMR (75 MHz, DMSO) δ 164.38, 135.87, 132.60, 129.17, 119.06, 40.85, 20.41; HRMS (EI): calcd. for C_9_H_12_N_2_O: 164.0949, found:164.09395.

**2-amino-N-(4-methoxyphenyl)acetamide (4e)**

White solid; ^1^H NMR (300 MHz, DMSO-d6) δ 10.80 (s, 1H), 8.36 (s, 3H), 7.57 (d, *J* = 9.1 Hz, 2H), 6.91 (d, *J* = 9.1 Hz, 2H), 3.77 (s, 2H), 3.73 (s, 3H); ^13^C NMR (75 MHz, DMSO) δ 164.08, 155.42, 131.48, 120.56, 113.93, 55.13, 40.74; HRMS (EI): calcd. for C_9_H_12_N_2_O_2_: 180.0898, found:180.08916.

**2-amino-N-(4-chlorophenyl)acetamide (4f)**

White solid; ^1^H NMR (300 MHz, DMSO-d6) δ 11.21 (s, 1H), 8.39 (s, 3H), 7.71 (d, *J* = 8.9 Hz, 2H), 7.40 (d, *J* = 8.9 Hz, 2H), 3.83 (s, 2H); ^13^C NMR (75 MHz, DMSO) δ 164.88, 137.33, 128.74, 127.24, 120.60, 40.94; HRMS (EI): calcd. for C_8_H_9_ClN_2_O: 184.0403, found: 184.04017

**2-amino-N-(3,4-dimethoxyphenyl)acetamide (4g)**

Off-white solid; ^1^H NMR (300 MHz, DMSO-d6) δ 10.82 (s, 1H), 8.38 (s, 3H), 7.35 (d, *J* = 2.4 Hz, 1H), 7.18 (dd, *J* = 8.7, 2.4 Hz, 1H), 6.92 (d, *J* = 8.8 Hz, 1H), 3.77 (s, 2H), 3.73 (s, 6H); ^13^C NMR (75 MHz, DMSO) δ 164.11, 148.47, 145.08, 131.88, 112.00, 111.07, 104.20, 55.64, 55.32, 40.77; HRMS (EI): calcd. for C_10_H_14_N_2_O_3_: 210.1004, found: 210.10017.

**2-amino-N-(4-morpholinophenyl)acetamide (4h)**

White solid; ^1^H NMR (300 MHz, DMSO-d6) δ 11.26 (s, 1H), 8.41 (s, 3H), 7.75 (d, *J* = 9.1 Hz, 2H), 7.68 (d, *J* = 8.8 Hz, 2H), 4.03 (s, 4H), 3.83 (d, *J* = 5.7 Hz, 2H), 3.47 (s, 4H); ^13^C NMR (75 MHz, DMSO) δ 164.80, 139.80, 136.97, 120.58, 119.86, 63.93, 52.77, 40.81;^13^C NMR (75 MHz, DMSO) δ 164.85, 140.19, 136.88, 120.49, 119.97, 64.07, 52.74, 40.90; HRMS (EI): calcd. for C_12_H_17_N_3_O_2_: 235.1320, found: 235.13279.

**2-amino-N-mesitylacetamide (4i)**

White solid; ^1^H NMR (300 MHz, DMSO-d6) δ 10.01 (s, 1H), 8.37 (s, 3H), 6.88 (s, 2H), 3.82 (s, 2H), 2.22 (s, 3H), 2.12 (s, 6H);^13^C NMR (75 MHz, DMSO) δ 164.61, 135.65, 134.73, 131.47, 128.27, 40.29, 20.39, 18.05; HRMS (EI): calcd. for C_11_H_16_N_2_O: 192.1262, found: 192.12642.

**2-amino-N-(3-fluorophenyl)acetamide (4j)**

White solid; ^1^H NMR (300 MHz, DMSO-d6) δ 11.28 (s, 1H), 8.40 (s, 3H), 7.69 – 7.57 (m, 1H), 7.47 – 7.31 (m, 3H), 7.00 – 6.86 (m, 1H), 3.84 (s, 2H); ^13^C NMR (75 MHz, DMSO) δ 165.12, 163.62, 160.42, 140.12, 139.97, 130.57, 130.44, 114.85, 110.28, 110.01, 106.05, 105.70, 40.99; HRMS (EI): calcd. for C_8_H_9_FN_2_O: 168.0698, found: 168.06975.

**2-amino-N-(3,5-dimethylphenyl)acetamide (4k)**

White solid; ^1^H NMR (300 MHz, Chloroform-d) δ 8.26 (s, 1H), 7.14 (s, 2H), 6.75 (s, 1H), 5.45 (s, 1H), 3.93 (d, *J* = 5.6 Hz, 3H), 2.27 (s, 6H), 1.47 (s, 9H); ^13^C NMR (75 MHz, CDCl_3_) δ 167.71, 156.41, 138.67, 137.28, 126.23, 117.77, 80.53, 45.46, 28.31, 21.33; HRMS (EI): calcd. for C_15_H_22_N_2_O_3_: 278.1630, found: 278.16088.

**2-amino-N-(3-methoxyphenyl)acetamide (4l)**

White solid; ^1^H NMR (300 MHz, DMSO-d6) δ 10.94 (s, 1H), 8.38 (s, 3H), 7.39 – 7.31 (m, 1H), 7.28 – 7.14 (m, 2H), 6.68 (ddd, *J* = 7.9, 2.4, 1.3 Hz, 1H), 3.80 (s, 2H), 3.73 (s, 3H); ^13^C NMR (75 MHz, CDCl_3_) δ 169.97, 164.71, 144.74, 134.90, 116.66, 114.16, 110.35, 60.21, 46.19; HRMS (EI): calcd. for C_9_H_12_N_2_O_2_: 180.0898, found: 180.08792.

**2-amino-N-(4-ethylphenyl)acetamide (4m)**

White solid; ^1^H NMR (300 MHz, DMSO-d6) δ 10.88 (s, 1H), 8.39 (s, 3H), 7.56 (d, *J* = 8.5 Hz, 2H), 7.17 (d, *J* = 8.5 Hz, 2H), 3.80 (s, 2H), 2.55 (q, *J* = 7.6, 7.6, 7.6 Hz, 2H), 1.15 (t, *J* = 7.6, 7.6 Hz, 3H); ^13^C NMR (75 MHz, DMSO) δ 164.40, 139.05, 136.04, 127.98, 119.14, 40.83, 27.55, 15.60; HRMS (EI): calcd. for C_10_H_14_N_2_O: 178.1106, found:178.11113.

**2-amino-N-(2,4-dichlorophenyl)acetamide (4n)**

White solid; ^1^H NMR (300 MHz, DMSO-d6) δ 11.49 (s, 1H), 8.39 (s, 3H), 8.02 (d, *J* = 1.3 Hz, 1H), 7.65-7.58 (m, 2H), 3.86 (s, 2H); ^13^C NMR (75 MHz, DMSO) δ 165.30, 138.43, 131.01, 130.80, 125.16, 120.20, 119.11, 41.02; HRMS (EI): calcd. for C_8_H_8_Cl_2_N_2_O: 218.0013, found: 218.00237.

**2-amino-N-(2,4-dimethylphenyl)acetamide (4o)**

White solid; ^1^H NMR (300 MHz, DMSO-d6) δ 10.08 (s, 1H), 8.40 (s, 3H), 7.28 (d, *J* = 8.0 Hz, 1H), 7.09 – 6.92 (m, 2H), 3.83 (s, 2H), 2.25 (s, 3H), 2.20 (s, 3H); ^13^C NMR (75 MHz, DMSO) δ 164.84, 134.60, 132.78, 131.60, 130.89, 126.47, 124.69, 40.53, 20.44, 17.89; HRMS (EI): calcd. for C_10_H_14_N_2_O: 178.1106, found: 178.11027.

**2-amino-N-(2,4-difluorophenyl)acetamide (4p)**

White solid; ^1^H NMR (300 MHz, DMSO-d6) δ 10.58 (s, 1H), 8.45 (s, 3H), 7.82 (td, *J* = 9.0, 9.0, 6.2 Hz, 1H), 7.37 (ddd, *J* = 11.6, 9.0, 2.8 Hz, 1H), 7.19 – 7.05 (m, 2H), 3.86 (s, 2H); ^13^C NMR (75 MHz, DMSO) δ 165.34, 160.45, 160.30, 157.22, 157.06, 155.73, 155.56, 152.43, 152.26, 125.53, 125.36, 121.74, 121.63, 111.41, 111.12, 104.67, 104.35, 104.31, 103.99, 40.62; HRMS (EI): calcd. for C_8_H_8_F_2_N_2_O: 186.0604, found:186.05964.

**2-amino-N-(3,5-dimethoxyphenyl)acetamide (4q)**

White solid; ^1^H NMR (300 MHz, DMSO-d6) δ 10.90 (s, 1H), 8.37 (s, 3H), 6.90 (d, t, *J* = 2.2 Hz, 2H), 6.27 (t, *J* = 2.2, 2.2 Hz, 1H), 3.79 (s, 2H), 3.72 (s, 6H); ^13^C NMR (75 MHz, DMSO) δ 164.76, 160.47, 139.91, 97.58, 95.38, 55.06, 40.95; HRMS (EI): calcd. for C_10_H_14_N_2_O_3_: 210.1004, found: 210.10013.

**2-amino-N-(2-hydroxy-5-methylphenyl)acetamide (4r)**

Light brown solid; ^1^H NMR (300 MHz, DMSO-d6) δ 9.66 (s, 1H), 8.95 (s, 1H), 7.77 (s, 1H), 7.32 (t, *J* = 5.6, 5.6 Hz, 1H), 6.79 – 6.66 (m, 2H), 3.72 (d, *J* = 6.0 Hz, 2H), 2.18 (s, 3H), 1.41 (s, 9H); ^13^C NMR (75 MHz, DMSO) δ 168.05, 155.91, 144.38, 127.50, 125.80, 124.32, 120.95, 114.79, 78.35, 44.21, 28.12, 20.48; HRMS (EI): calcd. for C_14_H_20_N_2_O_4_: 280.1423, found: 280.14227.

**2-amino-N-(2-ethyl-6-methylphenyl)acetamide (4s)**

White solid; ^1^H NMR (300 MHz, DMSO-d6) δ 10.15 (s, 1H), 8.39 (s, 3H), 7.21 – 7.05 (m, 3H), 3.85 (s, 3H), 2.58 (q, *J* = 7.6, 7.6, 7.5 Hz, 2H), 2.15 (s, 3H), 1.10 (t, *J* = 7.5, 7.5 Hz, 3H); ^13^C NMR (75 MHz, DMSO) δ 164.92, 140.96, 135.49, 133.47, 127.67, 127.01, 125.98, 40.28, 24.20, 18.16, 14.62; HRMS (EI): calcd. for C_11_H_16_N_2_O: 192.1262, found:192.12568.

**2-amino-N-(4-butylphenyl)acetamide (4t)**

White solid; ^1^H NMR (300 MHz, DMSO-d6) δ 10.82 (s, 1H), 8.36 (s, 3H), 7.54 (d, *J* = 8.5 Hz, 2H), 7.15 (d, *J* = 8.5 Hz, 2H), 3.79 (s, 2H), 2.56 – 2.49 (m, 2H), 1.52 (p, *J* = 7.7, 7.7, 7.4, 7.4 Hz, 2H), 1.27 (dt, *J* = 14.3, 7.3, 7.3 Hz, 2H), 0.89 (t, *J* = 7.3, 7.3 Hz, 3H); ^13^C NMR (75 MHz, DMSO) δ 164.40, 137.63, 136.02, 128.52, 119.07, 40.83, 34.19, 33.13, 21.65, 13.74; HRMS (EI): calcd. for C_12_H_18_N_2_O: 206.1419, found: 206.1438.

**2-amino-N-(4-(trifluoromethyl)phenyl)acetamide (4u)**

White solid; ^1^H NMR (300 MHz, DMSO-d6) δ 11.44 (s, 1H), 8.40 (s, 3H), 7.89 (d, *J* = 8.5 Hz, 2H), 7.72 (d, *J* = 8.6 Hz, 2H), 3.88 (s, 2H); ^13^C NMR (75 MHz, DMSO) δ 165.49, 141.91, 126.24, 123.86, 123.44, 122.44, 119.00, 41.08; HRMS (EI): calcd. for C_9_H_9_F_3_N_2_O: 218.0667, found: 218.0658.

**2-amino-N-(naphthalen-1-yl)acetamide (4v)**

White solid; ^1^H NMR (300 MHz, DMSO-d6) δ 10.82 (s, 1H), 8.49 (s, 3H), 8.27-8.21 (m, 1H), 8.01 – 7.90 (m, 1H), 7.81 (d, *J* = 8.2 Hz, 1H), 7.72 (d, *J* = 7.1 Hz, 1H), 7.62 – 7.47 (m, 3H), 4.04 (s, 2H); ^13^C NMR (75 MHz, DMSO) δ 165.74, 133.67, 132.65, 128.06, 127.54, 126.13, 125.88, 125.67, 125.52, 122.99, 121.42, 40.85; HRMS (EI): calcd. for C_12_H_12_N_2_O: 200.0949, found: 200.0970.

**2-amino-N-(9H-fluoren-2-yl)acetamide (4w)**

White solid; ^1^H NMR (300 MHz, DMSO-d6) δ 11.10 (s, 1H), 8.42 (s, 3H), 7.99 – 7.92 (m, 1H), 7.84 (t, *J* = 8.0, 8.0 Hz, 2H), 7.68 (dd, *J* = 8.3, 1.8 Hz, 1H), 7.55 (d, *J* = 7.3 Hz, 1H), 7.36 (td, *J* = 7.5, 7.5, 1.3 Hz, 1H), 7.27 (td, *J* = 7.4, 7.4, 1.1 Hz, 1H), 3.91 (s, 2H), 3.87 (s, 2H); ^13^C NMR (75 MHz, DMSO) δ 164.59, 143.76, 142.75, 140.80, 137.41, 136.75, 126.70, 126.18, 124.97, 120.21, 119.49, 117.87, 115.85, 66.30, 40.98; HRMS (EI): calcd. for C_15_H_14_N_2_O: 238.1106, found: 222.0939 (-NH_2_).

**2-amino-N-(4-(phenylamino)phenyl)acetamide (4x)**

Light-brown solid; ^1^H NMR (300 MHz, DMSO-d6) δ 10.61 (s, 1H), 8.29 (s, 3H), 7.50 (d, *J* = 8.6 Hz, 2H), 7.20 (t, *J* = 7.6, 7.6 Hz, 2H), 7.05 (dd, *J* = 11.9, 8.7 Hz, 4H), 6.79 (d, *J* = 7.9 Hz, 1H), 3.76 (q, *J* = 5.5, 5.5, 5.3 Hz, 2H); ^13^C NMR (75 MHz, DMSO) δ 163.92, 143.75, 139.23, 131.06, 129.07, 120.36, 119.15, 117.60, 115.95, 40.73; HRMS (EI): calcd. for C_14_H_15_N_3_O: 241.1215, found: 241.1211.

**2-amino-N-(5-chloropyridin-2-yl)acetamide (4y)**

White solid; ^1^H NMR (300 MHz, DMSO-d6) δ 11.22 (s, 1H), 8.54 (s, 3H), 8.41 (d, *J* = 3.1 Hz, 1H), 8.08 (d, *J* = 8.7 Hz, 1H), 7.97 (dd, *J* = 8.9, 2.6 Hz, 1H), 3.86 (d, *J* = 5.6 Hz, 2H); ^13^C NMR (75 MHz, DMSO) δ 165.85, 149.78, 146.48, 138.19, 125.57, 114.57, 40.96; HRMS (EI): calcd. for C_7_H_8_ClN_3_O: 185.0355, found: 185.0369.

**2-amino-N-(anthracen-2-yl)acetamide (4z)**

White solid; ^1^H NMR (300 MHz, DMSO-d6) δ 11.01 (s, 1H), 8.51 (d, *J* = 9.5 Hz, 3H), 8.31 (s, 3H), 8.14 – 8.02 (m, 3H), 7.63 (dd, *J* = 9.1, 1.9 Hz, 1H), 7.65-7.45 (m, 2H), 3.90 (s, 2H);^13^C NMR (75 MHz, DMSO) δ 165.15, 135.26, 131.70, 131.37, 130.53, 129.14, 128.57, 128.05, 127.65, 125.94, 125.74, 125.16, 125.11, 120.49, 114.21, 41.13; HRMS (EI): calcd. for C_16_H_14_N_2_O: 250.1106, found: 250.1211.

**2-amino-N-(4-nitrophenyl)acetamide (4aa)**

Light-yellow solid; ^1^H NMR (300 MHz, DMSO-d6) δ 11.59 (s, 1H), 8.34 (s, 3H), 8.27 (d, *J* = 9.3 Hz, 2H), 7.91 (d, *J* = 9.3 Hz, 2H), 3.90 (s, 2H); ^13^C NMR (75 MHz, DMSO) δ 165.90, 144.38, 142.56, 125.10, 118.90, 41.28; HRMS (EI): calcd. for C_8_H_9_N_3_O_3_: 195.0643, found:195.06217.

**2-amino-N-(4-phenoxyphenyl)acetamide (4ab)**

White solid; ^1^H NMR (300 MHz, DMSO-d6) δ 10.83 (s, 1H), 8.28 (s, 3H), 7.65 (d, *J* = 9.0 Hz, 2H), 7.37 (tt, *J* = 7.5, 7.5, 2.2, 2.2 Hz, 2H), 7.11 (tt, *J* = 7.1, 7.1, 1.0, 1.0 Hz, 1H), 7.06 – 6.94 (m, 4H), 3.79 (s, 2H); ^13^C NMR (75 MHz, DMSO) δ 164.52, 157.17, 152.05, 134.23, 129.96, 123.04, 120.79, 119.51, 117.89, 40.87; HRMS (EI): calcd. for C_14_H_14_N_2_O_2_: 242.1055, found: 242.10486.

**2-amino-N-(4-pentylphenyl)acetamide (4ac)**

Off-white solid; ^1^H NMR (300 MHz, DMSO-d6) δ 10.79 (s, 1H), 8.34 (s, 3H), 7.54 (d, *J* = 8.5 Hz, 2H), 7.15 (d, *J* = 8.5 Hz, 2H), 3.78 (d, *J* = 5.5 Hz, 2H), 2.52-2.48 (m, 2H), 1.54 (p, *J* = 7.5, 7.5, 7.3, 7.3 Hz, 2H), 1.34-1.23 (m, 4H), 0.85 (t, *J* = 6.9, 6.9 Hz, 3H); ^13^C NMR (101 MHz, DMSO) δ 164.89, 138.20, 136.50, 129.02, 119.61, 41.36, 34.98, 31.29, 31.10, 22.39, 14.37; HRMS (EI): calcd. for C_13_H_20_N_2_O: 220.1575, found: 220.1562.

**2-amino-N-(4-hexylphenyl)acetamide (4ad)**

White solid; ^1^H NMR (300 MHz, DMSO-d6) δ 10.73 (s, 1H), 8.30 (s, 3H), 7.52 (d, *J* = 8.5 Hz, 2H), 7.14 (d, *J* = 8.5 Hz, 2H), 3.77 (s, 2H), 2.50 (dt, *J* = 3.6, 1.8, 1.8 Hz, 2H), 1.53 (p, *J* = 7.6, 7.6, 7.2, 7.2 Hz, 2H), 1.30 – 1.21 (m, 6H), 0.88 – 0.81 (m, 3H);^13^C NMR (75 MHz, DMSO) δ 164.90, 138.21, 136.47, 129.05, 119.58, 41.35, 35.02, 31.56, 31.42, 28.72, 22.52, 14.41; HRMS (EI): calcd. for C_14_H_22_N_2_O: 234.1732, found: 234.1730.

**2-amino-N-(3-benzylphenyl)acetamide (4ae)**

White solid; ^1^H NMR (300 MHz, DMSO-d6) δ 10.87 (s, 1H), 8.40 (s, 3H), 7.51 (d, *J* = 5.6 Hz, 2H), 7.33 – 7.19 (m, 6H), 6.99 (d, *J* = 7.5 Hz, 1H), 3.93 (s, 2H), 3.79 (s, 2H); ^13^C NMR (75 MHz, DMSO) δ 164.63, 141.96, 140.94, 138.48, 128.87, 128.67, 128.42, 126.00, 124.16, 119.35, 116.88, 41.12, 40.89.

**2-amino-N-(3-benzoylphenyl)acetamide (4af)**

White solid; ^1^H NMR (300 MHz, DMSO-d6) δ 11.45 (s, 1H), 8.45 (s, 3H), 7.87 (d, *J* = 8.7 Hz, 2H), 7.77 (d, *J* = 8.7 Hz, 2H), 7.72 (d, *J* = 6.9 Hz, 2H), 7.66 (d, *J* = 7.4 Hz, 1H), 7.56 (t, *J* = 7.4, 7.4 Hz, 2H), 3.92 (s, 2H); ^13^C NMR (75 MHz, DMSO) δ 194.51, 165.47, 142.43, 137.35, 132.30, 131.80, 131.16, 131.08, 129.37, 128.47, 128.08, 118.45, 41.17; HRMS (EI): calcd. for C_15_H_14_N_2_O_2_: 254.1055, found: 255.10

**2-amino-N-(9-oxo-9H-fluoren-2-yl)acetamide (4ag)**

Yellow solid; ^1^H NMR (300 MHz, DMSO-d6) δ 11.42 (s, 1H), 8.41 (s, 3H), 7.99 (s, 1H), 7.84 – 7.67 (m, 3H), 7.58 (t, *J* = 7.3, 7.3 Hz, 2H), 7.32 (t, *J* = 7.4, 7.4 Hz, 1H), 3.88 (s, 2H); ^13^C NMR (75 MHz, DMSO) δ 192.84, 165.12, 143.96, 139.58, 138.83, 135.44, 134.05, 133.37, 128.73, 124.71, 123.92, 121.75, 120.69, 114.55, 41.05; ESI-MS: calcd. for C_15_H_12_N_2_O_2_: 252.0898, found: 253.0 [M+H]^+^

**2-amino-N-(9H-fluoren-9-yl)acetamide (4ah)**

White solid; ^1^H NMR (300 MHz, DMSO-d6) δ 8.93 (d, *J* = 8.1 Hz, 1H), 8.10 (s, 2H), 7.88 (d, *J* = 7.5 Hz, 2H), 7.53 (d, *J* = 7.1 Hz, 2H), 7.46 (t, *J* = 7.4, 7.4 Hz, 2H), 7.36 (td, *J* = 7.5, 7.5, 1.2 Hz, 2H), 6.04 (d, *J* = 8.4 Hz, 1H), 3.64 (s, 2H); ^13^C NMR (75 MHz, DMSO) δ 166.89, 143.98, 140.07, 128.68, 127.66, 124.93, 120.29, 62.56, 54.20; HRMS (EI): calcd. for C_15_H_14_N_2_O.HCl: 273.6, found: 274.0 [M+H]^+^.

- 1. **Synthetic route when R is a terminal or internal alkyne (Sonogashira reaction)**

**(synthesis of vancomycin analogue substituents 11n, Vanc-37, 38, 68-70)**

**Scheme 4.**

**Reagents and conditions:** i) alkyne, piperidine, Pd(PPh_3_)Cl_2_, 85 ^o^C, 30 min; ii) when R’=TMS; water-dioxane (9:1), 140 ^o^C, 4h; when R’= cyclic and acyclic alkane; 4N HCl in 1,4-dioxane, DCM, 0 ^o^C to rt, 1h.

**Procedure**

**Synthesis of 5.**

In a 25 mL round-bottom ﬂask was placed: **3a** (R-I; 1.04 mmol), terminal alkyne (when R’=H; TMS acetylene was used) (2.08 mmol), piperidine (10 equiv.), PdCl_2_(PPh_3_)_2_ (0.04 mmol, 4 mol%) and a magnetic stir bar. The reaction mixture was stirred at 85 ^o^C for 30-40 min. After completion of the reaction, the reaction mass was poured in water (25 mL) and extracted with ethyl acetate (3 x 30 mL). The combined organic layer was washed with 1N HCl solution, brine solution (25 mL) and dried over Na_2_SO_4_. After filtration, the solvent was evaporated under reduced pressure, and the crude product was subjected to silica gel column chromatography (2-4% methanol in DCM) to afford the corresponding compound **5**.^3^

**Synthesis of 6**:

**When R’=H**;

Compound **3** (0.5 mmol) was dissolved in 0.5 mL dioxane and 4.5 mL H_2_O in a screw-capped vial and heated at 140 ^o^C for 3h. After completion of reaction, the reaction mass was cooled to rt, hence product **4** fell out as a white- to yellow-colored solid (depending upon the substitution). It was filtered through a Buchner funnel, washed with diethyl ether, and dried under vacuum. Along with Boc, the TMS group was also removed by this procedure.^2^

**When R’= cyclic or acyclic alkane;**

Boc deprotection of compound **5** was carried out by following the procedure (step ii’) used for the synthesis of **4**.

**Table S11**

| Entry | Product | R’ | Yield %  **5** | Yield %  **6** | Vancomycin analogue |
| --- | --- | --- | --- | --- | --- |
| 1 | **6a** | H | 69 | 95 | Vanc-N |
| 2 | **6b** | C_4_H_9_ | 70 | 69 | Vanc-37 |
| 3 | **6c** | Ph | 69 | 65 | Vanc-38 |
| 4 | **6d** | C_3_H_7_ | 65 | 62 | Vanc-68 |
| 5 | **6e** | cyclopentane | 75 | 66 | Vanc-69 |
| 6 | **6f** | cyclopropane | 77 | 69 | Vanc-70 |

**Spectral data of compound 5a-f**

**tert-Butyl 2-(4-ethynylphenylamino)-2-oxoethylcarbamate (5a).**

White solid; ^1^H NMR (300 MHz, CDCl_3_): δ 8.30 (br s, 1H), 7.46 (q, *J* = 8.4 Hz, 4H), 5.24 (br s, 1H), 3.92 (d, *J* = 5.8Hz, 2H), 3.04 (s, 1H), 1.48 (s, 9H); ^13^C NMR (75 MHz, MeOD): δ 170.61, 158.59, 140.09, 133.62, 120.80, 119.18, 84.21, 80.84, 78.05, 45.08, 28.72; HRMS (EI): calcd. for C_15_H_18_N_2_O_3_: 274.1300, found: 274.1300.

**tert-butyl 2-(4-(hex-1-ynyl)phenylamino)-2-oxoethylcarbamate (5b)**

Light-brown solid; ^1^H NMR (300 MHz, DMSO-d6) δ 10.03 (s, 1H), 7.57 (d, *J* = 8.6 Hz, 2H), 7.31 (d, *J* = 8.6 Hz, 2H), 7.05 (t, *J* = 5.9, 5.9 Hz, 1H), 3.73 (d, *J* = 6.0 Hz, 2H), 2.40 (t, *J* = 6.8, 6.8 Hz, 2H), 1.57 – 1.42 (m, 4H), 1.40 (s, 9H), 0.91 (t, *J* = 7.2, 7.2 Hz, 3H); ^13^C NMR (75 MHz, DMSO) δ 168.34, 155.93, 138.40, 131.74, 117.76, 89.61, 80.39, 78.14, 43.71, 30.28, 28.12, 21.38, 18.25, 13.42; HRMS (EI): calcd. for C_19_H_26_N_2_O_3_: 330.1943, found: 330.19444.

**tert-butyl 2-oxo-2-(4-(phenylethynyl)phenylamino)ethylcarbamate (5c)**

Light-brown solid; ^1^H NMR (300 MHz, DMSO-d6) δ 10.15 (s, 1H), 7.66 (d, *J* = 8.7 Hz, 2H), 7.56 – 7.48 (m, 4H), 7.46 – 7.37 (m, 3H), 7.10 (t, *J* = 6.1, 6.1 Hz, 1H), 3.75 (d, *J* = 6.1 Hz, 2H), 1.40 (s, 9H); ^13^C NMR (75 MHz, DMSO) δ 168.48, 155.91, 139.35, 132.06, 131.18, 128.69, 128.51, 122.47, 118.91, 116.48, 89.43, 88.48, 78.05, 43.82, 28.16; HRMS (EI): calcd. for C_21_H_22_N_2_O_3_: 350.1630, found: 350.16273

**tert-butyl 2-oxo-2-(4-(pent-1-ynyl)phenylamino)ethylcarbamate (5d)**

Yellow solid; ^1^H NMR (300 MHz, DMSO-d6) δ 10.03 (s, 1H), 7.56 (d, *J* = 8.4 Hz, 2H), 7.32 (d, *J* = 8.3 Hz, 2H), 7.05 (t, *J* = 6.1, 6.1 Hz, 1H), 3.72 (d, *J* = 6.0 Hz, 2H), 2.37 (t, *J* = 7.0, 7.0 Hz, 2H), 1.55 (h, *J* = 7.2, 7.2, 7.2, 7.0, 7.0 Hz, 2H), 1.39 (s, 9H), 0.99 (t, *J* = 7.3, 7.3 Hz, 3H); ^13^C NMR (75 MHz, DMSO) δ 168.32, 155.90, 138.53, 131.78, 118.85, 117.71, 89.41, 80.61, 78.04, 43.80, 28.18, 21.71, 20.58, 13.34; HRMS (EI): calcd. for C_17_H_22_N_2_O_3_: 316.1786, found: 316.177

**tert-butyl 2-(4-(cyclopentylethynyl)phenylamino)-2-oxoethylcarbamate (5e)**

Compound was used directly for next step without further purification. HRMS (EI): calcd. for C_20_H_26_N_2_O_3_: 342.1943, found: 342.1935

**tert-butyl 2-(4-(cyclopropylethynyl)phenylamino)-2-oxoethylcarbamate (5f)**

Light-yellow solid, ^1^H NMR (300 MHz, DMSO-d6) δ 10.86 (s, 1H), 8.23 (s, 3H), 7.59 (d, *J* = 8.7 Hz, 2H), 7.34 (d, *J* = 8.7 Hz, 2H), 3.79 (s, 2H), 1.52 (tt, *J* = 8.2, 8.2, 5.0, 5.0 Hz, 1H), 0.94 – 0.81 (m, 2H), 0.76 – 0.61 (m, 2H); ^13^C NMR (75 MHz, DMSO) δ 168.55, 156.14, 138.73, 132.10, 119.06, 117.83, 92.94, 78.28, 75.76, 44.05, 28.41, 8.54, 0.00; HRMS (EI): calcd. for C_18_H_22_N_2_O_3_: 314.1630, found: 314.1635.

**Spectral data of compounds 6a-f**

**2-Amino-*N*-(4-ethynylphenyl)acetamide (6a)**.

Viscous liquid; ^1^H NMR (300 MHz, DMSO-d6) δ 7.66 (d, *J* = 8.6 Hz, 2H), 7.41 (d, *J* = 8.6 Hz, 2H), 4.07 (d, *J* = 0.9 Hz, 1H), 3.28 (s, 2H); ^13^C NMR (75 MHz, DMSO) δ 172.35, 139.35, 132.33, 118.82, 115.96, 83.55, 79.71, 45.56; HRMS (EI): calcd. for C_10_H_10_N_2_O: 174.0793, found: 174.0798.

**2-amino-N-(4-(hex-1-ynyl)phenyl)acetamide (6b)**

Light-brown solid; ^1^H NMR (300 MHz, DMSO-d6) δ 10.96 (s, 1H), 8.29 (s, 3H), 7.61 (d, *J* = 8.7 Hz, 2H), 7.35 (d, *J* = 8.7 Hz, 2H), 3.80 (s, 2H), 2.40 (t, *J* = 6.8, 6.8 Hz, 2H), 1.60 – 1.33 (m, 4H), 0.91 (t, *J* = 7.2, 7.2 Hz, 3H); ^13^C NMR (75 MHz, DMSO) δ 164.47, 137.29, 131.90, 119.28, 118.66, 90.38, 80.09, 40.82, 30.08, 21.27, 18.12, 13.33; HRMS (EI): calcd. for C_14_H_18_N_2_O: 230.1419, found: 230.14169.

**2-amino-N-(4-(phenylethynyl)phenyl)acetamide (6c)**

White solid; ^1^H NMR (300 MHz, DMSO-d6) δ 11.10 (s, 1H), 8.32 (s, 3H), 7.72 (d, *J* = 8.7 Hz, 2H), 7.59 – 7.49 (m, 4H), 7.48 – 7.37 (m, 3H), 3.84 (s, 2H); ^13^C NMR (75 MHz, DMSO) δ 165.03, 138.81, 132.16, 131.22, 128.71, 128.60, 122.39, 119.03, 117.10, 89.32, 88.72, 41.07; HRMS (EI): calcd. for C_16_H_14_N_2_O: 250.1106, found: 250.11064.

**2-amino-N-(4-(pent-1-ynyl)phenyl)acetamide (6d)**

Yellow solid; ^1^H NMR (300 MHz, DMSO-d6) δ 11.01 (s, 1H), 8.31 (s, 3H), 7.62 (d, *J* = 8.6 Hz, 2H), 7.36 (d, *J* = 8.6 Hz, 2H), 3.81 (s, 2H), 2.38 (t, *J* = 7.0, 7.0 Hz, 2H), 1.55 (h, *J* = 7.2, 7.2, 7.1, 7.1, 7.1 Hz, 2H), 0.99 (t, *J* = 7.3, 7.3 Hz, 3H); ^13^C NMR (75 MHz, DMSO) δ 164.83, 137.89, 131.89, 118.94, 118.34, 89.74, 80.47, 41.01, 21.67, 20.55, 13.32.

**2-amino-N-(4-(cyclopentylethynyl)phenyl)acetamide (6e)**

Brown solid; ^1^H NMR (300 MHz, DMSO-d6) δ 10.97 (s, 1H), 8.29 (s, 3H), 7.61 (dd, *J* = 8.8, 2.6 Hz, 2H), 7.34 (dd, *J* = 8.8, 1.0 Hz, 2H), 3.80 (q, *J* = 5.2, 5.2, 5.0 Hz, 2H), 2.88 – 2.79 (m, 1H), 2.01 – 1.37 (m, 6H), 0.90 – 0.82 (m, 1H), 0.74 – 0.67 (m, 1H); ^13^C NMR (75 MHz, DMSO) δ 165.33, 138.32, 132.49, 132.37, 119.38, 99.98, 93.53, 41.51, 33.95, 30.49, 25.05; HRMS (EI): calcd. for C_15_H_18_N_2_O: 242.1419, found: 242.14304

**2-amino-N-(4-(cyclopropylethynyl)phenyl)acetamide (6f)**

White solid; ^1^H NMR (300 MHz, DMSO-d6) δ 11.01 (s, 1H), 8.31 (s, 3H), 7.61 (d, *J* = 8.8 Hz, 2H), 7.33 (d, *J* = 8.7 Hz, 2H), 3.81 (s, 2H), 1.56-1.47 (m, 1H), 0.90 – 0.83 (m, 2H), 0.73 – 0.67 (m, 2H) ^13^C NMR (75 MHz, DMSO) δ 165.33, 138.36, 132.48, 119.38, 118.69, 93.53, 75.87, 41.50, 8.80, 0.23; HRMS (EI): calcd. for C_13_H_14_N_2_O: 214.1106, found: 214.11138

**Synthetic route when R is alkene (Heck reaction)**

**Synthetic route for vancomycin analogue substituents derived from aryl amines (vanc-R, vanc 58)**

**Scheme 5.**

**Reagents and conditions:** i) Substituted styrene, Pd(OAc)_2_, tetrabutylammonium bromide, potassium acetate, 80 ^o^C, 4-5h; ii) 4N HCl in 1,4-dioxane, 2-3h; iii) If R= Br; trimethylsilylacetylene, piperidine, Pd(PPh_3_)Cl_2_, 85 ^o^C, 30 min; iv) TBAF, THF, rt, 30 min.

**Procedure:**

1. **Synthesis of 7a-b.**

In a round-bottom, 2-necked ﬂask, **3a** (500 mg, 1.33 mmol) was added to a mixture of tetrabutylammonium bromide (322 mg, 1.99 mmol), potassium acetate (210 mg, 2.14 mmol), palladium acetate (15 mg, 0.066 mmol) and substituted styrene (1.46 mmol) and stirred in DMF (12 mL) at rt under argon. The reaction mixture was heated up to 80 ^o^C for 4-5h and then cooled to room temperature. After completion of the reaction as monitored by TLC (6:4, heptane/ethyl acetate) and mass analysis, it was cooled to rt, poured in water (50 mL) and extracted with ethylacetate (3x 30 mL). The combined organic layer was washed with brine solution (50 mL) and dried over Na_2_SO_4_. After filtration, the solvent was evaporated under reduced pressure and the crude product was subjected to silica gel column chromatography (30-40% ethylacetate in heptane) to afford the compound **7** with 55-65% yield.^4^

1. **Synthesis of 8a-b**

This compound **8** was synthesized by following the procedure used for the synthesis of **4**.

1. **Synthesis of 9.**

In a 25 mL round-bottom ﬂask was placed: **8** (R=Br; 1.04 mmol), TMS acetylene (2.08 mmol), piperidine (1.10 mL, 10 equiv.), PdCl_2_(PPh_3_)_2_ (0.04 mmol, 4 mol%) and a magnetic stir bar. The reaction mixture was stirred at 85 ^o^C for 30-40 min. After completion of the reaction, the reaction mass was poured in water (25 mL) and extracted with ethyl acetate (3 x 30 mL). The organic layer was concentrated under reduced pressure yielding a brown-colored solid product **9.** This was dissolved in THF (2mL) and cooled in an ice bath at 0 ^o^C. A solution of TBAF (tetrabutylammonium flouride) in THF (1.2 equiv, 0.15 µM solution) was added dropwise to it and the mixture was allowed to stir at room temperature for 30 min. After completion of the reaction, THF was removed *in* *vacuo* and the solid residue obtained was dissolved in a water/ACN mixture, and purified by preparative HPLC using a gradient of 35% ACN in water/HCOOH. The solvent was removed under reduced pressure, and dried to obtain pure product in 50% yield.

**Spectral data**

**tert-Butyl 2-(4-iodophenylamino)-2-oxoethylcarbamate (3a’).**

White solid, yield 68%; ^1^H NMR (300 MHz, DMSO-d6): δ 10.03 (s, 1H), 7.64 (d, *J* = 8.8 Hz, 2H), 7.43 (d, *J* = 8.8 Hz, 2H), 7.06 (t, *J* = 6.0, 6.0 Hz, 1H), 3.71 (d, *J* = 6.1 Hz, 2H), 1.39 (s, 9H); ^13^C NMR (75 MHz, DMSO): δ 168.39, 155.89, 138.75, 137.34, 121.22, 86.51, 78.04, 43.80, 28.17; HRMS (EI): calcd. for C_13_H_17_N_2_O_3_: 376.0283, found: 376.0276.

**(E)-tert-butyl 2-(4-(4-aminostyryl)phenylamino)-2-oxoethylcarbamate (7a)**

Brown solid, used directly for next reaction

**(*E*)-tert-Butyl 2-(4-(4-bromostyryl)phenylamino)-2-oxoethylcarbamate (7b).**

Yellow solid, yield 62%; ^1^H NMR (300 MHz, DMSO-d_6_): δ 10.02 (s, 1H), 7.64-7.51 (m, 8H), 7.18 (dd, *J* = 15.8 Hz,15.8 Hz, 2H), 7.06 (t, 5.9 Hz, 1H), 3.73 (d, *J* = 6.0 Hz, 2H), 1.40 (s, 9H); ^13^C NMR (75 MHz, DMSO-d_6_): δ 168.21, 155.90, 138.67, 136.55, 131.68, 131.53, 128.93, 128.19, 127.09, 125.67, 120.12, 119.10, 78.03, 43.78, 28.18.; HRMS (EI): calcd. for C_21_H_23_N_2_O_3_Br: 430.0892, found: 430.0888.

**(E)-2-amino-N-(4-(4-aminostyryl)phenyl)acetamide (8a)**

Brown solid; ^1^H NMR (300 MHz, DMSO-d6) δ 11.05 (s, 1H), 8.37 (s, 1H), 7.96 – 7.58 (m, 5H), 7.40 (d, J= 8.56 Hz, 2H), 7.23 – 7.11 (m, 2H), 6.86-6.51 (m, 1H), 3.83 (d, 5.21, 1H); ^13^C NMR (75 MHz, DMSO) δ 164.70, 138.01, 136.61, 132.26, 131.26, 128.75, 127.28, 127.16, 126.12, 123.31, 119.18, 40.98; HRMS (EI): calcd. for C_16_H_17_N_3_O: 267.1371, found: 267.13667.

**(*E*)-2-Amino-*N*-(4-(4-bromostyryl)phenyl)acetamide (8b).**

White solid, yield 85%; ^1^H NMR (300 MHz, DMSO-d_6_): ^1^H NMR (300 MHz, DMSO-d6) δ 10.98 (s, 1H), 8.33 (s, 3H), 7.69 (s, 1H), 7.64 (d, *J* = 16.5 Hz, 2H), 7.58 (s, 1H), 7.55 (s, 4H), 7.21 (q, *J* = 16.5, 16.5, 16.4 Hz, 2H), 3.83 (s, 2H).; ^13^C NMR (75 MHz, DMSO-d_6_): 164.7, 138.0, 136.4, 132.3, 131.5, 128.8, 128.2, 127.2, 126.0, 120.2, 119.1, 40.9.; MS (CI): calcd. for C_21_H_23_N_2_O_3_Br: 330.036, found: 331.0.

**(*E*)-2-Amino-*N*-(4-(4-ethynylstyryl)phenyl)acetamide (9).**

Yellow solid, yield 40%; ^1^H NMR (300 MHz, DMSO-d_6_): δ 8.31 (s, 1H), 7.66 (d, 8.0 Hz, 2H), 7.64-7.57 (m, 4H), 7.46 (d, 8.0 Hz, 2H), 7.22 (dd, *J* = 15.5 Hz, 2H), 4.23 (s, 1H), 3.45 (s, 2H); ^13^C NMR (75 MHz, DMSO) δ 164.58, 137.89, 137.59, 132.02, 131.67, 130.63, 129.08, 128.71, 128.31, 127.25, 126.40, 120.26, 118.79, 83.44, 81.22, 43.80^.^; HRMS (EI): calcd. for C_18_H_16_N_2_O: 276.1263, found: 276.1262.

- 1. **Synthesis of vancomycin analogue substituents for Vanc- 60-62 (sulphonamide synthesis).**

**Scheme 6.**

**Reagents and condition**: i) pyridine, rt, 5-6h; ii) 4N HCl in 1,4-dioxane, DCM, 0 ^o^C to rt, 2h iii) ethylchloroformate, N-Boc-glycine, THF, TEA*,* overnight, 0 ^o^C to rt.

**Procedure**.

1. **Synthesis of 12.**

N-Boc -p-phenylenediamine **10** (1.0 mmol) and benzenesulphonyl chloride **11** (1.12 mmol) were dissolved in pyridine (1 mL) and stirred in a screw-capped vial for 5-6h at rt. The progress of the reaction was monitored by TLC (EA/heptane; 1:1). After completion of the reaction, 1N HCl was added till pH 6, and extracted with ethyl acetate (3 x 25 mL), and washed with brine. The solvent was removed under reduced pressure. This crude product was subjected to silica gel column chromatography (30-50% ethylacetate in heptane) to afford the compound **12** with 70-84% yield.

1. **Synthesis of 13.**

**13** was synthesised by following the procedure used for the synthesis of **4**. Yield 90-95%.

1. **Synthesis of 14.**

**14** was synthesised by following the procedure used for the synthesis of **3**. Yield: 65-70%.

1. **Synthesis of 15.**

**9d’** was synthesised by following the procedure used for the synthesis of **4**. Yield 85-90%.

**Table S12**

| Entry | Product | R | Yield %  **12** | Yield %  **13** | Yield %  **14** | Yield %  **15** | Vancomycin analogue |
| --- | --- | --- | --- | --- | --- | --- | --- |
| 1 | **15a** | H | 84 | 85 | 65 | 70 | Vanc-60 |
| 2 | **15b** | 2,4-DiCl | 59 | 82 | 51 | 69 | Vanc-61 |

**Spectral data of 12a-b**

**tert-butyl 4-(phenylsulfonamido)phenylcarbamate (12a)**

White solid; ^1^H NMR (300 MHz, DMSO-d6) δ 9.99 (s, 1H), 9.27 (s, 1H), 7.75 – 7.67 (m, 2H), 7.64 – 7.48 (m, 3H), 7.29 (d, *J* = 8.9 Hz, 2H), 6.95 (d, *J* = 8.9 Hz, 2H), 1.44 (s, 9H); ^13^C NMR (75 MHz, DMSO) δ 152.66, 139.39, 136.34, 132.66, 131.48, 129.06, 126.62, 121.90, 118.67, 78.93, 28.04; HRMS (EI): calcd. for C_17_H_20_N_2_O_4_S: 348.1143, found: 348.1121.

**tert-butyl 4-(2,4-dichlorophenylsulfonamido)phenylcarbamate (12b)**

White solid; ^1^H NMR (300 MHz, DMSO-d6): δ 10.41 (s, 1H), 9.27 (s, 1H), 7.92 (d, *J* = 8.6 Hz, 1H), 7.85 (d, *J* = 2.1 Hz, 1H), 7.57 (dd, *J* = 8.6, 2.1 Hz, 1H), 7.29 (d, *J* = 8.9 Hz, 2H), 6.97 (d, *J* = 8.9 Hz, 2H), 1.44 (s, 9H); ^13^C NMR (75 MHz, DMSO) δ 152.64, 138.39, 136.49, 135.49, 132.88, 131.92, 131.19, 130.44, 127.80, 121.50, 118.78, 78.97, 28.03; HRMS (EI): calcd. for C_17_H_18_Cl_2_N_2_O_4_S: 416.0364, found: 416.03943.

**Spectral data of 13a-b**

**N-(4-aminophenyl)benzenesulfonamide (13a)**

White solid; ^1^H NMR (300 MHz, DMSO-d6) δ 10.43 (s, 1H), 7.84 – 7.71 (m, 3H), 7.63-7.52 (m, 4H), 7.24-7.13 (m, 5H); ^13^C NMR (75 MHz, DMSO) δ 139.19, 132.96, 130.49, 129.23, 126.63, 126.47, 122.81, 121.27; HRMS (EI): calcd. for C_12_H_12_N_2_O_2_S: 248.0619, found: 248.0618.

**N-(4-aminophenyl)-2,4-dichlorobenzenesulfonamide (13b)**

Ligh-pink solid; ^1^H NMR (300 MHz, DMSO-d6) δ 10.89 (s, 1H), 9.76 (s, 2H), 8.02 (d, *J* = 8.6 Hz, 1H), 7.86 (d, *J* = 2.1 Hz, 1H), 7.62 (dd, *J* = 8.6, 2.1 Hz, 1H), 7.18 (q, *J* = 9.1, 9.1, 9.1 Hz, 4H); ^13^C NMR (75 MHz, DMSO) δ 138.73, 135.60, 135.21, 132.95, 131.90, 131.40, 128.95, 127.97, 123.56, 120.59; HRMS (EI): calcd. for C_12_H_10_Cl_2_N_2_O_2_S: 315.9840, found: 315.98076.

**Spectral data of 14a-b**

**tert-butyl 2-oxo-2-(4-(phenylsulfonamido)phenylamino)ethylcarbamate (14a)**

White solid; ^1^H NMR (300 MHz, DMSO-d6) δ 9.85 (s, 1H), 7.71-7.68 (m, 2H), 7.66 – 7.46 (m, 4H), 7.42 (d, *J* = 8.9 Hz, 2H), 7.04-6.98(m, 3H), 3.65 (d, *J* = 6.1 Hz, 2H), 1.38 (s, 9H); ^13^C NMR (75 MHz, DMSO) δ 167.97, 155.84, 139.34, 135.67, 132.73, 132.46, 129.11, 126.59, 121.63, 119.69, 77.97, 43.62, 28.15; HRMS (EI): calcd. for C_19_H_23_N_3_O_5_S: 405.1358, found: 405.1330.

**tert-butyl 2-(4-(2,4-dichlorophenylsulfonamido)phenylamino)-2-oxoethylcarbamate (14b)**

White solid; ^1^H NMR (300 MHz, DMSO-d6) δ 10.53 (s, 1H), 9.86 (s, 1H), 7.94 (d, *J* = 8.6 Hz, 1H), 7.85 (d, *J* = 2.1 Hz, 1H), 7.57 (dd, *J* = 8.5, 2.1 Hz, 1H), 7.43 (d, *J* = 8.9 Hz, 2H), 7.02 (d, *J* = 8.9 Hz, 3H), 3.65 (d, *J* = 6.1 Hz, 2H), 1.38 (d, *J* = 1.4 Hz, 9H); ^13^C NMR (75 MHz, DMSO) δ 168.01, 155.83, 138.45, 135.77, 135.48, 132.85, 131.91, 131.47, 131.23, 127.84, 121.19, 119.82, 77.98, 43.62, 28.14; HRMS (EI): calcd. for C_19_H_21_Cl_2_N_3_O_5_S: 473.0579, found: 473.06256.

**Spectral data of 15a-b**

**2-amino-N-(4-(phenylsulfonamido)phenyl)acetamide (15a)**

White solid; ^1^H NMR (300 MHz, DMSO-d6) δ 10.56 (s, 1H), 10.20 (s, 1H), 8.16 (s, 3H), 7.75 – 7.67 (m, 2H), 7.65 – 7.36 (m, 3H), 7.45 (d, *J* = 8.9 Hz, 2H), 7.05 (d, *J* = 8.9 Hz, 2H), 3.72 (d, *J* = 5.4 Hz, 2H); ^13^C NMR (75 MHz, DMSO) δ 164.50, 139.36, 134.91, 133.14, 132.79, 129.15, 126.62, 121.57, 119.80, 40.85; HRMS (EI): calcd. for C_14_H_15_N_3_O_3_S: 305.0834, found: 305.0843.

**2-amino-N-(4-(2,4-dichlorophenylsulfonamido)phenyl)acetamide (15b)**

White solid; ^1^H NMR (300 MHz, DMSO-d6) δ 10.81 (s, 1H), 10.62 (s, 1H), 8.29 (s, 3H), 7.97 (d, *J* = 8.6 Hz, 1H), 7.85 (d, *J* = 2.1 Hz, 1H), 7.59 (dd, *J* = 8.6, 2.1 Hz, 1H), 7.50 (d, *J* = 8.9 Hz, 2H), 7.07 (d, *J* = 8.9 Hz, 2H), 3.74 (s, 2H); ^13^C NMR (75 MHz, DMSO) δ 164.50, 138.48, 135.49, 135.05, 132.84, 132.11, 131.93, 131.26, 127.87, 121.08, 119.90, 40.81; HRMS (EI): calcd. for C_14_H_13_Cl_2_N_3_O_3_S: 373.0054, found: 373.0024.

- 1. **Synthesis of vancomycin analogue substituents for Vanc- 72, 76-78, 91-92 (click chemistry).**

**Scheme 7.**

**Reagents and condition**: i) NaN_3_, CuI, L-proline, NaOH, DMSO, 60 ^o^C, 7-8h; i’) NaN_3_, H_2_O, 90 ^o^C, 10h; ii) CuI, DIPEA, THF, 60 ^o^C, 20h; iii) 4N HCl in 1,4-dioxane, DCM, 0 ^o^C to rt, 2h.

**Procedure**.

1. **Synthesis of substituted/unsubstituted phenyl azide 16a-c (when R-X = aryl iodide)**

Substituted or unsubstituted phenyl iodide (4.2 mmol), NaN_3_ (8.4 mmol), CuI (10 mol%), L-proline (20 mol%) and NaOH (20 mol%) were suspended in DMSO (5mL) in a screw-capped vial and stirred at 60 ^o^C for 7-8h. Progress of the reaction was monitored by TLC and mass spectrometry. After completion of the reaction, water was added to the reaction mixture, which was extracted with ethyl acetate (3 x 30 mL), washed with brine and concentrated under reduced pressure. The crude product was subjected to silica gel column chromatography (5-10% ethylacetate in heptane) to afford the compound with 40-50% yield.^5^

**ii’. Synthesis of aliphatic azide 16d-f (when R-X = alkyl bromide)**

Alkyl bromide (0.5 mmol) and NaN_3_ (1 mmol) were suspended in water (5 mL) in a screw-capped vial and stirred at 90 ^o^C for 10h. After completion of the reaction, the reaction mass was allowed to settle for a while in a separating funnel, and the organic layer was separated from aqueous layer. The organic layer was dried over Na_2_SO_4_ and used directly for the next step.

**Synthesis of 17a-f.**

THF 1.5 mL and DIPEA (1.0 equiv) were added to a mixture of **3** (0.36 mmol, 1.0 equiv.), azide **16** (2.0 equiv.), and CuI (10 mol%), and the reaction mixture was stirred at 60 ^o^C for 20h. The progress of the reaction was monitored by TLC (EA/heptane; 6:4). After completion of the reaction, the reaction mixture was concentrated and subjected to silica gel column chromatography (60-70% ethyl acetate in heptane) to afford the compound with 70-75% yield.

**Synthesis of 18a-f.**

**18a-f** was synthesized by following the procedure used for the synthesis of **4**. Yield: 95%

**Table S13**

| Entry | Product | R | Yield %  **16** | Yield %  **17** | Yield %  **18** | Vancomycin analogue |
| --- | --- | --- | --- | --- | --- | --- |
| 1 | **18a** | 4-OCH_3_-Ph | 61 | 45 | 86 | Vanc-72 |
| 2 | **18b** | 4-CH_3_-Ph | 57 | 51 | 85 | Vanc-76 |
| 3 | **18c** | Ph | 59 | 50 | 89 | Vanc-77 |
| 4 | **18d** | Acetyl protected sugar | 75 | 49 | 88 | Vanc-78 |
| 5 | **18f** | C_5_H_11_ | 84 | 46 | 89 | Vanc-91 |
| 6 | **18f** | C_6_H_13_ | 85 | 49 | 88 | Vanc-92 |

**Spectral data of compound 17a-f**

**tert-butyl 2-(4-(1-(4-methoxyphenyl)-1H-1,2,3-triazol-4-yl)phenylamino)-2-oxoethylcarbamate (17a)**

White solid; ^1^H NMR (300 MHz, DMSO-d6) δ 10.08 (s, 1H), 9.10 (s, 1H), 7.86 (t, *J* = 8.8, 8.8 Hz, 4H), 7.72 (d, *J* = 8.7 Hz, 2H), 7.17 (d, *J* = 9.1 Hz, 2H), 7.09 (t, *J* = 6.0, 6.0 Hz, 1H), 3.84 (s, 3H), 3.76 (d, *J* = 6.0 Hz, 2H), 1.41 (s, 9H); ^13^C NMR (75 MHz, DMSO) δ 168.79, 159.71, 156.41, 147.42, 139.33, 130.56, 126.27, 125.72, 122.06, 119.81, 119.42, 115.36, 78.53, 56.00, 44.27, 28.66; HRMS (EI): calcd. for C_22_H_25_N_5_O_4_: 423.1906, found: 322.11325.

**tert-butyl 2-oxo-2-(4-(1-p-tolyl-1H-1,2,3-triazol-4-yl)phenylamino)ethylcarbamate (17b)**

Light-brown solid, used directly for next step without further purifications.

**tert-butyl 2-oxo-2-(4-(1-phenyl-1H-1,2,3-triazol-4-yl)phenylamino)ethylcarbamate (17c)**

Light-brown solid, used directly for next step; HRMS (EI): calcd. for C_21_H_23_N_5_O_3_: 393.1800, found: 393.1780.

**(2R,3R,4R,5R,6R)-2-(acetoxymethyl)-6-(5-(4-(2-(tert-butoxycarbonylamino)acetamido)phenyl)-1H-1,2,3-triazol-1-yl)tetrahydro-2H-pyran-3,4,5-triyl triacetate (17d)**

White solid; ^1^H NMR (300 MHz, DMSO-d6) δ 10.06 (s, 1H), 8.86 (s, 1H), 7.78 (d, *J* = 8.8 Hz, 2H), 7.69 (d, *J* = 8.8 Hz, 2H), 7.07 (t, *J* = 6.0, 6.0 Hz, 1H), 6.40 (d, *J* = 8.8 Hz, 1H), 5.73 – 5.55 (m, 2H), 5.18 (t, *J* = 9.6, 9.6 Hz, 1H), 4.42 (ddd, *J* = 10.1, 5.2, 2.6 Hz, 1H), 4.21 – 4.06 (m, 2H), 3.74 (d, *J* = 6.1 Hz, 2H), 2.04 (s, 3H), 2.01 (s, 3H), 1.98 (s, 3H), 1.82 (s, 3H), 1.40 (s, 9H).

^13^C NMR (75 MHz, DMSO) δ 170.49, 170.02, 169.84, 169.05, 163.62, 156.39, 147.21, 139.44, 126.25, 125.33, 120.17, 119.79, 84.38, 78.52, 73.67, 72.55, 70.71, 68.00, 61.87, 44.28, 28.67, 20.97, 20.85, 20.72, 20.39; ESI-MS: calcd. for C_29_H_37_N_5_O_12_: 647.2438, found: 670.4 [M+Na]^+^.

**tert-butyl 2-oxo-2-(4-(1-pentyl-1H-1,2,3-triazol-4-yl)phenylamino)ethylcarbamate (17e)**

White solid; ^1^H NMR (300 MHz, DMSO-d6) δ 10.02 (s, 1H), 8.50 (s, 1H), 7.77 (d, *J* = 8.6 Hz, 2H), 7.66 (d, *J* = 8.6 Hz, 2H), 7.07 (t, *J* = 6.0, 6.0 Hz, 1H), 4.37 (t, *J* = 7.0, 7.0 Hz, 2H), 3.73 (d, *J* = 6.1 Hz, 2H), 1.86 (p, *J* = 7.2, 7.2, 7.2, 7.2 Hz, 2H), 1.40 (s, 9H), 1.33 – 1.21 (m, 4H), 0.86 (t, *J* = 7.0, 7.0 Hz, 3H); ^13^C NMR (75 MHz, DMSO) δ 168.23, 155.91, 146.08, 138.51, 125.77, 125.54, 120.60, 119.27, 78.02, 49.43, 43.77, 29.30, 28.01, 21.52, 13.77; ESI-MS calcd. for C_20_H_29_N_5_O_3_: 387.2270, found:ESI-MS: [775.0 2M+H]^+^.

**tert-butyl 2-(4-(1-hexyl-1H-1,2,3-triazol-4-yl)phenylamino)-2-oxoethylcarbamate (17f)**

White solid; ^1^H NMR (300 MHz, DMSO-d6) δ 10.03 (s, 1H), 8.50 (s, 1H), 7.77 (d, *J* = 8.1 Hz, 2H), 7.66 (d, *J* = 8.5 Hz, 2H), 7.08 (t, *J* = 6.4, 6.4 Hz, 1H), 4.37 (t, *J* = 7.1, 7.1 Hz, 2H), 3.73 (d, *J* = 5.9 Hz, 2H), 1.90 – 1.79 (m, 2H), 1.40 (s, 9H), 1.31 – 1.21 (m, 6H), 0.85 (t, *J* = 6.5, 6.5 Hz, 3H); ^13^C NMR (75 MHz, DMSO) δ 168.23, 155.91, 146.08, 138.51, 125.77, 125.54, 120.59, 119.27, 78.02, 49.44, 43.77, 30.57, 29.56, 28.18, 25.49, 21.89, 13.82; ESI-MS calcd. for C_21_H_31_N_5_O_3_: 401.2426, found: ESI-MS: 825.1 [2M+Na]^+^.

**Spectral data of compound 18a-f**

**2-amino-N-(4-(1-(4-methoxyphenyl)-1H-1,2,3-triazol-4-yl)phenyl)acetamide (18a)**

White solid; ^1^H NMR (300 MHz, DMSO-d6) δ 10.88 (s, 1H), 9.16 (s, 1H), 8.26 (s, 3H), 7.92 (d, *J* = 8.7 Hz, 2H), 7.86 (d, *J* = 9.1 Hz, 2H), 7.75 (d, *J* = 8.8 Hz, 2H), 7.18 (d, *J* = 9.1 Hz, 2H), 3.85 (s, 3H), 3.83 (d, *J* = 6.5 Hz, 2H); ^13^C NMR (75 MHz, DMSO) δ 164.81, 159.25, 152.89, 146.76, 142.32, 138.12, 125.93, 121.61, 119.42, 119.15, 114.89, 55.56, 42.01; HRMS (EI): calcd. for C_17_H_17_N_5_O_2_: 323.1382, found: 323.1389.

**2-amino-N-(4-(1-p-tolyl-1H-1,2,3-triazol-4-yl)phenyl)acetamide (18b)**

Light-brown solid; ^1^H NMR (300 MHz, DMSO-d6) δ 10.91 (s, 1H), 9.22 (s, 1H), 8.28 (s, 3H), 7.92 (d, *J* = 8.7 Hz, 2H), 7.83 (d, *J* = 8.4 Hz, 2H), 7.76 (d, *J* = 8.8 Hz, 2H), 7.44 (d, *J* = 8.1 Hz, 2H), 3.83 (d, *J* = 5.7 Hz, 2H), 2.40 (s, 3H); ^13^C NMR (75 MHz, DMSO) δ 165.31, 147.36, 138.78, 138.68, 134.86, 133.85, 130.73, 126.45, 120.31, 119.92, 119.53, 41.53, 21.07; HRMS (EI): calcd. for C_17_H_17_N_5_O: 307.1433, found: 307.14267.

**2-amino-N-(4-(1-phenyl-1H-1,2,3-triazol-4-yl)phenyl)acetamide (18c)**

Light-brown solid; ^1^H NMR (300 MHz, DMSO-d6) δ 11.05 (d, *J* = 1.9 Hz, 1H), 9.30 (s, 1H), 8.36 (s, 3H), 7.95 (t, *J* = 8.0, 8.0 Hz, 4H), 7.79 (d, *J* = 8.6 Hz, 2H), 7.64 (t, *J* = 7.7, 7.7 Hz, 2H), 7.52 (t, *J* = 7.4, 7.4 Hz, 1H), 3.85 (d, *J*= 5.67 Hz, 2H); ^13^C NMR (75 MHz, DMSO) δ 164.82, 147.00, 138.30, 136.61, 129.89, 128.64, 125.96, 125.69, 119.93, 119.44, 119.17, 41.02; HRMS (EI): calcd. for C_16_H_15_N_5_O: 293.1276, found: 293.12796.

**(2R,3R,4R,5R,6R)-2-(acetoxymethyl)-6-(5-(4-(2-aminoacetamido)phenyl)-1H-1,2,3-triazol-1-yl)tetrahydro-2H-pyran-3,4,5-triyl triacetate (18d)**

White solid; ^1^H NMR (300 MHz, DMSO-d6) δ 10.86 (s, 1H), 8.88 (s, 1H), 8.25 (s, 3H), 7.83 (d, *J* = 8.8 Hz, 2H), 7.73 (d, *J* = 8.8 Hz, 2H), 6.40 (d, *J* = 8.8 Hz, 1H), 5.73 – 5.52 (m, 2H), 5.18 (t, *J* = 9.6, 9.6 Hz, 1H), 4.47 – 4.37 (m, 1H), 4.22 – 4.06 (m, 2H), 3.82 (d, *J* = 5.8 Hz, 2H), 2.05 (s, 3H), 2.01 (s, 3H), 1.98 (s, 3H), 1.82 (s, 3H); ^13^C NMR (75 MHz, DMSO) δ 170.00, 169.53, 169.37, 168.58, 164.84, 146.59, 138.32, 125.88, 125.47, 119.87, 119.43, 83.89, 73.19, 72.04, 70.22, 67.52, 61.74, 41.03, 20.50, 20.37, 20.24, 19.91; HRMS (EI): calcd. for C_24_H_29_N_5_O_10_: 547.1914, found: ESI-MS 548.0 [M+H]^+^.

**2-amino-N-(4-(1-pentyl-1H-1,2,3-triazol-4-yl)phenyl)acetamide (18e)**

White solid; ^1^H NMR (300 MHz, DMSO-d6) δ 11.10 (s, 1H), 8.57 (s, 1H), 8.41 (s, 3H), 7.83 (d, *J* = 8.6 Hz, 2H), 7.75 (d, *J* = 8.6 Hz, 2H), 4.39 (t, *J* = 7.0, 7.0 Hz, 2H), 3.85 (d, *J* = 5.6 Hz, 2H), 1.86 (p, *J* = 7.1, 7.1, 6.9, 6.9 Hz, 2H), 1.37 – 1.19 (m, 4H), 0.86 (t, *J* = 7.0, 7.0 Hz, 3H); ^13^C NMR (75 MHz, DMSO) δ 164.77, 145.95, 137.98, 126.31, 125.68, 120.85, 119.39, 49.49, 41.00, 29.32, 28.02, 21.54, 13.79; HRMS (EI): calcd. for C_15_H_21_N_5_O: 287.1746, found: 304.7 [M+H_2_O]^+^

**2-amino-N-(4-(1-hexyl-1H-1,2,3-triazol-4-yl)phenyl)acetamide (18f)**

Brown solid; ^1^H NMR (300 MHz, DMSO-d6) δ 11.13 (s, 1H), 8.57 (s, 1H), 8.42 (s, 3H), 7.83 (d, *J* = 8.6 Hz, 2H), 7.75 (d, *J* = 8.3 Hz, 2H), 4.39 (t, *J* = 6.9, 6.9 Hz, 2H), 3.85 (d, *J* = 5.5 Hz, 2H), 1.85 (p, *J* = 6.7, 6.7, 6.3, 6.3 Hz, 2H), 1.27 (s, 6H), 0.85 (t, *J* = 6.1, 6.1 Hz, 3H); ^13^C NMR (75 MHz, DMSO) δ 168.08, 149.22, 128.98, 125.00, 122.70, 120.20, 118.79, 48.94, 33.89, 32.87, 29.84, 25.22, 17.16, 13.24; ESI-MS: calcd. for C_16_H_23_N_5_O: 301.1902, found: 318.9 [M+H_2_O]^+^.

**Synthesis of vancomycin analogue substituents for Vanc-79-82 (possessing cataionic residue)**

**Scheme 8**.

**Reagents and conditions**: i) ethylchloroformate, THF, TEA, overnight, 0 ^o^C to rt; ii) LiOH/H_2_O, THF (1:1); 60 ^0^C, 5-6h; iii) HATU, DIPEA, DMF, rt, 7-8h; iv) alkyl bromide, chloroform, reflux, 10-12h; v) 4N HCl in 1,4-dioxane, DCM, 0 ^o^C to rt, 1h.

**Procedure**

1. **Synthesis of compound 19**

Compound **19** was synthesised by following the procedure used for the synthesis of compound **3**.

**methyl 4-(2-(tert-butoxycarbonylamino)acetamido)benzoate (19)**

White solid; ^1^H NMR (300 MHz, DMSO-d6) δ 10.29 (s, 1H), 7.93 (d, J = 8.8 Hz, 2H), 7.73 (d, J = 8.8 Hz, 2H), 7.11 (t, J = 6.1, 6.1 Hz, 1H), 3.83 (s, 3H), 3.76 (d, J = 6.1 Hz, 2H), 1.40 (s, 9H); ^13^C NMR (75 MHz, DMSO) δ 168.88, 165.80, 155.95, 143.34, 130.32, 123.87, 118.41, 78.11, 51.86, 43.92, 28.19; ESI-MS: calcd. for C_15_H_20_N_2_O_5_: 308.1372 found: 308.6.

**Spectral data of 20**

**tert-butyl 2-(4-(3-(dimethylamino)propylcarbamoyl)phenylamino)-2-oxoethylcarbamate (20)**

White solid; ^1^H NMR (300 MHz, DMSO-d6) δ 10.16 (s, 1H), 8.50 (t, J = 5.6, 5.6 Hz, 1H), 7.81 (d, J = 8.8 Hz, 1H), 7.67 (d, J = 8.7 Hz, 1H), 7.09 (t, J = 6.0, 6.0 Hz, 1H), 3.73 (d, J = 6.1 Hz, 2H), 3.30 (s, 2H), 3.13 – 3.03 (m, 2H), 2.78 (d, J = 4.7 Hz, 6H), 1.86 (dt, J = 14.3, 6.7, 6.7 Hz, 2H), 1.40 (s, 9H); ESI-MS calcd. for C_19_H_30_N_4_O_4_: 378.22, found: 379.60.

**Spectral data of 24d**

**4-(2-aminoacetamido)-N-(3-(dimethylamino)propyl)benzamide (24d)**

White solid; ^1^H NMR (300 MHz, DMSO-d6) δ 10.30 (s, 1H), 8.67 (t, J = 5.2, 5.2 Hz, 1H), 8.30 (s, 3H), 7.99 – 7.82 (m, 2H), 7.73 (t, J = 10.0, 10.0 Hz, 1H), 7.20 (d, J = 6.7 Hz, 1H), 3.84 (d, J = 5.6 Hz, 2H), 3.39-3.30 (m, 2H), 3.16 – 3.02 (m, 2H), 2.76 (s, 3H), 2.74 (s, 3H), 2.05 – 1.84 (m, 2H); ESI-MS calcd. for C_14_H_22_N_4_O_2_: 278.174 found: 279.8.

1. **Synthesis of compound 21**

**Compound 19** (0.5 mmol) was dissolved in THF (4 mL) and a solution of LiOH (2.5 mmol) in water (4 mL) was added dropwise, and the mixture was allowed to stir at rt for 5h. After completion of the reaction, THF was removed *in vacuo* and 1N HCl solution was added till pH 5, so that a white solid precipitated. This was washed with diethyl ether, dried *in* *vacuo* and used for the next step without purification.

1. **Synthesis of compound 22a-c**

Compounds **22a-c** were synthesised by according to a procedure reported in the literature.^6^

**N-(3-aminopropyl)-N,N-dimethylhexan-1-aminium hydrochloride salt (22a)**

Colorless viscous liquid; ^1^H NMR (300 MHz, DMSO-d6) δ 8.44 (s, 3H), 3.53 – 3.43 (m, 2H), 3.34 – 3.23 (m, 2H), 3.04 (s, 6H), 2.85 (hept, J = 6.2, 6.2, 6.2, 6.2, 5.7, 5.7 Hz, 2H), 2.12 – 1.99 (m, 2H), 1.76 – 1.60 (m, 2H), 1.30 (t, J = 14.4, 14.4 Hz, 6H), 0.90 – 0.84 (m, 3H); ^13^C NMR (75 MHz, DMSO) δ 64.00, 60.43, 50.57, 49.00, 36.45, 31.12, 25.91, 22.36, 22.09, 20.75, 14.31; ESI-MS calcd. for C_11_H_27_N_2_^+^: 187.21, found: 187.1.

**N-(3-aminopropyl)-N,N-dimethylpentan-1-aminium hydrochloride (22b)**

Colorless viscous liquid; ^1^H NMR (300 MHz, DMSO-d6) δ 8.58 (s, 3H), 3.61 – 3.49 (m, 2H), 3.37-3.31 (m, 2H), 3.09 (s, 6H), 2.94 – 2.81 (m, 2H), 2.10 (p, J = 6.8, 6.8, 6.5, 6.5 Hz, 2H), 1.72 (t, J = 11.2, 11.2 Hz, 2H), 1.39-1.23 (ddd, J = 25.4, 14.2, 6.3 Hz, 4H), 0.90 (t, J = 7.1, 7.1 Hz, 3H); ^13^C NMR (75 MHz, DMSO) δ 63.49, 59.93, 50.05, 35.95, 27.87, 21.62, 21.34, 20.28, 13.75.

**22c.**

**N-(3-aminopropyl)-N,N-dimethyloctan-1-aminium hydrochloride salt (22c)**

Clorless viscous liquid; spectral data were found in accordance with available literature.^6^

1. **Synthesis of compound 23**

To a solution of compound **21** (1.7 mmol) in DMF (5 mL) were added: HATU (1.96 mmol), **22** (1.80 mmol) and DIPEA (2.13 mmol), and the reaction mixture was stirred at rt for 4h. After completion of the reaction, the mixture was poured into water and extracted with ethyl acetate (2 x 30 mL). The organic layer was washed with brine and concentrated *in* *vacuo*. The crude product 23 was used directly for next step without further purification.

1. **Synthesis of compound 24.**

Compound **24** was synthesised by following the procedure used for the synthesis of compound **4**. The products were purified using preparative HPLC using water with 0.1% formic acid and ACN as eluent.

**Table S14**

| Entry | Product | R | Yield %  **24** | Vancomycin analogue |
| --- | --- | --- | --- | --- |
| 1 | **24a** | C_6_H_13_ | 40 | Vanc-79 |
| 2 | **24b** | C_5_H_11_ | 38 | Vanc-80 |
| 3 | **24c** | C_8_H_17_ | 35 | Vanc-81 |

**Spectral data of 21**

**4-(2-(tert-butoxycarbonylamino)acetamido)benzoic acid**

White solid; ^1^H NMR (300 MHz, DMSO-d6) δ 12.44 (s, 1H), 10.30 (s, 1H), 7.90 (d, J = 7.0 Hz, 1H), 7.71 (d, J = 7.2 Hz, 1H), 7.61 (d, J = 6.8 Hz, 1H), 7.14 – 7.04 (m, 1H), 6.55 (d, J = 6.9 Hz, 1H), 3.75 (d, J = 5.9 Hz, 2H), 1.40 (s, 9H); ^13^C NMR (75 MHz, DMSO) δ 168.73, 166.97, 155.89, 142.82, 130.34, 125.36, 118.24, 78.05, 43.86, 28.15; ESI-MS calculated for C_14_H_18_N_2_O_5_: 294.1215, found: 295.01 [M+H]^+^.

**Spectral data of 24a-c**

**24a**

**N-(3-(4-(2-aminoacetamido)benzamido)propyl)-N,N-dimethylhexan-1-aminium-formic acid salt**

White solid; ^1^H NMR (300 MHz, DMSO-d6) δ 11.3 (s, 1H), 8.43 (s, 1H), 7.76 (d, J = 8.8 Hz, 2H), 7.53 (d, J = 8.8 Hz, 2H), 3.31 – 3.17 (m, 8H), 3.00 (s, 6H), 1.96 – 1.82 (m, 2H), 1.70 – 1.55 (m, 2H), 1.49 (s, 9H), 1.34-1.24 (m, 6H), 0.95 – 0.76 (m, 3H).

**24b**

**N-(3-(4-(2-aminoacetamido)benzamido)propyl)-N,N-dimethylpentan-1-aminium-formic acid salt**

White solid; ^1^H NMR (300 MHz, DMSO-d6) δ 11.56 (bs, 2H), 11.38 (s, 1H), 8.97 – 8.82 (m, 1H), 8.76 (d, J = 3.5 Hz, 1H), 8.53 (d, J = 8.3 Hz, 2H), 7.77 (d, J = 8.5 Hz, 1H), 7.51 (dd, J = 8.4, 4.4 Hz, 1H), 3.91-3.79 (m, 2H), 3.63 – 3.52 (m, 2H), 3.47 – 3.22 (m, 4H), 3.07 (s, 6H), 1.99-1.88 (m, 2H), 1.33-1.19 (m, 6H), 0.88-0.80 (m, 3H); ^13^C NMR (75 MHz, DMSO) δ 166.07, 165.76, 165.07, 140.96, 128.64, 128.25, 120.57, 118.19, 62.83, 60.97, 53.19, 50.08, 41.50, 41.00, 38.18, 27.77, 21.52, 13.60. MS calcd. for C_19_H_33_N_4_O_2_^+^: 349.2590, found (LCMS): 349.30.

**24c**

**N-(3-(4-(2-aminoacetamido)benzamido)propyl)-N,N-dimethyloctan-1-aminium-formic acid salt**

^1^H NMR (300 MHz, DMSO-d6) δ 10.65 (s, 1H), 8.70 (t, J = 5.4, 5.4 Hz, 1H), 8.46 – 8.24 (m, 2H), 8.17 (d, J = 8.4 Hz, 1H), 7.88 (dd, J = 8.3, 3.7 Hz, 2H), 7.70 (t, J = 9.0, 9.0 Hz, 2H), 7.33 – 7.15 (m, 1H), 3.72 (s, 2H), 3.27-3.22 (m, 4H), 3.01 (s, 6H), 2.7-2.68 (m, 2H), 2.03 – 1.85 (m, 2H), 1.64-1.57 (m, 2H), 1.28-1.18(m, 10H), 0.85 (t, J = 6.3, 6.3 Hz, 3H); ^13^C NMR (75 MHz, DMSO) δ 165.83, 160.00, 146.78, 130.36, 128.20, 127.07, 118.41, 118.24, 62.87, 61.01, 50.10, 42.33, 38.22, 36.22, 31.13, 25.76, 22.55, 22.00, 21.66, 13.93. ESI-MS calcd. for C_22_H_39_N_4_O_2_^+^: 391.306, found: 391.8.

- 1. **Synthesis of biphenyl analog of vancomycin**

**Scheme 9**

**Reagents and conditions**: i) Pd(PPh_3_)_4_, Na_2_CO_3_, dioxane/H_2_O (7:3), MW, 100 ^o^C, 30 min; ii) 4N HCl in 1,4-dioxane, DCM, 0 ^o^C to rt, 1h.

1. **Synthesis of 26**

Phenyl boronic acid **25a-d**, (0.73 mmol, 1.1 equiv.), Pd(PPh_3_)_4_ (5 mol-%), and Na_2_CO_3_ (1.32 mmol, 2 equiv.) were added to a 10 mL reaction vial containing compound **3** (0.66 mmol, 1 equiv.). Then a mixture of dioxane/water (7:3; 2 mL) was added, and the vial was sealed tightly with a Teflon® cap. The mixture was irradiated for 30 min at a pre-selected temperature of 110 °C, with maximum irradiation power of 300 W. After the reaction was complete, as monitored by TLC and MS analysis, water (25 mL) was added, and the mixture was extracted with ethyl acetate (2 x 30 mL). The combined organic extracts were washed with brine (25 mL), and dried with Na_2_SO_4_. After filtration, the solvent was evaporated under reduced pressure, and the crude product was subjected to silica gel column chromatography (70–80% ethyl acetate in heptane) to give compound **27a-e**.

**Table S15**

| Entry | Product | R | Yield | Vancomycin analogue |
| --- | --- | --- | --- | --- |
| 1 | **27a** | OCH_3_ | 79 | Vanc-57 |
| 2 | **27b** | H | 78 | Vanc-83 |
| 3 | **27c** | Cl | 87 | Vanc-84 |
| 4 | **27d** | COCH_3_ | 83 | Vanc-85 |
| 5 | **27e** | OC_2_H_5_ | 82 | Vanc-86 |

**Spectral data of 26a-e**

**tert-butyl 2-(4'-methoxybiphenyl-4-ylamino)-2-oxoethylcarbamate (26a)**

White solid; ^1^H NMR (300 MHz, DMSO-d6) δ 9.99 (s, 1H), 7.65 (d, *J* = 8.7 Hz, 2H), 7.57 (dd, *J* = 8.7, 3.4 Hz, 4H), 7.07 (t, *J* = 6.0, 6.0 Hz, 1H), 7.00 (d, *J* = 8.8 Hz, 2H), 3.79 (s, 3H), 3.74 (d, *J* = 6.1 Hz, 2H), 1.40 (s, 9H); ^13^C NMR (75 MHz, DMSO) δ 168.30, 155.90, 138.70, 138.43, 133.32, 131.75, 128.78, 127.89, 126.90, 119.36, 78.02, 43.76, 28.16; ESI-MS calcd. for C_20_H_24_N_2_O_4_: 356.1736, found: ESI-MS: 256.9 [M+H]^+^ (-Boc).

**tert-butyl 2-(biphenyl-4-ylamino)-2-oxoethylcarbamate (26b)**

White solid; ^1^H NMR (300 MHz, DMSO-d6) δ 10.08 (s, 1H), 7.75 – 7.65 (m, 6H), 7.48 (t, *J* = 7.5, 7.5 Hz, 2H), 7.36 (t, *J* = 7.3, 7.3 Hz, 1H), 7.13 (t, *J* = 6.1, 6.1 Hz, 1H), 3.80 (d, *J* = 6.1 Hz, 2H), 1.45 (s, 9H); ^13^C NMR (75 MHz, DMSO) δ 168.24, 155.91, 139.64, 138.39, 134.76, 128.84, 126.95, 126.89, 126.18, 119.37, 78.02, 43.77, 28.17; ESI-MS: calcd. for C_19_H_22_N_2_O_3_: 326.1630, found: 326.7.

**tert-butyl 2-(4'-chlorobiphenyl-4-ylamino)-2-oxoethylcarbamate (26c)**

White solid; ^1^H NMR (300 MHz, DMSO-d6) δ 10.06 (s, 1H), 7.71-7.62 (m, 6H), 7.49 (d, *J* = 8.5 Hz, 2H), 7.08 (t, *J* = 6.0, 6.0 Hz, 1H), 3.75 (d, *J* = 6.1 Hz, 2H), 1.40 (s, 9H); ^13^C NMR (75 MHz, D_2_O) δ 161.22, 158.61, 140.43, 137.24, 134.77, 129.96, 129.03, 122.08, 116.96, 80.72, 46.44, 30.87; ESI-MS calcd. for C_19_H_21_ClN_2_O_3_: 326.1630, found 344.6 [M+H_2_O]^+^.

**tert-butyl 2-(4'-acetylbiphenyl-4-ylamino)-2-oxoethylcarbamate (26d)**

White solid; ^1^H NMR (300 MHz, DMSO-d6) δ 10.11 (s, 1H), 8.02 (d, *J* = 8.2 Hz, 2H), 7.81 (d, *J* = 8.3 Hz, 2H), 7.73 (s, 4H), 7.09 (t, *J* = 5.9, 5.9 Hz, 1H), 3.76 (d, *J* = 6.0 Hz, 2H), 2.60 (s, 3H), 1.41 (s, 9H); ^13^C NMR (75 MHz, DMSO) δ 197.34, 168.38, 155.90, 143.96, 139.27, 135.14, 133.27, 128.87, 127.34, 126.20, 119.36, 78.03, 43.78, 28.17, 26.69.

**tert-butyl 2-(4'-ethoxybiphenyl-4-ylamino)-2-oxoethylcarbamate (26e)**

White solid; ^1^H NMR (300 MHz, DMSO-d6) δ 9.98 (s, 1H), 7.64 (d, *J* = 8.6 Hz, 2H), 7.56 (d, *J* = 8.7 Hz, 4H), 7.07 (t, *J* = 5.7, 5.7 Hz, 1H), 6.98 (d, *J* = 8.5 Hz, 2H), 4.05 (q, *J* = 6.9, 6.9, 6.9 Hz, 2H), 3.74 (d, *J* = 6.0 Hz, 2H), 1.38 (d, *J* = 11.6 Hz, 9H), 1.33 (d, *J* = 6.9 Hz, 3H); ^13^C NMR (75 MHz, DMSO) δ 168.12, 157.78, 155.89, 137.70, 134.54, 131.92, 127.23, 126.29, 119.36, 114.72, 78.00, 62.99, 43.73, 28.17, 14.64; ESI-MS calcd. for C_21_H_26_N_2_O_4_: 370.1892, found: 372.0 [M+H]^+^

1. **Synthesis of compound 27a-e**

Compounds **27a-e** were synthesised by following the procedure used for the synthesis of **4**.

**Spectral data of 27a-e**

**2-amino-N-(4'-methoxybiphenyl-4-yl)acetamide (27a)**

White solid; ^1^H NMR (300 MHz, DMSO-d6) δ 10.93 (s, 1H), 8.33 (s, 3H), 7.71 (d, *J* = 8.6 Hz, 2H), 7.60 (dd, *J* = 8.5, 5.8 Hz, 4H), 7.01 (d, *J* = 8.7 Hz, 2H), 3.83 (s, 2H), 3.79 (s, 3H); ^13^C NMR (75 MHz, DMSO) δ 164.64, 158.62, 137.13, 135.12, 131.92, 127.32, 126.45, 119.48, 114.29, 55.12, 40.96.

**2-amino-N-(biphenyl-4-yl)acetamide (27b)**

White solid; ^1^H NMR (300 MHz, DMSO-d6) δ 11.13 (s, 1H), 8.43 (s, 3H), 7.79 (d, *J* = 8.6 Hz, 2H), 7.68-7.64 (m, 4H), 7.45 (t, *J* = 7.5, 7.5 Hz, 2H), 7.34 (t, *J* = 7.3, 7.3 Hz, 1H), 3.88 (s, 2H); ^13^C NMR (151 MHz, DMSO) δ 165.27, 140.01, 138.34, 135.82, 129.36, 127.57, 127.51, 126.73, 119.98, 41.50; HRMS (EI): calcd. for C_14_H_14_N_2_O: 226.1106, found: ESI-MS: 227.0 [M+H]^+^.

**2-amino-N-(4'-chlorobiphenyl-4-yl)acetamide (27c)**

White solid; ^1^H NMR (300 MHz, DMSO-d6) δ 10.06 (s, 1H), 7.71-7.62 (m, 6H), 7.49 (d, *J* = 8.5 Hz, 2H), 7.08 (t, *J* = 6.0, 6.0 Hz, 1H), 3.75 (d, *J* = 6.1 Hz, 2H), 1.40 (s, 9H); ^13^C NMR (75 MHz, D_2_O) δ 161.22, 158.61, 140.43, 137.24, 134.77, 129.96, 129.03, 122.08, 116.96, 80.72, 46.44, 30.87; ESI-MS: calcd. for C_14_H_13_ClN_2_O: 260.0716, found: 260.9 [M+H]^+^.

**N-(4'-acetylbiphenyl-4-yl)-2-aminoacetamide (27d)**

White solid; ^1^H NMR (300 MHz, DMSO-d6) δ 11.13 (s, 1H), 8.38 (s, 3H), 8.03 (d, *J* = 8.2 Hz, 2H), 7.84-7.76 (m, 6H), 3.87 (s, 2H), 2.61 (s, 3H); ^13^C NMR (75 MHz, DMSO) δ 197.40, 164.95, 143.83, 138.67, 135.27, 133.91, 128.90, 127.51, 126.32, 119.49, 41.05, 26.74; ESI-MS: calcd. for C_16_H_16_N_2_O_2_: 268.1211, found: 268.9.

**2-amino-N-(4'-ethoxybiphenyl-4-yl)acetamide (27e)**

White solid; ^1^H NMR (300 MHz, DMSO-d6) δ 11.02 (s, 1H), 8.39 (s, 3H), 7.73 (d, *J* = 8.6 Hz, 2H), 7.64 – 7.54 (m, 3H), 6.98 (d, *J* = 8.7 Hz, 2H), 4.05 (q, *J* = 7.0, 6.9, 6.9 Hz, 2H), 3.84 (s, 2H), 1.34 (t, *J* = 6.9, 6.9 Hz, 3H); ^13^C NMR (101 MHz, DMSO) δ 165.13, 158.39, 137.62, 135.64, 132.30, 127.80, 126.90, 120.00, 115.27, 63.53, 41.47, 15.13; ESI-MS calcd. for C_16_H_18_N_2_O_2_: 270.1368, found: 271.0 [M+H]^+^.

1. **N-Boc-1,2 ethylenediamine (Scheme 10)**

**Scheme 10**

**Reagents and conditions:** i) HATU, DIPEA, DMF, rt, 4h; ii) 4N HCl in 1,4-dioxane, DCM, 0 ^o^C to rt, 1h.

**2.1. Detailed synthetic route for the development of vancomycin analogue side chain R^2^NHR^1^NH_2_ (cinnamic acid derivatives).**

**2.1. For the synthesis of vancomycin analogue substituents derived from cinnamic acids (11t, Vanc- 21-23, 36).**

**Scheme 8.**

**Reagents and conditions:** i) *N*-Boc-ethylenediamine, HATU, DIPEA, DMF, rt, 4h; ii) 4N HCl in 1,4-dioxane, DCM, 0 ^o^C to rt, 1h; iii) substituted acetylene (when R’=H; TMS acetylene was used), piperidine, Pd(PPh_3_)Cl_2_, 85 ^o^C, 30 min; iv) If R’= TMS (trimethylsilane): TBAF, THF, rt, 30 min.

**Procedure:**

1. **Synthesis of 29.**

To a solution of compound **28** (1.63 mmol) in DMF (5 mL) was added: HATU (1.96 mmol), *N*-Boc-ethylenediamine **29** (1.80 mmol) and DIPEA (2.13 mmol), and the mixture was stirred at rt for 4h. After completion of the reaction, the reaction mass was poured into water and extracted with ethyl acetate (2 x 25 mL). The organic layer was washed with brine and concentrated under vacuum pressure, and crude product was subjected to silica gel column chromatography (2-4% methanol in DCM) to afford the corresponding compound **31**. Yield: 75-82%.

1. **Synthesis of 31**

**31** was synthesized by following the procedure used for the synthesis of **4**. Yield 62%.

1. **Synthesis of 32.**

**32** was synthesized by following the procedure used for the synthesis of product **5**.

1. **Synthesis of** **33**

**When R’=H**;

Compound **32** (0.5 mmol) was dissolved in 0.5 mL dioxane and 4.5 mL H_2_O in a screw-capped vial and heated at 140 ^o^C for 3h. After completion of the reaction, the reaction mass was cooled to rt, hence product **33** fell out as a white- to yellow-colored solid (depending upon the substitution). It was filtered through a Buchner funnel, washed with diethyl ether and dried under vacuum. Along with Boc, the TMS group was also removed by this procedure.^2^

**When R’= acyclic alkane;**

Boc deprotection of compound **32** was carried out by following the procedure (step ii’) used for the synthesis of **4**.

**Table S16**

| Entry | Product | R | Yield %  **30a-g** | Yield %  **31a-g** | Vancomycin analogue |
| --- | --- | --- | --- | --- | --- |
| 1 | **31a** | 4-Cl | 65 | 79 | Vanc-20 |
| 2 | **31b** | H | 72 | 71 | Vanc-21 |
| 3 | **31c** | 4-F | 69 | 70 | Vanc-22 |
| 4 | **31d** | 3-OCH_3_ | 73 | 73 | Vanc-23 |
| 5 | **31e** | 4-OCH_3_ | 75 | 82 | Vanc-36 |
| 6 | **31f** | 2, 4-Di Cl | 68 | 69 | Vanc-51 |
| 7 | **31g** | 4-CH_3_ | 72 | 72 | Vanc-53 |

**Spectral data of 30a-g**

**(E)-tert-butyl 2-(3-(4-chlorophenyl)acrylamido)ethylcarbamate (30a)**

Off-white solid; ^1^H NMR (300 MHz, DMSO-d6) δ 8.18 (t, *J* = 5.3, 5.3 Hz, 1H), 7.59 (d, *J* = 8.5 Hz, 2H), 7.48 (d, *J* = 8.5 Hz, 2H), 7.42 (d, *J* = 15.9 Hz, 1H), 6.87 (t, *J* = 5.4, 5.4 Hz, 1H), 6.62 (d, *J* = 15.8 Hz, 1H), 3.21 (q, *J* = 6.2, 6.2, 6.0 Hz, 2H), 3.04 (q, *J* = 6.0, 6.0, 6.0 Hz, 2H), 1.38 (s, 9H); ^13^C NMR (75 MHz, DMSO) δ 164.85, 155.59, 137.19, 133.82, 133.78, 129.13, 128.91, 122.96, 77.64, 38.20, 28.18; HRMS (EI): calcd. for C_16_H_21_ClN_2_O_3_: 324.1240, found: 324.12339.

**(E)-tert-butyl 2-cinnamamidoethylcarbamate (30b)**

White solid; ^1^H NMR (300 MHz, DMSO-d6) δ 8.15 (t, *J* = 5.0, 5.0 Hz, 1H), 7.56 (d, *J* = 6.5 Hz, 2H), 7.45-7.37 (m, 4H), 6.86 (t, *J* = 5.6, 5.6 Hz, 1H), 6.61 (d, *J* = 15.8 Hz, 1H), 3.21 (q, *J* = 6.1, 6.1, 6.0 Hz, 2H), 3.04 (q, *J* = 5.9, 5.9, 5.9 Hz, 2H), 1.38 (s, 9H); ^13^C NMR (75 MHz, DMSO) δ 165.06, 155.55, 138.53, 134.86, 129.36, 128.88, 127.43, 122.17, 77.64, 38.83, 38.65, 28.19; HRMS (EI): calcd. for C_16_H_22_N_2_O_3_: 290.1630, found: 290.16413.

**(E)-tert-butyl 2-(3-(4-fluorophenyl)acrylamido)ethylcarbamate (30c)**

White solid; ^1^H NMR (300 MHz, DMSO-d6) δ 8.15 (t, *J* = 5.7, 5.7 Hz, 1H), 7.63 (dd, *J* = 8.7, 5.6 Hz, 2H), 7.43 (d, *J* = 15.8 Hz, 1H), 7.25 (t, *J* = 8.9, 8.9 Hz, 2H), 6.87 (t, *J* = 5.5, 5.5 Hz, 1H), 6.56 (d, *J* = 15.8 Hz, 1H), 3.21 (q, *J* = 6.2, 6.2, 6.1 Hz, 2H), 3.04 (q, *J* = 6.4, 6.3, 6.3 Hz, 2H), 1.38 (s, 9H); ^13^C NMR (75 MHz, DMSO) δ 165.06, 164.24, 160.97, 155.62, 137.39, 131.47, 131.43, 129.59, 129.47, 122.01, 115.94, 115.65, 77.65, 39.74, 38.12, 28.12; HRMS (EI): calcd. for C_16_H_21_FN_2_O_3_: 308.1536, found: 308.15409.

**(E)-tert-butyl 2-(3-(3-methoxyphenyl)acrylamido)ethylcarbamate (30d)**

White solid; ^1^H NMR (300 MHz, DMSO-d6) δ 8.14 (t, *J* = 5.5, 5.5 Hz, 1H), 7.39 (d, *J* = 15.8 Hz, 1H), 7.33 (t, *J* = 7.9, 7.9 Hz, 1H), 7.16 – 7.10 (m, 2H), 6.95 (dd, *J* = 7.9, 2.1 Hz, 1H), 6.87 (t, *J* = 5.4, 5.4 Hz, 1H), 6.61 (d, *J* = 15.8 Hz, 1H), 3.78 (s, 3H), 3.21 (q, *J* = 5.5, 5.5, 4.5 Hz, 2H), 3.03 (q, *J* = 6.4, 6.2, 6.2 Hz, 2H), 1.38 (s, 9H); ^13^C NMR (75 MHz, DMSO) δ 165.02, 159.52, 155.59, 138.47, 136.28, 129.92, 122.48, 119.79, 115.16, 112.52, 77.56, 55.05, 38.19, 35.73, 28.18; HRMS (EI): calcd. for C_17_H_24_N_2_O_4_: 320.1736, found: 320.17562.

**(E)-tert-butyl 2-(3-(4-methoxyphenyl)acrylamido)ethylcarbamate (30e)**

White solid; ^1^H NMR (300 MHz, DMSO-d6) δ 8.06 (t, *J* = 5.5, 5.5 Hz, 1H), 7.51 (d, *J* = 8.7 Hz, 2H), 7.38 (d, *J* = 15.8 Hz, 1H), 6.97 (d, *J* = 8.8 Hz, 2H), 6.86 (t, *J* = 5.0, 5.0 Hz, 1H), 6.46 (d, *J* = 15.8 Hz, 1H), 3.79 (s, 3H), 3.20 (q, *J* = 6.2, 6.2, 6.0 Hz, 2H), 3.04 (q, *J* = 6.1, 6.0, 6.0 Hz, 2H), 1.38 (s, 9H); ^13^C NMR (75 MHz, DMSO) δ 165.33, 160.19, 155.54, 138.22, 128.95, 127.35, 119.59, 114.27, 77.59, 55.14, 38.85, 38.73, 28.13; HRMS (EI): calcd. for C_17_H_24_N_2_O_4_: 320.1736, found: 320.17333.

**(E)-tert-butyl 2-(3-(2,4-dichlorophenyl)acrylamido)ethylcarbamate (30f)**

Light-yellow solid; ^1^H NMR (300 MHz, DMSO-d6) δ 8.29 (t, *J* = 5.5, 5.5 Hz, 1H), 7.76 – 7.62 (m, 3H), 7.50 (dd, *J* = 8.5, 1.9 Hz, 1H), 6.88 (t, *J* = 5.5, 5.5 Hz, 1H), 6.68 (d, *J* = 15.7 Hz, 1H), 3.22 (q, *J* = 6.2, 6.2, 6.0 Hz, 2H), 3.05 (q, *J* = 6.2, 6.1, 6.1 Hz, 2H), 1.38 (s, 9H); ^13^C NMR (75 MHz, DMSO) δ 164.35, 155.59, 134.37, 133.98, 132.71, 131.75, 129.36, 128.72, 128.00, 125.91, 77.65, 39.61, 28.18; HRMS (EI): calcd. for C_16_H_20_Cl_2_N_2_O_3_: 358.0851, found: 358.0869.

**(E)-tert-butyl 2-(3-p-tolylacrylamido)ethylcarbamate (30g)**

White solid; ^1^H NMR (300 MHz, DMSO-d6) δ 8.12 (t, *J* = 5.8, 5.8 Hz, 1H), 7.45 (d, *J* = 7.8 Hz, 2H), 7.39 (d, *J* = 15.7 Hz, 1H), 7.22 (d, *J* = 7.8 Hz, 2H), 6.87 (t, *J* = 5.7, 5.7 Hz, 1H), 6.56 (d, *J* = 15.8 Hz, 1H), 3.21 (q, *J* = 6.3, 6.3, 6.1 Hz, 2H), 3.04 (q, *J* = 6.5, 6.3, 6.3 Hz, 2H), 2.32 (s, 3H), 1.38 (s, 9H); ^13^C NMR (75 MHz, DMSO) δ 165.21, 155.59, 139.09, 138.49, 132.08, 129.47, 127.40, 121.09, 77.62, 38.80, 38.61, 28.18, 20.88; HRMS (EI): calcd. for C_17_H_24_N_2_O_3_: 304.1786, found: 304.1778.

**Spectral data of 31a-g**

**(E)-N-(2-aminoethyl)-3-(4-chlorophenyl)acrylamide (31a)**

White solid; ^1^H NMR (300 MHz, DMSO-d6) δ 8.79 (t, *J* = 5.5, 5.5 Hz, 1H), 8.33 (bs, 3H), 7.58 (d, *J* = 8.5 Hz, 1H), 7.46 (d, *J* = 8.6 Hz, 1H), 7.43 (s, 1H), 6.69 (d, *J* = 15.9 Hz, 1H), 3.45 (q, *J* = 6.1, 6.1, 6.1 Hz, 2H), 2.92 (q, *J* = 6.0, 5.9, 5.9 Hz, 2H); ^13^C NMR (75 MHz, DMSO) δ 165.28, 137.44, 133.89, 133.70, 129.18, 128.93, 122.70, 38.38, 36.61; HRMS (EI): calcd. for C_11_H_13_ClN_2_O: 224.0716, found: 224.07166.

**N-(2-aminoethyl)cinnamamide (31b)**

White solid; ^1^H NMR (300 MHz, DMSO-d6) δ 8.77 (t, *J* = 5.5, 5.5 Hz, 1H), 8.33 (s, 3H), 7.58 (dd, *J* = 7.8, 1.5 Hz, 2H), 7.49 (d, *J* = 16.0 Hz, 1H), 7.45 – 7.34 (m, 3H), 6.70 (d, *J* = 15.8 Hz, 1H), 3.47 (q, *J* = 6.1, 6.1, 6.1 Hz, 2H), 2.94 (q, *J* = 6.0, 5.9, 5.9 Hz, 2H); ^13^C NMR (75 MHz, DMSO) δ 165.42, 138.74, 134.66, 129.43, 128.83, 127.41, 121.82, 38.35, 36.54; HRMS (EI): calcd. for C_11_H_14_N_2_O: 190.1106, found ESI-MS: 191.10.

**(E)-N-(2-aminoethyl)-3-(4-fluorophenyl)acrylamide (31c)**

White solid; ^1^H NMR (300 MHz, DMSO-d6) δ 8.81 (t, *J* = 5.5, 5.5 Hz, 1H), 8.40 (s, 3H), 7.64 (dd, *J* = 8.8, 5.6 Hz, 2H), 7.49 (d, *J* = 15.9 Hz, 1H), 7.27 (t, *J* = 8.9, 8.9 Hz, 2H), 6.67 (d, *J* = 15.9 Hz, 1H), 3.48 (q, *J* = 6.1, 6.1, 6.1 Hz, 2H), 2.94 (q, *J* = 6.0, 5.9, 5.9 Hz, 2H); ^13^C NMR (75 MHz, DMSO) δ 165.39, 164.28, 161.00, 137.57, 131.39, 129.68, 129.57, 121.82, 121.79, 116.01, 115.72, 38.38, 36.59; HRMS (EI): calcd. for C_11_H_13_FN_2_O: 208.1011, found: 208.10266.

**(E)-N-(2-aminoethyl)-3-(3-methoxyphenyl)acrylamide (31d)**

White solid; ^1^H NMR (300 MHz, DMSO-d6) δ 8.14 (t, *J* = 5.5, 5.5 Hz, 1H), 7.39 (d, *J* = 15.8 Hz, 1H), 7.33 (t, *J* = 7.9, 7.9 Hz, 1H), 7.16 – 7.10 (m, 2H), 6.95 (dd, *J* = 7.9, 2.1 Hz, 1H), 6.87 (t, *J* = 5.4, 5.4 Hz, 1H), 6.61 (d, *J* = 15.8 Hz, 1H), 3.78 (s, 3H), 3.21 (q, *J* = 5.5, 5.5, 4.5 Hz, 2H), 3.03 (q, *J* = 6.4, 6.2, 6.2 Hz, 2H), 1.38 (s, 9H); ^13^C NMR (75 MHz, DMSO) δ 165.02, 159.52, 155.59, 138.47, 136.28, 129.92, 122.48, 119.79, 115.16, 112.52, 77.56, 55.05, 38.19, 35.73, 28.18; HRMS (EI): calcd. for C_17_H_24_N_2_O_4_: 320.1736, found: 320.17562.

**(E)-N-(2-aminoethyl)-3-(4-methoxyphenyl)acrylamide (31e)**

White solid; ^1^H NMR (300 MHz, DMSO-d6) δ 8.66 (t, *J* = 5.5, 5.5 Hz, 1H), 8.33 (s, 3H), 7.52 (d, *J* = 8.8 Hz, 2H), 7.44 (d, *J* = 15.9 Hz, 1H), 6.98 (d, *J* = 8.8 Hz, 2H), 6.55 (d, *J* = 15.8 Hz, 1H), 3.79 (s, 3H), 3.46 (q, *J* = 6.1, 6.1, 6.0 Hz, 2H), 2.93 (t, *J* = 6.3, 6.3 Hz, 2H); ^13^C NMR (75 MHz, DMSO) δ 165.81, 160.32, 138.56, 129.07, 127.28, 119.35, 114.34, 55.22, 38.49, 36.59; HRMS (EI): calcd. for C_12_H_16_N_2_O_2_: 220.1211, found: 220.12165.

**(E)-N-(2-aminoethyl)-3-(2,4-dichlorophenyl)acrylamide (31f)**

White solid; ^1^H NMR (300 MHz, DMSO-d6) δ 8.85 (t, *J* = 5.5, 5.5 Hz, 1H), 8.24 (s, 3H), 7.75 – 7.66 (m, 3H), 7.52 (dd, *J* = 8.5, 2.1 Hz, 1H), 6.76 (d, *J* = 15.7 Hz, 1H), 3.47 (q, *J* = 6.1, 6.1, 6.1 Hz, 2H), 2.95 (s, 2H); ^13^C NMR (75 MHz, DMSO) δ 164.81, 134.49, 134.03, 132.96, 131.62, 129.38, 128.73, 128.04, 125.59, 38.36, 36.69; HRMS (EI): calcd. for C_11_H_22_Cl_2_N_2_O: 258.0326, found: 258.0320.

**(E)-N-(2-aminoethyl)-3-p-tolylacrylamide (31g)**

White solid; ^1^H NMR (300 MHz, DMSO-d6) δ 8.71 (t, *J* = 5.5, 5.5 Hz, 1H), 8.32 (s, 3H), 7.51 – 7.41 (m, 3H), 7.23 (d, *J* = 8.0 Hz, 2H), 6.65 (d, *J* = 15.9 Hz, 1H), 3.47 (q, *J* = 5.9, 5.9, 5.7 Hz, 2H), 2.95 (t, *J* = 6.2, 6.2 Hz, 2H), 2.32 (s, 3H); ^13^C NMR (75 MHz, DMSO) δ 165.66, 139.25, 138.80, 131.96, 129.49, 127.46, 120.80, 38.47, 36.60, 20.90; HRMS (EI): calcd. for C_12_H_16_N_2_O: 204.1262, found: 204.1274.

**Table S18**

| Entry: | Product | R’ | Yield %  **32a-b** | Yield %  **33a-b** | Vancomycin analogue |
| --- | --- | --- | --- | --- | --- |
| 1 | 33a | C_4_H_9_ | 65 | 72 | Vanc-48 |
| 2 | 33b | H | 78 | 75 | Vanc-Q |

**Spectral data of compound 32a-b and 33a-b**

**(E)-tert-butyl 2-(3-(4-(hex-1-ynyl)phenyl)acrylamido)ethylcarbamate (32a)**

Light-yellow solid; ^1^H NMR (300 MHz, DMSO-d6) δ 8.17 (t, *J* = 5.8, 5.8 Hz, 1H), 7.53 (d, *J* = 8.1 Hz, 2H), 7.46 – 7.35 (m, 3H), 6.87 (t, *J* = 5.1, 5.1 Hz, 1H), 6.61 (d, *J* = 15.8 Hz, 1H), 3.21 (q, *J* = 5.8, 5.7, 5.7 Hz, 2H), 3.10 – 2.98 (m, 2H), 2.44 (t, *J* = 6.7, 6.7 Hz, 2H), 1.59 – 1.41 (m, 4H), 1.38 (s, 9H), 0.92 (t, *J* = 7.1, 7.1 Hz, 3H); ^13^C NMR (75 MHz, DMSO) δ 164.91, 155.58, 137.71, 134.28, 131.67, 127.56, 124.10, 122.73, 92.38, 80.38, 77.62, 38.91, 38.83, 30.17, 28.18, 21.40, 18.36, 13.43; HRMS (EI): calcd. for C_22_H_30_N_2_O_3_: 370.2256, found: 370.2258.

**(E)-N-(2-aminoethyl)-3-(4-(hex-1-ynyl)phenyl)acrylamide (33a)**

White solid; ^1^H NMR (300 MHz, DMSO-d6) δ 8.71 (t, *J* = 5.5, 5.5 Hz, 1H), 8.22 (s, 3H), 7.55 (d, *J* = 8.3 Hz, 2H), 7.46 (d, *J* = 15.9 Hz, 1H), 7.41 (d, *J* = 8.3 Hz, 2H), 6.68 (d, *J* = 15.8 Hz, 1H), 3.46 (q, *J* = 6.0, 6.0, 6.0 Hz, 2H), 2.94 (q, *J* = 5.7, 5.6, 5.6 Hz, 2H), 2.44 (t, *J* = 6.8, 6.8 Hz, 2H), 1.58-1.37 (m, 4H), 0.92 (t, *J* = 7.2, 7.2 Hz, 3H); ^13^C NMR (75 MHz, DMSO) δ 165.38, 138.02, 134.14, 131.69, 127.62, 124.23, 122.41, 92.49, 80.37, 38.45, 36.63, 30.16, 21.40, 18.36, 13.44; HRMS (EI): calcd. for C_17_H_22_N_2_O: 270.1732, found: 270.1713.

**(*E*)-*tert*-Butyl 2-(3-(4-(trimethylsilyl)ethynyl)phenyl) acrylamido)ethylcarbamate (32b).**

White solid; ^1^H NMR (300 MHz, DMSO-d_6_): δ 8.17 (t, *J* = 5.8 Hz, 1H), 7.54 (d, *J* = 8.4 Hz, 2H,), 7.46 (d, *J* = 8.4 Hz, 2H), 7.40 (d, *J* = 15.6 Hz, 1H), 6.85 (t, *J* = 5.8 Hz, 1H), 6.63 (t, *J* = 15.6 Hz, 1H), 3.20 (q, *J* = 6.1 Hz, 2H), 3.02 (q, *J* = 6.1 Hz, 2H), 1.37 (s, 9H), .0.02 (s, 9H); ^13^C NMR (75 MHz, DMSO-d_6_): 165.0, 155.7, 137.6, 135.5, 132.2, 127.8, 123.6, 122.9, 105.0, 95.9, 77.8, 40.4, 38.8, 28.3, 0.014; HRMS (EI): calcd. for C_21_H_30_N_2_O_3_Si: 386.2026, found: 386.2037.

**(*E*)-N-(2-Aminoethyl)-3-(4-ethynylphenyl)acrylamide (33b).**

White solid; ^1^H NMR (300 MHz, DMSO-d_6_): δ 8.15 (s, 1H), 7.31 (dd, 8.0 Hz, 8.0 Hz, 4H), 7.20 (d, *J* = 15.8, Hz, 1H), 6.46 (d, 15.8 Hz, 1H), 4.09 (s, 1H), 3.46 (s, 2H), 3.00 (d, *J* = 5.8 Hz, 2H); ^13^C NMR (75 MHz, DMSO-d_6_): δ 164.83, 137.41, 135.43, 132.18, 127.64, 123.49, 122.36, 83.24, 82.24, 41.54, 40.81; HRMS (EI): calcd. for C_13_H_14_N_2_O: 214.1106, found: 214.1125.

**2.2. Synthesis of Vancomycin analogue substituents for Vanc- 54-56 & 64 (sulphonamide derivative).**

**Scheme 9.**

**Reagents and condition**: i) pyridine, rt, 5-6h; ii) *N*-Boc-1,2-ethanediamine, HATU, DIPEA, DMF, rt, 4h; iii) 4N HCl in 1,4-dioxane, DCM, 0 ^o^C to rt, 2h.

**Procedure**

1. Synthesis of compound **36**

Compound **36** was synthesised by following the procedure used for the synthesis of product **12,** using **34** and **35** as starting materials.

1. Synthesis of compound **37**

Compound **37** was synthesised by following the procedure used for the synthesis of compound **29**.

1. Synthesis of compound **38**

Compounds **37** was dissolved in CH_2_Cl_2_ (5 mL), HCl-dioxane (4N solution, 4 mL) was added, and the mixture was stirred at rt for 3-4h. The reaction was monitored by TLC (MeOH/DCM, 1:9, Rf = 0.1). After completion of the reaction, the solvents were removed *in vacuo* and the product **4** was washed with diethyl ether and dried *in* *vacuo*. Yield: 70%

**Table S18**

| Entry | Product | R | Yield %  **36a-e** | Yield %  **37a-e** | Yield %  **38a-e** | Vancomycin analogue |
| --- | --- | --- | --- | --- | --- | --- |
| 1 | **38a** | H | 76 | 75 | 72 | Vanc-54 |
| 2 | **38b** | CH_3_ | 78 | 72 | 75 | Vanc-55 |
| 3 | **38c** | Isopropyl | 74 | 72 | 74 | Vanc-56 |
| 4 | **38d** | 4-Cl | 70 | 71 | 70 | Vanc-62 |
| 5 | **38e** | 2,4-DiCl | 70 | 71 | 72 | Vanc-64 |

**Spectral data of 36a-e**

**(E)-3-(4-(phenylsulfonamido)phenyl)acrylic acid (36a)**

White solid;^1^H NMR (300 MHz, DMSO-d6) δ 12.34 (s, 1H), 10.66 (s, 1H), 7.86 – 7.78 (m, 2H), 7.66-7.54 (m, 5H), 7.48 (d, *J* = 16.0 Hz, 1H), 7.14 (d, *J* = 8.6 Hz, 2H), 6.39 (d, *J* = 16.0 Hz, 1H); ^13^C NMR (75 MHz, DMSO) δ 167.55, 143.11, 139.42, 139.32, 133.06, 129.63, 129.34, 129.32, 126.59, 119.15, 117.92; HRMS (EI): calcd. for C_15_H_13_NO_4_S: 303.0565, found: 303.0545.

**(E)-3-(4-(4-methylphenylsulfonamido)phenyl)acrylic acid (36b)**

Yellow solid; ^1^H NMR (300 MHz, DMSO-d6) δ 12.31 (s, 1H), 10.57 (s, 1H), 7.69 (d, *J* = 8.3 Hz, 2H), 7.56 (d, *J* = 8.7 Hz, 2H), 7.46 (d, *J* = 16.0 Hz, 1H), 7.36 (d, *J* = 8.1 Hz, 2H), 7.12 (d, *J* = 8.6 Hz, 2H), 6.38 (d, *J* = 15.9 Hz, 1H), 2.33 (s, 3H); ^13^C NMR (75 MHz, DMSO) δ 167.56, 143.47, 143.14, 139.57, 136.47, 129.76, 129.49, 129.30, 126.65, 119.01, 117.84, 20.91; HRMS (EI): calcd. for C_16_H_15_NO_4_S: 317.0721, found: 317.0728.

**(E)-3-(4-(4-isopropylphenylsulfonamido)phenyl)acrylic acid (36c)**

White solid; ^1^H NMR (300 MHz, DMSO-d6) δ 12.29 (s, 1H), 10.60 (s, 1H), 7.73 (d, *J* = 8.4 Hz, 2H), 7.56 (d, *J* = 8.7 Hz, 2H), 7.49 – 7.41 (m, 3H), 7.13 (d, *J* = 8.6 Hz, 2H), 6.37 (d, *J* = 16.0 Hz, 1H), 2.93 (p, *J* = 6.8, 6.8, 6.8, 6.8 Hz, 1H), 1.17 (d, *J* = 6.9 Hz, 6H); ^13^C NMR (75 MHz, DMSO) δ 167.56, 153.83, 143.14, 139.61, 136.96, 129.39, 129.35, 127.28, 126.75, 118.77, 117.80, 33.27, 23.30; HRMS (EI): calcd. for C_18_H_19_NO_4_S: 345.1034, found: 345.1042.

**(E)-3-(4-(4-chlorophenylsulfonamido)phenyl)acrylic acid (36d)**

White solid; ^1^H NMR (300 MHz, DMSO-d6) δ 12.17 (s, 1H), 10.73 (s, 1H), 7.81 (d, *J* = 8.6 Hz, 2H), 7.62 (dd, *J* = 19.5, 8.6 Hz, 4H), 7.49 (d, *J* = 16.0 Hz, 1H), 7.14 (d, *J* = 8.5 Hz, 2H), 6.41 (d, *J* = 16.0 Hz, 1H); ^13^C NMR (75 MHz, DMSO) δ 167.53, 143.04, 139.08, 138.12, 137.97, 129.93, 129.53, 129.38, 128.54, 119.48, 118.12; HRMS (EI): calcd. for C_15_H_12_ClNO_4_S: 337.0175, found: 337.01775.

**(E)-3-(4-(2,4-dichlorophenylsulfonamido)phenyl)acrylic acid (36e)**

White solid; ^1^H NMR (300 MHz, DMSO-d6) δ 12.32 (s, 1H), 11.05 (s, 1H), 8.08 (d, *J* = 8.6 Hz, 1H), 7.86 (d, *J* = 2.0 Hz, 1H), 7.63 (dd, *J* = 8.6, 2.0 Hz, 1H), 7.56 (d, *J* = 8.6 Hz, 2H), 7.45 (d, *J* = 16.0 Hz, 1H), 7.10 (d, *J* = 8.6 Hz, 2H), 6.37 (d, *J* = 16.0 Hz, 1H); ^13^C NMR (75 MHz, DMSO) δ 167.52, 143.01, 138.85, 138.47, 135.27, 132.96, 131.91, 131.45, 129.72, 129.39, 128.02, 118.59, 118.06; HRMS (EI): calcd. for C_15_H_11_Cl_2_NO_4_S: 370.9785, found: 370.97539.

**Spectral data of 37a-e**

**(E)-tert-butyl 2-(3-(4-(phenylsulfonamido)phenyl)acrylamido)ethylcarbamate (37a)**

Yellow solid; ^1^H NMR (300 MHz, DMSO-d6) δ 10.56 (s, 1H), 8.09 (t, *J* = 5.5, 5.5 Hz, 1H), 7.84 – 7.76 (m, 2H), 7.67 – 7.48 (m, 3H), 7.43 (d, *J* = 8.7 Hz, 2H), 7.29 (d, *J* = 15.8 Hz, 1H), 7.13 (d, *J* = 8.6 Hz, 2H), 6.85 (t, *J* = 5.6, 5.6 Hz, 1H), 6.45 (d, *J* = 15.8 Hz, 1H), 3.18 (q, *J* = 6.2, 6.2, 6.0 Hz, 2H), 3.01 (q, *J* = 6.3, 6.1, 6.1 Hz, 2H), 1.37 (s, 9H); ^13^C NMR (75 MHz, DMSO) δ 165.08, 155.56, 150.89, 139.38, 138.66, 137.77, 133.01, 130.40, 129.30, 128.50, 126.58, 119.54, 77.63, 64.87, 35.73, 30.72, 28.17; ESI-MS: calcd. for C_22_H_27_N_3_O_5_S: 445.1671, found: ESI-MS: 446.0

**(E)-tert-butyl 2-(3-(4-(4-methylphenylsulfonamido)phenyl)acrylamido)ethylcarbamate (37b)**

White solid; ^1^H NMR (300 MHz, DMSO-d6) δ 10.50 (s, 1H), 8.10 (t, *J* = 5.7, 5.7 Hz, 1H), 7.69 (d, *J* = 8.3 Hz, 2H), 7.43 (d, *J* = 8.7 Hz, 2H), 7.36 (d, *J* = 8.0 Hz, 2H), 7.31 (d, *J* = 15.8 Hz, 1H), 7.13 (d, *J* = 8.6 Hz, 2H), 6.86 (t, *J* = 5.5, 5.5 Hz, 1H), 6.46 (d, *J* = 15.8 Hz, 1H), 3.19 (q, *J* = 6.2, 6.2, 6.0 Hz, 2H), 3.03 (q, *J* = 6.5, 6.3, 6.3 Hz, 2H), 2.33 (s, 3H), 1.38 (s, 9H); ^13^C NMR (75 MHz, DMSO) δ 165.11, 155.57, 143.39, 138.82, 137.81, 136.53, 130.26, 129.71, 128.77, 128.49, 126.64, 119.38, 77.6235.72, 30.71, 28.17, 20.90; HRMS (EI): calcd. for C_23_H_29_N_3_O_5_S: 459.1827, found: 359.1268 [M]^+^-Boc.

**(E)-tert-butyl 2-(3-(4-(4-isopropylphenylsulfonamido)phenyl)acrylamido)ethylcarbamate (37c)**

White solid; ^1^H NMR (300 MHz, DMSO-d6) δ 10.54 (s, 1H), 8.10 (t, *J* = 5.5, 5.5 Hz, 1H), 7.73 (d, *J* = 8.4 Hz, 2H), 7.44 (d, *J* = 8.5 Hz, 4H), 7.30 (d, *J* = 15.8 Hz, 1H), 7.15 (d, *J* = 8.6 Hz, 2H), 6.85 (t, *J* = 5.5, 5.5 Hz, 1H), 6.45 (d, *J* = 15.8 Hz, 1H), 3.19 (d, *J* = 6.1 Hz, 2H), 3.08 – 2.97 (m, 2H), 2.92 (d, *J* = 6.9 Hz, 1H), 1.37 (s, 9H), 1.17 (d, *J* = 6.9 Hz, 6H); ^13^C NMR (75 MHz, DMSO) δ 165.10, 155.57, 153.77, 138.85, 137.81, 137.02, 130.17, 128.53, 127.24, 126.75, 120.95, 119.14, 77.62, 38.78, 38.19, 33.27, 28.17, 23.30; HRMS (EI).

**(E)-tert-butyl 2-(3-(4-(4-chlorophenylsulfonamido)phenyl)acrylamido)ethylcarbamate (37d)**

White solid; ^1^H NMR (300 MHz, DMSO-d6) δ 10.64 (s, 1H), 8.10 (t, *J* = 5.6, 5.6 Hz, 1H), 7.78 (d, *J* = 8.8 Hz, 2H), 7.64 (d, *J* = 8.8 Hz, 2H), 7.44 (d, *J* = 8.6 Hz, 2H), 7.30 (d, *J* = 15.8 Hz, 1H), 7.12 (d, *J* = 8.6 Hz, 2H), 6.85 (t, *J* = 5.6Hz, 5.6 Hz, 1H, 6.45 (d, *J* = 15.8 Hz, 1H), 3.18 (q, *J* = 6.2, 6.2, 6.2 Hz, 2H), 3.01 (q, *J* = 6.5, 6.3, 6.3 Hz, 2H), 1.37 (s, 9H); ^13^C NMR (75 MHz, DMSO) δ 165.56, 144.27, 142.46, 138.54, 138.25, 138.09, 137.88, 130.54, 129.46, 128.58, 120.92, 119.89, 38.48, 36.61.

**(E)-tert-butyl 2-(3-(4-(2,4-dichlorophenylsulfonamido)phenyl)acrylamido)ethylcarbamate (37e)**

White solid; ^1^H NMR (300 MHz, DMSO-d6) δ 10.98 (s, 1H), 8.07 (d, *J* = 8.6 Hz, 2H), 7.88 – 7.85 (m, 1H), 7.63 (dd, *J* = 8.6, 2.1 Hz, 1H), 7.49 – 7.40 (m, 2H), 7.29 (dd, *J* = 15.8, 2.7 Hz, 1H), 7.11 (d, *J* = 8.6 Hz, 2H), 6.84 (t, *J* = 5.7, 5.7 Hz, 1H), 6.45 (dd, *J* = 15.8, 5.1 Hz, 1H), 3.18 (q, *J* = 7.2, 6.8, 6.8 Hz, 2H), 3.01 (q, *J* = 5.8, 5.8, 5.8 Hz, 2H), 1.36 (s, 9H); ^13^C NMR (75 MHz, DMSO) δ 165.09, 155.58, 138.64, 138.29, 138.18, 137.75, 132.84, 131.93, 131.39, 128.59, 128.40, 127.95, 121.04, 119.04, 77.64, 38.91, 38.63, 28.19; HRMS (EI): calcd. for C_22_H_25_Cl_2_N_3_O_5_S: 513.0892; found: 394.0164.

**Spectral data of 38a-e**

**(E)-N-(2-aminoethyl)-3-(4-(phenylsulfonamido)phenyl)acrylamide (38a)**

White solid; ^1^H NMR (300 MHz, DMSO-d6) δ 10.67 (s, 1H), 8.58 – 8.48 (m, 1H), 8.14 (s, 3H), 7.84-7.80 (m, 2H), 7.67 – 7.49 (m, 3H), 7.44 (d, *J* = 8.6 Hz, 2H), 7.34 (d, *J* = 15.8 Hz, 1H), 7.16 (d, *J* = 8.6 Hz, 2H), 6.49 (d, *J* = 15.8 Hz, 1H), 3.46 – 3.34 (m, 2H), 2.93-2.87 (m, 2H); ; ^13^C NMR (75 MHz, DMSO) δ 165.58, 139.40, 138.86, 138.16, 133.00, 130.17, 129.29, 128.55, 126.61, 120.69, 119.52, 38.48, 36.59.

**(E)-N-(2-aminoethyl)-3-(4-(4-methylphenylsulfonamido)phenyl)acrylamide (38b)**

Yellow solid; ^1^H NMR (300 MHz, DMSO-d6) δ 10.60 (s, 1H), 8.63 – 8.48 (m, 1H), 8.17 (s, 3H), 7.69 (d, *J* = 8.3 Hz, 2H), 7.43 (d, *J* = 8.6 Hz, 2H), 7.37 (s, 1H), 7.33 (d, *J* = 7.0 Hz, 2H), 7.15 (d, *J* = 8.6 Hz, 2H), 6.50 (d, *J* = 15.8 Hz, 1H), 3.45-3.37 (m, 2H), 2.93-2.87 (m, 2H), 2.33 (s, 3H); ^13^C NMR (75 MHz, DMSO) δ 165.59, 143.38, 139.01, 138.18, 136.56, 130.07, 129.71, 128.53, 126.67, 120.63, 119.37, 38.47, 36.60, 20.92; HRMS (EI): calcd. for C_18_H_21_N_3_O_3_S: 359.1303, found:; Observed: 378.12074 [M+H_2_O]^+^

**(E)-N-(2-aminoethyl)-3-(4-(4-isopropylphenylsulfonamido)phenyl)acrylamide (38c)**

Light-yellow solid; ^1^H NMR (300 MHz, DMSO-d6) δ 10.66 (s, 1H), 8.58 (t, *J* = 5.6, 5.6 Hz, 1H), 8.19 (s, 3H), 7.75 (d, *J* = 8.4 Hz, 2H), 7.44 (dd, *J* = 8.5, 2.2 Hz, 4H), 7.35 (d, *J* = 15.8 Hz, 1H), 7.18 (d, *J* = 8.6 Hz, 2H), 6.51 (d, *J* = 15.83 Hz, 1H), 3.45-3.37 (m, 2H), 2.98 – 2.86 (m, 2H), 1.17 (d, *J* = 6.9 Hz, 6H); ^13^C NMR (75 MHz, DMSO) δ 165.59, 153.75, 139.05, 138.19, 137.05, 129.97, 128.58, 127.24, 126.77, 120.58, 119.12, 38.47, 36.60, 33.27, 23.32; HRMS (EI): calcd. for C_20_H_25_N_3_O_3_S: 387.1616, found: 387.1628.

**(E)-N-(2-aminoethyl)-3-(4-(4-chlorophenylsulfonamido)phenyl)acrylamide (38d)**

White solid; ^1^H NMR (300 MHz, DMSO-d6) δ 10.73 (s, 1H), 8.51 (s, 1H), 8.06 (s, 3H), 7.80 (d, *J* = 8.8 Hz, 2H), 7.68 – 7.58 (m, 2H), 7.46 (d, *J* = 8.6 Hz, 2H), 7.35 (d, *J* = 15.8 Hz, 1H), 7.15 (d, *J* = 8.6 Hz, 2H), 6.50 (d, *J* = 15.8 Hz, 1H), 3.39 (dd, *J* = 11.7, 6.7 Hz, 2H), 2.90 (q, *J* = 5.9, 5.9, 5.8 Hz, 2H); ^13^C NMR (75 MHz, DMSO) δ 165.59, 138.52, 138.21, 138.15, 137.90, 131.10, 130.50, 129.49, 128.64, 128.57, 120.84, 119.84, 118.90, 38.52, 36.61; HRMS (EI): calcd. for C_17_H_18_ClN_3_O_3_S: 379.0757, found: 395.00839 and 363.05079.

**(E)-N-(2-aminoethyl)-3-(4-(2,4-dichlorophenylsulfonamido)phenyl)acrylamide (38e)**

White solid; ^1^H NMR (300 MHz, DMSO-d6) δ 11.06 (s, 1H), 8.62 (s, 1H), 8.22 (s, 3H), 8.08 (d, *J* = 8.6 Hz, 1H), 7.89 – 7.85 (m, 1H), 7.64 (dd, *J* = 8.6, 2.1 Hz, 1H), 7.52 – 7.29 (m, 3H), 7.14 (d, *J* = 8.6 Hz, 2H), 6.52 (dd, *J* = 15.8, 4.6 Hz, 1H), 3.46 – 3.37 (m, 2H), 2.90 (q, *J* = 5.6, 5.6, 5.5 Hz, 2H); ^13^C NMR (75 MHz, DMSO) δ 165.51, 138.75, 138.02, 135.39, 132.91, 131.93, 131.41, 130.34, 128.63, 128.00, 120.88, 118.98, 118.53, 38.43, 36.60; HRMS (EI): calcd. for C_17_H_17_Cl_2_N_3_O_3_S: 413.0367, found: 449.1356 (HCl salt).

**tert-butyl 2-(9H-fluorene-9-carboxamido)ethylcarbamate (30h)**

Light pink solid; ^1^H NMR (300 MHz, DMSO-d6) δ 8.42 (t, *J* = 5.3, 5.3 Hz, 1H), 7.87 (d, *J* = 7.4 Hz, 2H), 7.55 (d, *J* = 7.4 Hz, 2H), 7.41 (t, *J* = 7.3, 7.3 Hz, 2H), 7.34-7.29 (m, 3H), 6.90 (t, *J* = 5.2, 5.2 Hz, 1H), 4.79 (s, 1H), 3.21 – 3.13 (m, 2H), 3.09 – 3.01 (m, 2H), 1.41 (s, 9H); ^13^C NMR (75 MHz, DMSO) δ 169.74, 155.67, 142.85, 141.23, 140.33, 128.45, 128.14, 127.57, 127.13, 126.53, 124.84, 120.00, 77.66, 54.60, 39.06, 28.21; HRMS (EI): calcd. for C_21_H_24_N_2_O_3_: 352.1786, found: 352.1769.

**N-(2-aminoethyl)-9H-fluorene-9-carboxamide (31h)**

Light-pink solid; ^1^H NMR (300 MHz, DMSO-d6) δ 8.95 (t, *J* = 5.5, 5.5 Hz, 1H), 8.30 (s, 3H), 7.88 (d, *J* = 7.4 Hz, 2H), 7.63 (d, *J* = 7.5 Hz, 2H), 7.42 (t, *J* = 7.2, 7.2 Hz, 2H), 7.33 (td, *J* = 7.4, 7.4, 1.2 Hz, 3H), 4.88 (s, 1H), 3.48 – 3.37 (m, 2H), 2.93 (s, 2H); ^13^C NMR (75 MHz, DMSO) δ 170.25, 142.63, 141.23, 127.64, 127.14, 125.08, 120.01, 54.45, 38.36, 36.82; HRMS (EI): calcd. for C_16_H_16_N_2_O: 252.1262, found: 252.1270.

**tert-butyl 2-(3,3-diphenylpropanamido)ethylcarbamate (30i)**

Off-white solid; ^1^H NMR (300 MHz, DMSO-d6) δ 7.92 (t, *J* = 5.4, 5.4 Hz, 1H), 7.30 – 7.09 (m, 10H), 6.62 (t, *J* = 5.4, 5.4 Hz, 1H), 4.48 (s, 1H), 2.96 (d, *J* = 6.1 Hz, 2H), 2.81 (t, *J* = 8.2, 8.2 Hz, 4H), 1.37 (s, 9H); ^13^C NMR (75 MHz, DMSO) δ 170.20, 155.45, 144.25, 128.26, 127.51, 126.05, 77.61, 46.69, 41.23, 38.62, 38.45, 28.17; HRMS (EI): calcd. for C_22_H_28_N_2_O_3_: 368.2099, found: 368.15638.

**N-(2-aminoethyl)-3,3-diphenylpropanamide (31i)**

White solid; ^1^H NMR (300 MHz, DMSO-d6) δ 8.44 (t, *J* = 5.5, 5.5 Hz, 1H), 8.15 (s, 3H), 7.33 – 7.09 (m, 10H), 4.52 (t, *J* = 8.0, 8.0 Hz, 1H), 3.20 (q, *J* = 6.3, 6.3, 6.2 Hz, 2H), 2.90 (d, *J* = 8.1 Hz, 2H), 22.66 (q, *J* = 5.4, 5.2, 5.2 Hz, 2H); ^13^C NMR (75 MHz, DMSO) δ 170.70, 144.20, 128.29, 127.53, 126.09, 46.55, 41.10, 38.35, 36.12; HRMS (EI): calcd. for C_17_H_20_N_2_O: 268.1575, found: 268.1567.

**3) N-Boc-1,3 propanediamine (Scheme 10)**

**Scheme 10**

**Reagents and condition**: i) HATU, DIPEA, DMF, rt, 4h; ii) 4N HCl in 1,4-dioxane, DCM, 0 ^o^C to rt, 2h.

- 1. **Detailed synthetic route for the development of vancomycin analogue side chain R^2^NHR^1^NH2; where R1= CH_2_CH_2_CH_2_**
     1. **Synthesis of vancomycin analogue substituents for 11w, Vanc-28-30 (naphthoic acid derivatives).**

**Scheme 11.**

**Reagents and conditions:** i) *N*-Boc-1,3 propanediamine, HATU, DIPEA, DMF, rt, 4h; ii) 4N HCl in 1,4-dioxane, DCM, 0 ^o^C to rt, 1h; iii) substituted acetylene, piperidine, Pd(PPh_3_)Cl_2_, 85 ^o^C, 30 min; iv) if R’= TMS (trimethylsilane): TBAF, THF, rt, 30 min.

**Procedure.**

1. **Synthesis of product 41a-d**

**41** was synthesised by following the procedure used for the synthesis of **29** using **39a-c** and **40** as starting materials.

1. **Synthesis of 42a-d**

Compound **42a-d** was synthesised by following the procedure used for the synthesis of **38.**

1. **Synthesis of 43**

Compound **43** was synthesised by following the procedures applied for the synthesis of **9** using **42d**.

**Table S19**

| Entry | Product | R | Yield %  **41a-c** | Yield %  **42a-c** | Vancomycin analogue |
| --- | --- | --- | --- | --- | --- |
| 1 | **42a** | H | 69 | 79 | Vanc-28 |
| 2 | **42b** | OCH_3_ | 65 | 82 | Vanc-29 |
| 3 | **42c** | OH | 62 | 65 | Vanc-30 |
| 4 | **42d** | Br | 85 | 90 | -- |

**Spectral data of 41a-c**

**tert-butyl 3-(2-naphthamido)propylcarbamate (41a)**

White solid; ^1^H NMR (300 MHz, DMSO-d6) δ 8.64 (t, *J* = 5.6, 5.6 Hz, 1H), 8.46 (s, 1H), 8.08 – 7.89 (m, 4H), 7.68 – 7.53 (m, 2H), 6.87 (t, *J* = 5.6, 5.6 Hz, 1H), 3.34 (q, *J* = 6.8, 6.8, 6.7 Hz, 2H), 3.03 (q, *J* = 6.6, 6.6, 6.6 Hz, 2H), 1.70 (p, *J* = 7.0, 7.0, 6.9, 6.9 Hz, 2H), 1.39 (s, 9H); ^13^C NMR (75 MHz, DMSO) δ 166.18, 155.58, 134.02, 132.11, 131.90, 128.74, 127.77, 127.55, 127.45, 127.26, 126.64, 124.09, 77.45, 37.73, 37.05, 29.58, 28.21; HRMS (EI): calcd. for C_19_H_24_N_2_O_3_: 328.1786, found: 328.17682.

**tert-butyl 3-(6-methoxy-2-naphthamido)propylcarbamate (41b)**

White solid; ^1^H NMR (300 MHz, DMSO-d6) δ 8.54 (t, *J* = 5.6, 5.6 Hz, 1H), 8.37 (s, 1H), 7.97 – 7.82 (m, 3H), 7.38 (d, *J* = 2.5 Hz, 1H), 7.23 (dd, *J* = 9.0, 2.5 Hz, 1H), 6.85 (t, *J* = 5.6, 5.6 Hz, 1H), 3.90 (s, 2H), 3.38 – 3.24 (m, 2H), 3.01 (q, *J* = 6.6, 6.6, 6.6 Hz, 2H), 1.67 (p, *J* = 6.9, 6.9, 6.9, 6.9 Hz, 2H), 1.38 (s, 9H); ^13^C NMR (75 MHz, DMSO) δ 166.22, 158.41, 155.57, 135.63, 130.32, 129.59, 127.43, 127.14, 126.55, 124.60, 119.28, 105.79, 77.46, 55.26, 37.72, 36.98, 29.62, 28.21; HRMS (EI): calcd. for C_20_H_26_N_2_O_4_: 358.1892, found: 358.1888.

**tert-butyl 3-(6-hydroxy-2-naphthamido)propylcarbamate (41c)**

White solid; ^1^H NMR (300 MHz, DMSO-d6) δ 10.02 (s, 1H), 8.49 (t, *J* = 5.6, 5.6 Hz, 1H), 8.32 (s, 1H), 7.90 – 7.84 (m, 2H), 7.82 (d, *J* = 1.8 Hz, 1H), 7.17 (s, 1H), 7.14 (d, *J* = 2.4 Hz, 1H), 6.84 (t, *J* = 5.9, 5.9 Hz, 1H), 3.31 (q, *J* = 6.7, 6.7, 6.7 Hz, 2H), 3.01 (q, *J* = 6.7, 6.7, 6.6 Hz, 2H), 1.67 (p, *J* = 6.8, 6.8, 6.8, 6.8 Hz, 2H), 1.38 (s, 9H); ^13^C NMR (75 MHz, DMSO) δ 166.26, 156.67, 155.53, 135.86, 130.45, 128.64, 127.21, 126.57, 125.80, 124.27, 119.27, 108.49, 77.41, 59.67, 38.14, 36.89, 29.60, 28.16; HRMS (EI): calcd. for C_19_H_24_N_2_O_4_ : 344.1736, found: 344.1721.

***tert*-Butyl 3-(6-bromo-2-naphthamido)propylcarbamate (41d).**

White solid; ^1^H NMR (300 MHz, CDCl_3_): δ 8.36 (s, 1H), 8.03 (s,1H), 7.95 (d, *J* = 8.4 Hz,1H), 7.80 (d, *J* = 8.5 Hz, 2H), 7.61-7.53 (m, 2H), 4.93 (t, *J* = 5.7 Hz, 1H), 3.56 (q, *J* = 6.0 Hz, 6.0 Hz, 2H), 3.29 (q, *J* = 6.1 Hz, 6.1Hz, 2H), 1.79-1.71 (m, 2H), 1.46 (s, 9H); ^13^C NMR (75 MHz, CDCl_3_): δ 167.12, 157.10, 135.60, 132.20, 131.10, 130.58, 130.06, 129.79, 127.52, 127.40, 124.79, 121.72, 79.68, 37.07, 36.17, 30.21, 28.41; HRMS (EI): calcd. for C_19_H_23_N_2_O_3_Br: 406.0892, found: 406.0932.

**Spectral data of 42a-d**

**N-(3-aminopropyl)-2-naphthamide (42a)**

Off-white solid; ^1^H NMR (300 MHz, DMSO-d6) δ 9.02 (t, *J* = 5.7, 5.7 Hz, 1H), 8.55 (s, 1H), 8.21 (s, 3H), 8.07-7.93 (d, *J* = 10.0 Hz, 4H), 7.67 – 7.56 (m, 2H), 3.45 – 3.38 (m, 2H), 3.02 – 2.78 (m, 2H), 2.02 – 1.81 (m, 2H); ^13^C NMR (75 MHz, DMSO) δ 166.38, 134.08, 132.08, 131.54, 128.78, 127.79, 127.56, 127.53, 127.50, 126.66, 124.11, 36.66, 36.27, 27.21; HRMS (EI): calcd. for C_14_H_16_N_2_O: 228.1262, found: 228.1253.

**N-(3-aminopropyl)-6-methoxy-2-naphthamide (42b)**

White solid; ^1^H NMR (300 MHz, DMSO-d6) δ 8.92 (t, *J* = 5.7, 5.7 Hz, 5H), 8.46 (s, 5H), 8.18 (s, 3H), 8.02 – 7.88 (m, 3H), 7.39 (d, *J* = 2.5 Hz, 1H), 7.24 (dd, *J* = 8.9, 2.5 Hz, 1H), 3.91 (s, 3H), 3.41 (d, *J* = 5.2 Hz, 2H), 2.95 – 2.81 (m, 2H), 1.90 (p, *J* = 6.7, 6.7, 6.7, 6.7 Hz, 2H); ^13^C NMR (75 MHz, DMSO) δ 166.46, 158.46, 135.71, 130.36, 129.23, 127.42, 127.38, 126.56, 124.62, 119.29, 105.80, 55.28, 36.65, 36.18, 27.26; HR MS (EI): calcd. for C_15_H_18_N_2_O_2_: 258.1368, found: 258.1361.

**N-(3-aminopropyl)-6-hydroxy-2-naphthamide (42c)**

White solid; ^1^H NMR (300 MHz, DMSO-d6) δ 8.92 (t, *J* = 5.7, 5.7 Hz, 5H), 8.46 (s, 5H), 8.18 (s, 3H), 8.02 – 7.88 (m, 3H), 7.39 (d, *J* = 2.5 Hz, 1H), 7.24 (dd, *J* = 8.9, 2.5 Hz, 1H), 3.91 (s, 3H), 3.41 (d, *J* = 5.2 Hz, 2H), 2.95 – 2.81 (m, 2H), 1.90 (p, *J* = 6.7, 6.7, 6.7, 6.7 Hz, 2H); ^13^C NMR (75 MHz, DMSO) δ 166.46, 158.46, 135.71, 130.36, 129.23, 127.42, 127.38, 126.56, 124.62, 119.29, 105.80, 55.28, 36.65, 36.18, 27.26; ; HR MS (EI): calcd. for C_15_H_18_N_2_O_2_: 258.1368, found: 258.1361.

***N*-(3-Aminopropyl)-6-bromo-2-naphthamide (42d)**

White solid; ^1^H NMR (300 MHz, D_2_O): δ 7.80 (s, 1H), 7.67 (s,1H), 7.44-7.20 (m, 4H), 3.36 (t, *J* = 6.7 Hz, 2H), 2.98 (t, *J* = 6.7 Hz, 2H), 1.94-1.85 (m, 2H); ^13^C NMR (75 MHz, D_2_O):169.6, 134.9, 130.3, 130.0, 129.9, 129.6, 129.2, 127.3, 127.1, 123.7, 121.5, 37.1, 36.7, 26.8; HRMS (EI): calcd. for C_14_H_15_N_2_OBr: 306.0368, found: 306.0358.

***N*-(3-Aminopropyl)-6-ethynyl-2-naphthamide (43)**

White solid, yield 50%; ^1^H NMR (300 MHz,DMSO-d_6_): δ 8.98 (t, *J* = 5.5 Hz, 1H), 8.46 (s, 1H), 8.44 (s, 1H), 8.14 (s, 1H), 8.01-7.97 (m, 2H), 7.56 (d, *J* = 8.4 Hz, 1H), 4.35 (s, 1H), 3.35 (t, *J* = 6.5 Hz, 2H), 2.81 (t, *J* = 6.9 Hz, 2H), 2.48-2.47 (t, *J* = 2 Hz, 2H), 1.84-1.76 (m, 2H); ^13^C NMR (75 MHz, DMSO-d_6_): δ 166.16, 133.54, 132.66, 131.69, 131.41, 129.30, 128.85, 127.77, 127.27, 124.99, 120.56, 83.50, 81.99, 36.72, 36.48, 27.86.; HRMS (EI): calcd. for C_16_H_16_N_2_O: 252.1263, found: 252.1285.

- - 1. **Synthesis of naphthoic acid-derived vancomycin analogue substituents where R= sulphonamide (Vanc-63, 65 & 66 )**

**Scheme 12.**

**Reagents and condition**: i) pyridine, rt, 5-6h; ii) *N*-Boc-1,3-propanediamine, HATU, DIPEA, DMF, rt, 4h; iii) 4N HCl in 1,4-dioxane, DCM, 0 ^o^C to rt, 2h.

**Procedure**

1. Synthesis of **44**

Compound **44** was synthesised by following the procedure used for the synthesis of **12,** using **11** and **39d** as starting materials (Table S20).

1. Synthesis of **45** and **46**

Compounds **45** and **46** were synthesised by following the procedures used for the synthesis of **41** and **42,** respectively.

**Table S20**

| Entry | Product | R | Yield %  **44a-c** | Yield %  **45a-c** | Yield %  **46a-c** | Vancomycin analogue |
| --- | --- | --- | --- | --- | --- | --- |
| 1 | **46a** | H | 60 | 75 | 79 | Vanc-63 |
| 2 | **46b** | CH_3_ | 55 | 75 | 81 | Vanc-65 |
| 3 | **46c** | Isopropyl | 50 | 70 | 78 | Vanc-66 |

**Spectral data of 44a-c**

**6-(phenylsulfonamido)-2-naphthoic acid (44a)**

Light-brown solid; ^1^H NMR (300 MHz, DMSO-d6) δ 12.96 (s, 1H), 10.83 (s, 1H), 8.47 (s, 1H), 7.99 (d, *J* = 8.9 Hz, 1H), 7.90-7.83 (m, 4H), 7.68 – 7.48 (m, 4H), 7.38 (dd, *J* = 8.9, 2.1 Hz, 1H); ^13^C NMR (75 MHz, DMSO) δ 167.26, 139.28, 137.56, 135.37, 133.06, 130.68, 130.22, 129.31, 128.79, 127.41, 126.92, 126.67, 125.89, 120.43, 114.62; HRMS (EI): calcd. for C_17_H_13_NO_4_S: 327.0565, found: 327.0549.

**6-(4-methylphenylsulfonamido)-2-naphthoic acid (44b)**

White solid; ^1^H NMR (300 MHz, DMSO-d6) δ 13.00 (s, 1H), 10.75 (s, 1H), 8.48 (s, 1H), 8.00 (d, *J* = 9.0 Hz, 1H), 7.95 – 7.83 (m, 2H), 7.76 (d, *J* = 8.3 Hz, 2H), 7.65 (d, *J* = 1.9 Hz, 1H), 7.39 (dd, *J* = 8.9, 2.2 Hz, 1H), 7.34 (d, *J* = 8.0 Hz, 2H), 2.30 (s, 3H); ^13^C NMR (75 MHz, DMSO) δ 167.27, 143.47, 137.70, 136.41, 135.38, 130.65, 130.21, 129.73, 128.71, 127.38, 126.85, 126.74, 125.87, 120.33, 114.35, 20.87; HRMS (EI): calcd. for C_18_H_15_NO_4_S: 341.0721, found: 341.07212.

**6-(4-isopropylphenylsulfonamido)-2-naphthoic acid (44c)**

White solid; ^1^H NMR (300 MHz, DMSO-d6) δ 12.96 (s, 1H), 10.77 (s, 1H), 8.47 (s, 5H), 7.99 (d, *J* = 9.0 Hz, 1H), 7.91 (dd, *J* = 8.6, 1.5 Hz, 1H), 7.86-7.78 (m, 3H), 7.66 (d, *J* = 1.8 Hz, 1H), 7.43 – 7.36 (m, 3H), 2.86 (hept, *J* = 6.8, 6.8, 6.8, 6.8, 6.8, 6.8 Hz, 1H), 1.10 (d, *J* = 6.9 Hz, 6H); ^13^C NMR (75 MHz, DMSO) δ 167.27, 153.82, 137.74, 136.92, 135.42, 130.69, 130.23, 128.68, 127.37, 127.24, 126.83, 125.90, 120.20, 114.10, 99.48, 33.23, 23.23; HRMS (EI): calcd. for C_20_H_19_NO_4_S: 369.1034, found: 370.9960 (H+ salt).

**Spectral data of 45a-c**

**tert-butyl 3-(6-(phenylsulfonamido)-2-naphthamido)propylcarbamate (45a)**

White solid, ^1^H NMR (300 MHz, DMSO-d6) δ 10.71 (s, 1H), 8.54 (t, *J* = 5.6, 5.6 Hz, 1H), 8.31 (s, 1H), 7.93 – 7.82 (m, 5H), 7.64 – 7.50 (m, 4H), 7.37 (dd, *J* = 8.8, 2.2 Hz, 1H), 6.83 (t, *J* = 5.4, 5.4 Hz, 1H), 3.29 (q, *J* = 6.7, 6.7, 6.7 Hz, 2H), 2.99 (q, *J* = 6.6, 6.6, 6.6 Hz, 2H), 1.7-1.60 (m, 2H), 1.37 (s, 9H); ^13^C NMR (75 MHz, DMSO) δ 165.97, 155.55, 139.32, 136.79, 134.40, 133.01, 130.85, 130.16, 129.28, 128.88, 127.12, 127.05, 126.65, 124.81, 120.58, 114.98, 77.45, 37.69, 36.99, 29.55, 28.20.

**tert-butyl 3-(6-(4-methylphenylsulfonamido)-2-naphthamido)propylcarbamate (45b)**

White solid; ^1^H NMR (300 MHz, DMSO-d6) δ 10.64 (s, 1H), 8.54 (t, *J* = 5.6, 5.6 Hz, 1H), 8.31 (s, 1H), 7.92 – 7.81 (m, 3H), 7.73 (d, *J* = 8.3 Hz, 2H), 7.61 (d, *J* = 2.0 Hz, 1H), 7.39 – 7.29 (m, 3H), 6.83 (t, *J* = 5.6, 5.6 Hz, 1H), 3.29 (q, *J* = 6.7, 6.7, 6.6 Hz, 2H), 2.99 (q, *J* = 6.6, 6.6, 6.6 Hz, 2H), 2.29 (s, 3H), 1.65 (p, *J* = 6.8, 6.8, 6.2, 6.2 Hz, 2H), 1.37 (s, 9H); ^13^C NMR (75 MHz, DMSO) δ 165.99, 155.56, 143.42, 136.94, 136.46, 134.43, 130.77, 130.13, 129.70, 128.80, 127.09, 127.04, 126.71, 124.79, 120.48, 114.71, 77.46, 37.68, 36.99, 29.55, 28.19, 20.86.

**tert-butyl 3-(6-(4-isopropylphenylsulfonamido)-2-naphthamido)propylcarbamate (45c)**

Yellow solid; ^1^H NMR (300 MHz, DMSO-d6) δ 10.69 (s, 1H), 8.57 – 8.51 (m, 1H), 8.32 (s, 1H), 7.91 (d, *J* = 9.1 Hz, 1H), 7.87 – 7.81 (m, 2H), 7.78 (d, *J* = 8.4 Hz, 2H), 7.64 (d, *J* = 2.0 Hz, 1H), 7.44 – 7.36 (m, 3H), 6.84 (t, *J* = 5.5, 5.5 Hz, 1H), 3.29 (q, *J* = 6.7, 6.7, 6.7 Hz, 2H), 2.98 (p, *J* = 6.1, 6.1, 5.6, 5.6 Hz, 2H), 2.94 – 2.84 (m, 1H), 1.65 (p, *J* = 6.9, 6.9, 6.8, 6.8 Hz, 2H), 1.37 (s, 8H), 1.14 (d, *J*= 6.92 Hz, 6H); ^13^C NMR (75 MHz, DMSO) δ 165.96, 155.55, 153.78, 151.07, 136.96, 134.45, 130.75, 130.17, 128.77, 127.22, 127.09, 126.81, 124.81, 120.68, 120.35, 114.46, 77.44, 37.68, 36.99, 33.24, 29.55, 28.20, 23.27; HRMS (EI): calcd. for C_28_H_35_N_3_O_5_S: 525.2297, found: 425.17614 (-Boc).

**Spectral data of 46a-c**

**N-(3-aminopropyl)-6-(phenylsulfonamido)-2-naphthamide (46a)**

Light-brown solid; ^1^H NMR (300 MHz, DMSO-d6) δ 10.84 (s, 1H), 8.91 (t, *J* = 5.7, 5.7 Hz, 1H), 8.40 (s, 1H), 8.24 – 8.05 (m, 3H), 7.95 – 7.81 (m, 5H), 7.67 – 7.50 (m, 4H), 7.41 (dd, *J* = 8.9, 2.1 Hz, 1H), 3.38 (q, *J* = 7.0, 7.0, 6.9 Hz, 2H), 2.85 (q, *J* = 6.6, 6.6, 6.6 Hz, 2H), 1.87 (p, *J* = 6.7, 6.7, 6.7, 6.7 Hz, 2H); ^13^C NMR (75 MHz, DMSO) δ 166.22, 139.36, 136.93, 134.46, 132.99, 130.49, 130.14, 129.26, 128.84, 127.27, 127.12, 126.67, 124.81, 120.64, 114.99, 36.63, 36.21, 27.20; HRMS (EI): calcd. for C_20_H_21_N_3_O_3_S: 383.1303, found: 383.12776.

**N-(3-aminopropyl)-6-(4-methylphenylsulfonamido)-2-naphthamide (46b)**

White solid; ^1^H NMR (300 MHz, DMSO-d6) δ 10.77 (s, 1H), 8.90 (t, *J* = 5.6, 5.6 Hz, 1H), 8.39 (s, 1H), 8.11 (s, 3H), 7.95 – 7.80 (m, 3H), 7.74 (d, *J* = 8.3 Hz, 2H), 7.66 – 7.61 (m, 1H), 7.39 (dd, *J* = 8.8, 2.1 Hz, 1H), 7.33 (d, *J* = 8.1 Hz, 2H), 3.42-3.34 (m, 2H), 2.84 (s, 2H), 2.29 (s, 3H), 1.86 (p, *J* = 6.4, 6.4, 6.2, 6.2 Hz, 2H); ^13^C NMR (75 MHz, DMSO) δ 167.26, 143.82, 136.90, 135.99, 134.52, 130.28, 130.16, 129.72, 128.72, 127.30, 127.14, 126.72, 124.66, 120.49, 114.72, 113.23, 36.85, 36.28, 27.15, 20.77; HRMS (EI): calcd. for C_21_H_23_N_3_O_3_S: 397.1460, found: 397.14714.

**N-(3-aminopropyl)-6-(4-isopropylphenylsulfonamido)-2-naphthamide (46c)**

Yellow solid; ^1^H NMR (300 MHz, DMSO-d6) δ 10.82 (s, 1H), 8.92 (t, *J* = 5.6, 5.6 Hz, 1H), 8.41 (s, 1H), 8.15 (s, 3H), 7.96 – 7.78 (m, 5H), 7.67 (s, 1H), 7.45 (d, *J* = 2.2 Hz, 1H), 7.41 (d, *J* = 8.1 Hz, 2H), 3.39 (q, *J* = 5.4, 4.9, 4.9 Hz, 2H), 2.94-2.81 (m, 3H), 1.88 (p, *J* = 6.5, 6.5, 6.1, 6.1 Hz, 2H), 1.13 (d, *J* = 6.9 Hz, 6H); ^13^C NMR (75 MHz, DMSO) δ 166.21, 153.73, 151.00, 137.11, 136.99, 134.52, 130.38, 130.15, 128.73, 127.19, 126.84, 124.82, 120.62, 120.40, 114.45, 38.19, 36.63, 33.23, 27.20, 23.27; HRMS (EI): calcd. for C_23_H_27_N_3_O_3_S: 425.1773, found: 425.17961.

- 1. **Synthesis of vancomycin analogues (phenyl and biphenyl carboxylic acid derivatives; 11v, Vanc-33-35)**

**Scheme 13.**

**Reagents and conditions:** i) *N*-Boc-1,3-propanediamine, HATU, DIPEA, DMF, rt, 4h; ii) 4N HCl in 1,4-dioxane, DCM, 0 ^o^C to rt, 2h; iii) For R= H; trimethylsilylacetylene, piperidine, Pd(PPh_3_)Cl_2_, 85 ^o^C, 30 min; iv) TBAF, THF, rt, 30 min.

**Procedure**

Compounds **48**, **49** and **50** were synthesised by following the procedure used for the synthesis of compound **41**, **42** and **43,** respectively.

**Table S21**

| Entry | Product | R | Yield %  **48a-i** | Yield %  **49a-i** | Vancomycin analogue |
| --- | --- | --- | --- | --- | --- |
| 1 | **49a** | 4-heptyl | 95 | 94 | Vanc-31 |
| 2 | **49b** | 4-CF_3_ | 98 | 95 | Vanc-32 |
| 3 | **49c** | Ph | 85 | 96 | Vanc-33 |
| 4 | **49d** | 4-F- Ph | 82 | 85 | Vanc-34 |
| 5 | **49e** | Biphenyl | 45 | 89 | Vanc-35 |
| 6 | **49f** | 4-Pentyl | 97 | 92 | Vanc-49 |
| 7 | **49g** | 4-butoxy | 91 | 93 | Vanc-50 |
| 8 | **49h** | 4-*t*-Bu | 88 | 91 | Vanc-73 |
| 9 | **49i** | 4-Br-Ph | 85 | 85 |  |

**Spectral data of 48a-i**

**tert-butyl 3-(4-heptylbenzamido)propylcarbamate (48a)**

White solid; ^1^H NMR (300 MHz, DMSO-d6) δ 8.33 (t, *J* = 5.6, 5.6 Hz, 1H), 7.75 (d, *J* = 8.3 Hz, 2H), 7.26 (d, *J* = 8.3 Hz, 2H), 6.80 (t, *J* = 5.4, 5.4 Hz, 1H), 3.24 (q, *J* = 6.7, 6.7, 6.7 Hz, 2H), 2.97 (q, *J* = 6.6, 6.6, 6.5 Hz, 2H), 2.61 (t, *J* = 7.6, 7.6 Hz, 2H), 1.66-1.52 (m, 4H), 1.37 (s, 9H), 1.28 – 1.23 (m, 8H), 0.85 (t, *J* = 6.7, 6.7, Hz 3H); ^13^C NMR (75 MHz, DMSO) δ 166.02, 155.45, 145.60, 132.01, 128.04, 127.07, 77.42, 37.67, 36.82, 34.88, 31.19, 30.69, 29.59, 28.52, 28.46, 28.20, 22.02, 13.88; HRMS (EI): calcd. for C_22_H_36_N_2_O_3_: 376.2725, found: 376.27088.

**tert-butyl 3-(4-(trifluoromethyl)benzamido)propylcarbamate (48b)**

White solid; ^1^H NMR (300 MHz, DMSO-d6) δ 8.69 (t, *J* = 5.5, 5.5 Hz, 1H), 8.05 (d, *J* = 8.1 Hz, 2H), 7.85 (d, *J* = 8.2 Hz, 2H), 6.84 (t, *J* = 5.5, 5.5 Hz, 1H), 3.30 (q, *J* = 6.7, 6.7, 6.7 Hz, 2H), 3.01 (q, *J* = 6.6, 6.6, 6.6 Hz, 2H), 1.67 (p, *J* = 6.9, 6.9, 6.6, 6.6 Hz, 2H), 1.39 (s, 9H); ^13^C NMR (75 MHz, DMSO) δ 164.90, 155.54, 138.25, 131.18, 130.76, 127.97, 125.70, 125.23, 125.18, 122.09, 77.54, 37.64, 37.08, 29.38, 28.16; HRMS (EI): calcd. for C_16_H_21_F_3_N_2_O_3_: 346.1504, found: 346.1485.

**tert-butyl 3-biphenyl-4-ylcarboxamidopropylcarbamate (48c)**

White solid; ^1^H NMR (300 MHz, DMSO-d6) δ 8.50 (t, *J* = 5.6, 5.6 Hz, 1H), 7.94 (d, *J* = 8.5 Hz, 2H), 7.82 – 7.69 (m, 4H), 7.52 – 7.47 (m, 2H), 7.44 – 7.38 (m, 1H), 6.84 (t, *J* = 5.6, 5.6 Hz, 1H), 3.29 (q, *J* = 6.7, 6.7, 6.7 Hz, 2H), 3.00 (q, *J* = 6.6, 6.6, 6.6 Hz, 2H), 1.65 (p, *J* = 7.1, 7.1, 6.9, 6.9 Hz, 2H), 1.39 (s, 9H); ^13^C NMR (75 MHz, DMSO) δ 165.75, 155.51, 142.55, 139.15, 133.32, 128.97, 127.96, 127.75, 126.80, 126.42, 77.45, 37.68, 36.92, 29.58, 28.21; HRMS (EI): calcd. for C_8_H_10_N_2_O_2_: 354.1943, found: 354.19445.

**tert-butyl 3-(4'-fluorobiphenyl-4-ylcarboxamido)propylcarbamate (48d)**

White solid; ^1^H NMR (300 MHz, DMSO-d6) δ 8.51 (t, *J* = 5.6, 5.6 Hz, 1H), 7.94 (d, *J* = 8.5 Hz, 2H), 7.84 – 7.71 (m, 4H), 7.32 (t, *J* = 8.9, 8.9 Hz, 2H), 6.85 (t, *J* = 5.8, 5.8 Hz, 1H), 3.29 (q, *J* = 6.6, 6.6, 6.6 Hz, 2H), 3.00 (q, *J* = 6.7, 6.7, 6.6 Hz, 2H), 1.66 (p, *J* = 7.0, 7.0, 7.0, 7.0 Hz, 2H), 1.39 (s, 9H); ^13^C NMR (75 MHz, DMSO) δ 165.70, 163.78, 160.53, 155.58, 141.50, 135.64, 133.29, 128.92, 127.78, 115.93, 115.65, 77.46, 37.69, 36.94, 29.59, 28.22; HRMS (EI): calcd. for C_21_H_25_FN_2_O_3_: 372.1849, found: 372.18441

**N-(3-aminopropyl)terphenyl-4-carboxamide (48e)**

White solid; ESI-MS calcd. for C_27_H_30_FN_2_O_3_: 430.225, found 453.6 [M+Na]^+^; used directly fot the next step.

**tert-butyl 3-(4-pentylbenzamido)propylcarbamate (48f)**

White solid; ^1^H NMR (300 MHz, DMSO-d6) δ 8.36 (t, *J* = 5.6, 5.6 Hz, 1H), 7.76 (d, *J* = 8.2 Hz, 2H), 7.26 (d, *J* = 8.2 Hz, 2H), 6.81 (d, *J* = 5.6 Hz, 1H), 3.31 – 3.20 (m, 2H), 2.98 (q, *J* = 6.6, 6.6, 6.6 Hz, 2H), 2.61 (t, *J* = 7.6, 7.6 Hz, 2H), 1.60 (dp, *J* = 15.0, 7.3, 7.3, 7.1, 7.1 Hz, 4H), 1.38 (s, 9H), 1.27 (dq, *J* = 8.2, 5.0, 5.0, 4.3 Hz, 4H), 0.90 – 0.80 (m, 3H); ^13^C NMR (75 MHz, DMSO) δ 155.54, 145.59, 132.00, 128.03, 127.08, 77.40, 37.65, 36.81, 34.85, 30.78, 30.37, 29.59, 28.18, 21.89, 13.84; HRMS (EI): calcd.for C_20_H_32_N_2_O_3_: 348.2412, found: 348.2418.

**tert-butyl 3-(4-butoxybenzamido)propylcarbamate (48g)**

White solid; ^1^H NMR (300 MHz, DMSO-d6) δ 8.27 (s, 1H), 7.79 (d, *J* = 8.4 Hz, 2H), 6.97 (d, *J* = 8.4 Hz, 2H), 6.81 (s, 1H), 4.01 (t, *J* = 6.2, 6.2 Hz, 2H), 3.23 (q, *J* = 6.6, 6.6, 6.6 Hz, 2H), 2.96 (q, *J* = 6.7, 6.6, 6.6 Hz, 2H), 1.65 (dq, *J* = 20.1, 6.7, 6.7, 6.7 Hz, 4H), 1.50 – 1.39 (m, 2H), 1.37 (s, 9H), 0.93 (t, *J* = 7.2, 7.2 Hz, 3H); ^13^C NMR (75 MHz, DMSO) δ 165.59, 160.84, 155.54, 128.83, 126.56, 113.78, 77.42, 67.26, 37.66, 36.77, 30.61, 29.65, 28.20, 18.66, 13.63; HRMS (EI): calcd. for C_19_H_30_N_2_O_4_: 350.2205, found: 350.2184.

**tert-butyl 3-(4-tert-butylbenzamido)propylcarbamate (48h)**

White solid; ^1^H NMR (300 MHz, DMSO-d6) δ 8.35 (t, *J* = 5.7, 5.7 Hz, 1H), 7.76 (d, *J* = 8.5 Hz, 2H), 7.47 (d, *J* = 8.5 Hz, 2H), 6.81 (t, *J* = 5.7, 5.7 Hz, 1H), 3.24 (q, *J* = 6.7, 6.7, 6.7 Hz, 2H), 2.96 (q, *J* = 6.6, 6.6, 6.6 Hz, 2H), 1.61 (p, *J* = 6.8, 6.8, 6.8, 6.8 Hz, 2H), 1.37 (s, 9H), 1.29 (s, 9H); ^13^C NMR (75 MHz, DMSO) δ 166.48, 156.03, 154.24, 132.29, 127.39, 125.44, 77.93, 38.12, 37.29, 35.03, 31.41, 30.11, 28.71; HRMS (EI): calcd. for C_19_H_30_N_2_O_3_: 334.2256, found: 334.22258.

***tert*-Butyl 3-(4'-bromobiphenyl-4-ylcarboxamido)propylcarbamate (48i).**

White solid; ^1^H NMR (300 MHz, CDCl_3_): δ 7.93 (d, *J* = 8.5 Hz, 2H), 7.60 (dd, *J* = 8.5 Hz, 8.5 Hz, 4H), 7.47 (d, *J* = 8.5 Hz, 2H), 7.38 (bs, 1H), 4.92 (bs, 1H), 3.53 (dd, *J* = 6.0 Hz, 6.0 Hz, 2H), 3.27 (dd, *J* = 6.2 Hz, 6.2 Hz, 2H),1.6-1.67 (m, 2H), 1.46 (s, 9H); ^13^C NMR (75 MHz, CDCl_3_): δ 167.02, 157.07, 142.78, 139.02, 133.59, 132.00, 128.74, 127.67, 126.96, 122.25, 79.62, 37.01, 36.09, 30.23, 28.40; HRMS (EI): calcd. for C_21_H_25_BrN_2_O_3_: 432.1049, found: 432.1058.

**Spectral data of 49a-i**

**N-(3-aminopropyl)-4-heptylbenzamide (49a)**

White solid; ^1^H NMR (300 MHz, DMSO-d6) δ 8.73 (t, *J* = 5.8, 5.8 Hz, 1H), 8.13 (s, 3H), 7.82 (d, *J* = 8.2 Hz, 2H), 7.27 (d, *J* = 8.2 Hz, 2H), 3.33 (q, *J* = 6.4, 6.4, 6.3 Hz, 2H), 2.83 (t, *J* = 7.2, 7.2 Hz, 2H), 2.61 (t, *J* = 7.6, 7.6 Hz, 2H), 1.91 – 1.76 (m, 2H), 1.58 (q, *J* = 7.2, 7.2, 6.9 Hz, 2H), 1.32 – 1.19 (m, 8H), 0.85 (t, *J* = 6.6, 6.6, Hz 3H); ^13^C NMR (75 MHz, DMSO) δ 166.27, 145.79, 131.65, 128.06, 127.20, 36.58, 36.01, 34.88, 31.19, 30.69, 28.53, 28.46, 27.20, 22.03, 13.90; HRMS (EI): calcd. for C_17_H_28_N_2_O: 276.2201, found: 276.21922.

**N-(3-aminopropyl)-4-(trifluoromethyl)benzamide (49b)**

White solid; ^1^H NMR (300 MHz, DMSO-d6) δ 9.10 (t, *J* = 5.7, 5.7 Hz, 1H), 8.16 (s, 3H), 8.12 (d, *J* = 8.2 Hz, 2H), 7.86 (d, *J* = 8.2 Hz, 2H), 3.38 (q, *J* = 6.5, 6.5, 6.5 Hz, 2H), 2.86 (t, *J* = 6.6 Hz, 6.6 Hz, 2H), 1.88 (p, *J* = 6.8, 6.8, 6.7, 6.7 Hz, 2H); ^13^C NMR (75 MHz, DMSO) δ 165.10, 137.94, 131.26, 130.84, 128.12, 125.69, 125.25, 125.20, 122.08, 36.62, 36.32, 27.02; HRMS (EI): calcd. for C_11_H_13_F_3_N_2_O: 246.0980, found: 246.09685.

**N-(3-aminopropyl)biphenyl-4-carboxamide (49c)**

Off-white solid; ^1^H NMR (300 MHz, DMSO-d6) δ 8.90 (t, *J* = 5.8, 5.8 Hz, 1H), 8.17 (s, 3H), 8.02 (d, *J* = 8.5 Hz, 2H), 7.83 – 7.69 (m, 4H), 7.54 – 7.46 (m, 2H), 7.45 – 7.38 (m, 1H), 3.43 – 3.32 (m, 2H), 2.93 – 2.81 (m, 2H), 1.88 (p, *J* = 6.7, 6.7, 6.3, 6.3 Hz, 2H); ^13^C NMR (75 MHz, DMSO) δ 165.99, 142.68, 139.11, 132.99, 128.97, 127.98, 127.89, 126.80, 126.43, 36.62, 36.14, 27.18; HRMS (EI): calcd. for C_16_H_18_N_2_O: 254.1419, found: 254.14183.

**N-(3-aminopropyl)-4'-fluorobiphenyl-4-carboxamide (49d)**

White solid; ^1^H NMR (300 MHz, DMSO-d6) δ 8.92 (t, *J* = 5.7, 5.7 Hz, 1H), 8.19 (s, 3H), 8.02 (d, *J* = 8.5 Hz, 2H), 7.85 – 7.71 (m, 4H), 7.33 (t, *J* = 8.9, 8.9 Hz, 2H), 3.46 – 3.33 (m, 2H), 2.87 (h, *J* = 6.9, 6.9, 5.9, 5.9, 5.9 Hz, 2H), 1.89 (p, *J* = 6.7, 6.7, 6.7, 6.7 Hz, 2H); ^13^C NMR (75 MHz, DMSO) δ 165.87, 163.72, 160.52, 141.55, 135.50, 132.90, 128.88, 128.77, 127.85, 126.33, 115.89, 115.60, 36.57, 36.09, 27.13; HRMS (EI): calcd. for C_16_H_17_FN_2_O: 272.1324, found: 272.13289.

**N-(3-aminopropyl)terphenyl-4-carboxamide (49e)**

White solid; ^1^H NMR (300 MHz, DMSO-d6) δ 8.86 – 8.72 (m, 3H), 8.66 (d, *J* = 8.4 Hz, 1H), 8.07 – 7.94 (m, 3H), 7.91 – 7.69 (m, 7H), 7.65 – 7.36 (m, 4H), 6.77 (s, 1H), 3.47 – 3.21 (m, 2H), 2.85 (s, 2H), 1.86 (s, 2H); ^13^C NMR (75 MHz, DMSO) δ 166.06, 152.01, 139.10, 134.46, 129.39, 128.97, 127.90, 127.64, 127.33, 127.22, 126.57, 126.32, 121.47, 36.71, 36.18, 27.30; HRMS (EI): calcd. for C_22_H_22_N_2_O: 330.1732, found: ESI-MS: 331.01

**N-(3-aminopropyl)-4-pentylbenzamide (49f)**

White solid; ^1^H NMR (300 MHz, DMSO-d6) δ 8.71 (t, *J* = 5.7, 5.7 Hz, 1H), 8.09 (s, 3H), 7.81 (d, *J* = 8.2 Hz, 2H), 7.27 (d, *J* = 8.2 Hz, 2H), 3.33 (dd, *J* = 13.2, 7.1 Hz, 2H), 2.82 (t, *J* = 7.4, 7.4 Hz, 2H), 2.66 – 2.56 (m, 2H), 1.83 (p, *J* = 6.7, 6.7, 6.7, 6.7 Hz, 2H), 1.58 (p, *J* = 7.5, 7.5, 7.3, 7.3 Hz, 2H), 1.28 (td, *J* = 9.1, 8.1, 5.6 Hz, 4H), 0.86 (t, *J* = 6.9, 6.9 Hz, 3H); ^13^C NMR (75 MHz, DMSO) δ 166.29, 145.81, 131.67, 128.09, 127.20, 36.60, 36.02, 34.84, 30.78, 30.35, 27.23, 21.88, 13.87; HRMS (EI): calcd. for C_15_H_24_N_2_O: 248.1888, found: 248.1884.

**N-(3-aminopropyl)-4-butoxybenzamide (49g)**

White solid ^1^H NMR (300 MHz, DMSO-d6) δ 8.66 (t, *J* = 4.3, 4.3 Hz, 1H), 8.12 (s, 3H), 7.86 (d, *J* = 8.5 Hz, 2H), 6.98 (d, *J* = 8.7 Hz, 2H), 4.02 (t, *J* = 6.5, 6.5 Hz, 2H), 3.32 (q, *J* = 6.4, 6.4, 6.3 Hz, 1H), 2.82 (h, *J* = 5.4, 5.4, 5.2, 5.2, 5.2 Hz, 2H), 1.76 (dp, *J* = 36.9, 6.6, 6.6, 6.5, 6.5 Hz, 4H), 1.50 – 1.37 (m, 2H), 0.93 (t, *J* = 7.4, 7.4 Hz, 3H); 13C NMR (75 MHz, DMSO) δ 165.87, 160.96, 128.99, 126.21, 113.82, 67.29, 36.59, 35.96, 30.60, 27.26, 18.66, 13.64; HRMS (EI): calcd. for C_14_H_22_N_2_O_2_: 250.1681, found: 250.1168.

**N-(3-aminopropyl)-4-tert-butylbenzamide (49h)**

White solid; ^1^H NMR (300 MHz, DMSO-d6) δ 8.72 (t, *J* = 5.7, 5.7 Hz, 1H), 8.08 (s, 3H), 7.83 (d, *J* = 8.5 Hz, 2H), 7.47 (d, *J* = 8.5 Hz, 2H), 3.33 (q, *J* = 6.4, 6.4, 6.3 Hz, 2H), 2.82 (h, *J* = 5.6, 5.6, 5.4, 5.4, 5.4 Hz, 2H), 1.83 (p, *J* = 6.7, 6.7, 6.7, 6.7 Hz, 2H), 1.30 (s, 9H); ^13^C NMR (75 MHz, DMSO) δ 166.28, 153.92, 131.44, 127.04, 124.95, 36.57, 34.53, 33.97, 30.89, 27.17; HRMS (EI): calcd. for C_14_H_22_N_2_O: 234.1732, found: 234.1733.

***N*-(3-Aminopropyl)-4'-bromobiphenyl-4-carboxamide (49i).**

White solid; ^1^H NMR (300 MHz, DMSO-d_6_): δ 8.15 (bs, 1H), 8.00 (d, *J* = 8.4 Hz, 2H), 7.78 (d, *J* = 8.4 Hz, 2H), 7.70 (m, 4H), 3.38 (m, 2H), 2.84 (m, 2H), 1.88-1.84 (m, 2H); ^13^C NMR (75 MHz, DMSO-d_6_): δ 165.84, 141.38, 138.27, 133.29, 131.88, 128.93, 127.97, 126.40, 121.57, 36.49, 36.06, 27.13.; HRMS (EI): calcd. for C_16_H_17_BrN_2_O: 332.0524, found: 332.0529.

***N*-(3-Aminopropyl)-4'-ethynylbiphenyl-4-carboxamide (50).**

Yellow solid, yield 50%; ^1^H NMR (300 MHz, DMSO-d_6_): δ 8.9 (bs, 1H), 8.5 (bs, NH, 2H), 7.9 (d, *J* = 8.4 Hz, 2H), 7.77 (t, *J* = 8.4 Hz, 4H),7.58 (d, *J* = 8.3 Hz, 2H), 4.3 (s, 1H), 3.18 (t, *J* = 6.2 Hz, 2H), 2.84 (t, *J* = 6.2 Hz, 2H), 1.84 (m, 2H); ^13^C NMR (75 MHz, DMSO-d_6_): δ 165.87, 141.47, 139.44, 133.55, 132.30, 127.93, 127.02, 126.47, 121.28, 83.20, 81.82, 36.43, 27.63, 23.03; HRMS (EI): calcd. for C_18_H_18_N_2_O: 278.1419, found: 278.1430.

- 1. **Detailed synthesis of vancomycin analogue substituents having heterocyclic system (Vanc-24-27, 45, 52).**

**Scheme 14**

**Reagents and conditions:** i) *N*-Boc-1,3-propanediamine, HATU, DIPEA, DMF, rt, 4h; ii) 4N HCl in 1,4-dioxane, DCM, 0 ^o^C to rt, 2h.

**Procedure**

Compounds **52** and **50** were synthesised by following the procedure used for the synthesis of compound **41** and **42,** respectively.

**Table S22**

| Entry | Product | R | X | Y | Yield %  **52a-f** | Yield %  **53a-f** | Vancomycin analogue |
| --- | --- | --- | --- | --- | --- | --- | --- |
| 1 | **53a** | H | CH | NH | 96 | 85 | Vanc-24 |
| 2 | **53b** | H | CH | O | 97 | 84 | Vanc-25 |
| 3 | **53c** | H | CH | S | 95 | 89 | Vanc-26 |
| 4 | **53d** | H | CH=CH | N | 95 | 81 | Vanc-27 |
| 5 | **53e** | 5-F | CH | NH | 92 | 83 | Vanc-45 |
| 6 | **53f** | H | N | NH | 80 | 79 | Vanc-52 |

**Spectral data of 52a-e**

**tert-butyl 3-(1H-indole-2-carboxamido)propylcarbamate (52a)**

White solid; ^1^H NMR (300 MHz, DMSO-d6) δ 11.56 (s, 1H), 8.45 (t, *J* = 5.7, 5.7 Hz, 1H), 7.60 (d, *J* = 7.9 Hz, 1H), 7.42 (d, *J* = 8.1 Hz, 1H), 7.17 (t, *J* = 7.4, 7.4 Hz, 1H), 7.08 (d, *J* = 2.0 Hz, 1H), 7.02 (t, *J* = 7.4, 7.4 Hz, 1H), 6.85 (t, *J* = 5.4, 5.4 Hz, 1H), 3.28 (q, *J* = 6.7, 6.7, 6.7 Hz, 2H), 2.99 (q, *J* = 6.6, 6.6, 6.5 Hz, 2H), 1.69-1.59 (m, 2H), 1.38 (s, 9H); ^13^C NMR (75 MHz, DMSO) δ 161.04, 155.57, 136.31, 131.74, 127.04, 123.12, 121.37, 119.61, 112.22, 102.15, 77.54, 37.68, 36.50, 29.70, 28.21; HRMS (EI): calcd. for C_17_H_23_N_3_O_3_: 317.1739, found: 317.17593.

**tert-butyl 3-(benzofuran-2-carboxamido)propylcarbamate (52b)**

White solid; ^1^H NMR (300 MHz, DMSO-d6) δ 8.72 (t, *J* = 5.8, 5.8 Hz, 1H), 7.78 (d, *J* = 7.3 Hz, 1H), 7.66 (d, *J* = 9.1 Hz, 1H), 7.53 (d, *J* = 0.9 Hz, 1H), 7.47 (ddd, *J* = 8.4, 7.3, 1.3 Hz, 1H), 7.38 – 7.31 (m, 1H), 6.85 (t, *J* = 5.6, 5.6 Hz, 1H), 3.29 (q, *J* = 6.7, 6.7, 6.7 Hz, 2H), 3.00 (q, *J* = 6.6, 6.6, 6.6 Hz, 2H), 1.66 (p, *J* = 6.9, 6.9, 6.8, 6.8 Hz, 2H), 1.38 (s, 9H); ^13^C NMR (75 MHz, CDCl_3_) δ 163.26, 160.80, 159.36, 154.46, 132.38, 131.91, 128.85, 127.90, 116.93, 114.40, 82.70, 42.85, 41.71, 34.74, 33.44; HRMS (EI): calcd. for C_17_H_22_N_2_O_4_: 318.1579, found: 318.1575.

**tert-butyl 3-(benzo[b]thiophene-2-carboxamido)propylcarbamate (52c)**

Off-white solid; ^1^H NMR (300 MHz, DMSO-d6) δ 8.74 (t, *J* = 5.5, 5.5 Hz, 1H), 8.07 (s, 1H), 8.05 – 7.90 (m, 2H), 7.50 – 7.40 (m, 2H), 6.85 (t, *J* = 5.2, 5.2 Hz, 1H), 3.00 (q, *J* = 6.7, 6.7, 6.6 Hz, 2H), 1.66 (p, *J* = 6.7, 6.7, 6.6, 6.6 Hz, 2H), 1.38 (s, 9H); ^13^C NMR (75 MHz, DMSO) δ 161.37, 155.56, 140.07, 139.11, 126.05, 125.04, 124.82, 124.43, 122.73, 77.46, 37.69, 37.06, 29.49, 28.20; HRMS (EI): calcd. for C_17_H_22_N_2_O_3_S: 334.1351, found: 334.13516.

**tert-butyl 3-(quinoline-2-carboxamido)propylcarbamate (52d)**

White solid; ^1^H NMR (300 MHz, DMSO-d6) δ 9.03 (t, *J* = 6.0, 6.0 Hz, 1H), 8.57 (d, *J* = 8.3 Hz, 1H), 8.23 – 8.05 (m, 3H), 7.89 (ddd, *J* = 8.5, 6.9, 1.5 Hz, 1H), 7.73 (ddd, *J* = 8.1, 6.9, 1.3 Hz, 1H), 6.90 (t, *J* = 5.7, 5.7 Hz, 1H), 3.44 – 3.37 (m, 2H), 3.03 (q, *J* = 6.6, 6.6, 6.6 Hz, 2H), 1.71 (p, *J* = 6.8, 6.8, 6.8, 6.8 Hz, 2H), 1.39 (s, 9H); ^13^C NMR (75 MHz, DMSO) δ 163.95, 155.57, 150.14, 145.91, 137.72, 130.39, 129.07, 128.70, 128.01, 127.91, 118.55, 77.41, 37.50, 36.58, 29.65, 28.17; HRMS (EI): calcd. for C_18_H_23_N_3_O_3_: 329.1739, found: 329.17342.

**tert-butyl 3-(5-fluoro-1H-indole-2-carboxamido)propylcarbamate (52e)**

White solid; ^1^H NMR (300 MHz, DMSO-d6) δ 11.69 (s, 1H), 8.51 (t, *J* = 5.6, 5.6 Hz, 1H), 7.44-7.38 (m, 2H), 7.15 – 6.97 (m, 2H), 6.86 (t, *J* = 5.5, 5.5 Hz, 1H), 3.29 (q, *J* = 6.6, 6.6, 6.6 Hz, 2H), 3.00 (q, *J* = 6.5, 6.5, 6.5 Hz, 2H), 1.66 (p, *J* = 6.8, 6.8, 6.8, 6.8 Hz, 2H), 1.38 (s, 9H); ^13^C NMR (75 MHz, DMSO) δ 160.72, 158.61, 155.53, 133.46, 133.04, 127.17, 127.04, 113.44, 113.31, 111.97, 111.62, 105.70, 105.40, 102.21, 77.46, 37.67, 36.53, 29.65, 28.20; HRMS (EI): calcd. for C_17_H_22_FN_3_O_3_: 335.1645, found: 335.1642.

**tert-butyl 3-(1H-benzo[d]imidazole-2-carboxamido)propylcarbamate (52f)**

White solid; ^1^H NMR (300 MHz, DMSO-d6) δ 13.24 (s, 1H), 8.97 (s, 1H), 7.71 (d, J = 6.4 Hz, 1H), 7.53 (d, J = 6.2 Hz, 1H), 7.28 (s, 2H), 6.84 (s, 1H), 3.32 (s, 2H), 2.98 (s, 2H), 1.66 (s, 2H), 1.36 (s, 9H); ^13^C NMR (75 MHz, DMSO) δ 158.70, 155.56, 145.64, 142.45, 134.38, 123.94, 122.42, 119.73, 112.46, 77.43, 37.48, 36.48, 29.47, 28.17; HRMS (EI): calcd. for C_16_H_22_N_4_O_3_: 318.1691, found: 318.1693.

**Spectral data of 53a-e**

**N-(3-aminopropyl)-1H-indole-2-carboxamide (53a)**

White solid; ^1^H NMR (300 MHz, DMSO-d6) δ 11.65 (s, 1H), 8.78 (t, *J* = 5.7, 5.7 Hz, 2H), 7.98 (s, 3H), 7.61 (d, *J* = 7.9 Hz, 1H), 7.42 (d, *J* = 8.2 Hz, 1H), 7.24 – 7.12 (m, 2H), 7.03 (t, *J* = 7.5, 7.5 Hz, 1H), 3.36 (t, *J* = 3.1, 3.1 Hz, 2H), 2.92-2.81 (m, 2H), 1.84 (p, *J* = 6.7, 6.7, 6.5, 6.5 Hz, 2H); ^13^C NMR (75 MHz, DMSO) δ 161.19, 136.34, 131.59, 126.96, 123.20, 121.39, 119.64, 112.22, 102.79, 36.64, 35.74, 27.28; HRMS (EI): calcd. for C_12_H_15_N_3_O: 217.1215, found: 217.12102.

**N-(3-aminopropyl)benzofuran-2-carboxamide (53b)**

White solid; ^1^H NMR (300 MHz, DMSO-d6) δ 9.05 (t, *J* = 5.9, 5.9 Hz, 1H), 8.18 (s, 3H), 7.79 (d, *J* = 7.3 Hz, 1H), 7.71 – 7.60 (m, 2H), 7.48 (td, *J* = 8.4, 7.8, 1.3 Hz, 1H), 7.41 – 7.29 (m, 1H), 3.38 (q, *J* = 6.5, 6.5, 6.5 Hz, 2H), 2.86 (h, *J* = 5.6, 5.6, 5.6, 5.5, 5.5 Hz, 2H), 1.88 (p, *J* = 6.7, 6.7, 6.7, 6.7 Hz, 2H); ^13^C NMR (75 MHz, DMSO) δ 158.27, 154.14, 148.99, 127.08, 126.74, 123.64, 122.69, 111.71, 109.37, 36.62, 35.80, 27.14; HRMS (EI): calcd. for C_12_H_14_N_2_O_2_: 218.1055, found: 218.10472.

**N-(3-aminopropyl)benzo[b]thiophene-2-carboxamide (53c)**

White solid; ^1^H NMR (300 MHz, DMSO-d6) δ 9.19 (t, *J* = 5.7, 5.7 Hz, 1H), 8.24 (s, 1H), 8.17 (s, 3H), 8.01 – 8.04 (m, 1H), 7.95-7.92 (m, 1H), 7.53 – 7.38 (m, 2H), 3.38 (q, *J* = 6.5, 6.5, 6.4 Hz, 2H), 2.88 (t, *J* = 6.7, 6.7 Hz, 2H), 1.89 (p, *J* = 6.8, 6.8, 6.7, 6.7 Hz, 2H); ^13^C NMR (75 MHz, DMSO) δ 161.64, 140.08, 139.85, 139.12, 126.11, 125.07, 124.87, 122.74, 40.01, 36.66, 36.29, 27.12; HRMS (EI): calcd. for C_12_H_14_N_2_OS: 234.0826, found: 234.0824.

**N-(3-aminopropyl)quinoline-2-carboxamide (53d)**

Off-white solid; ^1^H NMR (300 MHz, DMSO-d6) δ 9.26 (t, *J* = 6.2, 6.2 Hz, 1H), 8.63 (d, *J* = 8.3 Hz, 1H), 8.27 (bs, 3H), 8.22– 8.11 (m, 2H), 7.91 (ddd, *J* = 8.5, 6.9, 1.5 Hz, 1H), 7.75 (ddd, *J* = 8.1, 6.9, 1.2 Hz, 1H), 7.29 (s, 1H), 3.48 (q, *J* = 6.5, 6.5, 6.5 Hz, 2H), 2.90-2.82 (m, 2H), 1.95 (p, *J* = 6.8, 6.8, 6.7, 6.7 Hz, 2H); ^13^C NMR (75 MHz, DMSO) δ 164.12, 149.80, 145.59, 138.21, 130.69, 128.77, 128.09, 118.65, 36.60, 36.10, 27.22; HRMS (EI): calcd. for C_13_H_15_N_3_O: 229.1215, found: 229.12132.

**N-(3-aminopropyl)-5-fluoro-1H-indole-2-carboxamide (53e)**

White solid; ^1^H NMR (300 MHz, DMSO-d6) δ 11.81 (s, 1H), 8.94 (t, *J* = 5.8, 5.8 Hz, 1H), 8.14 (s, 3H), 7.41 (td, *J* = 9.8, 9.7, 3.6 Hz, 2H), 7.19 (d, *J* = 1.5 Hz, 1H), 7.05 (td, *J* = 9.4, 9.3, 2.6 Hz, 1H), 3.37 (q, *J* = 6.4, 6.4, 6.3 Hz, 2H), 2.87 (h, *J* = 5.6, 5.6, 5.6, 5.5, 5.5 Hz, 2H), 1.87 (p, *J* = 6.7, 6.7, 6.7, 6.7 Hz, 2H); ^13^C NMR (75 MHz, DMSO) δ 160.88, 158.60, 155.52, 133.31, 133.08, 127.10, 126.96, 113.46, 113.33, 112.08, 111.72, 105.72, 105.42, 102.79, 102.72, 36.66, 35.80, 27.26; HRMS (EI): calcd. for C_12_H_14_FN_2_O: 235.1120, found: 235.1108.

**N-(3-aminopropyl)-1H-benzo[d]imidazole-2-carboxamide (53f)**

White solid; ^1^H NMR (300 MHz, DMSO-d6) δ 9.39 (s, 1H), 8.75 (bs, 1H), 8.08 (s, 3H), 7.70 (dd, J = 6.1, 3.2 Hz, 2H), 7.39 (dd, J = 6.1, 3.2 Hz, 2H), 3.42 (q, J = 6.5, 6.4, 6.4 Hz, 2H), 2.94 – 2.82 (m, 2H), 1.90 (p, J = 7.0, 7.0, 6.9, 6.9 Hz, 2H); ^13^C NMR (75 MHz, DMSO) δ 156.13, 143.60, 134.49, 125.28, 115.46, 36.62, 36.49, 26.81; HRMS (EI): calcd. for C_11_H_14_N_4_O: 218.1167, found: 218.1130.

**Spectral data of the *N*-Boc protected linkers 9e-j.**

**tert-Butyl 3-pent-4-ynamidopropylcarbamate (9f).**

White semisolid, yield 88%; ^1^H NMR (300 MHz, CDCl_3_): δ 6.32 (br s, 1H), 4.89 (br s, 1H), 3.30 (q, *J* = 6.2 Hz, 2H), 3.17 (q, *J* = 6.0 Hz, 2H), 2.56-2.52 (m, 2H), 2.41 (t, *J* = 6.9 Hz, 2H), 1.99 (s, 1H), 1.66-1.59 (s, 2H), 1.44 (s, 9H); ^13^C NMR (75 MHz, CDCl_3_): δ 171.34, 156.69, 82.99, 79.36, 69.24, 36.99, 35.92, 35.43, 30.18, 28.40, 14.98; MS (CI): calcd. for C_13_H_22_N_2_O_3_: 254.35, found: 255.0.

**Spectral data of linkers** **10a-m.**

***N*-(3-Aminopropyl)pent-4-ynamide (10f).**

Viscous liquid, yield 82%; the compound was obtained as a hydrochloride salt. ^1^H NMR (300 MHz, D_2_O): δ 3.24 (t, *J* = 6.4 Hz, 2H), 2.95 (t, *J* = 7.3 Hz, 2H), 2.44-2.30 (m, 4H), 2.29 (s, 1H), 1.85-1.76 (m, 2H); ^13^C NMR (75 MHz, D_2_O): δ 175.06, 83.28, 70.16, 48.85, 36.97, 35.95, 34.39, 14.57; HRMS (EI): calcd. for [C_8_H_14_N_2_O+H]^+^: 155.1178, found: 155.1182.

^1^H NMR of vancomycin analogues are presented below.

**Vanc-1**

**Vanc- 1**: ^1^H NMR (300 MHz, DMSO-d6) δ 9.45 (s, 1H), 9.14 – 8.99 (m, 2H), 8.69 – 8.60 (m, 1H), 8.51 (s, 1H), 8.33 – 8.21 (m, 3H), 8.05 (d, *J* = 9.4 Hz, 1H), 7.85 (s, 1H), 7.78-7.70 (m, 1H), 7.55 – 7.22 (m, 8H), 7.11 (dd, *J* = 9.0, 3.0 Hz, 1H), 6.88 (s, 1H), 6.77-6.69 (m, 5H), 6.37 (d, *J* = 13.3 Hz, 2H), 5.76 (d, *J* = 8.9 Hz, 2H), 5.54 (s, 1H), 5.33 - 5.11 (m, 5H), 4.92 - 4.78 (m, 1H), 4.67 (s, 1H), 4.49-4.25 (m, 3H),4.04- 3.87 (s, 4H), 3.73 – 3.64 (m, 2H), 3.56-3.53 (m, 3H), 3.17 (s, 1H), 2.31 (s, 4H), 1.96 – 1.83 (m, 2H), 1.81 – 1.60 (m, 3H), 1.55 – 1.35 (m, 3H), 1.29 (s, 2H), 1.07 (d, *J* = 6.4 Hz, 3H), 0.88 (dd, *J* = 12.7, 6.6 Hz, 6H).

**Vanc-2**

**Vanc-2**: ^1^H NMR (600 MHz, DMSO-d6) δ 9.46 (s, 1H), 8.62 (s, 1H), 8.45 (s, 1H), 8.32-8.30 (m, 4H), 7.87 (s, 1H), 7.77 (s, 1H), 7.69 (d, J= 8.12 Hz, ,1H), 7.51 (d, *J* = 8.2 Hz, 1H), 7.47 (d, *J* = 8.4 Hz, 1H), 7.36-7.33 (m, 4H), 7.27 (d, *J* = 8.4 Hz, 1H), 7.23 (s, 1H), 7.15 (d, *J* = 8.2 Hz, 1H), 6.88 (s, 1H), 6.76 (d, *J* = 2.0 Hz, 1H), 6.72 (d, *J* = 8.5 Hz, 1H), 6.68 (d, *J* = 11.5 Hz, 1H), 6.40 (d, *J* = 2.0 Hz, 1H), 6.37 (d, *J* = 2.0 Hz, 1H), 5.75 (d, *J* = 8.3 Hz, 1H), 5.54 (s, 1H), 5.31 – 5.27 (m, 2H), 5.25 – 5.21 (m, 2H), 5.16 (s, 1H), 4.89 (s, 1H), 4.69 (d, *J* = 6.7 Hz, 1H), 4.45 (d, *J* = 5.6 Hz, 3H), 4.42 (d, *J* = 5.3 Hz, 1H), 4.25 (d, *J* = 10.9 Hz, 1H), 4.20 – 4.12 (m, 1H), 3.93 (d, *J* = 12.8 Hz, 2H), 3.69 (d, *J* = 10.3 Hz, 2H), 3.58 – 3.51 (m, 5H), 3.48 – 3.44 (m, 3H), 3.32 – 3.25 (m, 4H), 3.17 (s, 1H), 3.05 (t, *J* = 7.2, 7.2 Hz, 1H), 2.39 (s, 1H), 2.31 (d, *J* = 9.6 Hz, 6H), 2.15 (d, *J* = 10.4 Hz, 1H), 1.90 (d, *J* = 9.6 Hz, 1H), 1.76 – 1.67 (m, 2H), 1.49 (dt, *J* = 12.2, 6.2, 6.2 Hz, 1H), 1.41 (dt, *J* = 13.3, 7.0, 7.0 Hz, 1H), 1.28 (s, 3H), 1.07 (d, *J* = 6.3 Hz, 3H), 0.91 (d, *J* = 6.6 Hz, 3H), 0.86 (d, *J* = 6.5 Hz, 3H).

**Vanc-3**

**Vanc-3**: ^1^H NMR (600 MHz, DMSO-d6) δ 9.73 (s, 1H), 8.65 (s, 1H), 8.51 (s, 1H), 8.39 – 8.35 (m, 2H), 8.34 (s, 2H), 7.87 (s, 1H), 7.65 (d, *J* = 7.7 Hz, 2H), 7.53 – 7.47 (m, 3H), 7.36 – 7.31 (m, 5H), 7.28 – 7.25 (m, 2H), 7.07 (t, *J* = 7.4, 7.4 Hz, 1H), 6.89 (s, 1H), 6.80 – 6.71 (m, 4H), 6.40 (d, *J* = 2.0 Hz, 1H), 6.36 (d, *J* = 2.0 Hz, 1H), 5.76 (d, *J* = 8.0 Hz, 2H), 5.54 (s, 1H), 5.33 – 5.28 (m, 2H), 5.23 (s, 2H), 5.16 (s, 1H), 4.89 (s, 1H), 4.69 (d, *J* = 6.6 Hz, 2H), 4.47 (d, *J* = 3.5 Hz, 2H), 4.38 (d, *J* = 4.9 Hz, 2H), 4.29 (d, *J* = 12.3 Hz, 2H), 4.07 (dd, *J* = 16.5, 5.9 Hz, 2H), 3.92 (dd, *J* = 16.75, 6.92 Hz, 2H), 3.69 (d, *J* = 9.8 Hz, 2H), 3.58 – 3.51 (m, 6H), 3.47-3.44 (m, 3H), 3.14 (s, 2H), 3.06 – 3.02 (m, 2H), 2.31 (s, 3H), 2.15 (d, *J* = 10.8 Hz, 1H), 1.87 (d, *J* = 9.5 Hz, 1H), 1.76 – 1.66 (m, 2H), 1.49 (dt, *J* = 13.5, 6.6, 6.6 Hz, 1H), 1.41 (dt, *J* = 14.8, 7.7, 7.7 Hz, 1H), 1.27 (s, 3H), 1.07 (d, *J* = 6.2 Hz, 3H), 0.91 (d, *J* = 6.6 Hz, 3H), 0.86 (d, *J* = 6.6 Hz, 3H).

**Vanc-4**

**Vanc-4**: ^1^H NMR (300 MHz, DMSO-d6) δ 8.63 (s, 2H), 8.44-8.38 (d, *J* = 19.4 Hz, 2H), 8.27 (s, 4H), 8.03 (d, *J* = 8.4 Hz, 2H), 7.86 (s, 1H), 7.79-7.70 (m, 2H), 7.58 – 7.21 (m, 9H), 7.20 – 7.00 (m, 2H), 6.92 – 6.66 (m, 7H), 6.46 – 6.23 (m, 3H), 5.97 (s, 2H), 5.75 (d, *J* = 8.3 Hz, 2H), 5.54 (s, 2H), 5.29-5016 (m, 5H), 4.91-4.85 (s, 1H), 4.68 (d, *J* = 5.8 Hz, 1H), 4.52 – 4.18 (m, 7H), 3.72-3.66 (m, 2H), 3.03 (d, *J* = 8.0 Hz, 2H), 2.31 (s, 3H), 2.26 (s, 2H), 1.90 (s, 2H), 1.79 – 1.65 (m, 3H), 1.55 – 1.36 (m, 3H), 1.27 (d, *J* = 10.7 Hz, 3H), 1.07 (d, *J* = 5.6 Hz, 3H), 0.88 (dd, *J* = 12.5, 6.4 Hz, 6H).

**Vanc-5**

**Vanc-5**: ^1^H NMR (600 MHz, DMSO-d6) δ 9.53 (s, 1H), 8.65 (s, 1H), 8.53 (s, 1H), 8.36 (s, 1H), 8.33 (s, 1H), 7.86 (s, 2H), 7.75 (s, 1H), 7.56 (d, *J* = 9.0 Hz, 2H), 7.50 (t, *J* = 8.9, 8.9 Hz, 2H), 7.35 (d, *J* = 8.3 Hz, 3H), 7.27 (d, *J* = 8.0 Hz, 2H), 6.90 (d, *J* = 9.1 Hz, 3H), 6.81 – 6.70 (m, 3H), 6.40 (d, *J* = 1.9 Hz, 1H), 6.34 (d, *J* = 2.0 Hz, 1H), 5.76 (d, *J* = 7.1 Hz, 1H), 5.54 (s, 1H), 5.33 – 5.28 (m, 2H), 5.23 (s, 2H), 5.16 (d, *J* = 3.0 Hz, 1H), 4.89 (s, 1H), 4.69 (d, *J* = 7.2 Hz, 1H), 4.47 (d, *J* = 6.9 Hz, 1H), 4.36 (d, *J* = 4.9 Hz, 1H), 4.30 (d, *J* = 13.4 Hz, 1H), 4.02 (dd, *J* = 17.1, 5.9 Hz, 2H), 3.90 (dd, *J* = 16.3, 6.6 Hz, 1H), 3.73 (s, 3H), 3.69 (d, *J* = 10.7 Hz, 2H), 3.59 – 3.50 (m, 5H), 3.48 – 3.44 (m, 4H), 3.29 – 3.25 (m, 4H), 3.14 (s, 1H), 3.04 (t, *J* = 6.1, 6.1 Hz, 1H), 2.31 (s, 3H), 2.15 (d, *J* = 11.9 Hz, 1H), 1.86 (s, 1H), 1.77 – 1.66 (m, 2H), 1.52 – 1.46 (m, 1H), 1.41 (dt, *J* = 14.6, 7.3, 7.3 Hz, 1H), 1.27 (s, 3H), 1.07 (d, *J* = 6.2 Hz, 3H), 0.91 (d, *J* = 6.6 Hz, 3H), 0.86 (d, *J* = 6.6 Hz, 3H).

**Vanc-6**

**Vanc-6**: ^1^H NMR (600 MHz, DMSO-d6) δ 9.84 (s, 1H), 8.66 (s, 1H), 8.54 (s, 1H), 8.45 (s, 1H), 8.32 (s, 2H), 7.86 (s, 1H), 7.76 (s, 1H), 7.70 (d, *J* = 8.9 Hz, 2H), 7.50 (t, *J* = 8.4, 8.4 Hz, 2H), 7.39 (d, *J* = 8.8 Hz, 3H), 7.35 (d, *J* = 8.4 Hz, 2H), 7.27 (d, *J* = 8.3 Hz, 2H), 6.89 (s, 1H), 6.82 – 6.77 (m, 2H), 6.72 (d, *J* = 8.5 Hz, 1H), 6.65 (s, 1H), 6.40 (d, *J* = 2.0 Hz, 1H), 6.35 (d, *J* = 2.0 Hz, 1H), 5.76 (d, *J* = 8.3 Hz, 1H), 5.54 (s, 1H), 5.33 – 5.26 (m, 3H), 5.24 (s, 2H), 5.16 (d, *J* = 3.5 Hz, 1H), 4.89 (s, 1H), 4.69 (d, *J* = 6.4 Hz, 1H), 4.48 (s, 1H), 4.36 (d, *J* = 5.1 Hz, 1H), 4.30 (d, *J* = 9.3 Hz, 1H), 4.05 (dd, *J* = 16.8, 6.4 Hz, 2H), 3.93 (dd, *J* = 17.3, 6.5 Hz, 2H), 3.69 (d, *J* = 10.6 Hz, 2H), 3.59 – 3.50 (m, 6H), 3.46 (t, *J* = 8.8, 8.8 Hz, 4H), 3.28 (dd, *J* = 17.0, 8.0 Hz, 4H), 3.16 (s, 1H), 3.05 (t, *J* = 7.2, 7.2 Hz, 2H), 2.31 (s, 3H), 2.15 (d, *J* = 11.9 Hz, 1H), 1.89 (d, *J* = 13.3 Hz, 1H), 1.77 – 1.66 (m, 2H), 1.49 (dt, *J* = 13.4, 6.6, 6.6 Hz, 1H), 1.44 – 1.38 (m, 1H), 1.28 (s, 3H), 1.07 (d, *J* = 6.3 Hz, 3H), 0.91 (d, *J* = 6.6 Hz, 3H), 0.86 (d, *J* = 6.6 Hz, 3H).

**Vanc-7**

**Vanc-7**: ^1^H NMR (600 MHz, DMSO-d6) δ 9.86 (s, 1H), 9.18 (s, 1H), 8.66 (s, 1H), 8.55 (s, 1H), 8.47 (s, 1H), 8.33 (s, 2H), 7.91 (d, *J* = 8.5 Hz, 2H), 7.87 (s, 1H), 7.84 (d, *J* = 8.4 Hz, 2H), 7.79 (d, *J* = 8.6 Hz, 2H), 7.51 (d, *J* = 8.5 Hz, 2H), 7.44 (d, *J* = 8.4 Hz, 2H), 7.36 (d, *J* = 8.3 Hz, 2H), 7.30 – 7.25 (m, 1H), 6.89 (s, 2H), 6.79 (t, *J* = 7.8, 7.8 Hz, 1H), 6.73 (d, *J* = 8.5 Hz,1), 6.41 (d, *J* = 2.3 Hz, 1H), 6.37 (d, *J* = 1.8 Hz, 1H), 5.77 (d, *J* = 8.4 Hz, 1H), 5.54 (s, 1H), 5.34 (s, 1H), 5.29 (d, *J* = 7.7 Hz, 1H), 5.24 (s, 2H), 5.16 (s, 1H), 4.89 (s, 1H), 4.69 (d, *J* = 6.3 Hz, 1H), 4.48 (s, 1H), 4.38 (d, *J* = 4.9 Hz, 1H), 4.31 (d, *J* = 11.4 Hz, 1H), 4.09 (dd, *J* = 16.8, 6.1 Hz, 1H), 3.96 (dd, *J* = 16.6, 5.5 Hz, 2H), 3.69 (d, *J* = 10.1 Hz, 2H), 3.58 – 3.51 (m, 6H), 3.46 (t, *J* = 8.7, 8.7 Hz, 4H), 3.32-3.29 (m, 4H), 3.18 (s, 1H), 3.06 (t, *J* = 7.3, 7.3 Hz, 1H), 2.41 (s, 3H), 2.32 (s, 3H), 1.90 (d, *J* = 8.6 Hz, 1H), 1.72 (dt, *J* = 19.4, 9.6, 9.6 Hz, 2H), 1.49 (dt, *J* = 14.0, 6.9, 6.9 Hz, 1H), 1.44 – 1.38 (m, 1H), 1.29 (s, 3H), 1.07 (d, *J* = 6.2 Hz, 3H), 0.91 (d, *J* = 6.6 Hz, 3H), 0.86 (d, *J* = 6.6 Hz, 3H).

**Vanc-9**

**Vanc-9**: ^1^H NMR (600 MHz, DMSO-d6) δ 8.98 (s, 1H), 8.62 (s, 1H), 8.47 (s, 1H), 8.32 (s, 2H), 7.85 (s, 1H), 7.50 (d, *J* = 8.7 Hz, 1H), 7.46 (d, *J* = 8.4 Hz, 1H), 7.36 – 7.31 (m, 3H), 7.28 – 7.23 (m, 2H), 6.86 (s, 2H), 6.76 (d, *J* = 10.2 Hz, 1H), 6.72 – 6.67 (m, 2H), 6.39 (d, *J* = 1.9 Hz, 1H), 6.34 (d, *J* = 2.0 Hz, 1H), 5.74 (d, *J* = 8.2 Hz, 2H), 5.53 (s, 1H), 5.28 (d, *J* = 8.7 Hz, 2H), 5.22 (s, 2H), 5.15 (s, 1H), 4.87 (s, 1H), 4.68 (d, *J* = 6.4 Hz, 1H), 4.44 (s, 1H), 4.36 (d, *J* = 4.9 Hz, 2H), 4.27 (d, *J* = 11.2 Hz, 1H), 4.06 (dd, *J* = 16.2, 6.1 Hz, 2H), 3.90 (dd, *J* = 15.7, 5.5 Hz, 2H), 3.68 (d, *J* = 10.8 Hz, 2H), 3.57 – 3.50 (m, 5H), 3.45-3.43 (m, 4H), 3.29 – 3.24 (m, 6H), 3.13 (s, 1H), 3.05 – 3.02 (m, 1H), 2.38 (s, 1H), 2.30 (s, 3H), 2.22 (s, 3H), 2.09 (s, 6H), 1.87 (d, *J* = 9.1 Hz, 1H), 1.75 – 1.66 (m, 2H), 1.50-1.46 (m, 1H), 1.41-1.38 (m, 1H), 1.26 (s, 3H), 1.06 (d, *J* = 6.2 Hz, 3H), 0.90 (d, *J* = 6.6 Hz, 3H), 0.85 (d, *J* = 6.6 Hz, 3H).

**Vanc-10**

**Vanc-10:** ^1^H NMR (600 MHz, DMSO-d6) δ 10.01 (s, 1H), 8.65 (s, 1H), 8.51 (s, 1H), 8.41 (s, 1H), 8.32 (s, 2H), 7.86 (s, 1H), 7.75 (s, 1H), 7.65 (d, *J* = 12.4 Hz, 1H), 7.53 – 7.46 (m, 2H), 7.40 – 7.31 (m, 5H), 7.26 (d, *J* = 7.7 Hz, 2H), 6.91-6.88 (m, 2H), 6.78 (d, *J* = 9.0 Hz, 1H), 6.72 (d, *J* = 8.6 Hz, 2H), 6.40 (d, *J* = 1.9 Hz, 1H), 6.35 (d, *J* = 2.1 Hz, 1H), 5.76 (d, *J* = 8.3 Hz, 2H), 5.53 (s, 1H), 5.31 (s, 1H), 5.28 (d, *J* = 7.7 Hz, 1H), 5.23 (s, 2H), 5.15 (s, 1H), 4.88 (s, 1H), 4.68 (d, *J* = 6.5 Hz, 1H), 4.46 (d, *J* = 5.2 Hz, 1H), 4.37 (s, 2H), 4.28 (d, *J* = 13.1 Hz, 1H), 4.07 (dd, *J* = 17.6, 7.2 Hz, 2H), 3.92 (dd, *J* = 16.5, 5.8 Hz, 1H), 3.68 (d, *J* = 10.7 Hz, 1H), 3.57 – 3.50 (m, 5H), 3.47 – 3.43 (m, 5H), 3.31-3.25 (m, 4H), 3.15 (s, 1H), 3.04 (t, *J* = 7.3, 7.3 Hz, 1H), 2.42 – 2.36 (m, 1H), 2.31 (s, 3H), 2.20 – 2.11 (m, 1H), 1.88 (d, *J* = 10.4 Hz, 1H), 1.77 – 1.65 (m, 2H), 1.48 (dt, *J* = 13.8, 7.1, 7.1 Hz, 1H), 1.44 – 1.37 (m, 1H), 1.27 (s, 3H), 1.06 (d, *J* = 6.3 Hz, 3H), 0.90 (d, *J* = 6.6 Hz, 3H), 0.86 (d, *J* = 6.6 Hz, 3H).

**Vanc-11**

**Vanc-11:** ^1^H NMR (600 MHz, DMSO-d6) δ 9.66 (s, 1H), 8.64 (s, 1H), 8.49 (s, 1H), 8.33 (s, 2H), 8.29 (s, 1H), 7.87 (s, 1H), 7.50 (dd, *J* = 14.3, 7.5 Hz, 2H), 7.37 – 7.33 (m, 3H), 7.28 – 7.24 (m, 4H), 6.89 (s, 1H), 6.79 – 6.77 (m, 1H), 6.72 (d, *J* = 10.3 Hz, 2H), 6.66 (d, *J* = 11.7 Hz, 2H), 6.40 (d, *J* = 2.0 Hz, 1H), 6.36 (d, *J* = 2.0 Hz, 1H), 5.76 (d, *J* = 8.6 Hz, 2H), 5.54 (s, 1H), 5.34 – 5.27 (m, 2H), 5.23 (s, 2H), 5.15 (s, 1H), 4.89 (s, 1H), 4.69 (d, *J* = 6.9 Hz, 1H), 4.46 (d, *J* = 4.4 Hz, 1H), 4.39 (d, *J* = 4.9 Hz, 2H), 4.28 (d, *J* = 13.0 Hz, 1H), 4.06 (dd, *J* = 16.5, 6.3 Hz, 2H), 3.86 (dd, *J* = 15.8, 5.9 Hz, 2H), 3.69 (d, *J* = 8.2 Hz, 2H), 3.59 – 3.51 (m, 5H), 3.48 – 3.42 (m, 5H), 3.28 (dd, *J* = 16.3, 8.8 Hz, 4H), 3.15 (s, 1H), 3.06 – 3.03 (m, 1H), 2.39 (s, 1H), 2.31 (s, 3H), 2.25 (s, 6H), 1.88 (d, *J* = 14.6 Hz, 1H), 1.70 (s, 2H), 1.51-1.47 (m, 1H), 1.44 – 1.37 (m, 1H), 1.27 (s, 3H), 1.07 (d, *J* = 6.4 Hz, 3H), 0.91 (d, *J* = 6.5 Hz, 3H), 0.86 (d, *J* = 6.5 Hz, 3H).

**Vanc-12**

**Vanc-12**: ^1^H NMR (600 MHz, DMSO-d6) δ 9.78 (s, 1H), 8.65 (s, 1H), 8.50 (d, *J* = 4.8 Hz, 1H), 8.33 (d, *J* = 8.9 Hz, 1H), 7.87 (s, 3H), 7.50 (dd, *J* = 16.2, 8.7 Hz, 2H), 7.40 – 7.31 (m, 4H), 7.29 – 7.20 (m, 3H), 7.17 (d, *J* = 8.2 Hz, 1H), 6.89 (s, 1H), 6.78 (d, *J* = 9.9 Hz, 1H), 6.72 (d, *J* = 8.4 Hz, 1H), 6.66 (t, *J* = 9.3, 9.3 Hz, 3H), 6.40 (d, *J* = 2.0 Hz, 1H), 6.36 (d, *J* = 1.9 Hz, 1H), 5.76 (d, *J* = 8.1 Hz, 1H), 5.54 (s, 1H), 5.33 – 5.27 (m, 3H), 5.23 (s, 2H), 5.16 (s, 1H), 4.89 (s, 1H), 4.69 (d, *J* = 6.8 Hz, 1H), 4.47 (d, *J* = 6.0 Hz, 2H), 4.38 (d, *J* = 5.2 Hz, 2H), 4.29 (d, *J* = 12.8 Hz, 2H), 4.06 (dd, *J* = 16.4, 6.3 Hz, 2H), 3.90 (dd, *J* = 16.0, 5.7 Hz, 2H), 3.75 (s, 3H), 3.69 (d, *J* = 10.0 Hz, 2H), 3.58 – 3.50 (m, 4H), 3.48 – 3.44 (m, 4H), 3.34 – 3.25 (m, 3H), 3.15 (s, 1H), 3.07 – 3.02 (m, 1H), 2.39 (s, 1H), 2.31 (s, 3H), 2.15 (d, *J* = 8.9 Hz, 1H), 1.89 (d, *J* = 12.3 Hz, 1H), 1.77 – 1.66 (m, 2H), 1.49 (dt, *J* = 14.6, 7.1, 7.1 Hz, 1H), 1.41 (dt, *J* = 14.6, 7.8, 7.8 Hz, 1H), 1.27 (s, 3H), 1.07 (d, *J* = 6.3 Hz, 3H), 0.91 (d, *J* = 6.6 Hz, 3H), 0.86 (d, *J* = 6.5 Hz, 3H).

**Vanc-13**

**Vanc-13:** ^1^H NMR (300 MHz, DMSO-d6) δ 9.62 (s, 1H), 8.65-8.51 (m, 3H), 8.33 (s, 2 H), 7.85 (s, 1H), 7.73 (s, 1H), 7.53-7.50 (m, 4H), 7.36-7.27 (m, 4H), 7.15 (d, *J* = 8.5 Hz, 2H), 6.96 – 6.55 (m, 6H), 6.37 (d, *J* = 14.9 Hz, 2H), 5.76 (d, *J* = 8.2 Hz, 1H), 5.53 (s, 1H), 5.38 – 5.09 (m, 5H), 4.87 (s, 1H), 4.70-4.67 (m, 1H), 4.56 – 4.23 (m, 3H), 4.12 – 3.79 (m, 1H), 4.06-3.85 (m, 5H), 3.71-3.67 (m, 2H), 3.56-3.53 (m, 4H), 3.43– 3.29 (m, 6H), 3.13 (s, 1H), 3.07-3.01 (m, 2H), 2.57 (s, 2H), 2.31 (s, 2H), 2.17 (s, 1H), 1.86 (d, *J* = 20.7 Hz, 2H), 1.77 – 1.59 (m, 2H), 1.44 (s, 2H), 1.26 (s, 3H), 1.22 – 1.01 (m, 5H), 0.88 (dd, *J* = 12.9, 6.4 Hz, 6H).

**Vanc-14**

**Vanc-14**: ^1^H NMR (300 MHz, DMSO-d6) δ 10.11 (s, 1H), 8.64 (s, 1H), 8.50 (d, *J* = 16.9 Hz, 2H), 8.29 (s, 3H), 8.23 (s, 1H), 8.08 (s, 1H), 7.86 (s, 1H), 7.79 (d, *J* = 9.3 Hz, 1H), 7.54 (dt, *J* = 23.5, 7.6, 7.6 Hz, 4H), 7.30 (dd, *J* = 23.4, 8.1 Hz, 4H), 6.88 (s, 1H), 6.73 (dt, *J* = 22.6, 11.3, 11.3 Hz, 3H), 6.37 (d, *J* = 15.3 Hz, 2H), 5.76 (d, *J* = 8.3 Hz, 2H), 5.59 – 5.44 (m, 2H), 5.37 – 5.21 (m, 4H), 5.16 (d, *J* = 3.9 Hz, 1H), 4.88 (s, 1H), 4.68 (d, *J* = 6.7 Hz, 1H), 4.49 – 4.22 (m, 4H), 4.12 – 3.87 (m, 4H), 3.75 – 3.35 (m, 7H), 3.28 (d, *J* = 6.8 Hz, 1H), 3.18 (s, 1H), 3.12 – 3.01 (m, 1H), 2.32 (s, 3H), 2.18-2.14(m-1H), 2.08 (d, *J* = 3.7 Hz, 2H), 1.98 – 1.81 (m, 2H), 1.79 – 1.62 (m, 3H), 1.56 – 1.35 (m, 2H), 1.29 (s, 3H), 1.07 (d, *J* = 6.0 Hz, 2H), 1.00 (d, *J* = 6.5 Hz, 3H), 0.88 (dd, *J* = 13.0, 6.5 Hz, 6H).

**Vanc-15**

**Vanc-15**: ^1^H NMR (300 MHz, DMSO-d6) δ 9.10 (s, 2H), 8.64 (s, 1H), 8.47 (s, 1H), 8.33 (s, 3H), 7.86 (s, 1H), 7.74 (s, 2H), 7.55 – 7.20 (m, 9H), 7.10 – 6.84 (m, 4H), 6.82 – 6.61 (m, 4H), 6.36 (d, *J* = 13.7 Hz, 2H), 5.75 (d, *J* = 9.9 Hz, 2H), 5.54 (s, 1H), 5.35 – 5.11 (m, 6H), 4.88 (s, 2H), 4.68 (d, *J* = 5.9 Hz, 1H), 4.52 – 4.24 (m, 4H), 4.16 – 4.02 (m, 2H), 3.90 (d, *J* = 19.8 Hz, 4H), 3.13 (s, 1H), 3.04 (t, *J* = 7.4, 7.4 Hz, 2H), 2.30 (s, 3H), 2.25 (s, 3H), 2.16 (s, 3H), 1.96 – 1.61 (m, 6H), 1.57 – 1.36 (m, 4H), 1.26 (s, 3H), 1.06 (d, *J* = 6.1 Hz, 3H), 0.88 (dd, *J* = 12.9, 6.6 Hz, 6H).

**Vanc-17**

**Vanc-17**: ^1^H NMR (300 MHz, DMSO-d6) δ 9.79 (s, 1H), 8.64 (s, 1H), 8.49 (s, 1H), 8.35 (s, 3H), 8.21 (s, 1H), 7.87 (s, 1H), 7.76 (s, 1H), 7.55 – 7.42 (m, 2H), 7.33 (d, *J* = 8.5 Hz, 3H), 7.26 (d, *J* = 8.0 Hz, 2H), 6.88 (d, *J* = 2.1 Hz, 2H), 6.81 – 6.69 (m, 3H), 6.56 (d, *J* = 11.7 Hz, 1H), 6.40 (s, 1H), 6.34 (s, 1H), 6.23 (t, *J* = 2.0, 2.0 Hz, 1H), 5.75 (d, *J* = 8.1 Hz, 1H), 5.51 (d, *J* = 13.2 Hz, 2H), 5.35 – 5.21 (m, 4H), 5.15 (d, *J* = 4.2 Hz, 1H), 4.87 (s, 1H), 4.68 (d, *J* = 6.7 Hz, 1H), 4.45 (d, *J* = 3.9 Hz, 1H), 4.37 (d, *J* = 4.8 Hz, 2H), 4.29 (d, *J* = 12.6 Hz, 1H), 4.11 – 4.00 (m, 2H), 3.90-3.86 (m, 4H), 3.73 (s, 6H), 3.68 – 3.43 (m, 8H), 3.28 (s, 1H), 3.15 (s, 1H), 3.09 – 3.01 (m, 1H), 2.31 (s, 3H), 2.21 – 2.04 (m, 2H), 1.89 (d, *J* = 10.3 Hz, 1H), 1.80 – 1.62 (m, 2H), 1.44 (dq, *J* = 21.7, 7.9, 7.6, 7.6 Hz, 2H), 1.27 (s, 3H), 1.06 (d, *J* = 6.3 Hz, 2H), 1.00 (d, *J* = 6.5 Hz, 3H), 0.88 (dd, *J* = 12.9, 6.5 Hz, 6H).

**Vanc-21**

**Vanc-21**: ^1^H NMR (300 MHz, DMSO-d6) δ 8.61 (s, 1H), 8.44 (s, 2H), 8.30 (s, 3H), 8.13 (s, 1H), 7.88 (s, 1H), 7.80 (s, 1H), 7.58-7.23 (m, 13H), 6.95 – 6.59 (m, 5H), 6.30 (d, *J* = 37.4 Hz, 1H), 5.75 (d, *J* = 7.6 Hz, 1H), 5.54 (s, 1H), 5.40 – 5.13 (m, 5H), 4.89 (s, 1H), 4.68 (d, *J* = 6.3 Hz, 1H), 4.50 – 4.34 (m, 5H), 4.24 (d, *J* = 13.7 Hz, 3H), 3.69 (d, *J* = 11.0 Hz, 4H), 3.56-3.45 (m, 6H), 3.44 – 3.24 (m, 7H), 3.19 (s, 1H), 3.11 – 3.05 (m, 1H), 2.32 (s, 3H), 2.18-2.15 (m, 1H), 2.08 (d, *J* = 3.9 Hz, 1H), 1.92 (d, *J* = 11.9 Hz, 1H), 1.72 (d, *J* = 8.1 Hz, 2H), 1.54 – 1.36 (m, 2H), 1.30 (s, 3H), 1.09 (dd, *J* = 9.9, 6.6 Hz, 3H), 0.88 (dd, *J* = 13.1, 6.4 Hz, 6H).

**Vanc-22**

**Vanc-22**: ^1^H NMR (300 MHz, DMSO-d6) δ 8.61 (s, 1H), 8.42 (s, 2H), 8.32 (s, 2H), 8.24 (s, 1H), 8.12 (s, 1H), 7.87 (s, 1H), 7.77 (s, 1H), 7.68 – 7.57 (m, 2H), 7.55 – 7.20 (m, 9H), 6.87 (s, 1H), 6.80 – 6.52 (m, 5H), 6.36 (s, 1H), 6.23 (s, 1H), 5.74 (d, *J* = 7.0 Hz, 2H), 5.54 (s, 2H), 5.36 – 5.11 (m, 6H), 4.88 (s, 1H), 4.68 (d, *J* = 7.0 Hz, 1H), 4.44 (d, *J* = 2.5 Hz, 2H), 4.36 (d, *J* = 5.5 Hz, 2H), 4.23 (d, *J* = 13.6 Hz, 2H), 3.29 (s, 8H), 3.17 (s, 2H), 3.08 – 3.01 (m, 3H), 2.31 (s, 3H), 2.19 – 2.06 (m, 2H), 1.94 – 1.85 (m, 2H), 1.71 (dd, *J* = 13.2, 7.6 Hz, 3H), 1.54 – 1.34 (m, 3H), 1.28 (s, 3H), 1.06 (d, *J* = 6.2 Hz, 3H), 0.88 (dd, *J* = 12.9, 6.5 Hz, 6H).

**Vanc-24**

**Vanc-24**: ^1^H NMR (600 MHz, DMSO-d6) δ 11.68 (s, 1H), 8.74 (s, 1H), 8.61 (s, 1H), 8.48 (s, 1H), 8.34 (s, 3H), 8.12 (s, 1H), 7.99 (s, 1H), 7.89 (s, 1H), 7.62 (d, *J* = 8.0 Hz, 1H), 7.52 (d, *J* = 8.3 Hz, 2H), 7.47 (d, *J* = 8.3 Hz, 1H), 7.44 (d, *J* = 8.2 Hz, 1H), 7.39 – 7.30 (m, 3H), 7.26 (d, *J* = 8.5 Hz, 2H), 7.18 (t, *J* = 7.6, 7.6 Hz, 1H), 7.13 (s, 1H), 7.04 (t, *J* = 7.5, 7.5 Hz, 1H), 6.88 (s, 1H), 6.77 (d, *J* = 8.5 Hz, 2H), 6.72 (d, *J* = 8.5 Hz, 2H), 6.38 (s, 1H), 6.27 (s, 1H), 5.77 (s, 1H), 5.75 (s, 1H), 5.54 (s, 1H), 5.35 (s, 1H), 5.29 (d, *J* = 7.5 Hz, 1H), 5.24 (s, 2H), 5.18 (s, 1H), 4.92 (s, 2H), 4.69 (d, *J* = 6.3 Hz, 2H), 4.45 (s, 2H), 4.38 (d, *J* = 5.1 Hz, 2H), 4.26 (d, *J* = 10.4 Hz, 1H), 3.69 (d, *J* = 10.3 Hz, 1H), 3.58-3.53 (m, 2H), 3.47 (t, *J* = 8.1, 8.1 Hz, 1H), 3.41 – 3.11 (m, 8H), 2.37 (s, 3H), 2.17 (d, *J* = 11.7 Hz, 1H), 1.93 (d, *J* = 9.2 Hz, 1H), 1.80 – 1.64 (m, 4H), 1.54 (dt, *J* = 12.4, 6.3, 6.3 Hz, 1H), 1.44 (dt, *J* = 13.2, 5.8, 5.8 Hz, 1H), 1.30 (s, 3H), 1.08 (d, *J* = 6.0 Hz, 3H), 0.91 (d, *J* = 6.5 Hz, 3H), 0.86 (d, *J* = 6.5 Hz, 3H).

**Vanc-25**

**Vanc-25**: ^1^H NMR (300 MHz, DMSO-d6) δ 8.94 – 8.85 (m, 1H), 8.61 (s, 1H), 8.47 (s, 1H), 8.32 (s, 2H), 8.21 (s, 2H), 8.06 (s, 1H), 7.87 (s, 1H), 7.78 (d, *J* = 7.6 Hz, 1H), 7.65 (d, *J* = 7.9 Hz, 1H), 7.60 – 7.42 (m, 4H), 7.41 – 7.19 (m, 5H), 6.88 (s, 1H), 6.81 – 6.64 (m, 4H), 6.36 (s, 1H), 6.26 (s, 1H), 5.73 (s, 1H), 5.54 (s, 1H), 5.37 – 5.12 (m, 7H), 4.87 (s, 2H), 4.68 (d, *J* = 7.2 Hz, 2H), 4.49 – 4.33 (m, 4H), 4.24 (d, *J* = 12.9 Hz, 2H), 4.03 (d, *J* = 7.0 Hz, 2H), 3.68 (d, *J* = 11.8 Hz, 4H), 3.28 (s, 2H), 3.15 (s, 2H), 3.09 – 2.98 (m, 4H), 2.31 (s, 4H), 2.08 (d, *J* = 3.9 Hz, 1H), 1.99 (s, 1H), 1.68 (d, *J* = 14.3 Hz, 4H), 1.54 – 1.33 (m, 3H), 1.27 (s, 3H), 1.06 (d, *J* = 6.0 Hz, 3H), 0.88 (dd, *J* = 12.9, 6.4 Hz, 6H).

**Vanc-26**

**Vanc-26**: ^1^H NMR (300 MHz, DMSO-d6) δ 8.98 – 8.91 (m, 1H), 8.61 (s, 1H), 8.46 (s, 1H), 8.31 (s, 2H), 8.22 (s, 1H), 8.10 (s, 1H), 8.02 (d, *J* = 8.2 Hz, 1H), 7.94 (d, *J* = 8.3 Hz, 1H), 7.87 (s, 1H), 7.76 (s, 1H), 7.55 – 7.41 (m, 4H), 7.36-7.23 (m, 5H), 6.88 (s, 1H), 6.80 – 6.63 (m, 4H), 6.36 (s, 1H), 6.26 (s, 1H), 5.75 (d, *J* = 7.3 Hz, 1H), 5.54 (s, 1H), 5.35 – 5.12 (m, 7H), 4.88 (s, 1H), 4.68 (d, *J* = 6.0 Hz, 2H), 4.44 (d, *J* = 4.9 Hz, 1H), 4.38 (d, *J* = 5.2 Hz, 2H), 4.26 (s, 2H), 3.68 (d, *J* = 10.9 Hz, 2H), 3.60 – 3.41 (m, 6H), 3.37 – 3.22 (m, 6H), 3.16 (s, 1H), 3.09 – 3.01 (m, 1H), 2.31 (s, 3H), 2.21 – 2.05 (m, 2H), 1.88 (d, *J* = 7.1 Hz, 2H), 1.80 – 1.64 (m, 4H), 1.54 – 1.34 (m, 3H), 1.27 (s, 3H), 1.05 (s, 3H), 0.88 (dd, *J* = 13.0, 6.4 Hz, 6H).

**Vanc-29**

**Vanc-29**: ^1^H NMR (300 MHz, DMSO-d6) δ 8.80 – 8.71 (m, 1H), 8.59 (s, 1H), 8.47 (s, 1H), 8.38 (d, *J* = 6.4 Hz, 4H), 8.20 (s, 1H), 8.10 (s, 1H), 7.89 (q, *J* = 8.6, 8.6, 7.9 Hz, 3H), 7.75 (s, 1H), 7.55 – 7.14 (m, 8H), 6.87 (s, 1H), 6.72 (dt, *J* = 16.4, 8.5, 8.5 Hz, 4H), 6.36 (s, 1H), 6.28 (s, 1H), 5.74 (d, *J* = 9.0 Hz, 1H), 5.51 (d, *J* = 7.3 Hz, 5H), 5.39 – 5.11 (m, 6H), 4.87 (s, 2H), 4.72 – 4.62 (m, 2H), 4.49 – 4.18 (m, 6H), 3.90 (s, 3H), 3.69-3.58 (m, 10H), 3.13-3.01 (3, *J* = 8.1 Hz, 4H), 2.31 (s, 2H), 2.13 (t, *J* = 15.9, 15.9 Hz, 2H), 1.88 (s, 1H), 1.80 – 1.62 (m, 4H), 1.55 – 1.33 (m, 2H), 1.28 – 1.15 (m, 3H), 1.05 (d, *J* = 6.2 Hz, 3H), 0.88 (dd, *J* = 13.0, 6.4 Hz, 6H).

**Vanc-33**

**Vanc-33**: ^1^H NMR (600 MHz, DMSO-d6) δ 9.09 (bs, 2H), 8.72 (t, *J* = 5.5, 5.5 Hz, 1H), 8.61 (s, 1H), 8.48 (s, 1H), 8.32 (s, 2H), 8.07 (t, *J* = 4.9, 4.9 Hz, 1H), 7.97 (d, *J* = 8.3 Hz, 2H), 7.88 (s, 1H), 7.79 (d, *J* = 8.2 Hz, 2H), 7.74 (d, *J* = 7.5 Hz, 2H), 7.50 (t, *J* = 7.8, 7.8 Hz, 4H), 7.46 (d, *J* = 8.3 Hz, 1H), 7.42 (t, *J* = 7.4, 7.4 Hz, 1H), 7.36 (s, 1H), 7.33 (d, *J* = 8.2 Hz, 1H), 7.27 (d, *J* = 8.3 Hz, 1H), 7.24 (s, 1H), 6.88 (s, 1H), 6.77 (d, *J* = 8.4 Hz, 1H), 6.73 – 6.68 (m, 2H), 6.37 (s, 1H), 6.28 (s, 1H), 5.75 (d, *J* = 8.0 Hz, 1H), 5.54 (s, 1H), 5.33-5.25 (m, 4H), 5.15 (s, 1H), 4.88 (s, 1H), 4.68 (q, *J* = 6.2, 6.0, 6.0 Hz, 1H), 4.45 (s, 1H), 4.38 (d, *J* = 5.3 Hz, 2H), 4.25 (d, *J* = 11.1 Hz, 1H), 3.69 (d, *J* = 10.4 Hz, 2H), 3.55 (dd, *J* = 16.7, 8.3 Hz, 7H), 3.32 – 3.23 (m, 8H), 3.22 – 3.14 (m, 4H), 3.08 – 3.02 (m, 2H), 2.41 – 2.35 (m, 1H), 2.32 (s, 3H), 2.15 (d, *J* = 11.0 Hz, 1H), 1.89 (d, *J* = 8.4 Hz, 1H), 1.72 (dq, *J* = 21.5, 14.0, 10.3, 10.3 Hz, 4H), 1.52 – 1.46 (m, 1H), 1.41 (dt, *J* = 14.0, 7.3, 7.3 Hz, 1H), 1.28 (s, 2H), 1.10 (t, *J* = 7.0, 7.0 Hz, 1H), 1.07 (d, *J* = 6.2 Hz, 3H), 0.91 (d, *J* = 6.5 Hz, 3H), 0.86 (d, *J* = 6.5 Hz, 3H).

**Vanc-34**

**Vanc-34**: ^1^H NMR (300 MHz, DMSO-d6) δ 8.79 – 8.69 (m, 2H), 8.61 (s, 1H), 8.48 (s, 1H), 8.33 (s, 2H), 8.23 (s, 1H), 8.09 (s, 2H), 7.96 (d, *J* = 8.3 Hz, 2H), 7.88 (s, 1H), 7.78 (t, *J* = 7.2, 7.2 Hz, 4H), 7.54 – 7.43 (m, 3H), 7.40 – 7.20 (m, 6H), 6.88 (s, 1H), 6.80 – 6.63 (m, 4H), 6.37 (s, 1H), 6.27 (s, 1H), 5.75 (d, *J* = 8.0 Hz, 2H), 5.54 (s, 2H), 5.38 – 5.13 (m, 6H), 4.88 (s, 2H), 4.68 (d, *J* = 6.2 Hz, 2H), 4.50 – 4.34 (m, 4H), 4.25 (d, *J* = 14.1 Hz, 2H), 3.68 (d, *J* = 10.6 Hz, 2H), 3.59 – 3.38 (m, 4H), 3.23 (d, *J* = 32.1 Hz, 5H), 3.11 – 3.01 (m, 1H), 2.31 (s, 3H), 2.17 (s, 1H), 1.90 (s, 1H), 1.72 (dt, *J* = 13.3, 7.1, 7.1 Hz, 4H), 1.56 – 1.33 (m, 3H), 1.28 (s, 3H), 1.06 (d, *J* = 6.1 Hz, 3H), 1.00 (d, *J* = 6.5 Hz, 2H), 0.88 (dd, *J* = 13.0, 6.5 Hz, 6H).

**Vanc-35**

**Vanc-35**: ^1^H NMR (300 MHz, DMSO-d6) δ 8.78 – 8.68 (m, 1H), 8.60 (s, 1H), 8.47 (s, 1H), 8.31 (s, 2H), 8.23 (s, 1H), 8.08 (s, 1H), 7.98 (d, *J* = 8.4 Hz, 2H), 7.87-7.82 (m, 9H), 7.56 – 7.20 (m, 10H), 6.93 – 6.64 (m, 5H), 6.37 (s, 1H), 6.28 (s,1H), 5.75 (d, *J* = 7.8 Hz, 1H), 5.59 – 5.46 (m, 2H), 5.38 – 5.15 (m, 6H), 4.88 (s, 2H), 4.68 (d, *J* = 6.2 Hz, 2H), 4.45 (d, *J* = 4.10 Hz, 2H), 4.38 (d, *J* = 5.16 Hz, 2H), 4.25 (d, *J* = 10.7 Hz, 2H), 3.75 – 3.36 (m, 12H), 3.18 (s, 2H), 3.10 – 3.02 (m, 2H), 2.31 (s, 3H), 2.22 – 2.09 (m, 1H), 1.77-1.68 (m, 3H), 1.55 – 1.34 (m, 3H), 1.28 (s, 3H), 1.06 (d, *J* = 6.2 Hz, 2H), 1.00 (d, *J* = 6.5 Hz, 3H), 0.88 (dd, *J* = 12.9, 6.5 Hz, 6H).

**Vanc-37**

**Vanc-37:** ^1^H NMR (300 MHz, DMSO-d6) δ 9.85 (s, 1H), 8.64 (s, 1H), 8.52 (s, 1H), 8.42 (s, 2H), 8.32 (s, 2H), 8.22 (s, 1H), 7.86 (s, 1H), 7.77 (s, 1H), 7.63 (d, *J* = 8.4 Hz, 2H), 7.55 – 7.43 (m, 2H), 7.40 – 7.22 (m, 6H), 6.88 (s, 1H), 6.73 (dt, *J* = 23.5, 11.5, 11.5 Hz, 4H), 6.37 (d, *J* = 14.2Hz, 2H), 5.76 (d, *J* = 8.4 Hz, 1H), 5.58 – 5.46 (m, 2H), 5.36 – 5.11 (m, 6H), 4.88 (s, 1H), 4.68 (d, *J* = 6.1 Hz, 1H), 4.53 – 4.22 (m, 5H), 4.10-3.87 (dd, *J* = 17.6, 6.2 Hz, 2H), 3.91 (dd, *J* = 18.5, 7.9 Hz, 2H), 3.74 – 3.40 (m, 7H), 3.28 (d, *J* = 6.6 Hz, 2H), 3.17 (s, 1H), 3.10 – 2.99 (m, 1H), 2.45 – 2.36 (m, 3H), 2.31 (s, 1H), 2.18-2.07 (m, 2H), 1.90 (d, J = 14.10 Hz, 1H), 1.81 – 1.64 (m, 2H), 1.49 (dq, *J* = 23.3, 7.9, 7.9, 7.4 Hz, 6H), 1.28 (s, 3H), 1.06 (d, *J* = 6.0 Hz, 2H), 1.00 (d, *J* = 6.5 Hz, 3H), 0.91 (t, *J* = 7.23 Hz, 3H), 0.88 (dd, *J* = 12.9, 6.8 Hz, 6H).

**Vanc-38**

**Vanc-38:** ^1^H NMR (300 MHz, DMSO-d6) δ 9.97 (s, 1H), 8.64 (s, 1H), 8.51 (d, *J* = 16.5 Hz, 2H), 8.34 (s, 2H), 8.22 (s, 1H), 7.86 (s, 1H), 7.73 (d, *J* = 8.7 Hz, 3H), 7.58 – 7.47 (m, 6H), 7.45 – 7.39 (m, 3H), 7.34 (d, *J* = 8.3 Hz, 2H), 7.26 (d, *J* = 8.2 Hz, 2H), 6.87 (s, 1H), 6.82 – 6.69 (m, 4H), 6.38 (d, *J* = 14.4 Hz, 2H), 5.76 (d, *J* = 7.7 Hz, 1H), 5.54 (s, 2H), 5.38 – 5.21 (m, 5H), 5.18 – 5.11 (m, 1H), 4.89 (s, 2H), 4.69 (d, *J* = 6.4 Hz, 2H), 4.51 – 4.25 (m, 5H), 4.12-3.90 (m, 4H), 3.73 – 3.42 (m, 6H), 3.29 (s, 2H), 3.17 (s, 1H), 3.06 (t, *J* = 7.2, 7.2 Hz, 1H), 2.31 (s, 3H), 2.15 (dd, *J* = 15.7, 5.4 Hz, 1H), 1.88 (s, 1H), 1.73 (dt, *J* = 14.1, 7.7, 7.7 Hz, 2H), 1.53-1.35 (m, 3H), 1.28 (s, 3H), 1.07 (s, 2H), 1.00 (d, *J* = 6.5 Hz, 4H), 0.88 (dd, *J* = 13.0, 6.5 Hz, 6H).

**Vanc-39**

**Vanc-39:** ^1^H NMR (300 MHz, DMSO-d6) δ 9.63 (s, 1H), 8.65 (s, 1H), 8.52 (s, 1H), 8.39 (s, 1H), 8.31 (s, 2H), 8.24 (s, 1H), 7.86 (s, 1H), 7.80 (s, 1H), 7.51 (dd, *J* = 14.5, 7.2 Hz, 4H), 7.34 (d, *J* = 8.4 Hz, 2H), 7.26 (d, *J* = 7.9 Hz, 2H), 7.13 (d, *J* = 8.3 Hz, 2H), 6.88 (s, 1H), 6.81 – 6.69 (m, 4H), 6.37 (d, *J* = 16.2 Hz, 2H), 5.76 (d, *J* = 7.6 Hz, 1H), 5.54 (s, 1H), 5.37 – 5.12 (m, 7H), 4.89 (s, 2H), 4.74 – 4.63 (m, 2H), 4.51 – 4.25 (m, 6H), 4.10 – 3.82 (m, 4H), 3.69 (d, *J* = 10.9 Hz, 2H), 3.61 – 3.41 (m, 4H), 3.28 (d, *J* = 6.8 Hz, 2H), 3.18 (s, 1H), 3.06 (t, *J* = 7.3, 7.3 Hz, 1H), 2.31 (s, 3H), 2.21 – 2.09 (m, 2H), 1.91 (d, *J* = 12.9 Hz, 1H), 1.78-1.68 (m, 2H), 1.62 – 1.39 (m, 5H), 1.36-1.23 (m, 5H), 1.07 (d, *J* = 6.2 Hz, 3H), 1.00 (d, *J* = 6.5 Hz, 1H), 0.94 – 0.79 (m, 9H).

**Vanc-40**

**Vanc-40:** ^1^H NMR (300 MHz, DMSO-d6) δ 10.06 (s, 1H), 8.64 (s, 1H), 8.52 (d, *J* = 10.7 Hz, 2H), 8.31 (s, 2H), 8.21 (s, 1H), 7.92 – 7.83 (m, 3H), 7.77 (s, 1H), 7.70 (d, *J* = 8.7 Hz, 2H), 7.50 (d, *J* = 8.1 Hz, 2H), 7.34 (d, *J* = 8.4 Hz, 3H), 7.26 (d, J = 8.21 Hz, 2H), 6.92 – 6.62 (m, 4H), 6.38 (d, *J* = 12.7 Hz, 2H), 5.76 (d, *J* = 8.7 Hz, 1H), 5.54 (s, 1H), 5.34 – 5.12 (m, 5H), 4.89 (s, 1H), 4.68 (d, *J* = 5.7 Hz, 1H), 4.51 – 4.25 (m, 4H), 4.14 – 3.89 (m, 3H), 3.69 (d, *J* = 9.5 Hz, 2H), 3.61 – 3.41 (m, 8H), 3.27 (d, *J* = 7.2 Hz, 4H), 3.16 (s, 1H), 3.04 (t, *J* = 7.4, 7.4 Hz, 2H), 2.31 (s, 3H), 2.18-2.11 (m, 2H), 1.89 (d, *J* = 12.6 Hz, 1H), 1.76 – 1.64 (m, 2H), 1.52 – 1.36 (m, 2H), 1.28 (s, 3H), 1.06 (d, *J* = 6.2 Hz, 3H), 0.88 (dd, *J* = 12.9, 6.5 Hz, 6H).

**Vanc-41**

**Vanc-41:** ^1^H NMR (300 MHz, DMSO-d6) δ 9.90 (s, 1H), 8.61 (s, 1H), 8.50 (s, 1H), 8.43 (s, 1H), 8.35 (s, 2H), 8.23 (s, 1H), 8.11 (d, *J* = 8.7 Hz, 1H), 7.94 (d, *J* = 9.0 Hz, 1H), 7.87 (s, 1H), 7.82 – 7.71 (m, 3H), 7.61 – 7.45 (m, 5H), 7.33 (d, *J* = 8.5 Hz, 2H), 7.25 (d, *J* = 8.2 Hz, 2H), 6.88 (s, 1H), 6.80 – 6.67 (m, 3H), 6.62 (d, *J* = 14.8 Hz, 1H), 6.40 – 6.37 (m, 2H), 5.75 (d, *J* = 7.8 Hz, 1H), 5.53 (s, 1H), 5.49 (s, 1H), 5.35 – 5.13 (m, 6H), 4.88 (s, 1H), 4.68 (d, *J* = 6.8 Hz, 2H), 4.42 (d, *J* = 5.1 Hz, 4H), 4.28 (d, *J* = 10.8 Hz, 3H), 4.08 (d, *J* = 11.0 Hz, 2H), 3.72 – 3.41 (m, 7H), 3.28 (s, 2H), 3.17 (s, 1H), 3.09 – 3.02 (m, 1H), 2.31 (s, 3H), 2.14 (dd, *J* = 15.3, 5.2 Hz, 1H), 1.90 (d, *J* = 9.5 Hz, 1H), 1.72 (dt, *J* = 16.5, 8.5, 8.5 Hz, 2H), 1.57 – 1.32 (m, 3H), 1.27 (s, 2H), 1.06 (d, *J* = 5.9 Hz, 2H), 1.00 (d, *J* = 5.4 Hz, 3H), 0.88 (dd, *J* = 12.9, 6.5 Hz, 6H).

**Vanc-42**

**Vanc-42**: ^1^H NMR (600 MHz, DMSO-d6) δ 9.86 (s, 1H), 8.65 (s, 1H), 8.54 (s, 1H), 8.43 (s, 1H), 8.32 (s, 2H), 7.99 (s, 1H), 7.87 (s, 1H), 7.87 (s, 1H), 7.84 – 7.78 (m, 3H), 7.60 (d, *J* = 8.6 Hz, 1H), 7.56 (d, *J* = 7.5 Hz, 1H), 7.53 – 7.49 (m, 2H), 7.39 – 7.33 (m, 4H), 7.28 (t, *J* = 7.1, 7.1 Hz, 3H), 6.89 (s, 1H), 6.79 (d, *J* = 9.6 Hz, 1H), 6.73 (d, *J* = 8.3 Hz, 2H),6.65 (bs, 1H), 6.41-6.38 (m, 2H), 5.77 (d, *J* = 7.9 Hz, 2H), 5.54 (s, 1H), 5.51 (d, *J* = 7.3 Hz, 1H), 5.33 (s, 1H), 5.29 (d, *J* = 7.7 Hz, 1H), 5.24 (s, 2H), 5.16 (s, 1H), 4.89 (s, 1H), 4.69 (q, *J* = 6.6, 6.3, 6.3 Hz, 1H), 4.48 (d, 5.40 Hz, 21), 4.39 (d, *J* = 5.0 Hz, 2H), 4.31 (d, *J* = 11.0 Hz, 1H), 4.10 (dd, *J* = 16.2, 5.6 Hz, 2H), 3.96 (d, *J* = 5.5 Hz, 1H), 3.93 (s, 3H), 3.69 (d, *J* = 10.1 Hz, 2H), 3.65-3.61 (m, 2H), 3.49 – 3.45 (m, 3H), 3.31-3.25 (m, 4H), 3.19 (s, 2H), 3.07 (t, *J* = 7.3, 7.3 Hz, 2H), 2.32 (s, 3H), 2.17-2.13 (m, 1H), 1.91-1.89 (m, 1H), 1.76-1.69 (m, 2H), 1.50 (dt, *J* = 13.4, 6.6, 6.6 Hz, 1H), 1.42 (dt, *J* = 14.0, 7.2, 7.2 Hz, 1H), 1.29 (s, 3H), 1.07 (d, *J* = 6.2 Hz, 3H), 1.01 (d, *J* = 6.5 Hz, 2H), 0.91 (d, *J* = 6.6 Hz, 3H), 0.86 (d, *J* = 6.5 Hz, 3H).

**Vanc-43**

**Vanc-43:** ^1^H NMR (300 MHz, DMSO-d6) δ 9.53 (s, 1H), 8.64 (s, 1H), 8.53 (s, 1H), 8.40 (s, 1H), 8.31 (s, 2H), 8.24 (s, 1H), 8.04 (s, 1H), 7.86 (s, 1H), 7.80 (s, 1H), 7.51 (t, *J* = 8.4, 8.4 Hz, 5H), 7.39 – 7.15 (m, 2H), 7.03 (t, *J* = 9.1, 9.1 Hz, 5H), 688 (s, 1H), 6.80 – 6.65 (m, 5H), 6.37 (d, *J* = 17.0 Hz, 2H), 5.76 (d, *J* = 8.0 Hz, 1H), 5.53 (s, 2H), 5.35 – 5.13 (m, 7H), 4.89 (s, 2H), 4.69 (d, *J* = 6.5 Hz, 2H), 4.51 – 4.25 (m, 6H), 4.05-3.86 (m, 4H), 3.72 – 3.41 (m, 6H), 3.28 (d, *J* = 6.5 Hz, 2H), 3.19 (s, 1H), 3.12 – 3.04 (m, 1H), 2.32 (s, 3H), 2.15 (d, *J* = 10.2 Hz, 2H), 1.91 (d, *J* = 11.3 Hz, 1H), 1.77-1.68 (m, 2H), 1.53-1.36 (m, 3H), 1.29 (s, 2H), 1.07 (d, *J* = 6.2 Hz, 3H), 1.00 (d, *J* = 6.5 Hz, 2H), 0.88 (dd, *J* = 13.1, 6.5 Hz, 6H).

**Vanc-44**

**Vanc-44:** ^1^H NMR (300 MHz, DMSO-d6) δ 10.71 (s, 1H), 8.63 (s, 1H), 8.45 (s, 1H), 8.39 (s, 1H), 8.34 (s, 2H), 8.23 (s, 1H), 8.15 (d, *J* = 9.4 Hz, 1H), 7.94 (d, *J* = 11.2 Hz, 1H), 7.86 (s, 1H), 7.76 (s, 1H), 7.52-7.44 (m, 8.2 Hz, 2H), 7.40 – 7.21 (m, 5H), 6.88 (s, 1H), 6.74 (q, *J* = 8.6, 8.4, 8.4 Hz, 4H), 6.40 (s, 2H), 5.75 (d, *J* = 6.7 Hz, 1H), 5.54 (s, 1H), 5.35 – 5.12 (m, 7H), 4.89 (s, 2H), 4.68 (d, *J* = 7.6 Hz, 2H), 4.41 (d, *J* = 4.6 Hz, 3H), 4.27-4.14 (m, 4H), 4.0-3.90 (m, 3H), 3.72-3.64 (m, 2H), 3.58-3.49 (m, 4H), 3.31-3.25 (s, 4H), 3.13 (s, 1H), 3.07 – 3.00 (m, 1H), 2.31 (s, 2H), 1.89-1.84 (m, 2H), 1.76 – 1.61 (m, 2H), 1.47 (s, 2H), 1.25 (s, 3H), 1.06 (d, *J* = 6.2 Hz, 3H), 1.00 (d, *J* = 6.5 Hz, 1H), 0.88 (dd, *J* = 13.0, 6.5 Hz, 6H).

**Vanc-45**

**Vanc-45:** ^1^H NMR (300 MHz, DMSO-d6) δ 11.79 (s, 1H), 8.85 – 8.68 (m, 1H), 8.60 (s, 1H), 8.44 (s, 1H), 8.32 (s, 2H), 8.08 (s, 1H), 7.87 (s, 1H), 7.76 (s, 1H), 7.55 – 7.19 (m, 9H), 7.15 – 6.99 (m, 2H), 6.88 (s, 1H), 6.82 – 6.62 (m, 4H), 6.36 (s, 1H), 6.26 (s, 1H), 5.75 (d, *J* = 7.5 Hz, 1H), 5.53 (s, 1H), 5.37 – 5.13 (m, 6H), 4.88 (s, 2H), 4.74 – 4.61 (m, 2H), 4.51 – 4.34 (m, 4H), 4.24 (d, *J* = 10.9 Hz, 1H), 3.68 (d, *J* = 10.5 Hz, 2H), 3.60 – 3.41 (m, 5H), 3.39 – 3.15 (m, 8H), 3.10 – 3.00 (m, 1H), 2.31 (s, 3H), 2.20 – 2.09 (m, 2H), 1.89 (s, 2H), 1.80 – 1.61 (m, 4H), 1.53 – 1.34 (m, 3H), 1.27 (s, 3H), 1.06 (d, *J* = 6.2 Hz, 3H), 1.00 (d, *J* = 6.6 Hz, 1H), 0.88 (dd, *J* = 12.9, 6.5 Hz, 6H).

**Vanc-46**

**Vanc-46:** ^1^H NMR (300 MHz, DMSO-d6) δ 8.59 (s, 2H), 8.48 (s, 1H), 8.38 (s, 2H), 8.21 (s, 1H), 8.10 (s, 1H), 7.87 (d, *J* = 6.8 Hz, 3H), 7.56 (t, *J* = 5.6, 5.6 Hz, 2H), 7.52 – 7.30 (m, 9H), 7.25 (d, *J* = 7.90 Hz, 2H), 6.87 (s, 1H), 6.80 – 6.62 (m, 4H), 6.37 (s, 1H), 6.26 (s, 1H), 5.74 (d, *J* = 8.3 Hz, 1H), 5.56 – 5.47 (m, 2H), 5.33 – 5.12 (m, 6H), 4.89 (s, 1H), 4.84 (s, 1H), 4.68 (d, *J* = 5.7 Hz, 1H), 4.45 (s, 1H), 4.40 (d, *J* = 5.1 Hz, 2H), 4.25 (d, *J* = 13.5 Hz, 1H), 3.72 – 3.41 (m, 10H), 3.36 – 3.16 (m, 5H), 3.14 (s, 1H), 3.08 – 3.00 (m, 2H), 2.31 (s, 3H), 2.21 – 2.11 (m, 2H), 1.87 (d, *J* = 15.2 Hz, 2H), 1.78 – 1.62 (m, 3H), 1.54 – 1.35 (m, 3H), 1.26 (s, 2H), 1.06 (d, *J* = 6.1 Hz, 2H), 1.00 (d, *J* = 6.5 Hz, 3H), 0.88 (dd, *J* = 12.9, 6.5 Hz, 6H).

**Vanc-47**

**Vanc-47:** ^1^H NMR (600 MHz, Deuterium Oxide) with DMSO: δ 8.36 (s, 2H), 7.79 (s, 2H), 7.58 (d, *J* = 7.0 Hz, 3H), 7.29 (s, 10H), 7.18 (s, 3H), 7.03 (d, *J* = 9.5 Hz, 1H), 6.91 (d, *J* = 8.3 Hz, 1H), 6.52 (d, *J* = 2.0 Hz, 1H), 6.32 – 6.29 (s, 1H), 5.65 (s, 1H), 5.51 (s, 1H), 5.37 (s, 2H), 5.27 (s, 1H), 4.60 (s, 1H), 4.51 (s, 1H), 4.40 (t, *J* = 8.2, 8.2 Hz, 1H), 4.32 (s, 2H), 4.05 (t, *J* = 7.2, 7.2 Hz, 1H), 3.72 (d, *J* = 8.8 Hz, 2H), 3.56 (s, 2H), 3.39 (s, 1H), 3.27 – 3.08 (m, 4H), 3.03 – 2.91 (m, 3H), 2.71 (s, 3H), 2.07 – 1.94 (m, 2H), 1.77 – 1.57 (m, 4H), 1.39 (s, 2H), 1.14 (d, *J* = 5.7 Hz, 3H), 0.88 – 0.85 (m, 6H).

**Vanc-48**

**Vanc-48:** ^1^H NMR (600 MHz, Deuterium Oxide) δ 8.42 (s, 2H), 7.82 – 7.56 (m, 3H), 7.49 (s, 2H), 7.47 – 7.29 (m, 4H), 7.19 (s, 1H), 7.05 (s, 1H), 6.97 (s, 1H), 6.87 (d, *J* = 12.3 Hz, 1H), 6.60 – 6.47 (m, 1H), 6.36 (s, 1H), 6.10 (d, *J* = 12.5 Hz, 1H), 5.70 (s, 1H), 5.56 – 5.27 (m, 4H), 4.74 (s, 2H), 4.54 (s, 1H), 4.29 (s, 1H), 4.10 (t, *J* = 6.4, 6.4 Hz, 1H), 3.78 (s, 2H), 3.68 – 3.25 (m, 6H), 2.80 (s, 1H), 2.76 (s, 3H), 2.72 – 2.65 (m, 2H), 2.42 (t, *J* = 6.5, 6.5 Hz, 3H), 2.02 (s, 2H), 1.89 – 1.63 (m, 4H), 1.58-1.43 (m, 8H), 1.23 – 1.13 (m, 3H), 1.00 – 0.84 (m, 9H).

**Vanc-49**

**Vanc-49:** ^1^H NMR (300 MHz, DMSO-d6) δ 8.59 (s, 3H), 8.46 (s, 1H), 8.33 (s, 2H), 8.22 (s, 1H), 8.04 (s, 1H), 7.87 (s, 1H), 7.77 (d, *J* = 7.9 Hz, 2H), 7.48 (dd, *J* = 15.0, 8.7 Hz, 2H), 7.38 – 7.22 (m, 6H), 6.88 (s, 1H), 6.79 – 6.65 (m, 4H), 6.36 (s, 1H), 6.27 (s, 1H), 5.75 (d, *J* = 7.9 Hz, 1H), 5.54 (s, 1H), 5.35 – 5.12 (m, 7H), 4.87 (s, 1H), 4.68 (d, *J* = 5.9 Hz, 2H), 4.48 – 4.19 (m, 6H), 3.68 (d, *J* = 10.3 Hz, 4H), 3.60 – 3.49 (m, 3H), 3.36-3.20 (m, 6H), 3.13 (s, 2H), 3.08 – 2.98 (m, 2H), 2.73 (s, 2H), 2.61 (t, J = 7.43, 7.43 Hz, 3H), 2.30 (s, 3H), 1.89-1.84 (m, 2H), 1.75 – 1.64 (m, 4H), 1.56-1.53 (m, 2H), 1.48 (s, 2H), 1.30 – 1.22 (m, 6H), 1.06 (d, *J* = 6.5 Hz, 3H), 0.93 – 0.80 (m, 9H).

**Vanc-50**

**Vanc-50:** ^1^H NMR (300 MHz, DMSO-d6) δ 8.65 – 8.42 (m, 4H), 8.34 (s, 2H), 8.21 (s, 1H), 8.05 (s, 1H), 7.91 – 7.73 (m, 4H), 7.48 (dd, *J* = 14.4, 8.7 Hz, 2H), 7.29 (dt, *J* = 22.2, 6.1, 6.1 Hz, 5H), 6.99 (d, *J* = 8.9 Hz, 2H), 6.87 (s, 1H), 6.78-6.66 (m, 4H), 6.36 (s, 1H), 6.26 (s, 1H), 5.74 (d, *J* = 8.0 Hz, 1H), 5.54-5.49 (m, 2H), 5.37 – 5.11 (m, 6H), 4.87 (s, 2H), 4.68 (d, *J* = 6.9 Hz, 2H), 4.44 (d, *J* = 4.7 Hz, 2H), 4.36 (d, *J* = 5.3 Hz, 2H), 4.24 (d, *J* = 11.7 Hz, 2H), 4.02 (t, *J* = 6.5, 6.5 Hz, 2H), 3.75 – 3.39 (m, 8H), 3.38 – 3.22 (m, 2H), 3.16 (s, 1H), 3.05 (t, J= 7.21, 7.21 Hz, 1H), 2.31 (s, 3H), 1.89 (d, *J* = 11.9 Hz, 1H), 1.76 – 1.63 (m, 5H), 1.43 (dq, *J* = 14.3, 7.1, 6.1, 6.1 Hz, 4H), 1.27 (s, 3H), 1.06 (d, *J* = 6.2 Hz, 3H), 1.00 (d, *J* = 6.5 Hz, 3H), 0.90 (dt, *J* = 19.0, 6.9, 6.9 Hz, 9H).

**Vanc-51**

**Vanc-51:** ^1^H NMR (300 MHz, DMSO-d6) δ 8.62 (s, 2H), 8.44 (s, 1H), 8.30 (s, 2H), 8.14 (s, 1H), 7.87 (s, 1H), 7.80 – 7.64 (m, 4H), 7.51-7.45 (m, 3H), 7.33 (d, *J* = 8.4 Hz, 3H), 7.29 – 7.20 (m, 2H), 6.88 (s, 1H), 6.79 – 6.58 (m, 5H), 6.36 (s, 1H), 6.22 (s, 1H), 5.75 (d, *J* = 7.9 Hz, 1H), 5.54 (s, 1H), 5.38 – 5.10 (m, 7H), 4.88 (s, 2H), 4.68 (d, *J* = 5.8 Hz, 2H), 4.48 – 4.32 (m, 4H), 4.24 (d, *J* = 11.4 Hz, 2H), 3.69 (d, *J* = 10.4 Hz, 2H), 3.60 – 3.39 (m, 5H), 3.39-3.25 (m, 5H), 3.18 (s, 1H), 3.06 (t, J = 7.34, 7.34 Hz, 2H), 2.31 (s, 3H), 2.22 – 2.06 (m, 2H), 1.95-1.87 (s, 1H), 1.80 – 1.64 (m, 2H), 1.46 (ddd, *J* = 26.2, 13.8, 6.0 Hz, 3H), 1.29 (s, 3H), 1.07 (d, *J* = 6.2 Hz, 3H), 0.88 (dd, *J* = 13.1, 6.5 Hz, 6H).

**Vanc-53**

**Vanc-53:** ^1^H NMR (300 MHz, DMSO-d6) δ 8.61 (s, 1H), 8.44 (s, 1H), 8.38 (s, 1H), 8.30 (s, 2H), 8.12 (s, 1H), 7.88 (s, 1H), 7.76 (s, 1H), 7.48 (dd, *J* = 13.9, 7.6 Hz, 5H), 7.40 – 7.20 (m, 7H), 6.88 (s, 1H), 6.81 – 6.62 (m, 4H), 6.58 (d, *J* = 15.8 Hz, 1H), 6.35 (s, 1H), 6.23 (s, 1H), 5.75 (d, *J* = 7.2 Hz, 1H), 5.54 (s, 1H), 5.37 – 5.11 (m, 7H), 4.87 (s, 2H), 4.68 (d, *J* = 7.3 Hz, 2H), 4.47 – 4.31 (m, 4H), 4.23 (d, *J* = 13.1 Hz, 2H), 3.69 (d, *J* = 10.6 Hz, 3H), 3.54 (d, *J* = 7.7 Hz, 2H), 3.40-3.25 (m, 7H), 3.17 (s, 1H), 3.08 – 3.02 (m, 2H), 2.32 (s, 3H), 2.32 (s, 3H), 2.19-2.11 (m, 1H), 1.95 – 1.83 (m, 2H), 1.78 – 1.64 (m, 2H), 1.52 – 1.37 (m, 3H), 1.28 (s, 3H), 1.06 (d, *J* = 6.3 Hz, 3H), 0.88 (dd, *J* = 13.0, 6.5 Hz, 6H).

**Vanc-55**

**Vanc-55:** ^1^H NMR (300 MHz, DMSO-d6) δ 8.61 (s, 1H), 8.42 (s, 1H), 8.29 (s, 3H), 8.06 (s, 1H), 7.85 (s, 1H), 7.66 (d, *J* = 7.8 Hz, 2H), 7.55 – 7.43 (m, 3H), 7.41 – 7.19 (m, 9H), 7.11 – 6.99 (m, 3H), 6.88 (s, 1H), 6.79 – 6.62 (m, 4H), 6.54 – 6.42 (m, 2H), 6.35 (s, 1H), 6.22 (s, 1H), 5.75 (d, *J* = 8.8 Hz, 1H), 5.54 (s, 1H), 5.34 – 5.13 (m, 7H), 4.88 (s, 2H), 4.69 (d, *J* = 7.0 Hz, 2H), 4.47 – 4.32 (m, 4H), 4.22 (d, *J* = 12.7 Hz, 2H), 3.71-3.66 (m, 2H), 3.60 – 3.40 (m, 8H), 3.33 – 3.24 (m, 4H), 3.19 (s, 1H), 3.09 – 3.00 (m, 2H), 2.32 (s, 3H), 2.31 (s, 3H), 2.17-2.09 (m, 1H), 1.90 (s, 2H), 1.74-1.66 (m, 2H), 1.51 – 1.35 (m, 3H), 1.29 (s, 3H), 1.07 (d, *J* = 6.0 Hz, 3H), 0.88 (dd, *J* = 13.0, 6.3 Hz, 6H).

**Vanc-57**

**Vanc-57:** ^1^H NMR (300 MHz, DMSO-d6) δ 9.77 (s, 1H), 8.64 (s, 1H), 8.52 (s, 1H), 8.44 (s, 1H), 8.33 (s, 2H), 8.22 (s, 1H), 7.87 (s, 1H), 7.71 (d, *J* = 8.7 Hz, 2H), 7.59 (d, *J* = 8.6 Hz, 4H), 7.50 (d, *J* = 5.8 Hz, 2H), 7.34 (d, *J* = 8.4 Hz, 3H), 7.26 (d, *J* = 8.4 Hz, 2H), 7.01 (d, *J* = 8.8 Hz, 2H), 6.87 (s, 1H), 6.82 – 6.64 (m, 4H), 6.38 (d, *J* = 11.9 Hz, 2H), 5.76 (d, *J* = 8.1 Hz, 1H), 5.52 (d, *J* = 13.7 Hz, 2H), 5.35 – 5.12 (m, 6H), 4.88 (s, 2H), 4.69 (d, *J* = 6.3 Hz, 2H), 4.38 (td, *J* = 27.4, 25.6, 7.9 Hz, 6H), 4.01 (ddd, *J* = 42.3, 16.2, 5.6 Hz, 4H), 3.79 (s, 3H), 3.74 – 3.40 (m, 6H), 3.28 (d, *J* = 6.4 Hz, 2H), 3.18 (s, 1H), 3.06 (t, *J* = 7.0, 7.0 Hz, 1H), 2.31 (s, 3H), 2.21 – 2.11 (m, 1H), 1.91 (d, *J* = 11.4 Hz, 1H), 1.73 (dt, *J* = 14.6, 7.9, 7.9 Hz, 2H), 1.45 (ddt, *J* = 27.7, 13.7, 7.3, 7.3 Hz, 3H), 1.28 (s, 3H), 1.07 (d, *J* = 6.2 Hz, 3H), 0.88 (dd, *J* = 12.9, 6.5 Hz, 6H).

**Vanc-59**

**Vanc-59:** ^1^H NMR (300 MHz, DMSO-d6) δ 10.03 (s, 1H), 8.65 (s, 1H), 8.55 (s, 2H), 8.49 (d, *J* = 9.00 Hz, 2H), 8.31 (s, 3H), 8.23 (bs, 1H), 8.12 – 8.01 (m, 3H), 7.88 (s, 1H), 7.78 (s, 1H), 7.63 (d, *J* = 9.1 Hz, 1H), 7.56 – 7.45 (m, 4H), 7.35 (d, *J* = 8.26 Hz, 3H), 7.27 (d, *J* = 8.72 Hz, 2H), 6.92 – 6.63 (m, 5H), 6.41 (d, *J* = 3.51 Hz, 2H), 5.76 (d, *J* = 8.0 Hz, 1H), 5.58 – 5.47 (m, 2H), 5.39 – 5.12 (m, 6H), 4.89 (s, 1H), 4.69 (d, *J* = 6.5 Hz, 2H), 4.53 – 4.26 (m, 6H), 4.22 – 4.08 (m, 2H), 4.04-3.96 (m, 2H), 3.75 – 3.42 (m, 7H), 3.32-3.24 (m, 2H), 3.18 (s, 1H), 3.06 (t, J = 7.07, 7.07 Hz, 1H), 2.31 (s, 3H), 2.22 – 2.11 (m, 1H), 1.93 (s, 1H), 1.77 – 1.64 (m, 2H), 1.55 – 1.34 (m, 3H), 1.28 (s, 3H), 1.07 (d, *J* = 6.2 Hz, 3H), 0.88 (dd, *J* = 12.9, 6.5 Hz, 6H).

**Vanc-63**

Vanc-63: ^1^H NMR of Vanc 63 (300 MHz, DMSO) δ 8.68 (s, 2H), 8.59 (s, 1H), 8.46 (s, 1H), 8.35 (s, 2H), 8.27 (d, J = 8.6 Hz, 2H), 8.08 (s, 2H), 7.81 (dd, J = 19.8, 11.7 Hz, 7H), 7.70 (d, J = 8.7 Hz, 2H), 7.48 (d, J = 7.0 Hz, 6H), 7.29 (dd, J = 19.1, 8.5 Hz, 5H), 6.87 (s, 1H), 6.73 (d, J = 9.4 Hz, 3H), 6.31 (d, J = 28.4 Hz, 2H), 5.76 (s, 2H), 5.54 (s, 1H), 5.37 – 5.11 (m, 5H), 4.92 – 4.81 (m, 1H), 4.67 (s, 2H), 4.32 (d, J = 32.8 Hz, 5H), 2.85 – 2.76 (m, 4H), 2.73 (s, 3H), 2.31 (s, 4H), 2.07 (s, 2H), 1.75 (d, J = 25.2 Hz, 6H), 1.51 – 1.35 (m, 5H), 1.27 (s, 4H), 1.06 (d, J = 5.7 Hz, 2H), 1.00 (d, J = 6.5 Hz, 2H), 0.88 (dd, J = 12.9, 6.5 Hz, 6H).

**Vanc-64**

Vanc-64: ^1^H NMR of Vanc 64 (300 MHz, DMSO): δ 8.61 (s, 2H), 8.45 – 8.38 (m, 2H), 8.25 (d, J = 9.5 Hz, 2H), 7.94 (d, J = 8.0 Hz, 2H), 7.80 (s, 2H), 7.60 (s, 1H), 7.54 – 7.42 (m, 4H), 7.41 – 7.13 (m, 7H), 6.93 – 6.58 (m, 6H), 6.26 (dd, J = 38.8, 14.0 Hz, 4H), 5.73 (s, 3H), 5.54 (s, 2H), 5.32-5.14 (m, 7H), 4.88 (s, 2H), 4.71 (s, 2H), 4.45-4.34 (s, 5H), 4.26-4.20 (s, 3H), 3.05-3.02 (m, 2H), 2.31 (s, 4H), 2.18-2.13 (m, 3H), 2.07 (2H), 1.97-1.90 (m, 4H), 1.70 (s, 3H), 1.42 (s, 3H), 1.31-1.24 (m, 3H), 1.08 (d, J = 5.9 Hz, 3H), 1.00 (d, J = 6.5 Hz, 2H), 0.88 (dd, J = 12.9, 6.4 Hz, 6H).

**Vanc-65**

Vanc-65: ^1^H NMR (300 MHz, DMSO-d6): δ 8.73 – 8.64 (m, 1H), 8.59 (s, 1H), 8.45 (s, 1H), 8.37 (s, 2H), 8.28 (s, 1H), 8.18 (s, 1H), 8.05 (s, 1H), 7.90 – 7.65 (m, 7H), 7.55 – 7.41 (m, 3H), 7.38 – 7.19 (m, 7H), 6.87 (s, 1H), 6.80 – 6.61 (m, 4H), 6.37 – 6.21 (m, 3H), 5.74 (d, J = 8.7 Hz, 2H), 5.60 – 5.42 (m, 2H), 5.37 – 5.06 (m, 6H), 4.87 (s, 2H), 4.68 (d, J = 6.1 Hz, 2H), 4.52 – 4.33 (m, 4H), 4.31 – 4.15 (m, 2H), 3.65 (d, J = 8.5 Hz, 3H), 3.04 (d, J = 7.2 Hz, 2H), 2.79 – 2.68 (m, 1H), 2.29 (d, J = 7.1 Hz, 7H), 2.17 (d, J = 5.7 Hz, 3H), 2.07 (s, 2H), 1.84 – 1.57 (m, 5H), 1.54 – 1.37 (m, 4H), 1.24 (s, 4H), 1.06 (d, J = 6.3 Hz, 3H), 1.00 (d, J = 6.5 Hz, 3H), 0.88 (dd, J = 12.8, 6.5 Hz, 6H)

**Vanc-66**

**Vanc-66:** ^1^H NMR (600 MHz, DMSO-d6): δ 8.35 (s, 3H), 8.31 (s, 1H), 7.93 (d, *J* = 9.1 Hz, 1H), 7.85 (s, 3H), 7.79 (d, *J* = 8.3 Hz, 2H), 7.63 (s, 1H), 7.55 (d, *J* = 9.1 Hz, 1H), 7.50 (d, *J* = 8.4 Hz, 1H), 7.42 (d, *J* = 8.4 Hz, 3H), 7.40 – 7.38 (m, 1H), 7.32 (d, *J* = 8.3 Hz, 1H), 7.26 (d, *J* = 8.2 Hz, 2H), 7.23 (s, 1H), 6.83 (d, *J* = 8.8 Hz, 1H), 6.77 (d, *J* = 8.5 Hz, 1H), 6.40 (d, *J* = 2.3 Hz, 1H), 6.28 (d, *J* = 2.0 Hz, 1H), 5.69 (s, 1H), 5.56 (s, 1H), 5.37 (s, 1H), 5.32 (d, *J* = 7.4 Hz, 1H), 5.26-5.21 (m, 3H), 4.91 (s, 1H), 4.69 (d, *J* = 6.5 Hz, 1H), 4.47 (s, 1H), 4.38 (s, 2H), 4.26 (s, 1H), 3.70-3.48 (m, 6H), 3.42-3.39 (m, 2H), 3.31 (s, 4H), 3.23 (d, *J* = 14.7 Hz, 3H), 3.19 (s, 13H), 2.90 (p, *J* = 7.0, 7.0, 6.9, 6.9 Hz, 1H), 2.41 (s, 3H), 1.94 (d, 9.63 Hz, 1H), 1.83 – 1.74 (m, 3H), 1.66 (dt, *J* = 13.1, 6.6, 6.6 Hz, 1H), 1.56 (dt, *J* = 13.1, 6.8, 6.8 Hz, 1H), 1.48 (dt, *J* = 13.9, 7.0, 7.0 Hz, 1H), 1.34 (s, 3H), 1.14 (d, *J* = 6.9 Hz, 6H), 1.08 (d, *J* = 6.7 Hz, 4H), 1.02 (d, *J* = 6.5 Hz, 3H), 0.91 (d, *J* = 6.4 Hz, 3H), 0.86 (d, *J* = 6.4 Hz, 3H).

**Vanc-69**

**Vanc-69:** ^1^H NMR (300 MHz, DMSO-d6) δ 9.84 (s, 2H), 8.63 (s, 1H), 8.52 (s, 1H), 8.40 (s, 3H), 8.22 (s, 1H), 7.85 (s, 1H), 7.73 (s, 2H), 7.63 (d, J = 8.9 Hz, 2H), 7.56 – 7.45 (m, 2H), 7.34 (d, J = 8.6 Hz, 3H), 7.25 (d, J = 8.3 Hz, 2H), 6.97 – 6.60 (m, 6H), 6.38 (d, J = 13.0 Hz, 2H), 5.75 (d, J = 7.5 Hz, 2H), 5.52 (d, J = 9.5 Hz, 2H), 5.37 – 5.11 (m, 5H), 4.88 (s, 2H), 4.68 (d, J = 5.0 Hz, 5H), 4.52 – 4.22 (m, 5H), 4.14 – 3.80 (m, 4H), 3.73 – 3.57 (m, 2H), 3.10 – 2.99 (m, 4H), 2.43 – 2.22 (m, 5H), 2.32 (s, 1H), 2.08 (s, 2H), 1.79 – 1.34 (m, 10H), 1.34 – 1.18 (m, 3H), 1.10 – 0.93 (m, 7H), 0.88 (dd, J = 12.7, 6.5 Hz, 6H).

**Vanc-70**

**Vanc-70:** ^1^H NMR of Vanc 70 (600 MHz, D_2_O) δ 8.36 (s, 2H), 7.55 (s, 2H), 7.44 – 7.23 (m, 8H), 6.90 (s, 2H), 6.50 (d, J = 20.7 Hz, 2H), 5.63 (s, 1H), 5.37 (s, 2H), 5.25 (s, 2H), 4.74 – 4.61 (m, 3H), 4.07 (s, 2H), 3.86 (s, 1H), 3.65 (dd, J = 24.2, 17.6 Hz, 4H), 3.56 (s, 1H), 3.38 (s, 1H), 2.72 (s, 1H), 2.61 (dd, J = 5.6, 3.9 Hz, 11H), 1.99 (d, J = 23.8 Hz, 3H), 1.65 (d, J = 42.6 Hz, 6H), 1.39 (d, J = 31.5 Hz, 6H), 1.11 (s, 5H), 1.03 (d, J = 6.5 Hz, 5H), 0.88 (s, 7H), 0.84 (s, 8H), 0.70 (s, 4H).

**Vanc-72**

**Vanc-72:** ^1^H NMR (600 MHz, DMSO-d6) δ 9.85 (s, 1H), 9.12 (s, 1H), 8.68 – 8.43 (m, 3H), 8.34 (s, 2H), 8.22 (s, 1H), 7.90 (d, *J* = 8.45 Hz, 2H), 7.86 (d, J = 9.01 Hz, 3Hz), 7.79 (d, J = 8.48 Hz, 2H), 7.51 (d, *J* = 6.4 Hz, 2H), 7.36 (d, *J* = 8.3 Hz, 2H), 7.30 – 7.24 (m, 3H), 7.18 (d, *J* = 9.0 Hz, 2H), 6.88 (s, 1H), 6.79 (t, *J* = 9.4, 9.4 Hz, 2H), 6.73 (d, *J* = 8.4 Hz, 1H), 6.67 (s, 1H), 6.41 (s, 1H), 6.37 (s, 1H), 5.77 (d, *J* = 7.9 Hz, 1H), 5.53 (d, *J* = 20.2 Hz, 2H), 5.34 (s, 1H), 5.29 (d, *J* = 7.7 Hz, 1H), 5.24 (s, 2H), 5.16 (s, 1H), 4.89 (s, 1H), 4.69 (d, *J* = 6.4 Hz, 1H), 4.50 – 4.48 (m, 1H), 4.38 (d, *J* = 4.7 Hz, 1H), 4.31 (s, 1H), 4.09 (dd, *J* = 16.0, 5.4 Hz, 1H), 3.98-3.90 (s, 1H), 3.86 (s, 3H), 3.69 (d, *J* = 10.2 Hz, 2H), 3.59 – 3.51 (m, 6H), 3.49 – 3.44 (m, 3H), 3.39 (dd, *J* = 14.0, 7.0 Hz, 3H), 3.29 (dt, *J* = 18.0, 9.7, 9.7 Hz, 4H), 3.17 (s, 1H), 3.10 – 3.03 (m, 1H), 2.32 (s, 3H), 2.19 – 2.12 (m, 1H), 1.90 (d, *J* = 9.1 Hz, 1H), 1.72 (dt, *J* = 20.2, 10.0, 10.0 Hz, 2H), 1.49 (dt, *J* = 13.5, 6.8, 6.8 Hz, 1H), 1.41 (dt, *J* = 13.7, 7.2, 7.2 Hz, 1H), 1.29 (s, 3H), 1.07 (d, *J* = 6.2 Hz, 3H), 0.91 (d, *J* = 6.5 Hz, 3H), 0.87 (d, *J* = 6.5 Hz, 3H).

**Vanc-73**

**Vanc-73:** 1H NMR of Vanc 73 (300 MHz, DMSO) δ 8.61 (s, 3H), 8.48 (s, 1H), 8.33 (s, 3H), 8.23 (s, 1H), 8.08 (s, 1H), 7.87 (s, 2H), 7.80 (d, J = 8.4 Hz, 3H), 7.56 – 7.41 (m, 5H), 7.38 – 7.20 (m, 5H), 6.87 (s, 1H), 6.73 (dd, J = 17.2, 8.7 Hz, 4H), 6.31 (d, J = 31.4 Hz, 3H), 5.75 (d, J = 7.4 Hz, 1H), 5.53 (s, 2H), 5.39 – 5.10 (m, 7H), 4.89 (s, 2H), 4.68 (d, J = 6.2 Hz, 3H), 4.46 – 4.33 (m, 3H), 4.24 (d, J = 11.4 Hz, 2H), 3.68 (d, J = 10.2 Hz, 2H), 3.51 (dd, J = 20.8, 7.8 Hz, 4H), 3.36 – 3.01 (m, 9H), 2.32 (s, 3H), 1.81 – 1.60 (m, 4H), 1.29 (s, 12H), 1.06 (d, J = 6.2 Hz, 3H), 1.00 (d, J = 6.5 Hz, 1H), 0.88 (dd, J = 13.1, 6.5 Hz, 6H).

**Vanc-74**

**Vanc-74:** ^1^H NMR (300 MHz, DMSO-d6) δ 9.63 (s, 1H), 8.64 (s, 1H), 8.51 (d, *J* = 3.7 Hz, 1H), 8.41 (s, 1H), 8.34 (s, 2H), 8.22 (s, 1H), 7.86 (s, 1H), 7.77 (s, 1H), 7.51 (dd, *J* = 14.7, 7.2 Hz, 4H), 7.34 (d, *J* = 8.2 Hz, 2H), 7.26 (d, *J* = 7.7 Hz, 2H), 7.13 (d, *J* = 8.4 Hz, 2H), 6.88 (s, 1H), 6.81 – 6.68 (m, 4H), 6.37 (d, *J* = 17.5 Hz, 2H), 5.76 (d, *J* = 7.8 Hz, 1H), 5.53 (s, 1H), 5.37 – 5.11 (m, 6H), 4.89 (s, 2H), 4.68 (d, *J* = 6.4 Hz, 2H), 4.50 – 4.23 (m, 6H), 3.97 (ddd, *J* = 42.6, 17.0, 6.1 Hz, 4H), 3.74 – 3.39 (m, 7H), 3.29 (s, 3H), 3.17 (s, 1H), 3.08 – 3.03 (m, 2H), 2.31 (s, 3H), 2.14 (d, *J* = 13.8 Hz, 2H), 1.90 (d, *J* = 10.3 Hz, 1H), 1.76 – 1.66 (m, 2H), 1.62 – 1.39 (m, 5H), 1.28 (s, 6H), 1.06 (d, *J* = 6.2 Hz, 3H), 0.95 – 0.76 (m, 9H).

**Vanc-75**

**Vanc-75:** ^1^H NMR (600 MHz, Deuterium Oxide) δ 8.36 (s, 1H), 7.73 (s, 1H), 7.60 (d, *J* = 7.2 Hz, 1H), 7.54 (d, *J* = 7.3 Hz, 2H), 7.32 (d, *J* = 6.3 Hz, 2H), 7.27 (s, 1H), 7.23 – 7.19 (m, 2H), 7.13 (s, 1H), 7.01 (s, 2H), 6.93 (s, 2H), 6.54 (s, 1H), 6.50 (s, 1H), 5.62 (s, 1H), 5.48 (s, 1H), 5.41 (s, 1H), 5.35 (s, 1H), 5.29 (s, 1H), 5.25 (s, 1H), 4.70 – 4.61 (m, 8H), 4.09 (d, *J* = 27.0 Hz, 2H), 3.94 (s, 2H), 3.72-3.62 (m, 4H), 3.39 (s, 1H), 2.65 (s, 2H), 2.55 (s, 2H), 2.06-1.93 (m, 2H), 1.71 (s, 1H), 1.62 (d, *J* = 3.7 Hz, 2H), 1.52 (s, 2H), 1.37 (s, 2H), 1.22 (s, 4H), 1.12 (d, *J* = 3.8 Hz, 3H), 1.03 (d, *J* = 6.6 Hz, 3H), 0.89 (d, *J* = 5.3 Hz, 3H), 0.84 (d, *J* = 4.8 Hz, 3H), 0.78 (s, 3H).

**Vanc-76**

**Vanc-76:** ^1^H NMR of Vanc 76 (300 MHz, DMSO) δ 9.84 (s, 1H), 9.17 (s, 1H), 8.64 (s, 1H), 8.54 (s, 1H), 8.44 (s, 1H), 8.38 (s, 1H), 8.21 (s, 1H), 7.82 (ddd, J = 40.9, 25.3, 17.4 Hz, 7H), 7.47 (dd, J = 19.9, 8.3 Hz, 4H), 7.30 (dd, J = 25.7, 8.6 Hz, 4H), 6.78 (dd, J = 30.8, 18.2 Hz, 5H), 6.39 (d, J = 10.4 Hz, 2H), 5.76 (d, J = 7.6 Hz, 2H), 5.54 (s, 1H), 5.36 – 5.26 (m, 2H), 5.24 (s, 2H), 5.15 (s, 1H), 4.87 (s, 1H), 4.68 (d, J = 6.6 Hz, 1H), 4.49 (s, 1H), 4.38 (d, J = 4.8 Hz, 2H), 4.35 – 4.23 (m, 2H), 4.02 (dd, J = 38.8, 11.2 Hz, 3H), 3.69 (d, J = 10.8 Hz, 3H), 3.53 (s, 5H), 3.12 – 3.00 (m, 5H), 2.73 (s, 1H), 2.40 (s, 3H), 2.31 (s, 3H), 2.16 (s, 1H), 2.08 (d, J = 4.0 Hz, 2H), 1.86 (d, J = 15.8 Hz, 1H), 1.69 (dd, J = 20.1, 10.1 Hz, 3H), 1.45 (d, J = 11.8 Hz, 2H), 1.26 (d, J = 10.1 Hz, 4H), 1.03 (dd, J = 18.3, 6.4 Hz, 4H), 0.88 (dd, J = 12.8, 6.5 Hz, 6H).

**Vanc-77**

**Vanc-77:** ^1^H NMR (600 MHz, DMSO-d6) δ 9.87 (s, 1H), 9.24 (s, 1H), 8.66 (s, 1H), 8.54 (s, 1H), 8.47 (s, 1H), 8.33 (s, 2H), 8.23 (s, 1H), 7.96 (d, *J* = 7.6 Hz, 2H), 7.92 (d, *J* = 8.6 Hz, 2H), 7.87 (s, 1H), 7.80 (d, *J* = 8.7 Hz, 2H), 7.65 (t, *J* = 7.9, 7.9 Hz, 2H), 7.56 – 7.47 (m, 3H), 7.36 (d, *J* = 8.3 Hz, 3H), 7.33 – 7.21 (m, 2H), 6.89 (s, 1H), 6.79 (t, *J* = 7.8, 7.8 Hz, 2H), 6.73 (d, *J* = 8.5 Hz, 1H), 6.67 (s, 1H), 6.41 (s, 1H), 6.37 (s, 1H), 5.77 (d, *J* = 8.1 Hz, 1H), 5.54 (s, 1H), 5.34 (s, 1H), 5.29 (d, *J* = 7.8 Hz, 2H), 5.24 (s, 2H), 5.16 (s, 1H), 4.89 (s, 1H), 4.69 (d, *J* = 6.3 Hz, 2H), 4.48 (s, 1H), 4.38 (d, *J* = 5.0 Hz, 2H), 4.32 (d, *J* = 11.7 Hz, 1H), 4.09 (dd, *J* = 17.0, 6.6 Hz, 2H), 3.97 (dd, *J* = 16.5, 5.3 Hz, 2H), 3.69 (d, *J* = 10.5 Hz, 2H), 3.58 – 3.51 (m, 4H), 3.49 – 3.44 (m, 3H), 3.29 (dt, *J* = 17.9, 8.7, 8.7 Hz, 4H), 3.18 (s, 2H), 3.06 (t, *J* = 6.9, 6.9 Hz, 1H), 2.32 (s, 3H), 2.14 (s, 1H), 1.91 (d, *J* = 10.0 Hz, 1H), 1.76 – 1.67 (m, 2H), 1.52 – 1.46 (m, 1H), 1.42 (dt, *J* = 13.8, 7.5, 7.5 Hz, 1H), 1.29 (s, 3H), 1.07 (d, *J* = 6.2 Hz, 3H), 0.91 (d, *J* = 6.6 Hz, 3H), 0.86 (d, *J* = 6.6 Hz, 3H).

**Vanc-78**

**Vanc-78:** ^1^H NMR (600 MHz, DMSO-d6) δ 9.85 (s, 1H), 9.10 (s, 2H), 8.87 (s, 1H), 8.65 (s, 1H), 8.54 (s, 1H), 8.40 (s, 1H), 8.35 (s, 2H), 7.87 (s, 1H), 7.81 (d, *J* = 8.5 Hz, 2H), 7.76 (d, *J* = 8.6 Hz, 2H), 7.53 – 7.48 (m, 2H), 7.39 – 7.32 (m, 2H), 7.30 – 7.24 (m, 2H), 6.89 (s, 1H), 6.80 (d, *J* = 9.3 Hz, 2H), 6.73 (d, *J* = 8.4 Hz, 1H), 6.66 (s, 1H), 6.44 – 6.36 (m, 3H), 5.76 (d, *J* = 7.7 Hz, 1H), 5.68 (t, *J* = 9.3, 9.3 Hz, 1H), 5.62 (t, *J* = 9.5, 9.5 Hz, 1H), 5.55 (s, 1H), 5.49 (d, *J* = 7.9 Hz, 1H), 5.34 – 5.28 (m, 2H), 5.26 – 5.13 (m, 4H), 4.89 (s, 1H), 4.69 (d, *J* = 6.7 Hz, 2H), 4.50 – 4.32 (m, 1H), 4.34 – 4.25 (m, 2H), 4.19-4.07 (m, 5H), 3.95 (dd, *J* = 16.0, 5.0 Hz, 2H), 3.74 – 3.60 (m, 4H), 3.58-3.47 m, 9H), 3.11 (s, 1H), 3.07 – 3.02 (m, 1H), 2.31 (s, 3H), 2.05 (s, 3H), 2.02 (s, 3H), 1.99 (s, 3H), 1.83 (s, 3H), 1.76-1.72 (m, 1H), 1.67 (d, *J* = 12.9 Hz, 1H), 1.50-1.46(m, 1H), 1.44-1.38 (m, 1H), 1.25 (s, 3H), 1.07 (d *J* = 6.30 Hz, 3H), 1.01 (d, *J* = 6.6 Hz, 4H), 0.91 (d, *J* = 6.5 Hz, 3H), 0.87 (d, *J* = 6.6 Hz, 3H).

**Vanc-80**

**Vanc-80:** ^1^H NMR (600 MHz, DMSO-d6) δ 9.90 (s, 1H), 9.15 (s, 1H), 8.74 – 8.48 (m, 4H), 8.40 (s, 3H), 7.89 – 7.80 (m, 3H), 7.74 (d, *J* = 8.6 Hz, 2H), 7.55 – 7.45 (m, 2H), 7.41 – 7.23 (m, 4H), 6.88 (s, 1H), 6.79 (d, *J* = 9.0 Hz, 1H), 6.75 – 6.62 (m, 2H), 6.41 (s, 1H), 6.33 (s, 1H), 5.76 (s, 1H), 5.54 (s, 1H), 5.50 (d, *J* = 8.3 Hz, 1H), 5.34 (s, 1H), 5.30 (d, *J* = 7.6 Hz, 1H), 5.26 – 5.14 (m, 3H), 4.88 (s, 1H), 4.69 (s, 1H), 4.48 (s, 1H), 4.43 – 4.26 (m, 3H), 4.09 – 3.93 (m, 3H), 3.70-3.63 (m, 3H), 3.60 – 3.49 (m, 10H), 3.25-3.20 (m, 6H), 3.17 (s, 1H), 3.07 (s, 1H), 3.03 (s, 1H), 3.00 (s, 6H), 2.39 (s, 1H), 2.31 (s, 3H), 2.18 – 2.11 (m, 1H), 1.98 – 1.90 (m, 2H), 1.84 – 1.79 (m, 1H), 1.76-1.70 (m, 1H), 1.68 – 1.60 (m, 3H), 1.49 (dt, *J* = 13.7, 6.8, 6.8 Hz, 1H), 1.41 (dt, *J* = 14.6, 7.8, 7.8 Hz, 1H), 1.32 (p, *J* = 7.1, 7.1, 6.9, 6.9 Hz, 2H), 1.27 – 1.21 (m, 4H), 1.06 (d, *J* = 6.3 Hz, 3H), 1.01 (d, *J* = 6.5 Hz, 3H), 0.91 (d, *J* = 7.5 Hz, 3H), 0.88 (d, *J* = 7.4 Hz, 3H), 0.86 (d, *J* = 7.5 Hz, 3H).

**Vanc-81**

**Vanc-81:** ^1^H NMR of Vanc 81 (300 MHz, DMSO) δ 9.92 (s, 2H), 8.58 (d, J = 30.9 Hz, 4H), 8.39 (s, 2H), 7.79 (dd, J = 30.3, 9.8 Hz, 5H), 7.50 (s, 2H), 7.39 – 7.23 (m, 3H), 6.77 (dd, J = 32.1, 19.4 Hz, 5H), 6.36 (d, J = 28.7 Hz, 3H), 5.74 (s, 2H), 5.53 (s, 2H), 5.37 – 5.11 (m, 5H), 4.78 (d, J = 56.9 Hz, 5H), 4.40 (d, J = 43.7 Hz, 4H), 3.99 (s, 5H), 3.45 (dd, J = 90.4, 25.4 Hz, 25H), 3.00 (s, 5H), 2.30 (s, 4H), 2.07 (s, 2H), 1.75 (d, J = 76.3 Hz, 8H), 1.46 (s, 2H), 1.25 (s, 8H), 1.05 (d, J = 6.0 Hz, 2H), 1.00 (d, J = 6.5 Hz, 1H), 0.88 (dd, J = 13.2, 6.2 Hz, 6H).

**Vanc-83**

**Vanc-83:** ^1^H NMR (600 MHz, DMSO-d6) δ 9.83 (s, 1H), 8.65 (s, 1H), 8.53 (s, 1H), 8.44 (s, 1H), 8.32 (s, 2H), 7.87 (s, 1H), 7.76 (d, *J* = 8.6 Hz, 3H), 7.66 (dd, *J* = 7.9, 3.6 Hz, 4H), 7.51 (t, *J* = 7.7, 7.7 Hz, 2H), 7.46 (t, *J* = 7.7, 7.7 Hz, 2H), 7.34 (dt, *J* = 9.6, 5.5, 5.5 Hz, 4H), 7.27 (d, *J* = 8.7 Hz, 2H), 6.88 (s, 1H), 6.79 (t, *J* = 9.61 Hz, 1H), 6.76-6.72 (m, 2H), 6.67 (bs, 1H), 6.41 (s, 1H), 6.38 (s, 1H), 5.77 (d, *J* = 7.9 Hz, 1H), 5.55 (s, 1H), 5.33 (s, 1H), 5.29 (d, *J* = 7.7 Hz, 1H), 5.24 (s, 2H), 5.16 (s, 1H), 4.89 (s, 1H), 4.69 (d, *J* = 6.4 Hz, 1H), 4.49 – 4.47 (m, 1H), 4.39 (d, *J* = 4.9 Hz, 2H), 4.31 (d, *J* = 11.2 Hz, 1H), 4.09 (dd, *J* = 16.2, 5.6 Hz, 2H), 3.95 (dd, *J* = 16.1, 5.4 Hz, 2H), 3.69 (d, *J* = 10.2 Hz, 2H), 3.58 – 3.51 (m, 5H), 3.49 – 3.44 (m, 3H), 3.39 (dd, *J* = 14.1, 7.1 Hz, 2H), 3.29 (dt, *J* = 17.9, 9.4, 9.4 Hz, 4H), 3.18 (s, 1H), 3.06 (t, *J* = 7.0, 7.0 Hz, 1H), 2.32 (s, 3H), 2.16 (dd, *J* = 16.1, 5.8 Hz, 1H), 1.90 (t, *J* = 8.1, 8.1 Hz, 1H), 1.77 – 1.68 (m, 2H), 1.49 (dt, *J* = 13.9, 6.9, 6.9 Hz, 1H), 1.42 (dt, *J* = 13.9, 7.3, 7.3 Hz, 1H), 1.29 (s, 3H), 1.07 (d, *J* = 6.4 Hz, 3H), 0.91 (d, *J* = 6.4 Hz, 3H), 0.86 (d, *J* = 6.6 Hz, 3H).

**Vanc-84**

**Vanc-84:** ^1^H NMR (300 MHz, DMSO-d6) δ 9.85 (s, 1H), 8.65 (s, 1H), 8.53 (s, 1H), 8.45 (s, 1H), 8.37 – 8.32 (m, 2H), 8.22 (s, 1H), 7.87 (s, 1H), 7.81 – 7.64 (m, 7H), 7.50 (d, *J* = 6.4 Hz, 4H), 7.41 – 7.21 (m, 4H), 6.94 – 6.64 (m, 5H), 6.39 (d, *J* = 12.8 Hz, 2H), 5.76 (d, *J* = 7.4 Hz, 1H), 5.54 (s, 1H), 5.36 – 5.12 (m, 6H), 4.88 (s, 1H), 4.68 (s, 1H), 4.52 – 4.25 (m, 5H), 4.17 – 3.88 (m, 4H), 3.73 – 3.41 (m, 9H), 3.29 (s, 4H), 3.15 (s, 1H), 3.07-3.03 (m, 1H), 2.31 (s, 3H), 2.21 – 2.03 (m, 1H), 1.92 – 1.84 (m, 1H), 1.78 – 1.62 (m, 2H), 1.53 – 1.35 (m, 2H), 1.27 (s, 3H), 1.09-1.04 (3, 3H), 0.93 – 0.83 (m, 6H).

**Vanc-85**

**Vanc-85:** ^1^H NMR (300 MHz, DMSO-d6) δ 9.90 (s, 1H), 8.65 (s, 1H), 8.51 (d, *J* = 16.1 Hz, 2H), 8.34 (s, 2H), 8.23 (s, 1H), 8.03 (d, *J* = 8.4 Hz, 2H), 7.91 – 7.73 (m, 8H), 7.50 (d, *J* = 7.7 Hz, 2H), 7.35 (d, *J* = 8.0 Hz, 2H), 7.26 (d, *J* = 8.3 Hz, 2H), 6.88 (s, 1H), 6.80-6.66 (m, 4H), 6.38 (d, *J* = 12.0 Hz, 2H), 5.76 (d, *J* = 8.3 Hz, 1H), 5.61 – 5.46 (m, 1H), 5.37 – 5.12 (m, 6H), 4.88 (s, 1H), 4.69 (d, *J* = 6.0 Hz, 1H), 4.52 – 4.25 (m, 5H), 4.15 – 3.89 (m, 5H), 3.73 – 3.39 (m, 9H), 3.27 (d, *J* = 7.2 Hz, 3H), 3.17 (s, 1H), 3.08 – 3.02 (m, 1H), 2.61 (s, 3H), 2.31 (s, 3H), 2.20-2.13 (d, *J* = 10.8 Hz, 1H), 1.93 – 1.82 (m, 1H), 1.75-1.67 (m, 2H), 1.55 – 1.35 (m, 2H), 1.27 (s, 3H), 1.06 (d, *J* = 6.0 Hz, 3H), 0.90 (d, *J* = 6.4 Hz, 3H), 0.86 (d, *J* = 6.4 Hz, 3H).

**Vanc-86**

**Vanc-86:** ^1^H NMR (300 MHz, DMSO-d6) δ 9.78 (s, 1H), 8.64 (s, 1H), 8.54 (s, 1H), 8.43 (s, 1H), 8.35 (s, 2H), 8.21 (s, 1H), 7.86 (s, 1H), 7.71 (d, *J* = 8.6 Hz, 3H), 7.62 – 7.45 (m, 6H), 7.34 (d, *J* = 8.3 Hz, 2H), 7.26 (d, *J* = 8.2 Hz, 2H), 6.99 (d, *J* = 8.7 Hz, 2H), 6.88 (s, 1H), 6.80-6.66 (m, 4H), 6.38 (d, *J* = 12.1 Hz, 2H), 5.76 (d, *J* = 8.2 Hz, 1H), 5.54 (s, 1H), 5.37 – 5.09 (m, 6H), 4.88 (s, 1H), 4.69 (d, *J* = 6.1 Hz, 2H), 4.51 – 4.20 (m, 7H), 4.06 (q, *J* = 6.9, 6.9, 6.9 Hz, 4H), 3.94 (dd, *J* = 16.5, 4.8 Hz, 2H), 3.69 (d, *J* = 10.8 Hz, 2H), 3.59-3.43 (m, 5H), 3.32 – 3.23 (m, 3H), 3.17 (s, 1H), 3.09 – 3.02 (m, 1H), 2.31 (s, 3H), 2.21 – 2.10 (m, 1H), 1.93-1.88 (m, 1H), 1.77-1.65 (m, 2H), 1.44-1.40 (m, 2H), 1.34 (t, *J* = 6.9, 6.9 Hz, 3H), 1.28 (s, 3H), 1.06 (d, *J* = 6.1 Hz, 3H), 0.90 (d, *J* = 6.5 Hz, 3H), 0.86 (d, *J* = 6.5 Hz, 3H).

**Vanc-87**

**Vanc-87:** ^1^H NMR (300 MHz, DMSO-d6) δ 8.60 (d, *J* = 8.1 Hz, 2H), 8.46 (s, 1H), 8.29 (s, 2H), 8.12 (s, 1H), 7.87 (d, *J* = 7.5 Hz, 3H), 7.79 (s, 1H), 7.58 (d, *J* = 7.2 Hz, 2H), 7.46 (dt, *J* = 14.7, 7.9, 7.9 Hz, 5H), 7.39 – 7.31 (m, 4H), 7.30 – 7.21 (m, 2H), 6.88 (s, 1H), 6.81 – 6.63 (m, 4H), 6.44 (d, *J* = 7.7 Hz, 2H), 6.05 (d, *J* = 8.4 Hz, 1H), 5.75 (d, *J* = 8.4 Hz, 1H), 5.54 (s, 1H), 5.34 – 5.13 (m, 6H), 4.89 (s, 1H), 4.73 – 4.64 (m, 2H), 4.49 – 4.35 (m, 6H), 4.25 (d, *J* = 12.2 Hz, 2H), 4.06 (dd, *J* = 16.4, 6.3 Hz, 4H), 3.82 – 3.65 (m, 4H), 3.60 – 3.41 (m, 5H), 3.30 – 3.28 (m, 4H), 3.18 (s, 1H), 3.10 – 3.03 (m, 1H), 2.32 (s, 1H), 1.97 – 1.84 (m, 2H), 1.79 – 1.64 (m, 2H), 1.55 – 1.35 (m, 2H), 1.29 (s, 3H), 1.07 (d, *J* = 6.0 Hz, 3H), 0.90 (d, *J* = 6.4 Hz, 3H), 0.86 (d, *J* = 6.5 Hz, 3H).

**Vanc-88**

**Vanc-88:** ^1^H NMR (600 MHz, DMSO-d6) δ 10.11 (s, 1H), 9.10 (s, 1H), 8.65 (s, 1H), 8.53 (s, 1H), 8.45 (s, 1H), 8.31 (s, 2H), 8.22 (s, 1H), 8.07 (s, 1H), 7.87 (s, 1H), 7.78 – 7.70 (m, 4H), 7.60 (t, *J* = 7.6, 7.6 Hz, 2H), 7.50 (t, *J* = 9.5, 9.5 Hz, 2H), 7.36 – 7.32 (m, 4H), 7.29 – 7.25 (m, 2H), 6.88 (s, 1H), 6.79 (d, *J* = 8.8 Hz, 1H), 6.72 (t, *J* = 9.9, 9.9 Hz, 3H), 6.41 (s, 1H), 6.37 (s, 1H), 5.77 (d, *J* = 8.1 Hz, 1H), 5.55 (s, 1H), 5.33 (s, 1H), 5.29 (d, *J* = 7.6 Hz, 1H), 5.24 (s, 2H), 5.16 (d, *J* = 4.3 Hz, 1H), 4.89 (s, 1H), 4.69 (q, *J* = 6.8, 6.6, 6.6 Hz, 1H), 4.48 (d, *J* = 5.4 Hz, 1H), 4.39 (d, *J* = 5.1 Hz, 2H), 4.30 (d, *J* = 11.8 Hz, 1H), 4.10 (dd, *J* = 16.3, 6.0 Hz, 2H), 3.95 (dd, *J* = 16.4, 5.7 Hz, 2H), 3.69 (d, *J* = 10.6 Hz, 2H), 3.58-3.52 (m, 6H), 3.46 (t, *J* = 8.8, 8.8 Hz, 4H), 3.32-3.26 (m, 4H), 3.18 (s, 1H), 3.06 (t, *J* = 7.3, 7.3 Hz, 1H), 2.32 (s, 3H), 2.16 (dd, *J* = 16.3, 5.8 Hz, 1H), 1.94 – 1.87 (m, 1H), 1.76-1.70 (m, 2H), 1.49 (dt, *J* = 13.6, 6.8, 6.8 Hz, 1H), 1.42 (dt, *J* = 13.8, 7.2, 7.2 Hz, 1H), 1.29 (s, 3H), 1.08 (d, *J* = 6.4 Hz, 3H), 0.91 (d, *J* = 6.6 Hz, 3H), 0.86 (d, *J* = 6.6 Hz, 3H).

**Vanc-89**

**Vanc-89:** ^1^H NMR (600 MHz, DMSO-d6) δ 9.81 (s, 1H), 9.14 (s, 2H), 8.65 (s, 1H), 8.54 (s, 1H), 8.51 (s, 1H), 8.42 (s, 1H), 8.32 (s, 2H), 8.23 (s, 1H), 7.87 (s, 1H), 7.80 (d, *J* = 8.5 Hz, 2H), 7.73 (d, *J* = 8.6 Hz, 2H), 7.63-7.58 (m, 1H), 7.51 (t, J = 7.54, 7.54 Hz, 2H), 7.35 (d, *J* = 8.4 Hz, 2H), 7.27 (d, *J* = 8.6 Hz, 2H), 6.89 (s, 1H), 6.79 (t, *J* = 9.0, 9.0 Hz, 2H), 6.73 (d, *J* = 8.4 Hz, 1H), 6.67 (s, 1H), 6.41 (s, 1H), 6.37 (s, 1H), 5.77 (d, *J* = 7.7 Hz, 1H), 5.55 (s, 1H), 5.33 (s, 1H), 5.29 (d, *J* = 7.6 Hz, 1H), 5.24 (s, 2H), 5.16 (s, 1H), 4.89 (s, 1H), 4.73 – 4.65 (m, 2H), 4.48 (s, 1H), 4.38 (t, *J* = 6.9, 6.9 Hz, 4H), 4.32 (s, 1H), 4.13 – 4.04 (m, 2H), 4.02 – 3.89 (m, 2H), 3.69 (d, *J* = 10.2 Hz, 2H), 3.60 – 3.51 (m, 4H), 3.49 – 3.43 (m, 2H), 3.32-3.26 (m, 4H), 3.17 (s, 1H), 3.09 – 2.98 (m, 1H), 2.32 (s, 3H), 2.19 – 2.12 (m, 1H), 1.91-1.84 (m, 2H), 1.76-1.69 (m, 2H), 1.49 (dt, *J* = 13.5, 6.8, 6.8 Hz, 1H), 1.41 (dt, *J* = 13.9, 7.4, 7.4 Hz, 1H), 1.29 (s, 3H), 1.08 (s, 3H), 0.91 (d, *J* = 6.5 Hz, 3H), 0.87 (d, *J* = 6.0 Hz, 3H).

**Vanc-90**

**Vanc-90:** ^1^H NMR (300 MHz, DMSO-d6) δ 9.71 (s, 1H), 8.65 (s, 1H), 8.49 (s, 1H), 8.31 (s, 2H), 8.21 (s, 1H), 7.86 (s, 1H), 7.76 (s, 1H), 7.48 (s, 4H), 7.40 – 7.15 (m, 10H), 6.94 (d, *J* = 7.8 Hz, 1H), 6.88 (s, 1H), 6.82 – 6.60 (m, 4H), 6.40 (s, 1H), 6.34 (s, 1H), 5.76 (d, *J* = 8.7 Hz, 1H), 5.54 (s, 1H), 5.35 – 5.14 (m, 7H), 4.88 (s, 1H), 4.68 (d, *J* = 6.8 Hz, 2H), 4.50 – 4.24 (m, 6H), 4.08-4.0 (m, 2H), 3.92 (s, 2H), 3.68 (d, *J* = 10.8 Hz, 2H), 3.58-3.43 (m, 7H), 3.27 (d, *J* = 7.3 Hz, 2H), 3.16 (s, 1H), 3.05 (t, J = 7.12, 7.12 Hz, 1H), 2.31 (s, 3H), 2.19 – 2.11 (m, 1H), 1.89 (s, 2H), 1.79 – 1.64 (m, 2H), 1.54 – 1.35 (m, 2H), 1.27 (s, 3H), 1.06 (d, *J* = 6.3 Hz, 3H), 0.90 (d, *J* = 6.5 Hz, 3H), 0.86 (d, *J* = 6.5 Hz, 3H).

**Vanc-91**

**Vanc-91:** ^1^H NMR (300 MHz, DMSO-d6) δ 9.82 (s, 1H), 8.64 (s, 1H), 8.54 (bs, 1H), 8.51 (s, 1H), 8.44 (s, 1H), 8.32 (s, 2H), 7.91 – 7.68 (m, 7H), 7.50 (d, *J* = 8.1 Hz, 2H), 7.35 (d, *J* = 8.2 Hz, 2H), 7.26 (d, *J* = 8.3 Hz, 2H), 6.88 (s, 1H), 6.84 – 6.63 (m, 4H), 6.41 (s, 1H), 6.36 (s, 1H), 5.76 (d, *J* = 8.4 Hz, 1H), 5.54 (s, 1H), 5.32-5.15 (m, 6H), 4.89 (s, 1H), 4.69 (d, *J* = 6.6 Hz, 2H), 4.48 (s, 2H), 4.42 – 4.31 (m, 6H), 4.17 – 3.88 (m, 5H), 3.69 (d, *J* = 10.5 Hz, 2H), 3.61 – 3.37 (m, 5H), 3.28 (d, *J* = 7.2 Hz, 2H), 3.18 (s, 1H), 3.11 – 3.01 (m, 1H), 2.31 (s, 3H), 2.20-2.13 (m, 1H), 1.95 – 1.81 (m, 3H), 1.79 – 1.63 (m, 3H), 1.45 (dt, *J* = 12.9, 6.6, 6.6 Hz, 2H), 1.38 – 1.19 (m, 8H), 1.07 (d, *J* = 6.2 Hz, 3H), 0.90 (d, *J* = 6.2 Hz, 6H), 0.86 (d, *J* = 6.8 Hz, 3H).

**Vanc-92**

**Vanc-92:** ^1^H NMR (600 MHz, DMSO-d6) δ 10.09 (s, 1H), 8.65 (s, 1H), 8.55 (s, 1H), 8.45 (s, 1H), 8.36 (s, 1H), 7.85 (d, *J* = 8.3 Hz, 3H), 7.78 (d, *J* = 8.6 Hz, 2H), 7.73 (d, *J* = 7.4 Hz, 2H), 7.68 (t, *J* = 7.4, 7.4 Hz, 1H), 7.57 (t, *J* = 7.6, 7.6 Hz, 3H), 7.50 (t, *J* = 8.8, 8.8 Hz, 2H), 7.38 – 7.33 (m, 3H), 7.27 (d, *J* = 10.6 Hz, 2H), 6.91 – 6.83 (m, 2H), 6.79 (d, *J* = 8.6 Hz, 1H), 6.73 (d, *J* = 8.4 Hz, 1H), 6.41 (s, 1H), 6.38 (s, 1H), 5.76 (d, *J* = 7.9 Hz, 1H), 5.54 (s, 1H), 5.33 – 5.29 (m, 3H), 5.23 (d, *J* = 7.1 Hz, 3H), 5.15 (s, 1H), 4.88 (s, 1H), 4.68 (q, *J* = 6.9, 6.7, 6.7 Hz, 2H), 4.48 (d, *J* = 3.3 Hz, 1H), 4.38 (d, *J* = 4.7 Hz, 2H), 4.32-4.27 (m, 1H), 4.10 (dd, *J* = 16.4, 5.4 Hz, 2H), 4.04 – 3.96 (m, 2H), 3.69 (d, *J* = 10.2 Hz, 2H), 3.58-3.50 (m, 5H), 3.48 – 3.40 (m, 8H), 3.27-3.23 (m, 4H), 3.10 (s, 1H), 3.04 (t, *J* = 7.2, 7.2 Hz, 1H), 2.31 (s, 3H), 2.15 (d, *J* = 10.7 Hz, 1H), 1.84 (d, *J* = 9.3 Hz, 1H), 1.77 – 1.63 (m, 3H), 1.45 (ddt, *J* = 45.5, 14.3, 7.3, 7.3 Hz, 3H), 1.25 (s, 3H), 1.07 (d, *J* = 6.2 Hz, 3H), 0.91 (d, *J* = 6.5 Hz, 6H), 0.87 (d, *J* = 6.5 Hz, 3H).

**Vanc-B**

**Vanc-B:** ^1^H NMR (300 MHz, DMSO-d6) δ 8.74 (t, *J* = 6.0, 6.0 Hz, 1H), 8.53 (d, *J* = 42.8 Hz, 2H), 8.31 (s, 2H), 8.07 (s, 1H), 7.97 (d, *J* = 8.3 Hz, 2H), 7.93 – 7.71 (m, 5H), 7.59 (d, *J* = 8.2 Hz, 2H), 7.55 – 7.19 (m, 5H), 6.81 – 6.61 (m, 2H), 6.32 (d, *J* = 26.6 Hz, 1H), 5.74 (d, *J* = 6.7 Hz, 1H), 5.54 (s, 1H), 5.42 – 5.09 (m, 5H), 4.88 (s, 1H), 4.68 (d, *J* = 6.8 Hz, 1H), 4.53 – 4.16 (m, 6H), 3.79 – 3.33 (m, 13H), 3.23 (d, *J* = 34.8 Hz, 5H), 3.05 (t, *J* = 6.1, 6.1 Hz, 4H), 2.31 (s, 3H), 2.22 – 1.83 (m, 1H), 1.81 – 1.60 (m, 3H), 1.49 (s, 2H), 1.28 (s, 2H), 1.06 (d, *J* = 6.1 Hz, 3H), 0.88 (dd, *J* = 12.9, 6.5 Hz, 6H).

**Vanc-C**

**Vanc-C:** ^1^H NMR (300 MHz, DMSO-d6) δ 8.60 (s, 1H), 8.42 (s, 1H), 8.32 (s, 2H), 7.99-7.78 (m, 4H), 7.49-7.24 (m, 7H), 6.88 (s, 1H), 6.72 (dt, J = 14.9, 7.5, 7.5 Hz, 3H), 6.37 (s, 1H), 6.23 (s, 1H), 5.76 (s, 1H), 5.75 (s, 1H), 5.54 (s, 1H), 5.32 – 5.10 (m, 5H), 4.94 – 4.80 (m, 1H), 4.68 (d, J = 6.2 Hz, 2H), 4.40 (dd, J = 21.1, 5.5 Hz, 4H), 4.22 (d, J = 12.9 Hz, 2H), 4.03 (dd, J = 14.3, 7.2 Hz, 2H), 3.68 (d, J = 10.9 Hz, 2H), 3.61 – 3.39 (m, 6H), 3.34 – 3.15 (m, 4H), 3.11 – 3.01 (m, 1H), 2.89 (s, 1H), 2.79 (t, J = 2.3, 2.3 Hz, 1H), 2.73 (s, 1H), 2.31 (s, 3H), 2.20-2.12 (m, 3H), 1.89 (s, 1H), 1.78 – 1.59 (m, 4H), 1.40 (s, 1H), 1.28 (s, 2H), 1.06 (d, J = 6.1 Hz, 3H), 0.88 (dd, J = 13.1, 6.5 Hz, 6H).

**Vanc-F**

**Vanc-F:** ^1^H NMR (300 MHz, DMSO-d6) δ 8.70 – 7.76 (m, 11H), 7.62 – 7.19 (m, 8H), 6.97 – 6.64 (m, 5H), 6.38 (s, 1H), 6.19 (s, 1H), 5.74 (d, J = 8.4 Hz, 1H), 5.54 (s, 1H), 5.46 – 5.09 (m, 7H), 4.78 (dd, J = 62.1, 6.2 Hz, 3H), 4.44-4.24 (m, 4H), 3.78 – 3.39 (m, 2H), 3.36 – 2.95 (m, 5H), 2.78-2.66 (m, 2H), 2.45 – 2.23 (m, 11H), 2.16-1.67 (m, 3H), 1.56 – 1.36 (m, 1H), 1.29 (s, 3H), 1.06 (d, J = 5.6 Hz, 3H), 0.88 (dd, J = 13.6, 6.7 Hz, 9H), 0.76 (s, 3H).

**Vanc-N**

**Vanc-N:** ^1^H NMR (600 MHz, D_2_O) δ 8.35 (s, 2H), 7.84 – 7.37 (m, 7H), 7.24 (d, *J* = 65.5 Hz, 2H), 6.98 (d, *J* = 57.5 Hz, 2H), 6.56-6.48 (m, 2H), 5.66 (s, 1H), 5.54 – 5.23 (m, 5H), 5.07 (s, 1H), 4.83 (s, 1H), 4.62 (s, 2H), 4.38 (s, 1H), 4.15-4.07 (m, 3H), 3.71 (s, 3H), 3.55 (d, *J* = 29.7 Hz, 2H), 3.39 (s, 1H), 3.28 (s, 3H), 2.72 (s, 3H), 2.63-2.61 (m, 4H), 2.05-1.99 (m, 1H), 1.75 (s, 1H), 1.65 (d, *J* = 37.3 Hz, 2H), 1.38 (s, 3H), 1.13 (s, 3H), 0.87 (dd, *J* = 23.6, 6.4 Hz, 6H).

**Vanc-S**

**Vanc-S:** ^1^H NMR (300 MHz, DMSO-d6) δ 8.75 (t, *J* = 5.9, 5.9 Hz, 1H), 8.60 (s, 1H), 8.47 (s, 1H), 8.31 (s, 2H), 8.22 (s, 1H), 8.08 (s, 1H), 7.97 (d, *J* = 8.3 Hz, 2H), 7.92 – 7.69 (m, 6H), 7.59 (d, *J* = 8.2 Hz, 2H), 7.56 – 7.18 (m, 5H), 6.88-6.68 (m, 3H), 6.32 (d, 1H), 5.75 (d, *J* = 8.4 Hz, 1H), 5.54 (s, 1H), 5.40 – 5.11 (m, 5H), 4.89 (s, 1H), 4.68 (d, *J* = 6.8 Hz, 1H), 4.45-4.29 (m, 6H), 3.76- 3.34 (m, 5H), 3.36 – 2.98 (m, 6H), 3.07 (t, *J* = 7.3, 7.3 Hz, 1H), 2.32 (s, 3H), 2.19 – 2.09 (m, 1H), 1.75 – 1.68 (m, 3H), 1.49 – 1.38 (m, 1H), 1.29 (s, 2H), 1.06 (d, *J* = 6.1 Hz, 2H), 0.88 (dd, *J* = 13.0, 6.5 Hz, 6H).

**References**.

1. Cakici M., Catir, M., Karabuga, S., Ulukanli S and Kilic, H. (2011). Synthesis and asymmetric catalytic activity of (1S,1′S)-4,4′-biquinazoline-based primary amines. Tetrahedron Asymm. 22, 300-308.
2. Wang, J., Liang, Y.-L. And Qu, J. (2009). Boiling water-catalyzed neutral and selective N-Boc deprotection. Chem. Commun. 0, 5144–5146.
3. Gu, Z., Li, Z., Liu, Z., Wang, Y., Liu, C. and Xiang, J. (2008). Simple, efficient copper-free Sonogashira coupling of haloaryl carboxylic acids or unactivated aryl bromides with terminal alkynes. Catal. Commun. 9, 2154–2157.
4. Sun, B., Hoshino, J., Jermihov, K., Marler, L., Pezzuto, J.M., Mesecar, A.D. and Cushman, M. (2010). Design, synthesis, and biological evaluation of resveratrol analogues as aromatase and quinone reductase 2 inhibitors for chemoprevention of cancer. Bioorg. Med. Chem. 18, 5352–5366.
5. Zhu, W. and Ma, D. (2004). Chem Commun. Synthesis of aryl azides and vinyl azides via proline-promoted CuI-catalyzed coupling reactions. 888-889.
6. Yarlagadda, V., Akkapeddi, P., Manjunath, G.B. and Haldar, J.J. (2014). Membrane Active Vancomycin Analogues: A Strategy to Combat Bacterial Resistance. J. Med Chem 57,4558−4568.

**Acknowledgement.**

The publication was financially supported by The Ministry of Education and Science of the Russian Federation (Agreement number 02.a03.0008).
